# Supplementary material for: A photoconversion model for full spectral programming and multiplexing of optogenetic systems
Source: Mol Syst Biol. 2017 Apr 24;13(4):926. doi: 10.15252/msb.20167456 (PMC5408778; doi:10.15252/msb.20167456)
Supplement: Supplementary file 1 — Appendix [file MSB-13-926-s001.pdf]

APPENDIX

A photoconversion model for full spectral programming and multiplexing of optogenetic systems

Evan J. Olson[1], Constantine N. Tzouanas[1], Jeffrey J. Tabor[1,2]

[1]Department of Bioengineering, Rice University, 6100 Main Street, Houston, Texas 77005, United States
[2]Department of Biosciences, Rice University, 6100 Main Street, Houston, Texas 77005, United States

Appendix Figure S1 – **1**
Alternative representations of CcaSR dynamic training experiments.

Appendix Figure S2 – **2**
Alternative representations of CcaSR spectral training experiments.

Appendix Figure S3 – **3**
Residuals of CcaSR model to the training data.

Appendix Figure S4 – **4**
Multicollinearity in the model fit results.

Appendix Figure S5 – **5**
Detailed results of best-fit splines.

Appendix Figure S6 – **6**
Spline knot number optimization via LOOCV.

Appendix Figure S7 – **7**
CcaSR dynamic validation results including cell culture density measurements.

Appendix Figure S8 – **8**
Final cell density measurements for CcaSR spectral characterization measurements.

Appendix Figure S9 – **9**
Alternative representations of Cph8-OmpR dynamic training experiments.

Appendix Figure S10 – **10**
Alternative representations of Cph8-OmpR spectral training experiments.

Appendix Figure S11 – **11**
Residuals of Cph8-OmpR model to the training data.

Appendix Figure S12 – **12**
Dual-system fluorescent reporter bleedthrough compensation.

Appendix Figure S13 – **13**
Optogenetic strain growth rate measurements.

Appendix Figure S14 – **14**
Comparison of response of single- vs. dual-system Cph8-OmpR to red light.

Appendix Supplementary Method S1 – **15**
Detailed bacterial growth and light exposure protocol.

Appendix Supplementary Method S2 – **18**
Detailed -80 °C preculture aliquot protocol.

Appendix Supplementary Method S3 – **19**
Detailed LED measurement protocol.

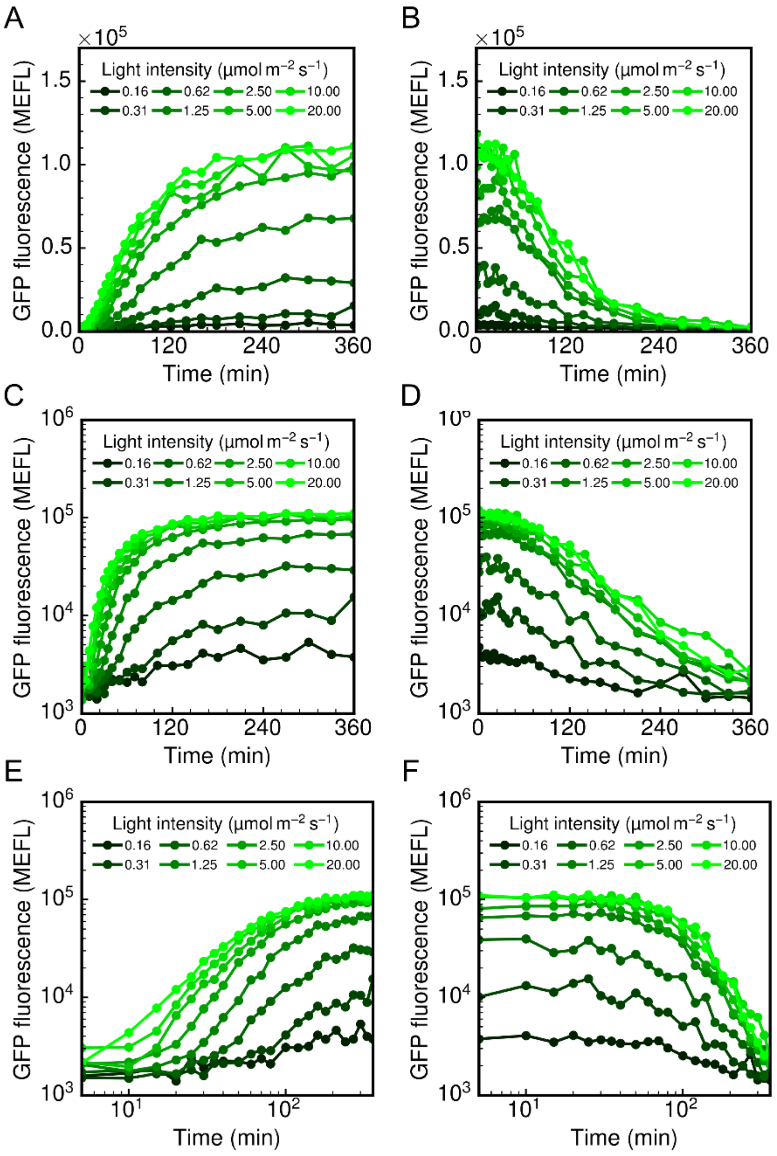

Appendix Figure S1 – Alternative representations of CcaSR dynamic training experiments. (A,C,E) Activating and (B,D,F) deactivating step response dynamics are shown with (A-B) linear axes, (C-D) semilog axes, and (E-F) log-log axes. Each data point represents the arithmetic mean of a single population of cells. Lines are linear interpolations between points, and are simply a guide to the eye. Data and analysis scripts available in **Dataset EV2**.

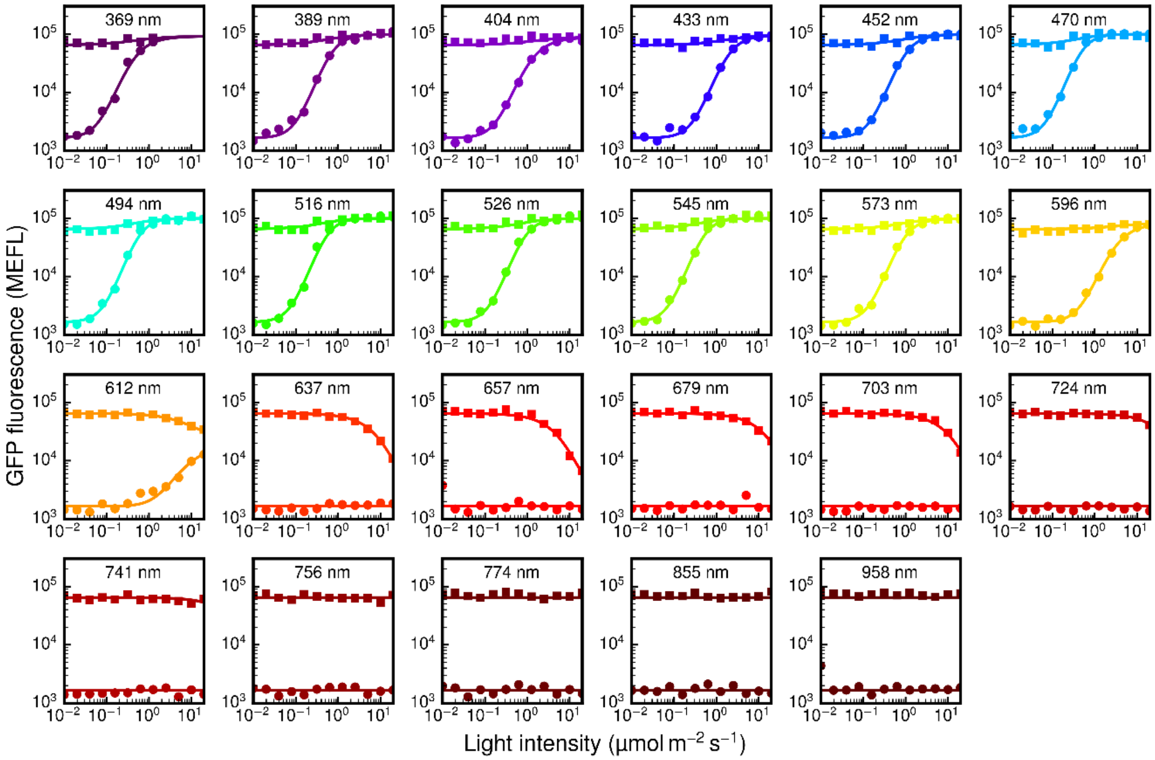

Appendix Figure S2 – Alternative representations of CcaSR spectral training experiments. Results of the spectral characterization experiments are shown LED-by-LED on log-log axes. Each plot shows the forward activation spectrum (circles) and reverse activation spectrum (squares) for each LED (centroid wavelength indicated). Each data point represents the arithmetic mean of a single population of cells. Lines are simulated results of the best-fit model. Data and analysis scripts available in **Dataset EV2**.

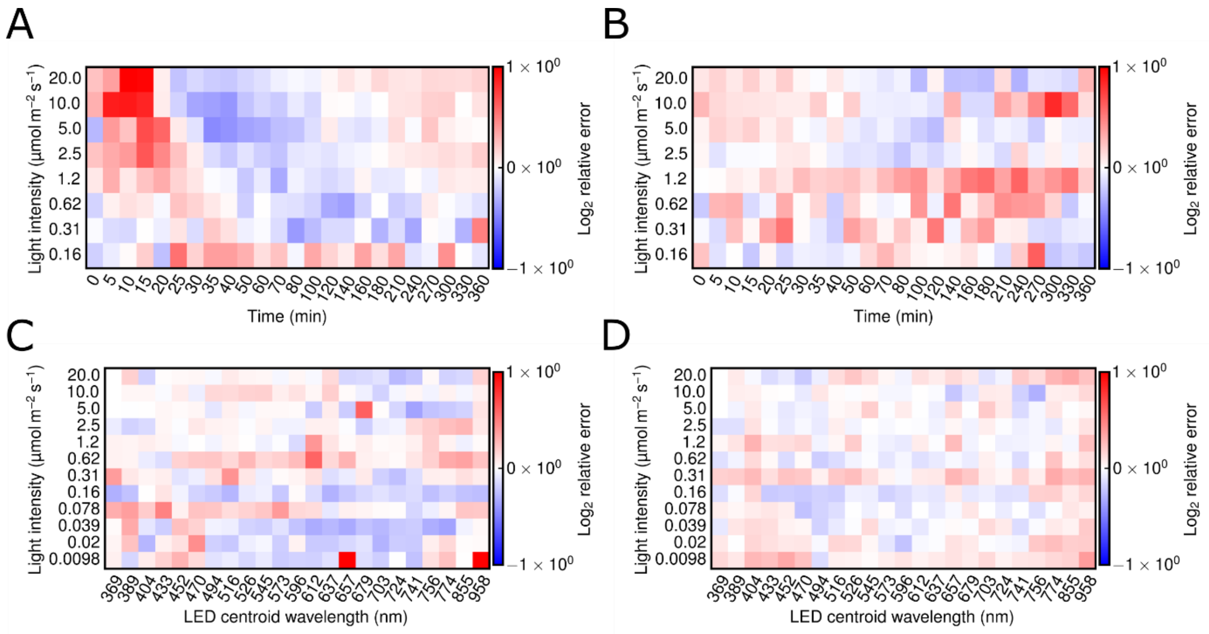

Appendix Figure S3 – Residuals of CcaSR model to the training data. Residuals between the data and the model are shown for the (A) activating and (B) deactivating step-responses as well as the (C) forward and (D) reverse spectral measurements. The residuals are expressed relative to the measured fluorescence data on a log (base 2) scale (i.e. $\log_2 F_{\mathrm{data}}/F_{\mathrm{model}}$). Data and analysis scripts available in **Dataset EV2**.

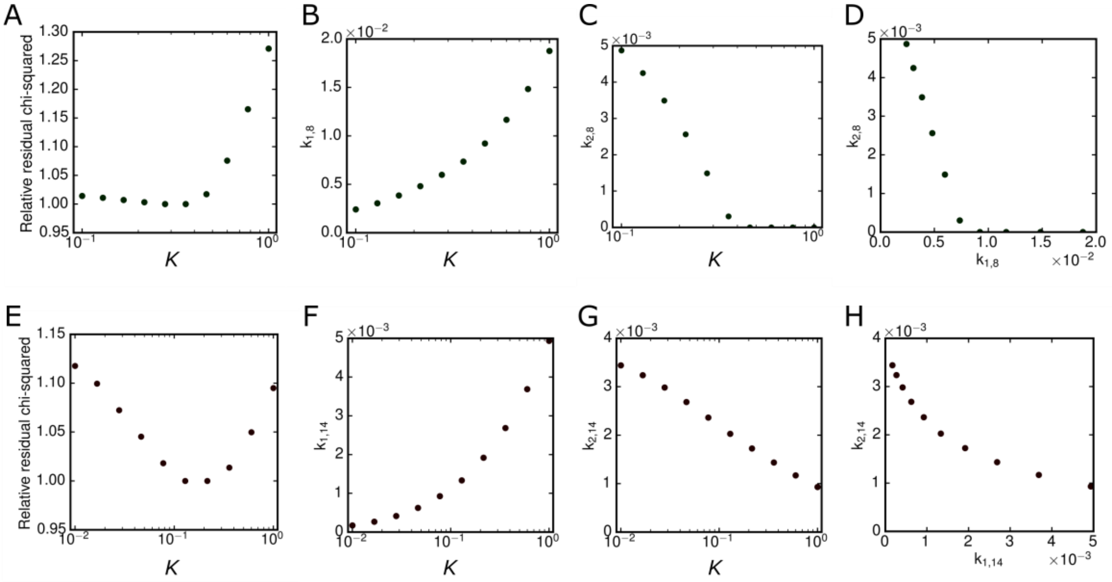

Appendix Figure S4 – Multicollinearity in the model fit results. The ability of the model parameters to compensate for one another was examined by repeatedly performing the model regression to the data while holding the *K* parameter fixed as an independent variable to a range of values. The CcaSR (A-D) and Cph8-OmpR (E-H) results are shown. The The quality of the fit relative to the best-fit models are shown (A,E), as well as the relationship between the forward (B,F) and reverse (C,G) photoconversion rates. For the CcaSR system, the photoconversion rates for the green LED (λ_C=526 nm, $k_{i,8}$) are shown, and for the Cph8-OmpR system, the red (λ_C=657 nm, $k_{i,14}$) is used. Finally, the forward and reverse rates are plotted against each other (D,H). Data and analysis scripts available in **Dataset EV2**.

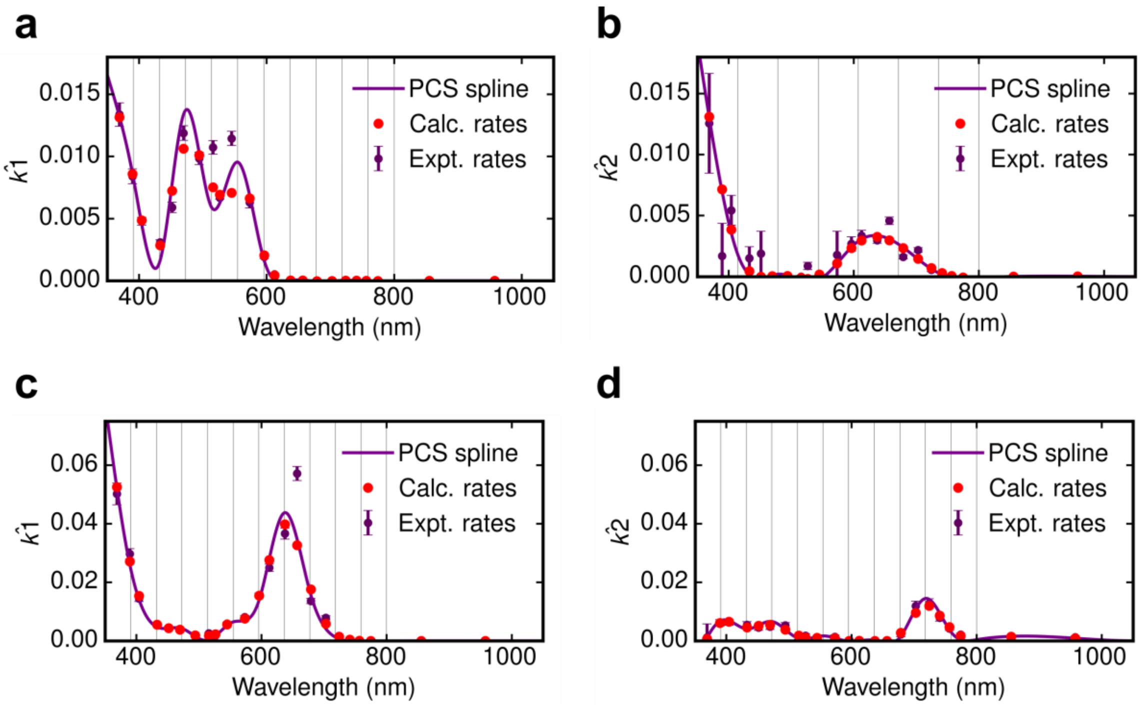

Appendix Figure S5 – Detailed results of best-fit splines. Spline results and photoconversion rates are shown for CcaSR (A-B) and Cph8-OmpR (C-D). Experimental unit photoconversion rates (blue, $\hat{k}_i^{\text{expt.}}$) are shown with errorbars indicating the uncertainty via the standard error of the model fits. The PCS spline estimate (green, $\sigma_i^{\text{est.}}(\lambda)$) is constructed by minimizing the sum of the squared error (weighted by the experimental uncertainty) between the experimental and spline-derived (calculated) rates. The spline-derived, estimated rates are calculated via $\hat{k}_i^{\text{est.}} = \int \sigma_i^{\text{est.}}(\lambda) \cdot n_{\text{light}}(\lambda) \text{d}\lambda$. Spline construction scripts available in **Dataset EV5**.

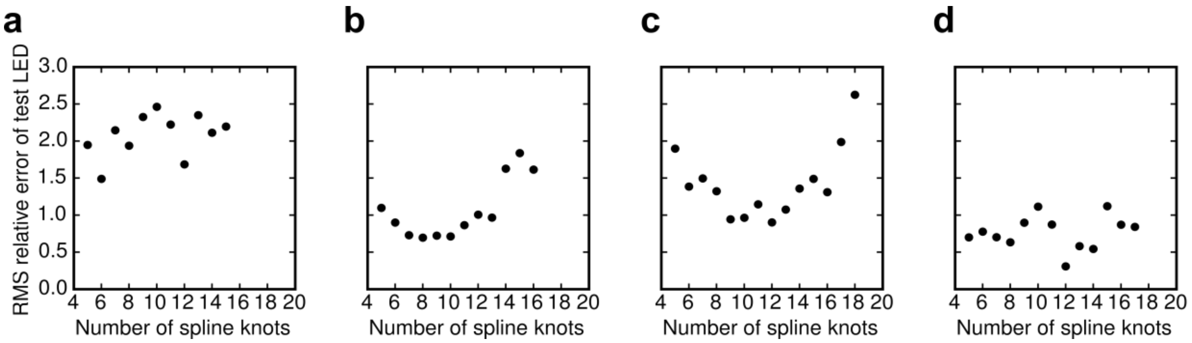

Appendix Figure S6 – Spline knot number optimization via LOOCV. To determine the appropriate spline complexity (i.e. number of knots) for each PCS estimate spline, we used a "Leave-one-out cross-validation (LOOCV)" approach. In this approach, the experimental dataset is split into a series of training and test LED datasets, each of which is constructed by pulling a single LED out of the training set to be used for testing. Then, for each number of spline knots to evaluate, we construct a spline to each training set (the spline knots are evenly distributed from 350 to 800 nm), predict the remaining test LED, and calculate the RMS of the relative errors of the predictions. The RMS errors for each level of spline complexity are shown for CcaSR forward (A) and reverse (B) spectra and for Cph8-OmpR forward (C) and reverse (D) spectra. Spline construction scripts available in **Dataset EV5**.

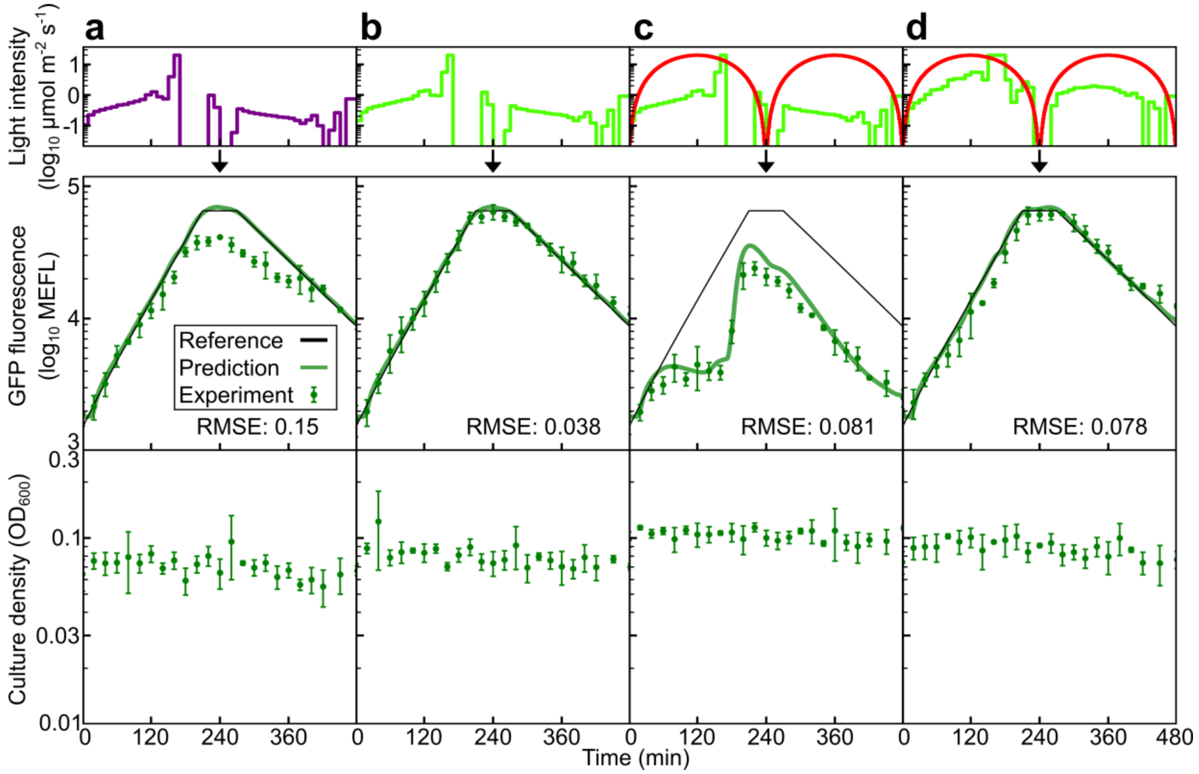

Appendix Figure S7 – CcaSR dynamic validation results including cell culture density measurements. The data shown are extended versions of the main-text **Fig 4**, incorporating the results of the cell culture density measurements of each sample.

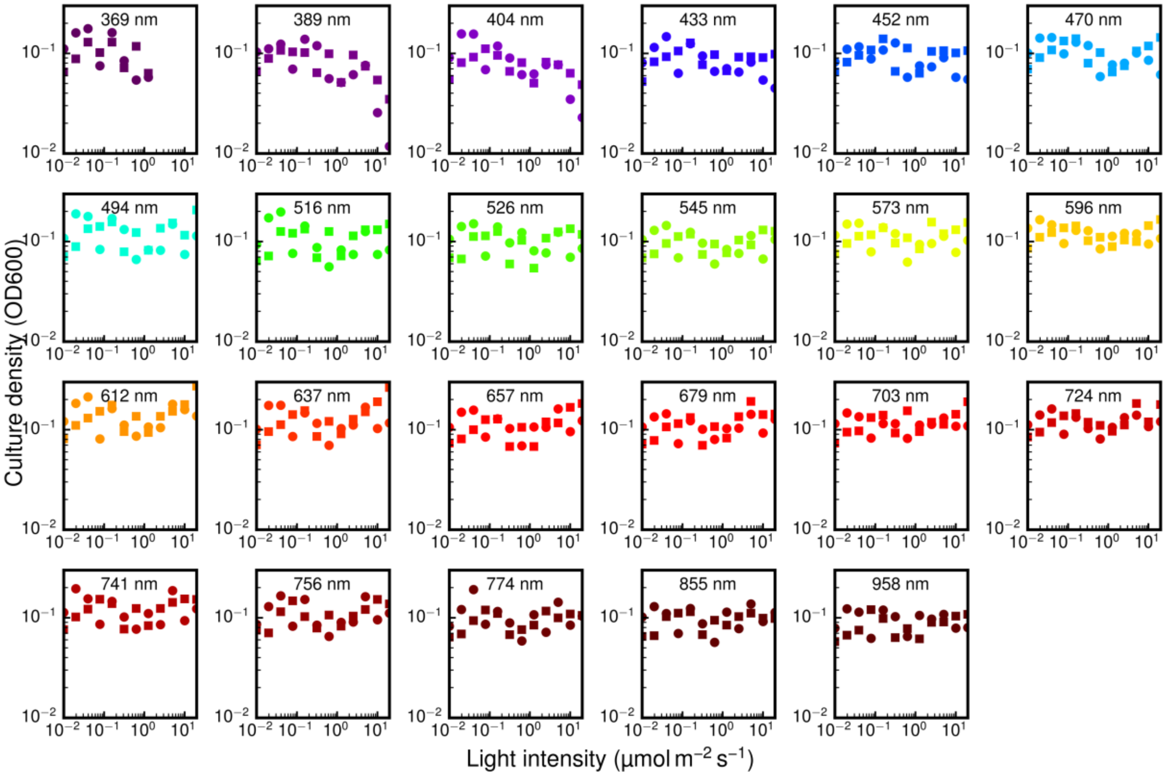

Appendix Figure S8 – Final cell density measurements for CcaSR spectral characterization measurements. Each plot shows the final culture density for forward activation spectrum (circles) and reverse activation spectrum (squares) data for each LED (centroid wavelength indicated). The data shown are the cell culture absorbance measurements (OD600) of the same samples shown in Fig **2D-E** and **Fig S2**.

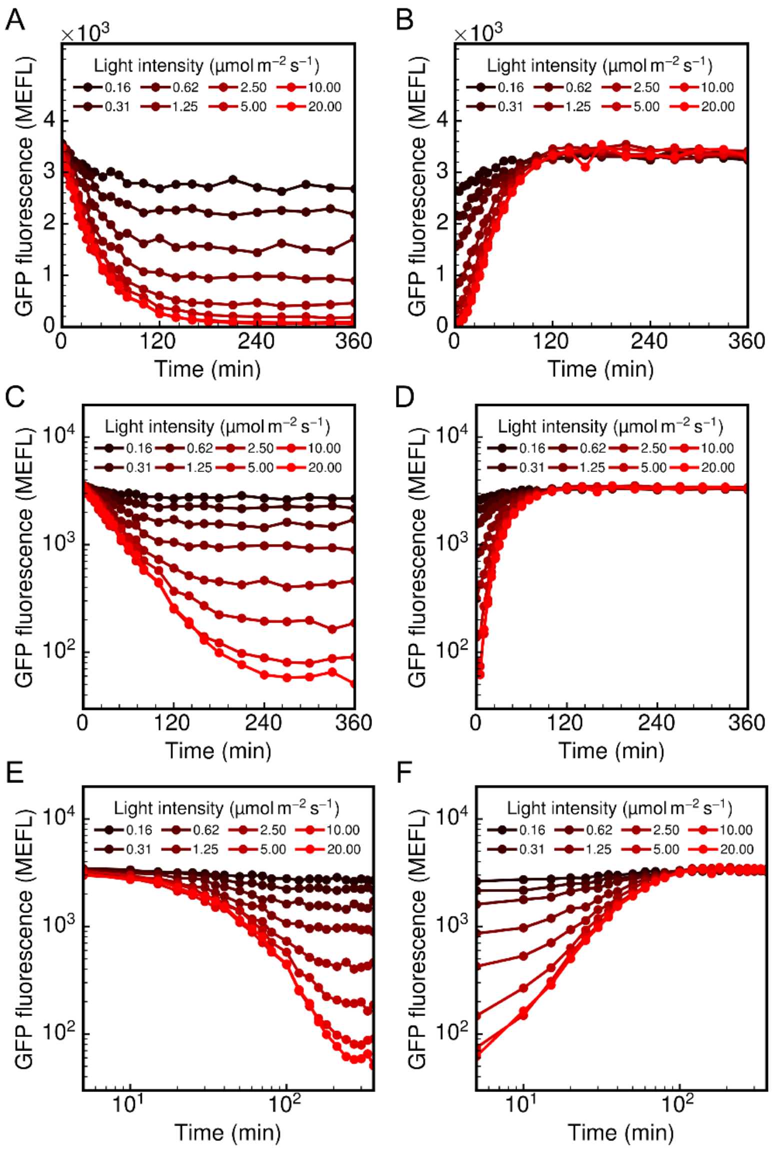

Appendix Figure S9 – Alternative representations of Cph8-OmpR dynamic training experiments. (A,C,E) Activating and (B,D,F) deactivating step response dynamics are shown with (A-B) linear axes, (C-D) semilog axes, and (E-F) log-log axes. Each data point represents the arithmetic mean of a single population of cells. Lines are linear interpolations between points, and are simply a guide to the eye. Data and analysis scripts available in **Dataset EV7**.

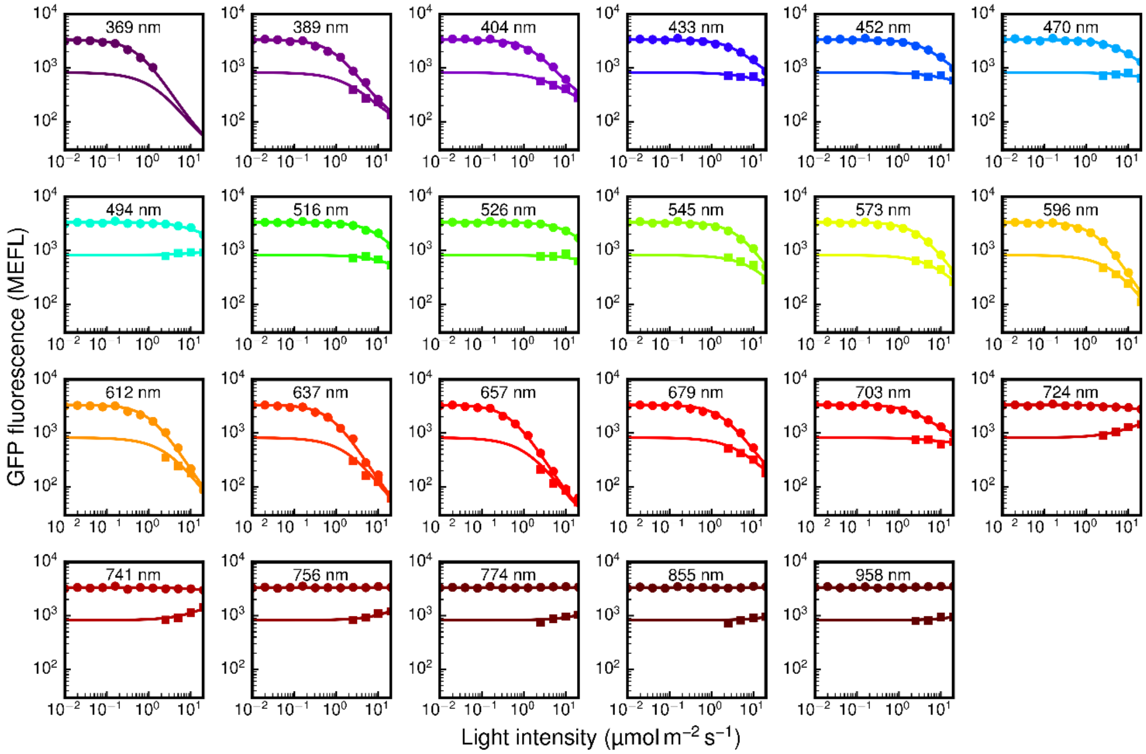

Appendix Figure S10 – Alternative representations of Cph8-OmpR spectral training experiments. Results of the spectral characterization experiments are shown LED-by-LED on log-log axes. Each plot shows the forward activation spectrum (circles) and reverse activation spectrum (squares) for each LED (centroid wavelength indicated). Each data point represents the arithmetic mean of a single population of cells. Lines are simulated results of the best-fit model. Data and analysis scripts available in **Dataset EV7**.

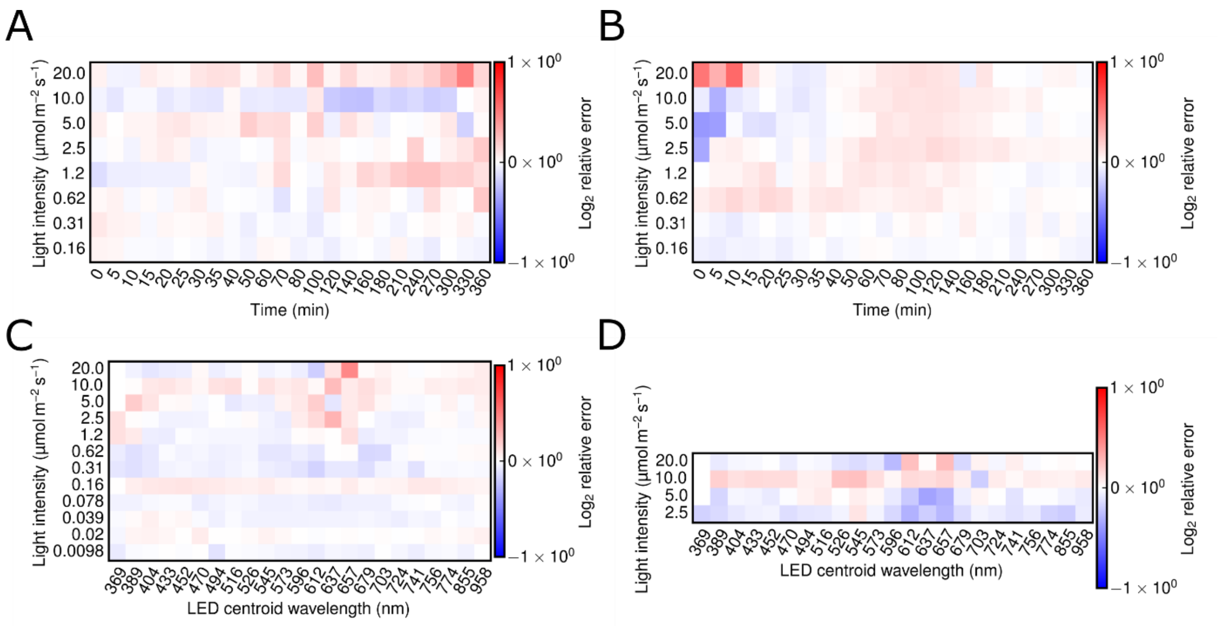

Appendix Figure S11 – Residuals of Cph8-OmpR model to the training data. Residuals between the data and the model are shown for the (A) activating and (B) deactivating step-responses as well as the (C) forward and (D) reverse spectral measurements. The residuals are expressed relative to the measured fluorescence data on a log (base 2) scale (i.e. $\log_2 F_{\mathrm{data}}/F_{\mathrm{model}}$). Data and analysis scripts available in **Dataset EV7**.

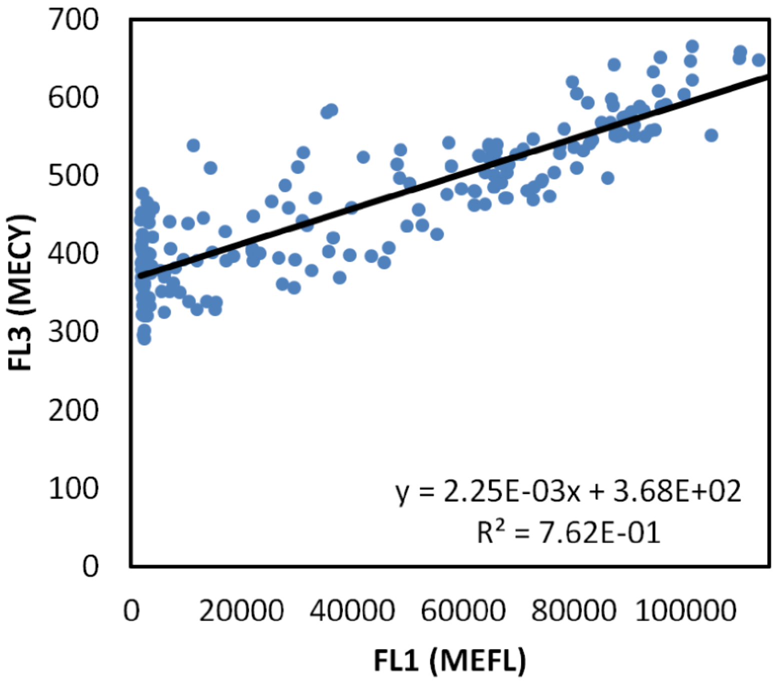

Appendix Figure S12 – Dual-system fluorescent reporter bleedthrough compensation. High intracellular concentrations of sfGFP produce signal in the red-shifted long-pass filter cytometer channel (FL3) typically used for mCherry readout. To compensate for this bleedthrough, the “rgv2_r01” experimental data (**Dataset EV2**), containing a wide range of sfGFP expression levels (and no mCherry) was analyzed and a linear relationship between FL3 and FL1 (the green-shifted channel used for sfGFP) was identified (**Dataset EV8**). A linear fit to the data was performed, and the fit is used to compensate and blank the FL3 channel to enable quantification of the mCherry signal. The compensation function used was $mCherry = \mathrm{FL3} - 368\ \mathrm{MECY} - \mathrm{FL1} \cdot 2.25{\times}10^{-3}\mathrm{MECY/MEFL}$.

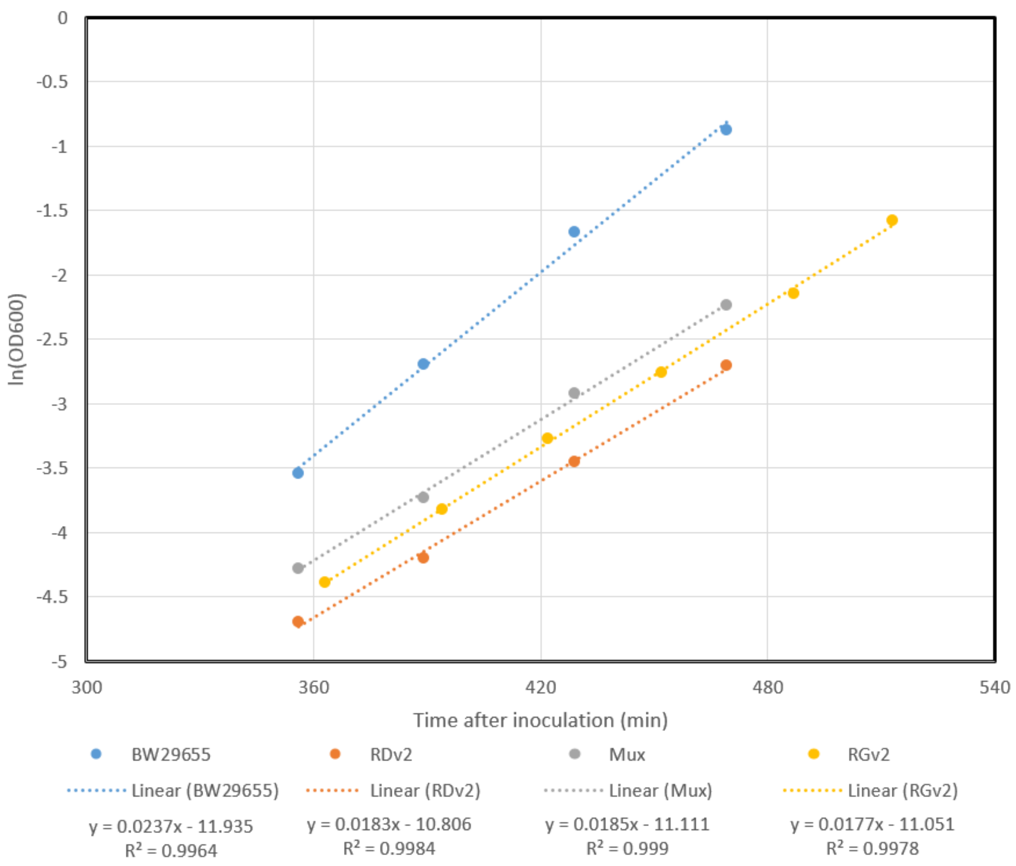

Appendix Figure S13 – Optogenetic strain growth rate measurements. Growth rates of cells containing no plasmids (BW29655), the Cph8-OmpR system (RDv2), the dual-system (Mux), and the CcaSR system (RGv2) were measured by inoculating 8 mL M9 cultures (with appropriate antibiotics, i.e. experimental media) to the initial densities used for experiments (**Method S1**) and later drawing 1 mL samples from these cultures for OD600 measurements in a Cary 50 spectrophotometer. Linear fits were made to semi-logged data in order to extract the exponential growth rates of the strains (linear fits and R^2 values shown below graph).

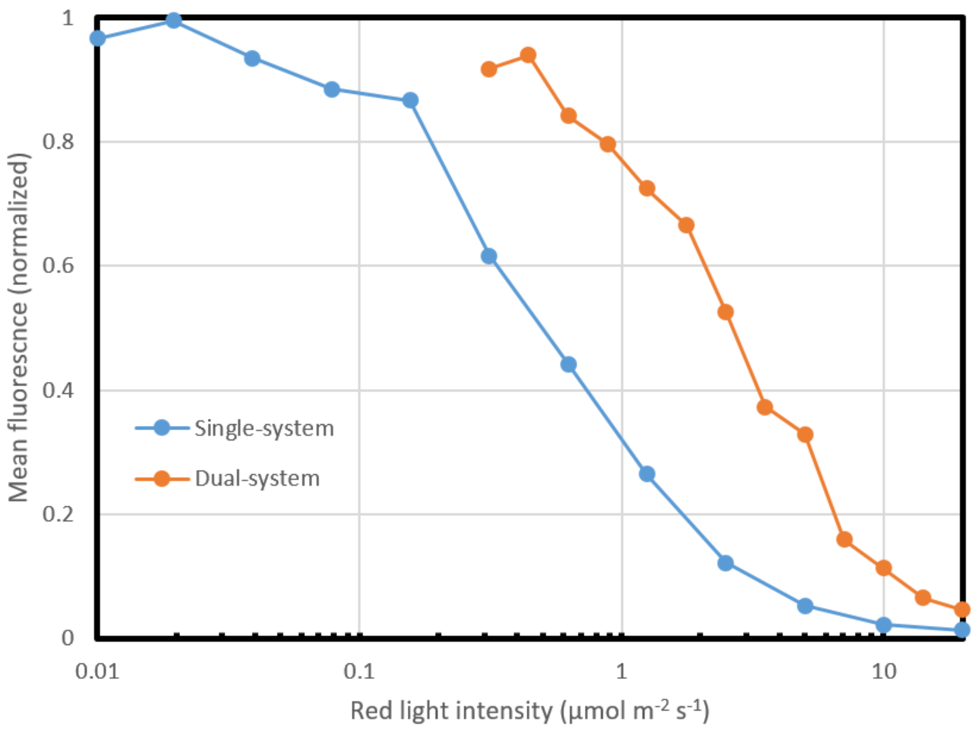

Appendix Figure S14 – Comparison of response of single- vs. dual-system Cph8-OmpR to red light. The decreased sensitivity of the dual-system to red light is made apparent when the normalized responses of the single and dual-system Cph8-OmpR are compared. The normalization is made using the best-fit *a* and *b* values for each system in order to account for the different units and different reporters used by these systems (sfGFP for the single-strain and mCherry for the dual-strain).

Appendix Supplementary Method S1 – Detailed bacterial growth and light exposure protocol.

1. Experiment initialization. This an approximately 2-hour process.

(a) Prepare program files on SD cards and load the SD cards into LPAs.

(b) Prepare 24-well plates for use (ArcticWhite AWLS-303008): Soak plates for at least 15 minutes in 70% EtOH. Triple-rinse the plates using DI water. Place rinsed plates onto a sheet of foil which will later wrap around the plate. Dry plates near a lit burner until the interior of the wells are dry (approximately 45 min). Use a paper wipe to dry the bottom of the plate and the foil. Wrap the plate in the foil.

(c) Prepare media and cell culture: Prepare 100 mL M9 medium (75.8 mL autoclaved, distilled H2O, 20 mL 5x M9 salts, 2mL 10% casamino acids, 2mL 20% glucose, 200 µL 1m MgSO4, 10 µL 1m CaCl2). (Note: prepare 1-2L of this media and pipet it into 50mL aliquots to be used at a later date. For 8x LPA experiments, combine two aliquots.). Add appropriate antibiotics to medium. Shake/stir the container to homogenize. Remove -80 °C culture aliquot from the freezer and allow to thaw. Lightly vortex thawed culture aliquot and briefly spin down in a microcentrifuge. Add appropriate culture volume to the 100mL media. (Note: inoculation densities for strains used in this work are CcaSR: 1E-5, Cph8-OmpR: 5E-6, Dual-system: 1E-5).

(d) Distribute inoculated media into 24-well plates: Prepare a row of 1mL tips in a box so that only every-other tip is present (i.e. 6 tips total). Shake to homogenize inoculated media. Arrange the 8x 24-well plates with the wells open and uncovered. Pour more than half of the media into a 50mL disposable multichannel tray. Use a 1mL 12-channel pipettor with the previously-prepared row of 6 tips to transfer 500 µL of media into each well. Pour remaining media into the tray and continue. Cover each plate with an adhesive-backed foil seal (VWR 60941-126).

(e) Load the LPAs and start the programs: Carry the 8x plates, 8x LPA lids, and 32x LPA wing nuts to the incubator containing the LPAs. (Note: a small carboard box or plastic container is helpful). Load the plates onto each LPA, ensuring that the plate is oriented correctly and is completely engaged with the LPA plate adapter. Place the lid onto each LPA, ensuring that the lid is oriented correctly. Engage 4x LPA wing nuts onto each device. Tighten the nuts evenly until the pressure of the rubber gaskets being compressed is felt (approximately 1-2 full turns after the nut engages with the lid). Start an 8-hour timer while synchronously releasing the reset button on one of the LPAs. Every 10 seconds, release the reset button on another LPA. Make sure to recall the order in which the LPAs were reset. This staggering of the start times enables the plates to be removed immediately when each of their programs ends. Allow the cells to grow in the LPAs at 37 °C for 8 hours.

2. Experiment completion and data collection. This is an approximately 4-hour process.

(a) 30 minutes prior to the end of the LPA programs, prepare a PBS+rifampicin solution: Fill a 250 mL beaker with 125 mL of a PBS solution in the pH7-7.2 range (VWR 72060-035). Add a stir-bar to the beaker and place on a stir-plate. Weigh 62.5 mg of rifampicin (rif, Tokyo

Chemical Industry, R0079). Adjust the beaker to be centered on the stir plate. Adjust the rate of the stir plate to produce a vortex which doesn't quite reach down to the bar. Add the rifampicin to the middle of the vortex. Slide the beaker to be slightly off-center on the stir plate. This often encourages the vortex to lower, leading to the stir-bar clipping the vortex with each rotation. This is the desired state for dissolving. Make any adjustments if necessary. Cover the dissolving solution with an opaque container (e.g. tin can) or foil, as the rif is light-sensitive.

(b) Finishing the LPA programs: Prepare two autoclave trays with ice-water baths. Make sure the water level is near the surface of the ice so that the submerged items will make contact with water and not just ice (Note: a utility cart is useful here). Submerge the 8x multichannel-ready tube racks into one of the icewater baths. Load the 192x wells in these racks with flow cytometry tubes (VWR 60818-419). Carry the other ice-water bath to the incubator with the LPAs. At 2 min time remaining on the LPA programs, open the incubator and unscrew all of the wing nuts (leave the lids in place). As the status LEDs on each LPA change to indicate that the program is complete, remove the enclosed plate and immediately submerge it at least halfway up the side of the plate in the ice-water bath. The temperature logs from the data-logging thermometer on the growth incubator can now be gathered.

(c) Preparing for culture transfers: After a 10 minute wait, remove the rif solution from the stir-plate. The solution should be a vivid, bright orange. If it is dark-colored, either there was light leakage, the stirring was too vigorous, or the saline was not in the pH 7-7.2 range, and the solution should be remade. Filter the rif solution using a 0.22 μm filter. Use a multichannel pipettor to load the 192x cytometry tubes each with 500 μL of the rif solution. Label 2x black-walled clear-bottomed 96-well plates (VWR 82050-748). Prepare 192x 200 μL tips by arranging them in four tip boxes such that every-other tip is present (i.e. rows of 6 tips).

(d) Transferring cultures for measurement: Remove the first 24-well plate from its water bath and place it on a paper towel. Use another towel to dry its top surface and sides. Carefully remove and discard the adhesive foil without spilling the contents of the plate. Use a 12-channel 200 μL pipettor to transfer 100 μL volumes of the cultures into the 96-well plate. Make sure to pipette up-and-down in the 24-well plate in several locations to ensure that the culture is homogenized before this transfer. Do not discard the tips after this transfer. After the transfer to the 96-well plate, using the same tips, transfer the same volume into the corresponding PBS+rif tubes in the tube racks. Discard the tips after this transfer. Place plastic caps onto the PBS+rif tubes containing the cultures. This aids in reducing pipetting errors. Repeat this process until all 192x samples have been transferred to the 96-well plates and the cytometry tubes. It is useful to fill the 96-well plates using an interleaved strategy, where the first 24-well plate begins in A1, the second plate in A2, the third plate in E1, and the fourth plate in E2. Once all samples have been transferred, the cytometry tubes should be homogenized. To do this, take the racks out one-at-a-time, tilt them nearly horizontally, and gently shake the rack. Rotate the rack 180° and repeat. This will cause the PBS+rif to roll up the sides of the tubes, mixing in any culture which was stuck on the sides of the tube.

(e) Preparing for the next experiment (optional): If another experiment is to be started as soon as possible (the 8x LPA growth protocol can be performed twice in a day), the 24-well plates should be cleaned immediately and dried after at least a 15-minute soak in 70% ethanol in water.

(f) Fluorescence maturation and culture OD measurements: Transfer the PBS+rif+culture tube racks into a 37 °C water bath. Allow 1 hour for maturation of fluorescent proteins. While the maturation is in process, use a plate reader to measure the absorbance of the cultures in the 96-well plates. When the fluorescence maturation is complete, remove the tube racks and place them back into an ice-water bath. The samples should remain on ice at least 30 minutes before measurement via flow cytometry. The temperature logs from the data-logging thermometer on the maturation incubator can now be gathered.

(g) Calibrated cytometry measurements: Prepare a bead sample for cytometry calibration. Pipette 500 µL of PBS into a cytometry tube. Add one drop of calibration beads (Spherotech RCP-30-5A) to the PBS. Cap the tube and place it on ice. Transport the PBS+rif+culture samples and the bead sample to the cytometer (modified BD FACScan[1]). Turn the cytometer on and allow it to pressurize and stabilize the laser. If applicable to your cytometer, perform several “Drain/Fill" cycles to clear the lines of any residue leached from the tubing. Also fill and drain the sheath and waste containers if necessary. Analyze the bead sample. The beads should be tightly-clustered in the center of the FSC vs. SSC scatter plot. The gain settings on the fluorescence channels should match the gain used to acquire the cell samples (if measuring a strain for the first time, it is wise to measure the cultures anticipated to have the highest and lowest fluorescence levels to establish an appropriate gain). Collect at least 5,000 events (more is better). Run the cell samples through the cytometer. The cells should be clearly visible on the FSC vs. SSC scatter plot. Collect at least 20,000 events per sample. Make sure that the fluorescence histograms are fully contained within the measurement range at the current gain settings. When complete, perform an instrument shutdown cycle by running 10% bleach and then water through both the droplet-containment line and the sample line.

Appendix Supplementary Method S2 – Detailed -80 °C preculture aliquot protocol.

1. Perform a plasmid transformation to produce your desired strain. From the transformation plate, pick a colony and grow a 3 mL culture in LB media + appropriate antibiotics. Grow to a OD600 of 0.1-0.2. Make a standard 1mL -80 °C glycerol stock from this culture (700 µL culture and 300 µL of 60% glycerol) and freeze the stock overnight.

2. Use a sterile toothpick or pipette tip to stab the glycerol stock and begin growing an 8 mL liquid preculture in a culture tube under experimental growth conditions (i.e. 37 °C for the experiments in this manuscript). It is critical to grow this culture using the same media which is specified for the experiments you will be performing with the aliquots. That is, if your experiments will be in M9+antibiotics, you should grow this culture in M9+antibiotics. Prepare a PCR rack with 48 sterile, opened PCR tubes. Cover the tubes with foil.

3. When the culture begins to exhibit the slightest bit of turbidity, take a 1 mL sample from it and check the density. Check the density again in 30 minutes. Use these measurements to (exponentially) extrapolate the time at which the culture will reach an OD600 of 0.1. If the culture OD600 is already above 0.1, but below 0.2, remove immediately. If the culture is above 0.2, start over with a new preculture.

4. When an OD600 of 0.1-0.2 is reached, remove the tube from the shaker. Do not put the culture on ice. Add a quantity of 60% glycerol to the preculture to bring the final glycerol concentration to 18%. Homogenize the culture by gently vortexing. Use a multichannel pipettor to transfer 100 µL of the preculture into each of the PCR tubes. Close all of the PCR tubes, and transfer the rack to a -80 °C freezer.

5. After 30 minutes, transfer the frozen PCR aliquots into a labeled 50 mL conical tube. Store the conical in the -80 °C freezer.

Appendix Supplementary Method S3 – Detailed LED measurement protocol.

1. Initialize the LPA by programming it with your desired settings. For LED calibration, first maximize all of the parameters for each LED by setting the DC values to 63, GCAL values to 255, and GS sequence to 4095. If a specific final intensity is desired, adjust the DC parameter to produce a measurement which is approximately 10-20% above your desired intensity.

2. Connect all wires to the spectrometer (StellarNet UVN-SR-25 LT16): power to wall-socket, USB to computer, and fiber-optic to the IS6.

3. Open the SpectraWiz software and say “No” to the pop-up dialog named “Confirm” if it shows up. If the software has not been installed, follow the manufacturer instructions for installation.

4. Load the IS6 calibration file. Click “View” → “Radiometer” → “Save or Load Cal File for attached light receptor !” and then click “Yes” to open a file explorer dialog. Select the calibration file (“MyCal-EPP10022524-RAD-IS6.CAL” for our instrument) and click “Open.” Click “OK.” at the dialog that pops up.

5. Switch the instrument to scope mode, which reads out the raw 16-bit values from the light detector. Click “View” → “Scope Mode.”

6. Place the LED into the IS6 LED adapter socket, and screw the adapter onto the IS6. The IS6 LED adapter is the screw-on cap with a wire coming out of the back of it terminated by a barrel-jack power connector. Connect power to the barrel-jack connector using the LPA-side of the power adapter. The LPA-side of the power adapter is the wire terminated in a male barrel-jack on one end and a two-pronged terminal on the other. The two-pronged end fits into the LED socket in the LPA in which the currently-measured LED will be placed. Note: to minimize measurement of back-emission from the LED, a 3d-printable cone which holds the LED in place is available.

7. Adjust the integration time on the spectrometer to maximize the signal from the LED. Click “Setup” → “Detector integration time” and type a value which produces a signal that peaks at approximately 75% of the detector range. Repeat if necessary until the full detector range is utilized. Note: By default the software will be averaging the spectra of 5 independent measurements. To speed up the measurement process, the number of repeats can be reduced by clicking “Setup” → “Number of scans to average” and specifying a smaller number.

8. Switch to a calibrated intensity y-axis. Click “View” → “Radiometer” → “MicroMoles per square meter per second” and click “Yes”. You may want to adjust the limits of the y-axis so that the spectrum is visible. To do this, click “View” → “Y scale” → “set Max Y” and specify a lower value. A typical value is 0.0001.

9. Blank the spectrometer. Remove power from the LED by disconnecting the power adapter at the barrel-jack connection. Allow the spectral measurement to update until the spectrum has

cleared the screen. Then click the dark light bulb icon (fifth icon from the left near the top of the screen). Reconnect power to the LED and allow the spectrum to stabilize.

10. Export the spectrum. Click the floppy disk icon (second icon from the left near the top of the screen). Choose a location and save the spectrum. If the “File save EXPORT parameters” dialog pops up, click “auto-set” on each of the fields to maximize the exported range and resolution of the spectrum.

11. To measure the next LED, remove the current LED and return to either step 5 or 6. Use step 6 if the next LED has an intensity known to be similar to the just-measured LED, or use step 5 to adjust the integration time if necessary. The output of the LED measurement protocol is a “.IRR” spectrum file which contains the complete LED power spectrum in $\Delta\lambda = 0.5$nm increments as well as all setup parameters for the measurement (i.e. integration time, number of averages, etc.). This spectra file can be viewed in a text editor or can be loaded into a spreadsheet viewer. The LED power spectrum can be used to calculate a number of characteristics, including the peak, centroid, FWHM, and total flux.
