# Supplementary material for: A photoconversion model for full spectral programming and multiplexing of optogenetic systems
Source: Mol Syst Biol. 2017 Apr 24;13(4):926. doi: 10.15252/msb.20167456 (PMC5408778; doi:10.15252/msb.20167456)
Supplement: Supplementary file 2 — Expanded View Figures PDF [file MSB-13-926-s002.pdf]

Expanded View Figures

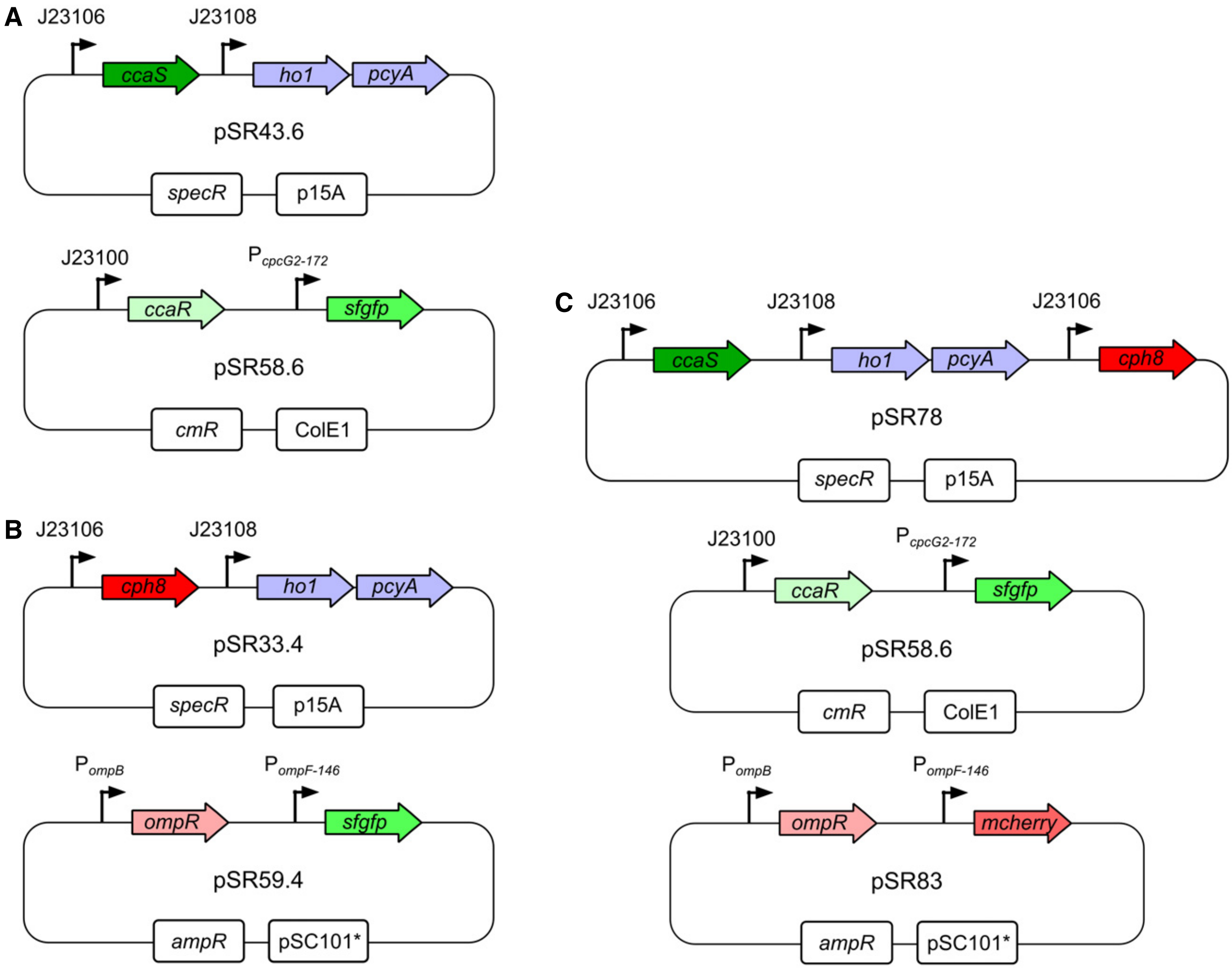

Figure EV1. CcaSR, Cph8-OmpR, and dual-system plasmid maps.

A Maps of plasmids pSR43.6 (top) and pSR58.6 (bottom) expressing the CcaSR system including the PCB biosynthetic enzymes ho1 and pcyA[2].

B Maps of plasmids pSR33.4 (top) and pSR59.4 (bottom) expressing the Cph8-OmpR system.

C Maps of plasmids pSR78 (top), pSR58.6 (middle), and pSR83 (bottom) used in the dual-system experiments. Plasmid sequence information is available (Dataset EV1).

© 2017 The Authors

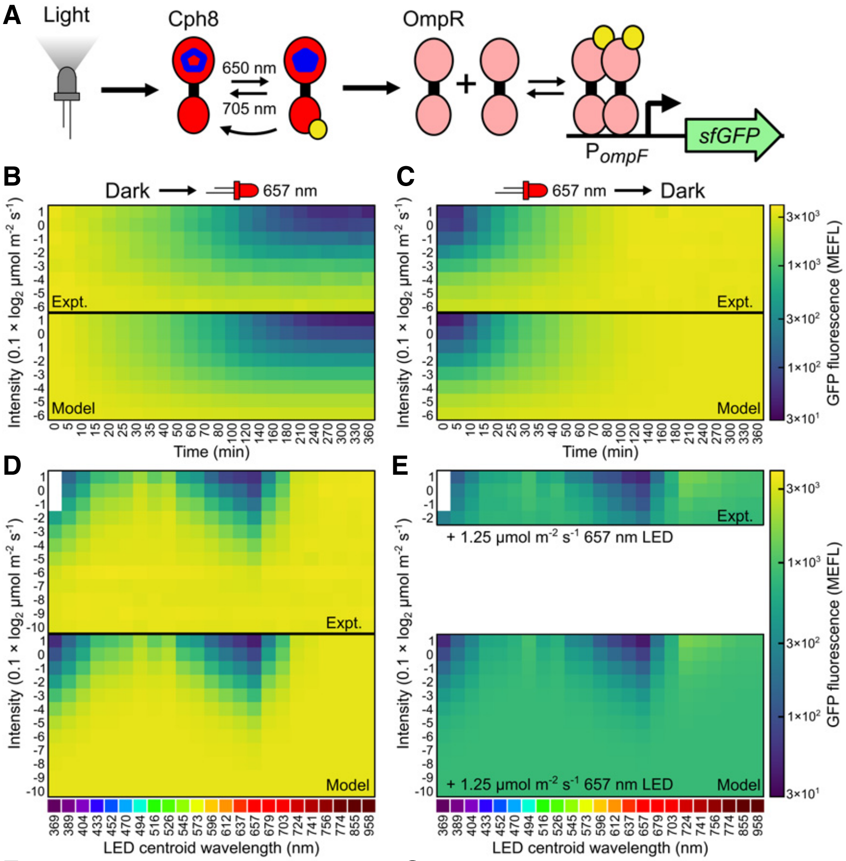

F

Param.	Description	Value	Unit
k_{dil}	Dilution (growth) rate	$2.37(2) \times 10^{-2}$	min^{-1}
k_{dr}	Dark reversion rate	$1.26(6) \times 10^{-1}$	min^{-1}
τ	Signaling delay	2.4(4)	min
b	Output fluor. minimum	$1.6(3) \times 10^{1}$	MEFL
a	Output fluor. range	$3.34(1) \times 10^{3}$	MEFL
K	S_{on}/S_{off} ratio at 50% output	0.197	
n	Signaling Hill coefficient	1.30(1)	

G

λ_c	$\hat{k}_1$	$\hat{k}_2$	λ_c	$\hat{k}_1$	$\hat{k}_2$
369	50(4)	1(5)	596	15.5(8)	0
389	30(2)	6(1)	612	25(1)	0
404	14(1)	6(1)	637	37(2)	0
433	5.6(5)	5(1)	657	57(2)	0
452	4.5(4)	5(1)	679	13.6(9)	2.2(7)
470	3.6(3)	5(1)	703	7.9(7)	12(2)
494	1.8(2)	5.3(9)	724	1.2(3)	13(2)
516	2.6(2)	1.2(6)	741	0.6(2)	8(1)
526	1.8(2)	1.8(7)	756	0	4(6)
545	5.9(4)	0.4(6)	774	0	2(5)
573	8.1(6)	1.4(7)	855	0	1(5)
			958	0	1(5)

Figure EV2. Cph8-OmpR characterization and model parameterization.

A–G Figure details are described in Fig 2, and data are available in Dataset EV7. Note that the reverse action spectrum measurements (E) contain only four intensities of the spectral LEDs rather than the full set of 12 used in the CcaSR experiments.

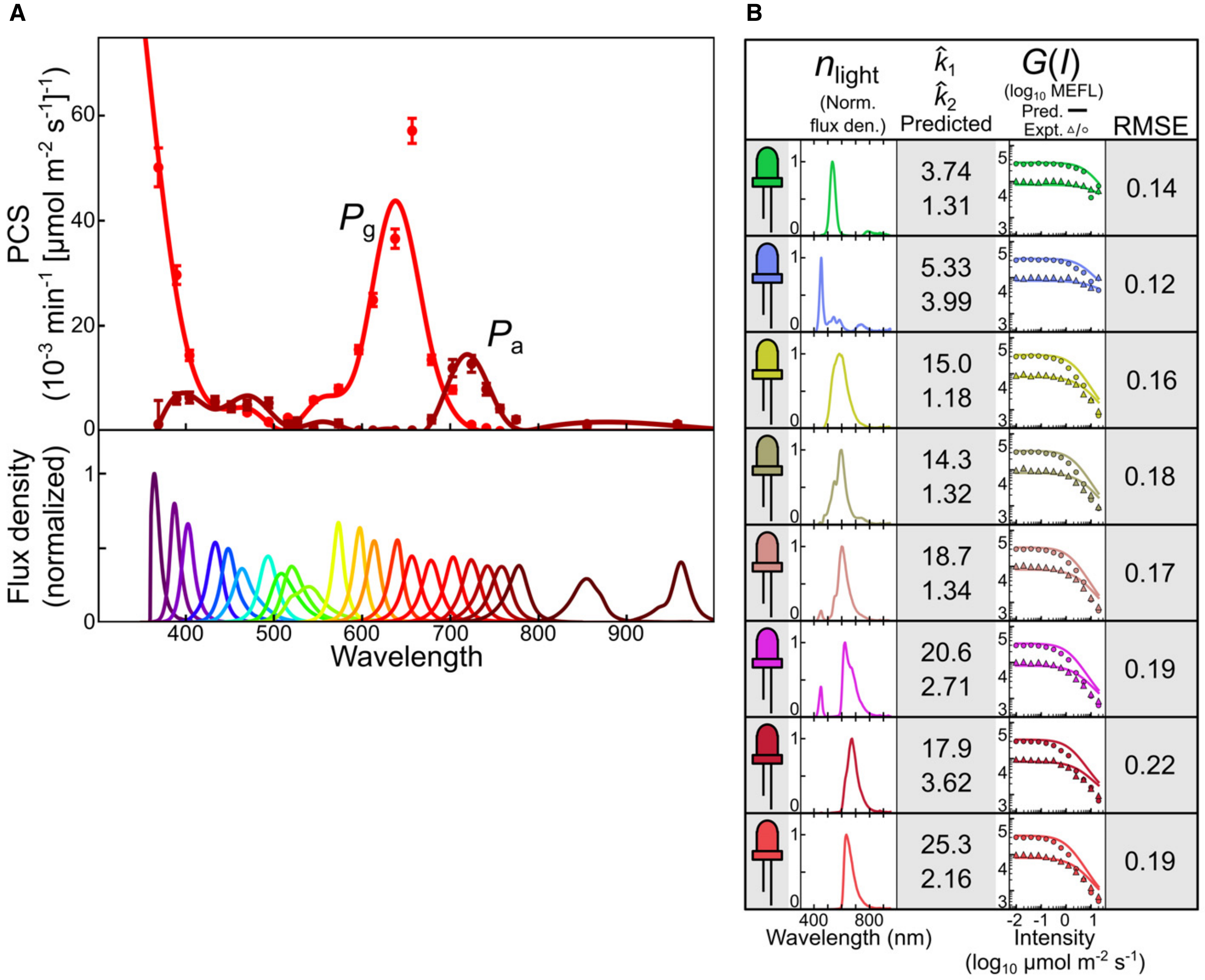

Figure EV3. Cph8 PCS estimation and Cph8-OmpR model spectral validation.

A, B Figure details are described in Fig 3, and data are available in Dataset EV7.

 © 2017 The Authors

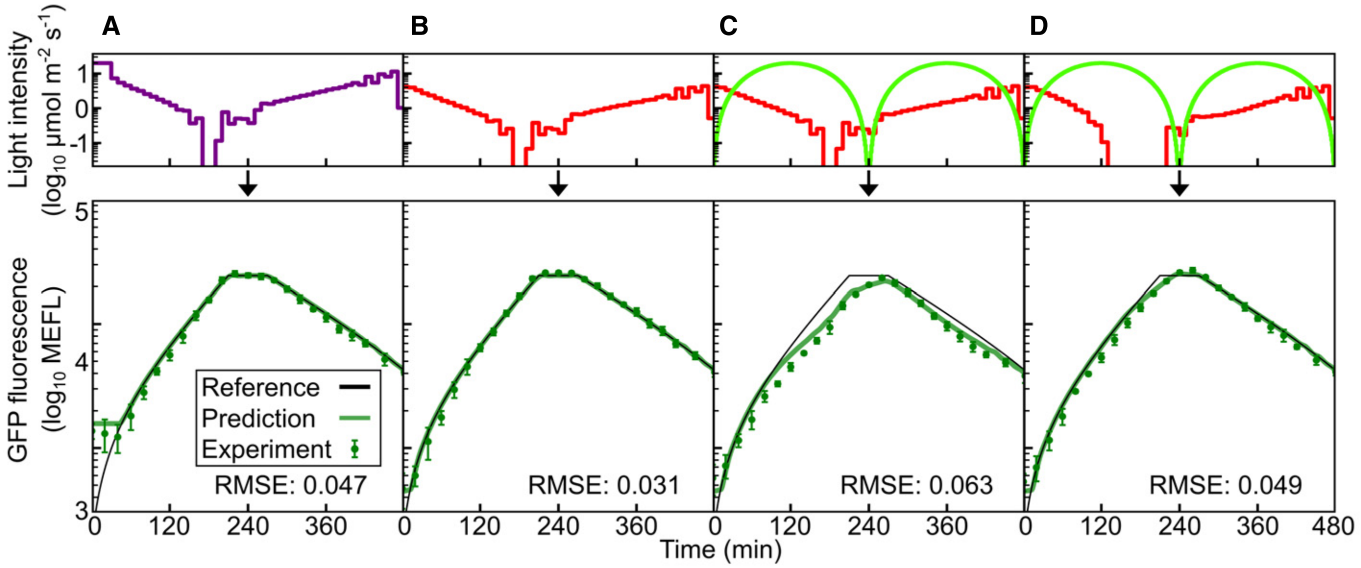

Figure EV4. Cph8-OmpR model dynamic programming validation.

A–D Figure details are described in Fig 4, and data are available in Dataset EV7.

© 2017 The Authors
