# Supplementary figures and images for: A photoconversion model for full spectral programming and multiplexing of optogenetic systems (part 1 of 3)
Source: Mol Syst Biol. 2017 Apr 24;13(4):926. doi: 10.15252/msb.20167456 (PMC5408778; doi:10.15252/msb.20167456)

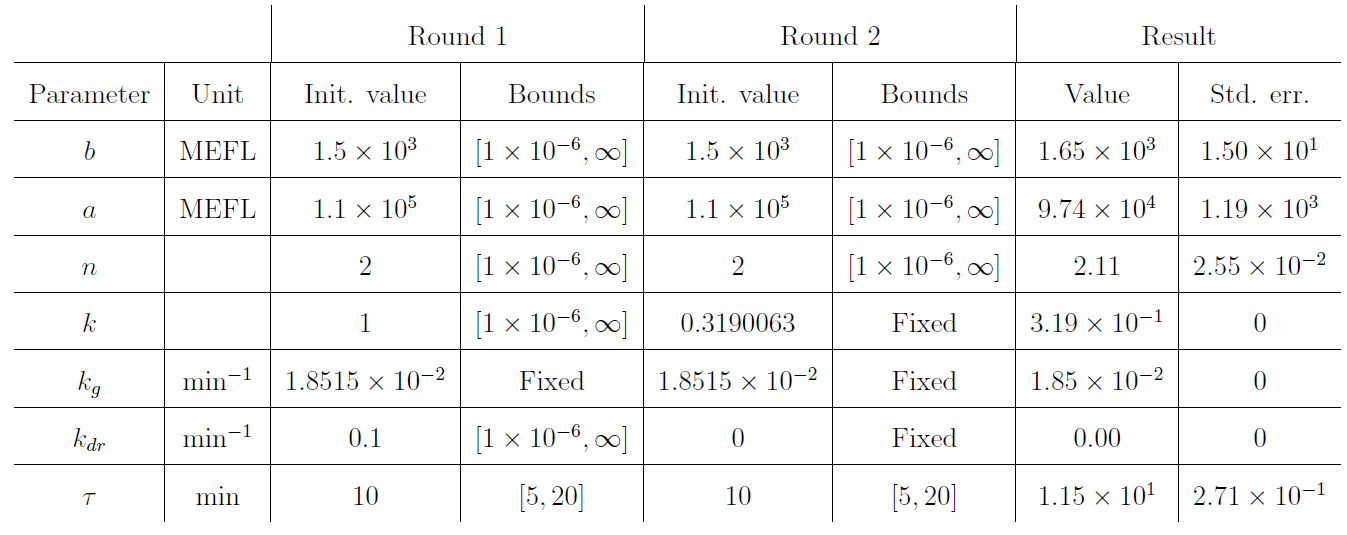

Supplement: Supplementary file 6 — Table EV4 [file MSB-13-926-s006.zip › table_ev4/rgv2_params_1.PNG]

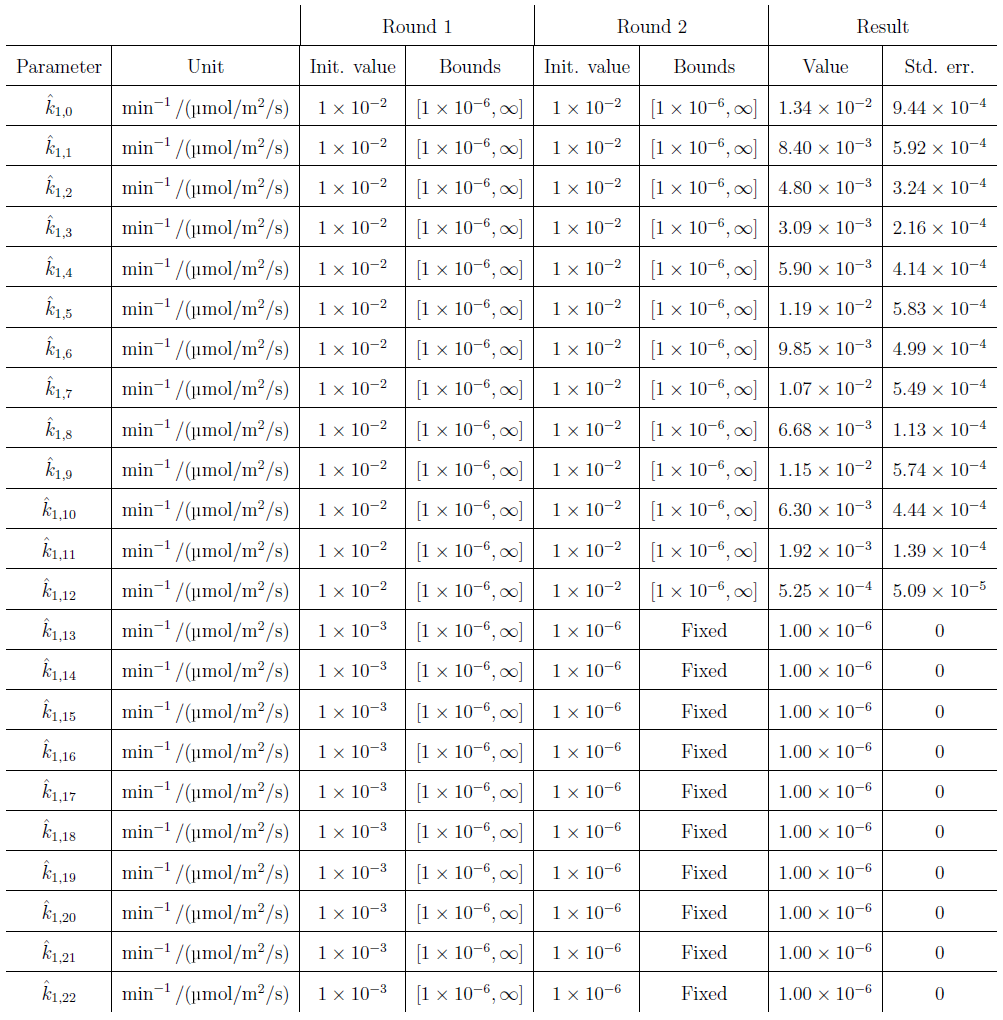

Supplement: Supplementary file 6 — Table EV4 [file MSB-13-926-s006.zip › table_ev4/rgv2_params_2.PNG]

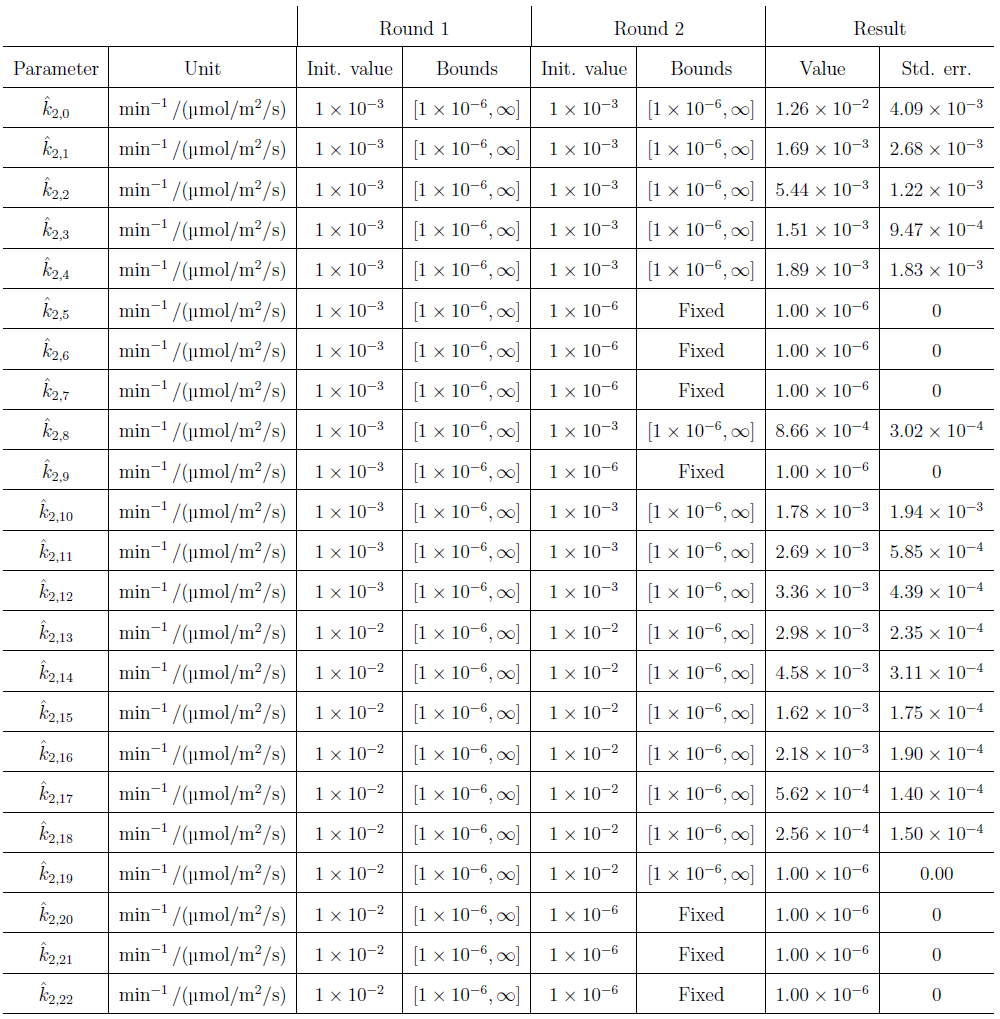

Supplement: Supplementary file 6 — Table EV4 [file MSB-13-926-s006.zip › table_ev4/rgv2_params_3.PNG]

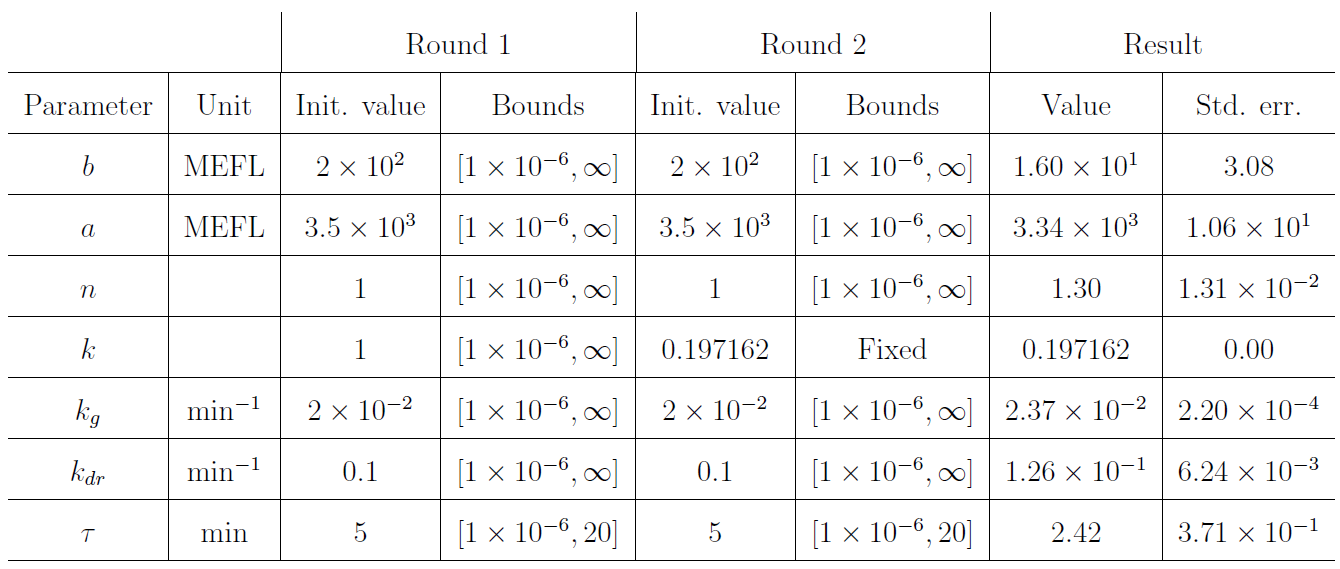

Supplement: Supplementary file 7 — Table EV5 [file MSB-13-926-s007.zip › table_ev5/rdv2_params_1.PNG]

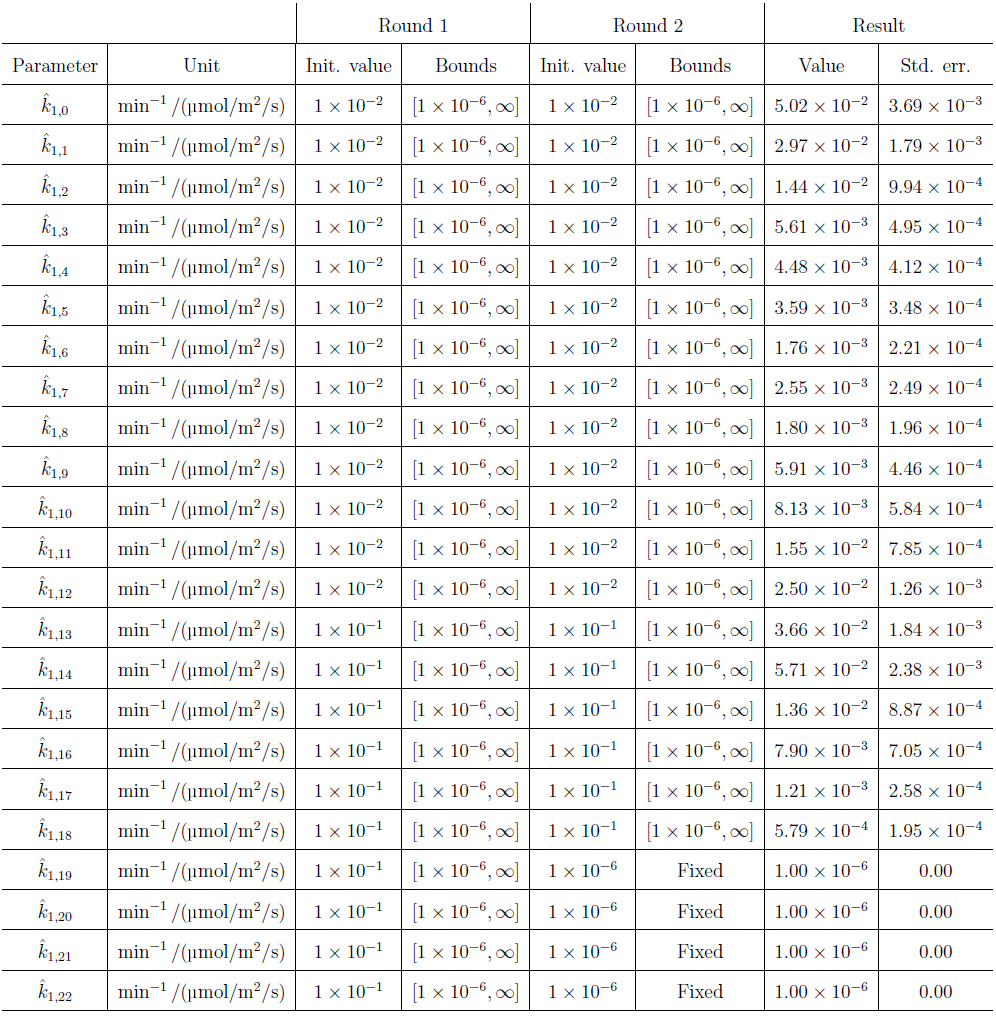

Supplement: Supplementary file 7 — Table EV5 [file MSB-13-926-s007.zip › table_ev5/rdv2_params_2.PNG]

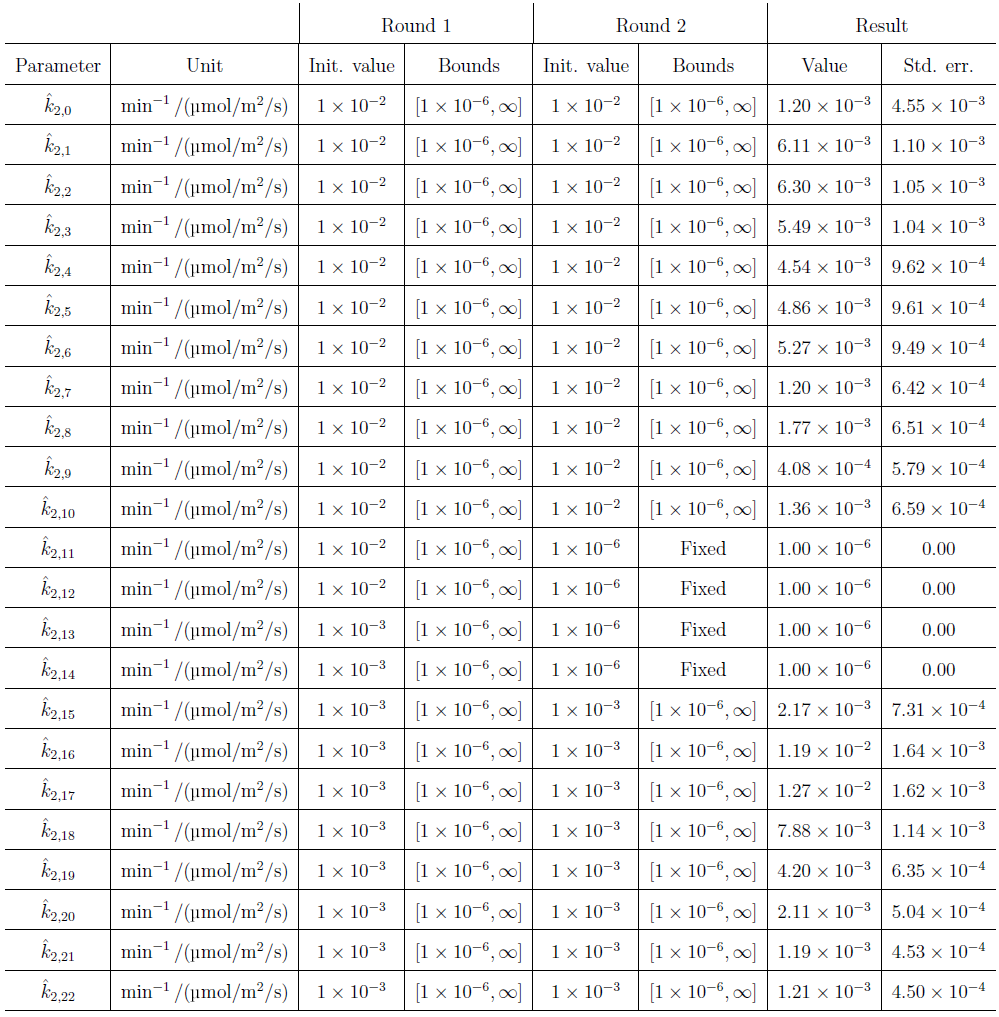

Supplement: Supplementary file 7 — Table EV5 [file MSB-13-926-s007.zip › table_ev5/rdv2_params_3.PNG]

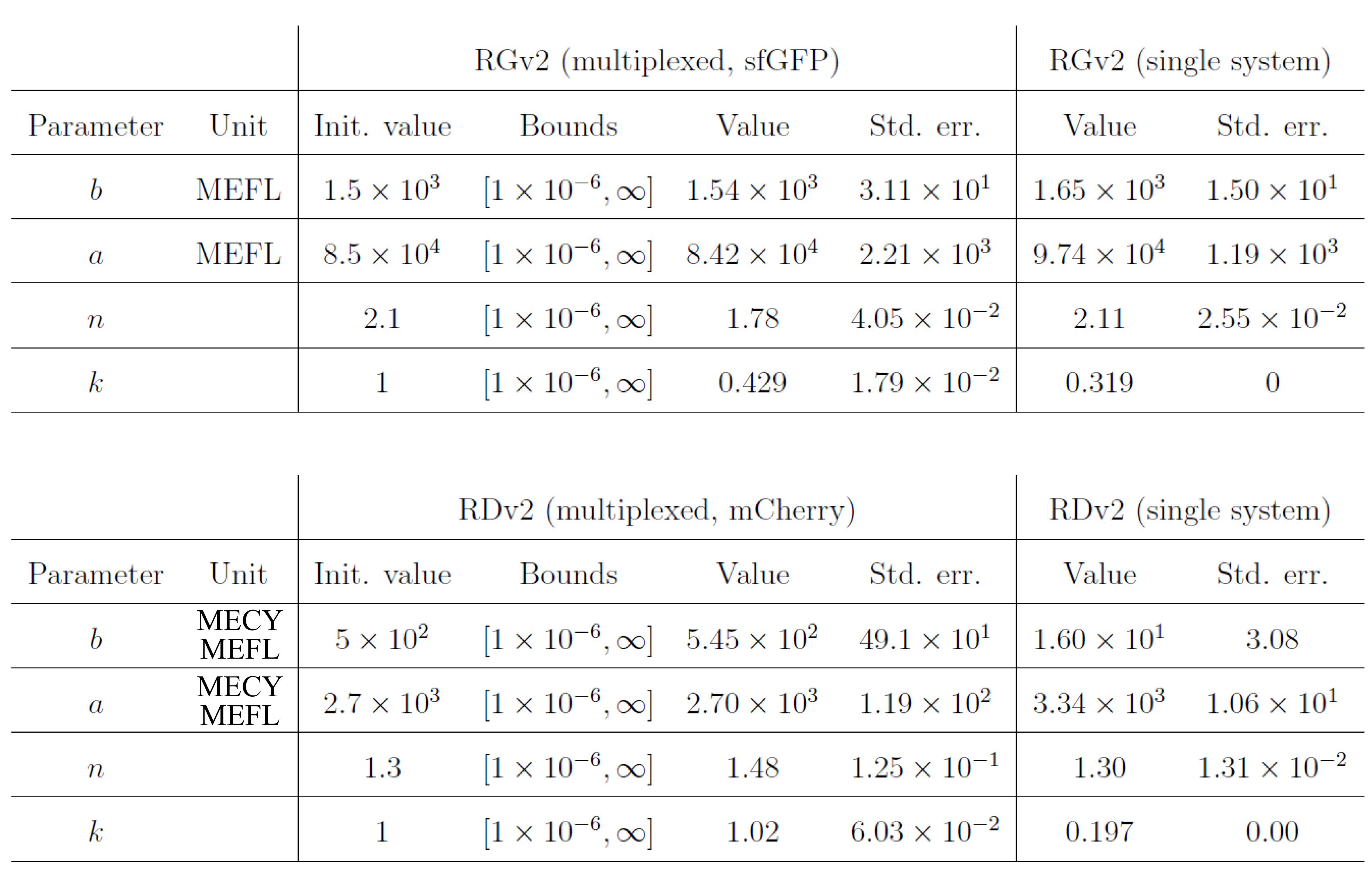

Supplement: Supplementary file 8 — Table EV6 [file MSB-13-926-s008.zip › table_ev6/mux_params.png]

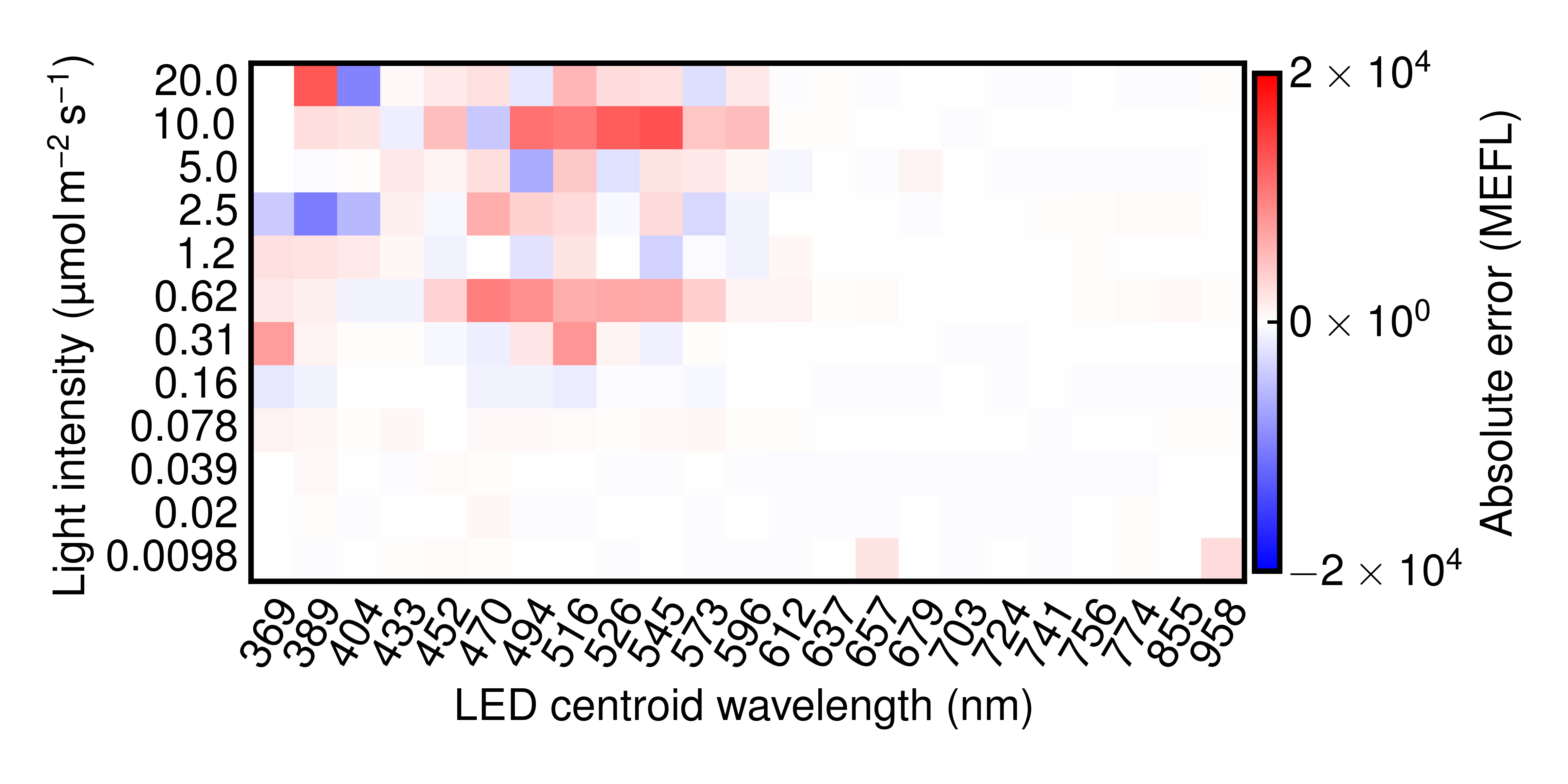

Supplement: Supplementary file 10 — Dataset EV2 [file MSB-13-926-s010.zip › dataset_ev2_ccasr_data_and_analysis/ccasr_analysis/plots/aas_abs_residual_heatmap.png]

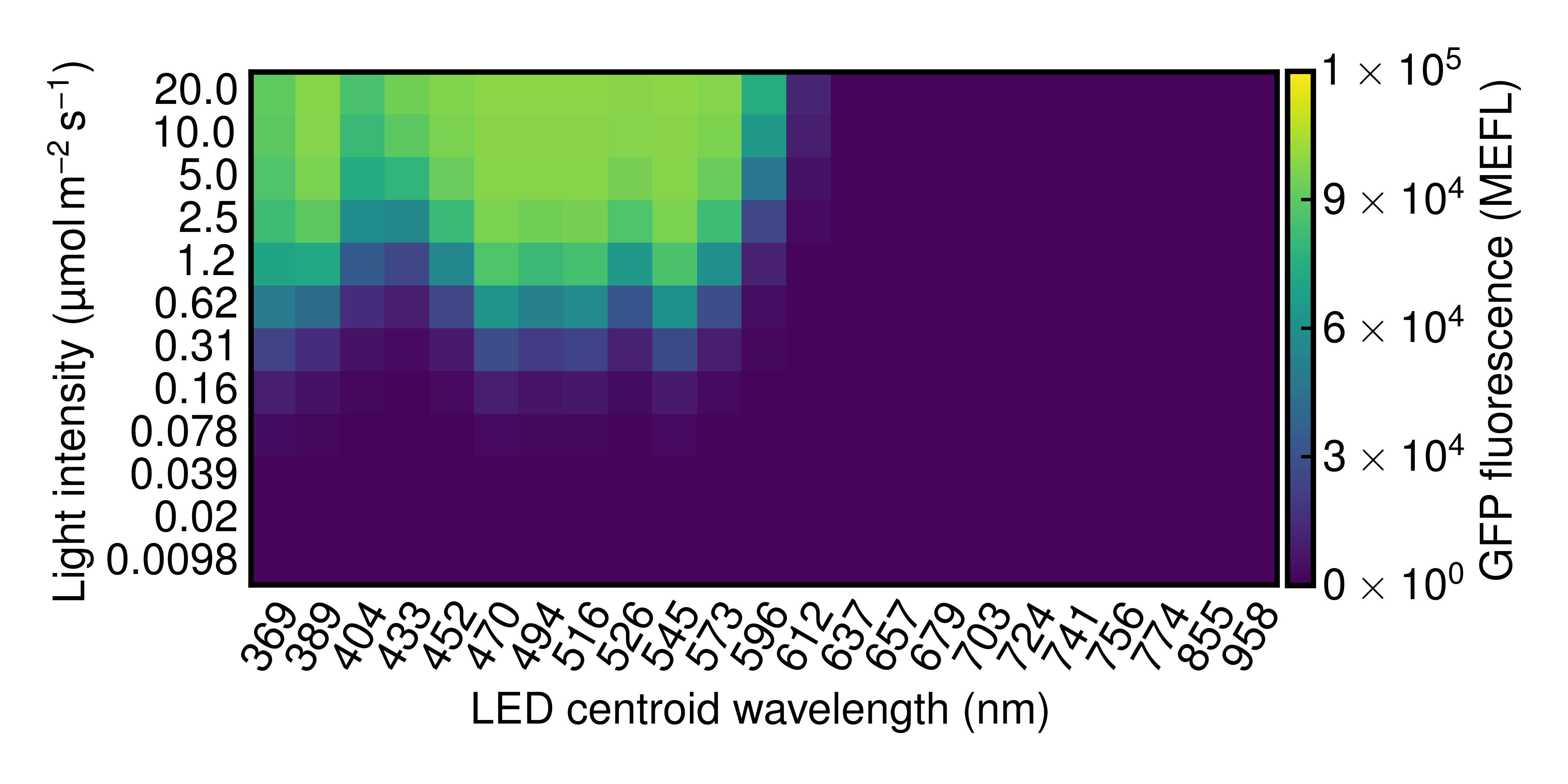

Supplement: Supplementary file 10 — Dataset EV2 [file MSB-13-926-s010.zip › dataset_ev2_ccasr_data_and_analysis/ccasr_analysis/plots/aas_lin_model_heatmap.png]

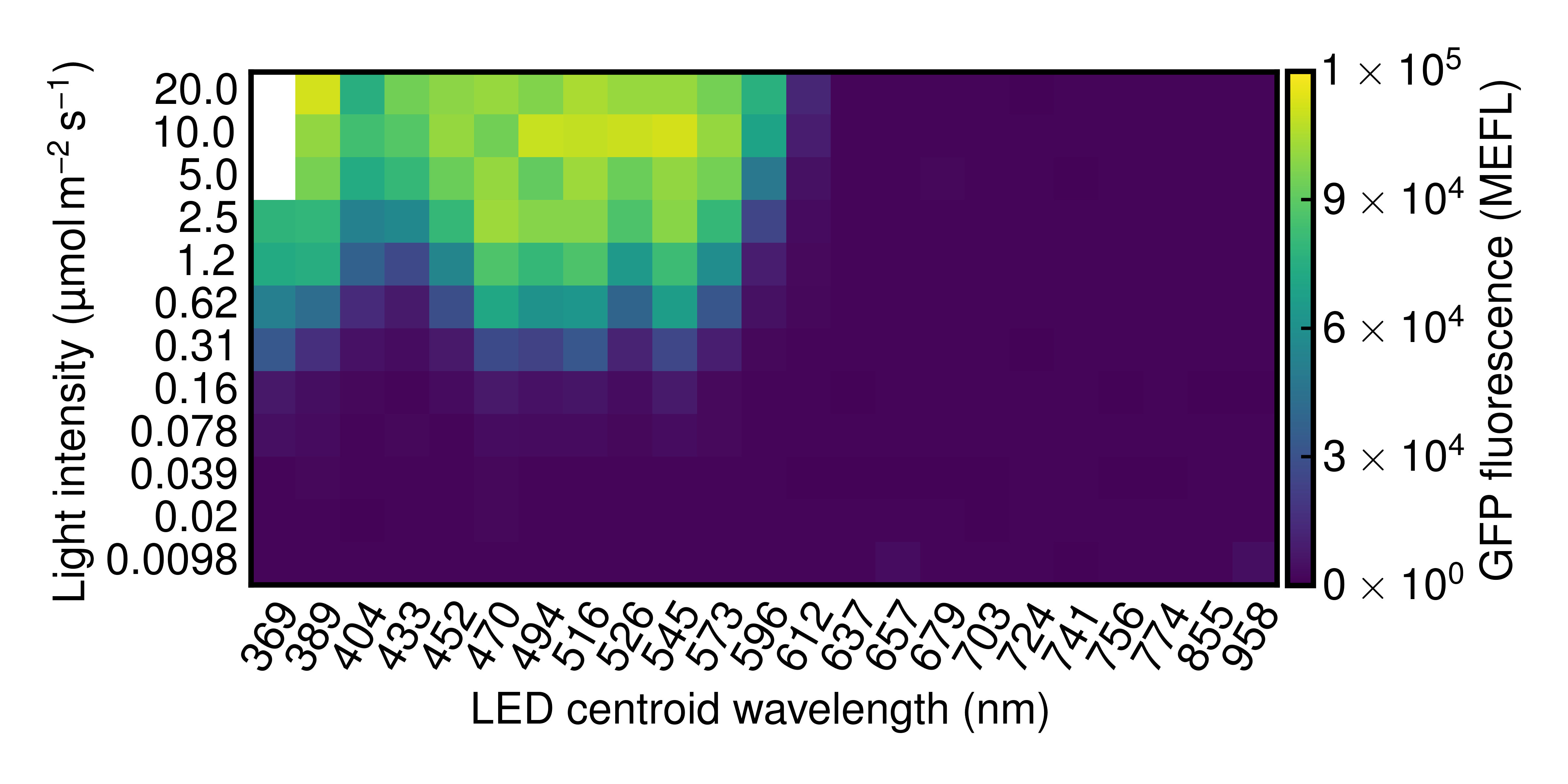

Supplement: Supplementary file 10 — Dataset EV2 [file MSB-13-926-s010.zip › dataset_ev2_ccasr_data_and_analysis/ccasr_analysis/plots/aas_lin_raw_heatmap.png]

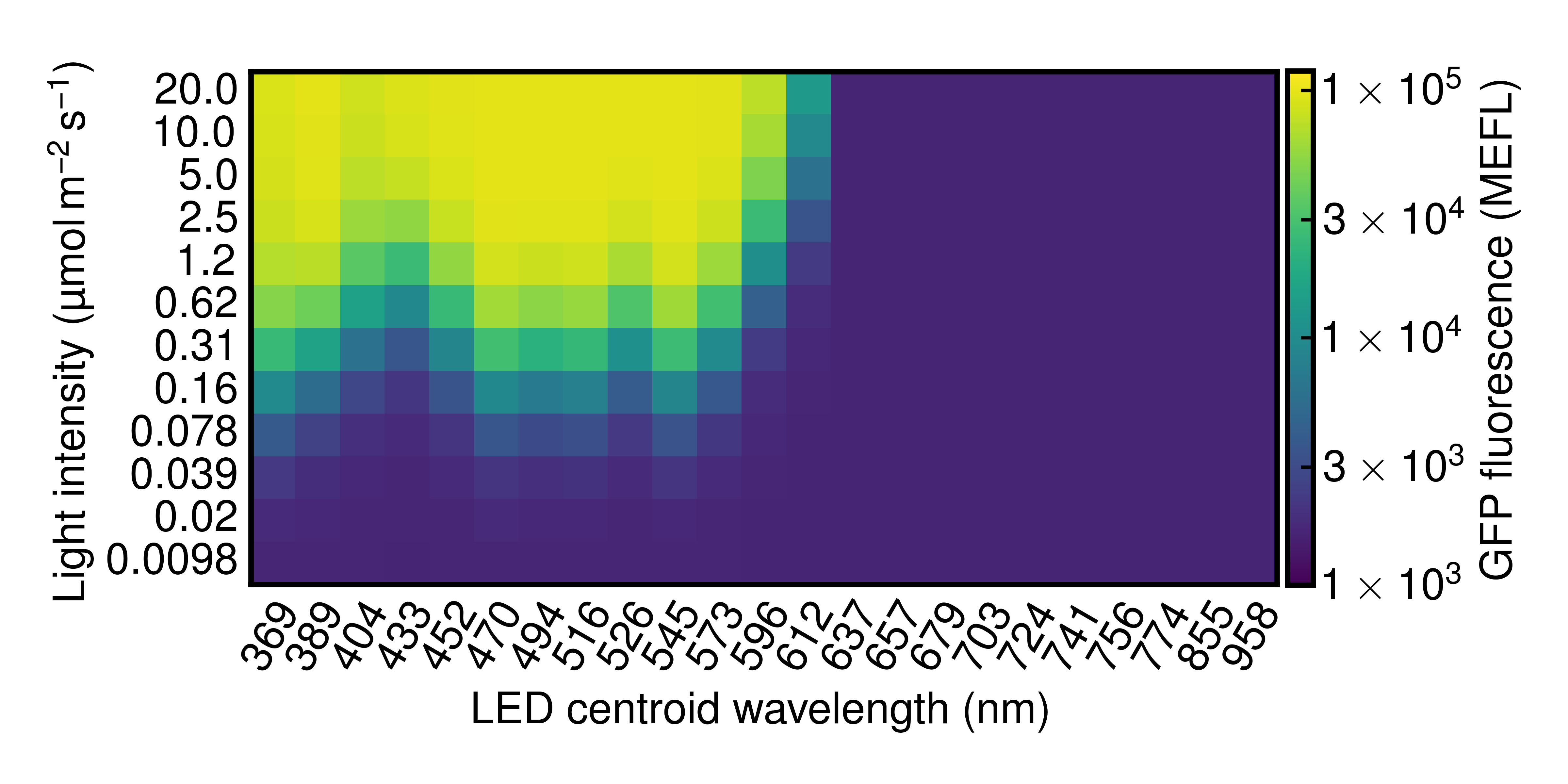

Supplement: Supplementary file 10 — Dataset EV2 [file MSB-13-926-s010.zip › dataset_ev2_ccasr_data_and_analysis/ccasr_analysis/plots/aas_logz_model_heatmap.png]

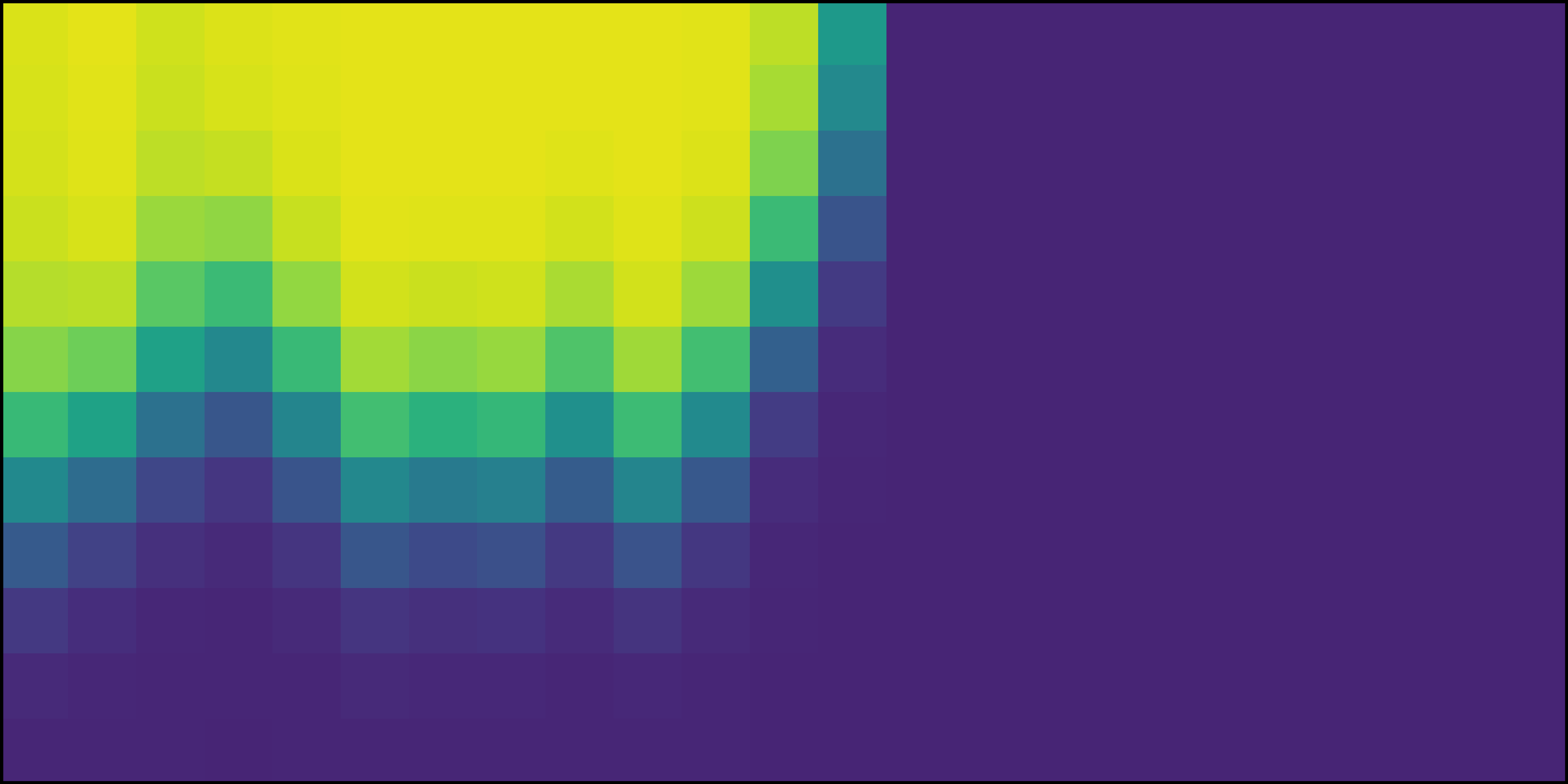

Supplement: Supplementary file 10 — Dataset EV2 [file MSB-13-926-s010.zip › dataset_ev2_ccasr_data_and_analysis/ccasr_analysis/plots/aas_logz_model_nolabel_heatmap.png]

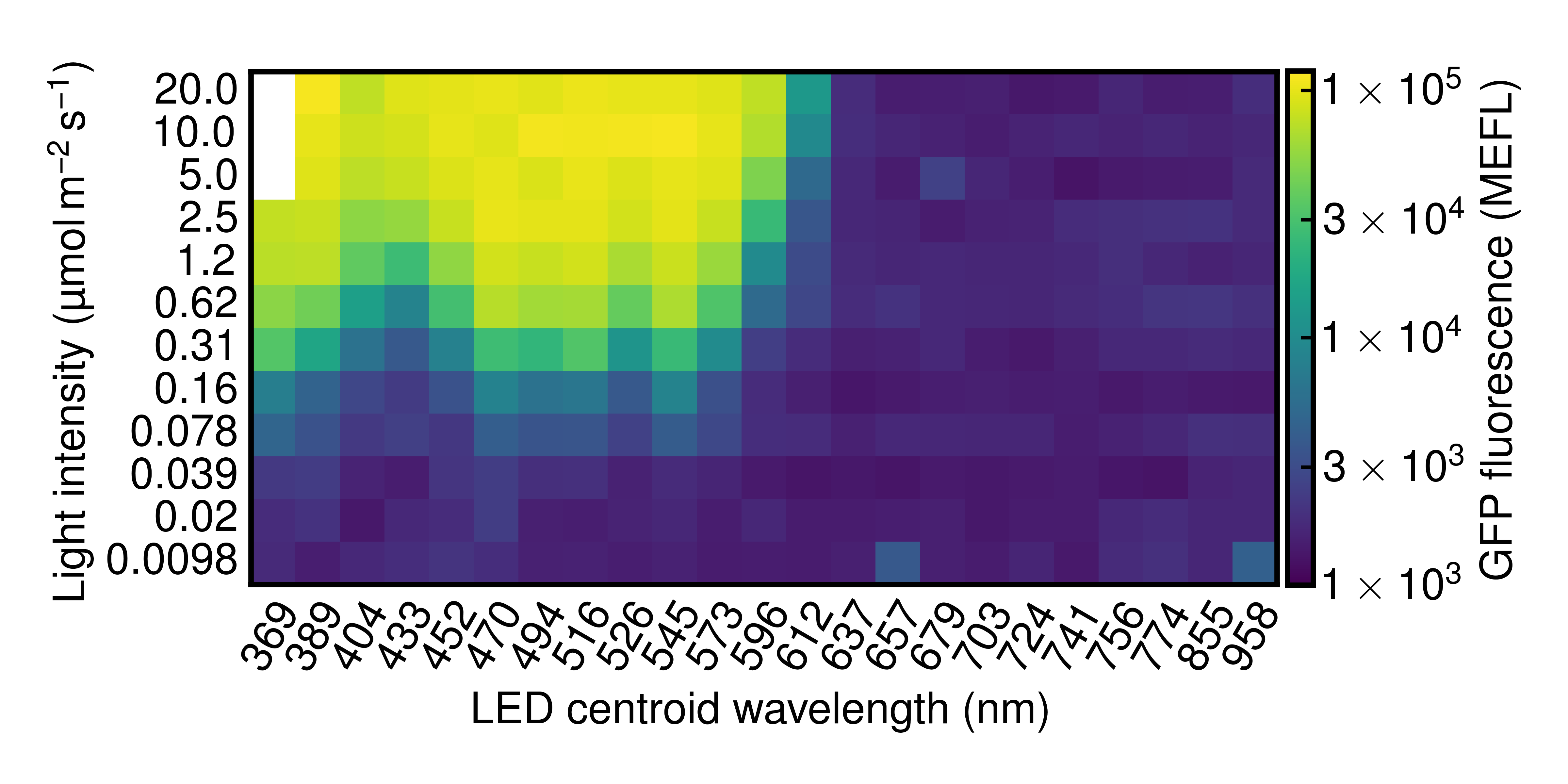

Supplement: Supplementary file 10 — Dataset EV2 [file MSB-13-926-s010.zip › dataset_ev2_ccasr_data_and_analysis/ccasr_analysis/plots/aas_logz_raw_heatmap.png]

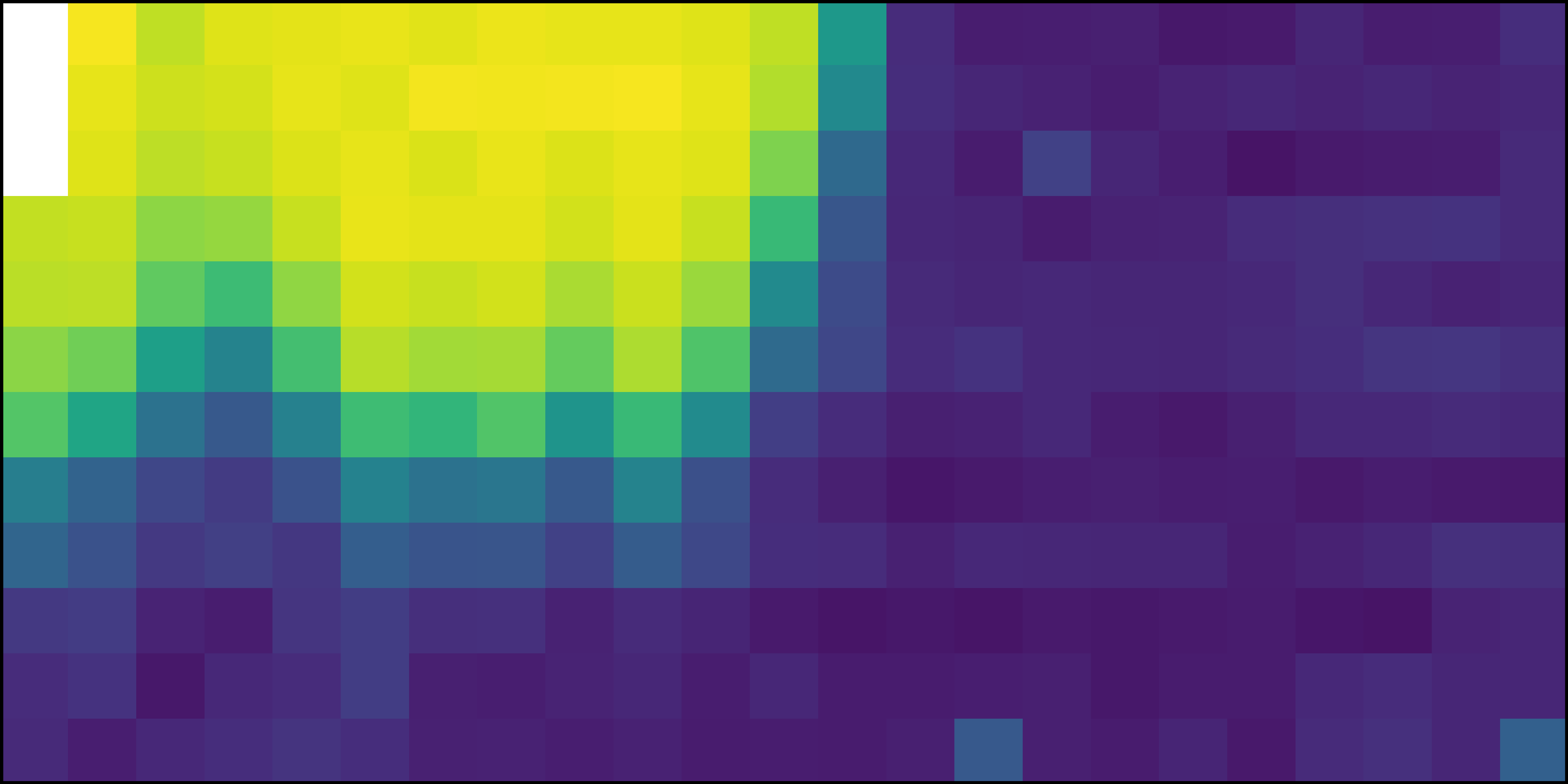

Supplement: Supplementary file 10 — Dataset EV2 [file MSB-13-926-s010.zip › dataset_ev2_ccasr_data_and_analysis/ccasr_analysis/plots/aas_logz_raw_nolabel_heatmap.png]

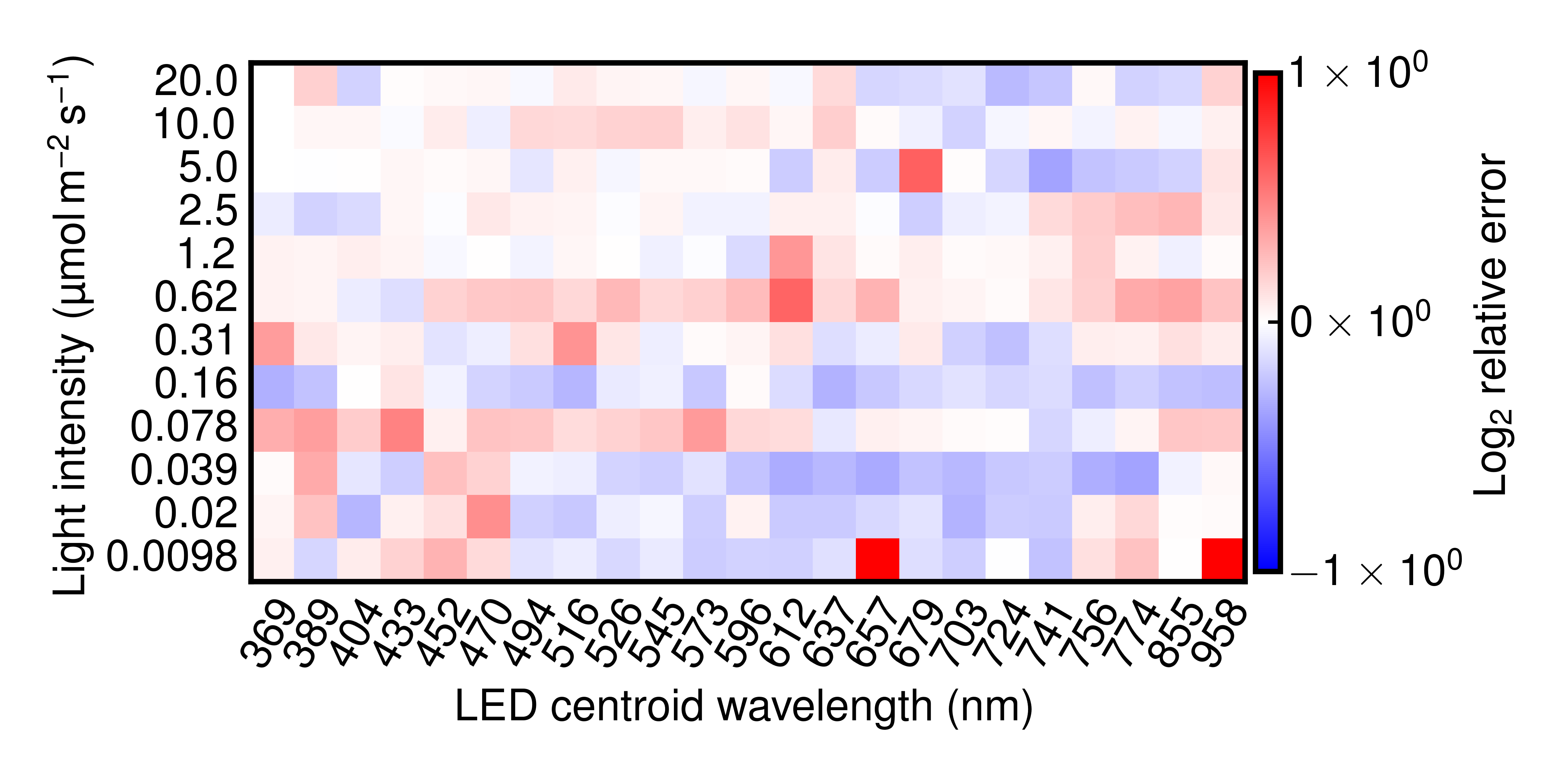

Supplement: Supplementary file 10 — Dataset EV2 [file MSB-13-926-s010.zip › dataset_ev2_ccasr_data_and_analysis/ccasr_analysis/plots/aas_rel_residual_heatmap.png]

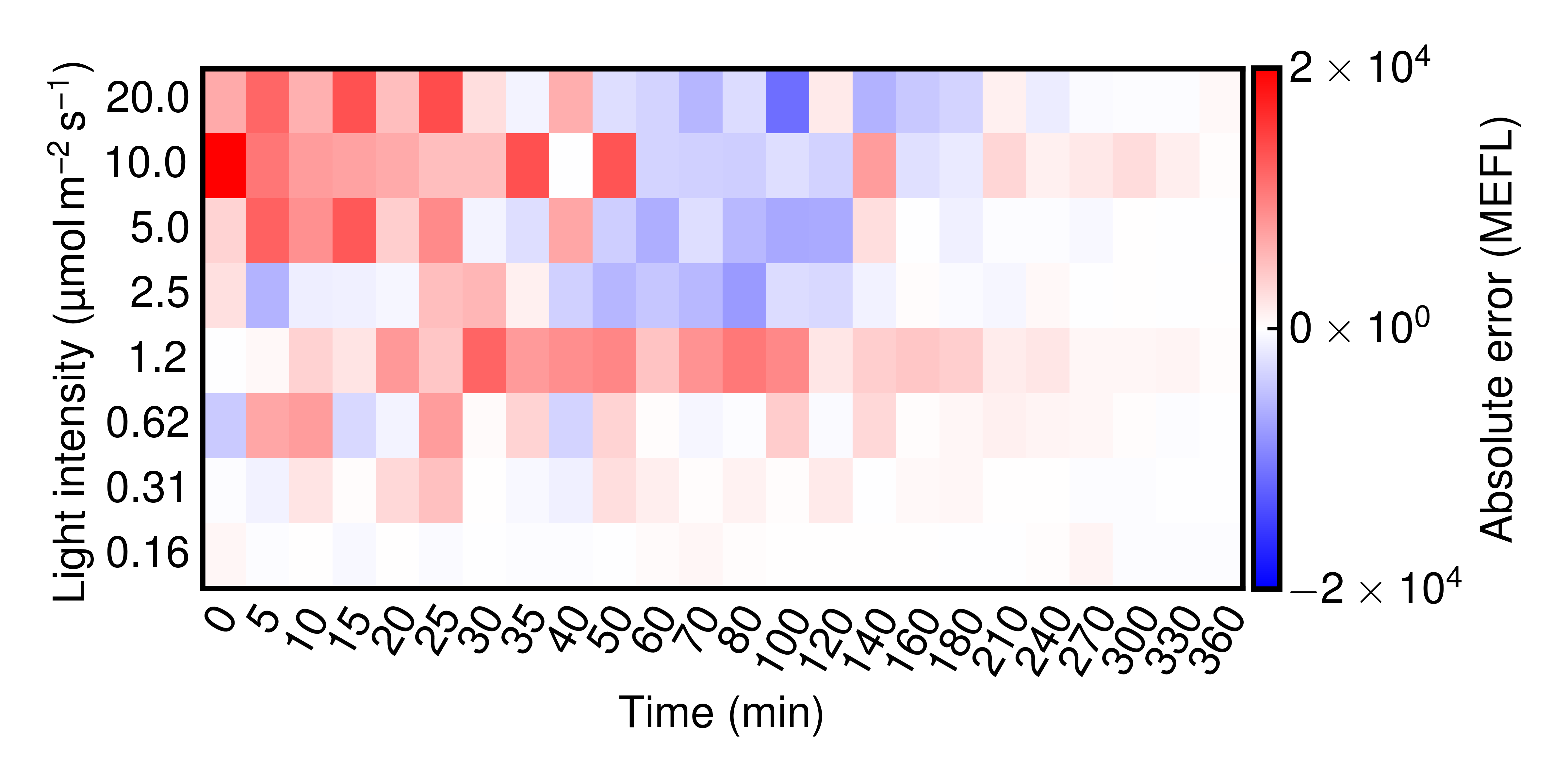

Supplement: Supplementary file 10 — Dataset EV2 [file MSB-13-926-s010.zip › dataset_ev2_ccasr_data_and_analysis/ccasr_analysis/plots/atd_abs_residual_hmap.png]

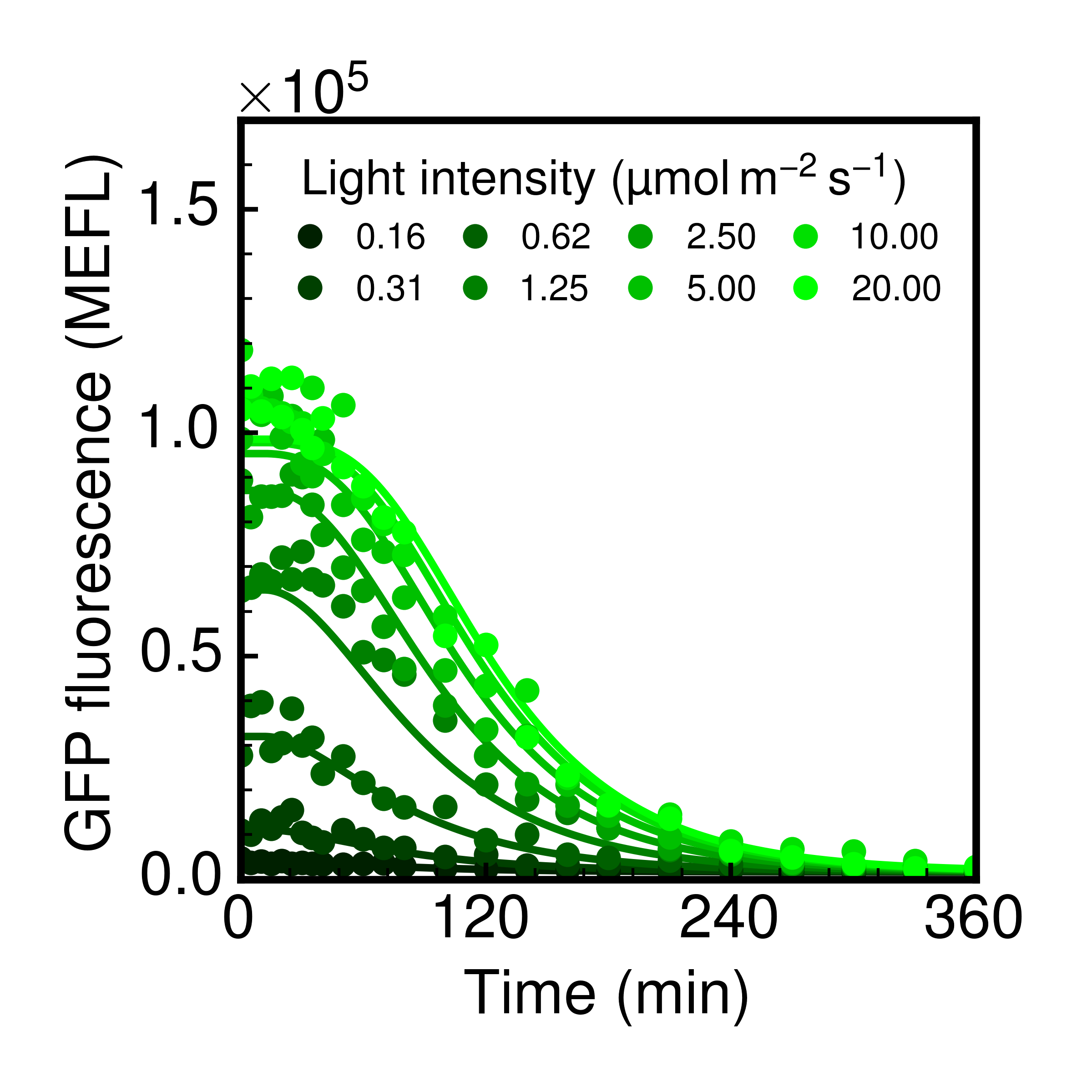

Supplement: Supplementary file 10 — Dataset EV2 [file MSB-13-926-s010.zip › dataset_ev2_ccasr_data_and_analysis/ccasr_analysis/plots/atd_lin_model.png]

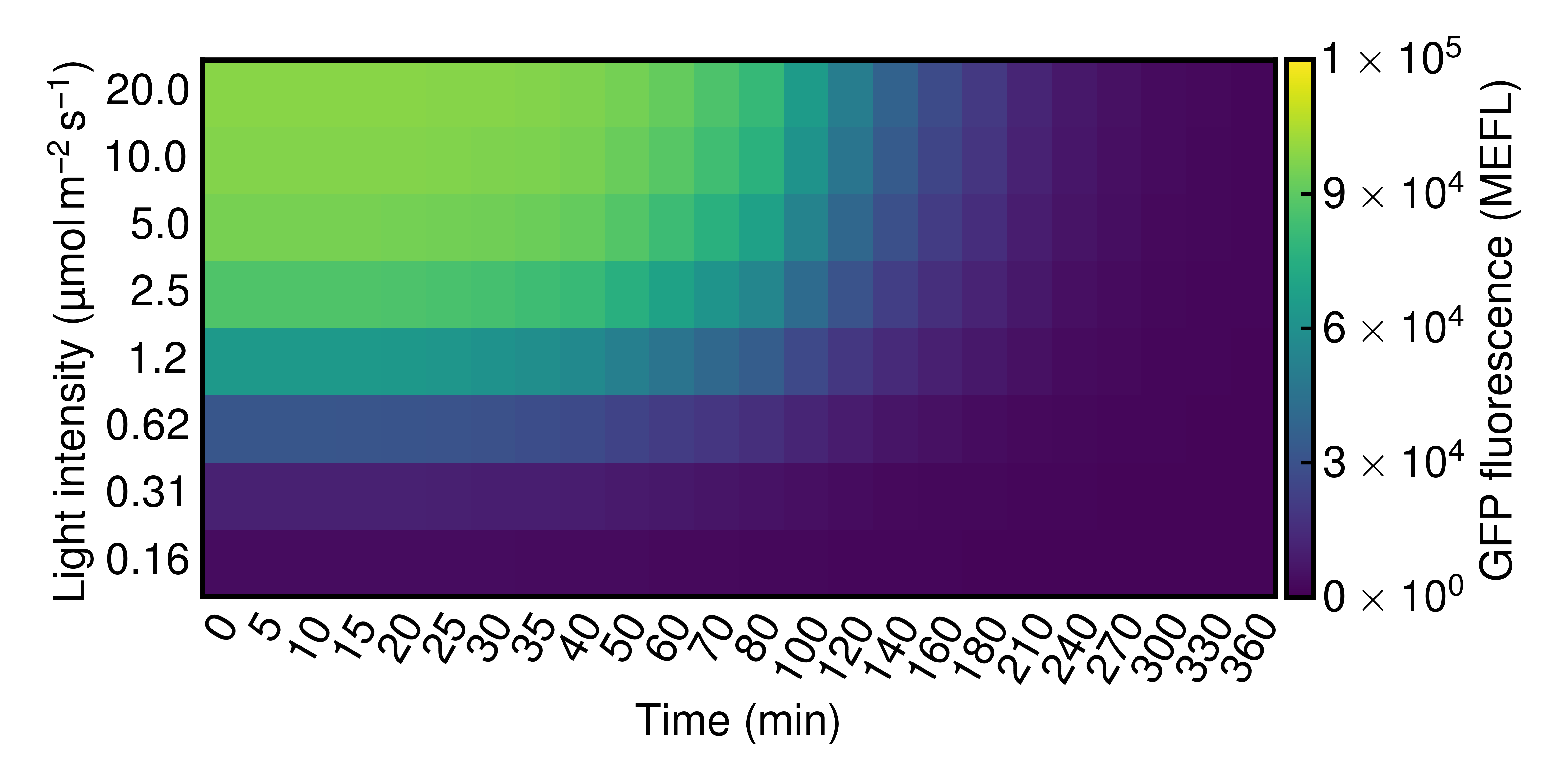

Supplement: Supplementary file 10 — Dataset EV2 [file MSB-13-926-s010.zip › dataset_ev2_ccasr_data_and_analysis/ccasr_analysis/plots/atd_lin_model_hmap.png]

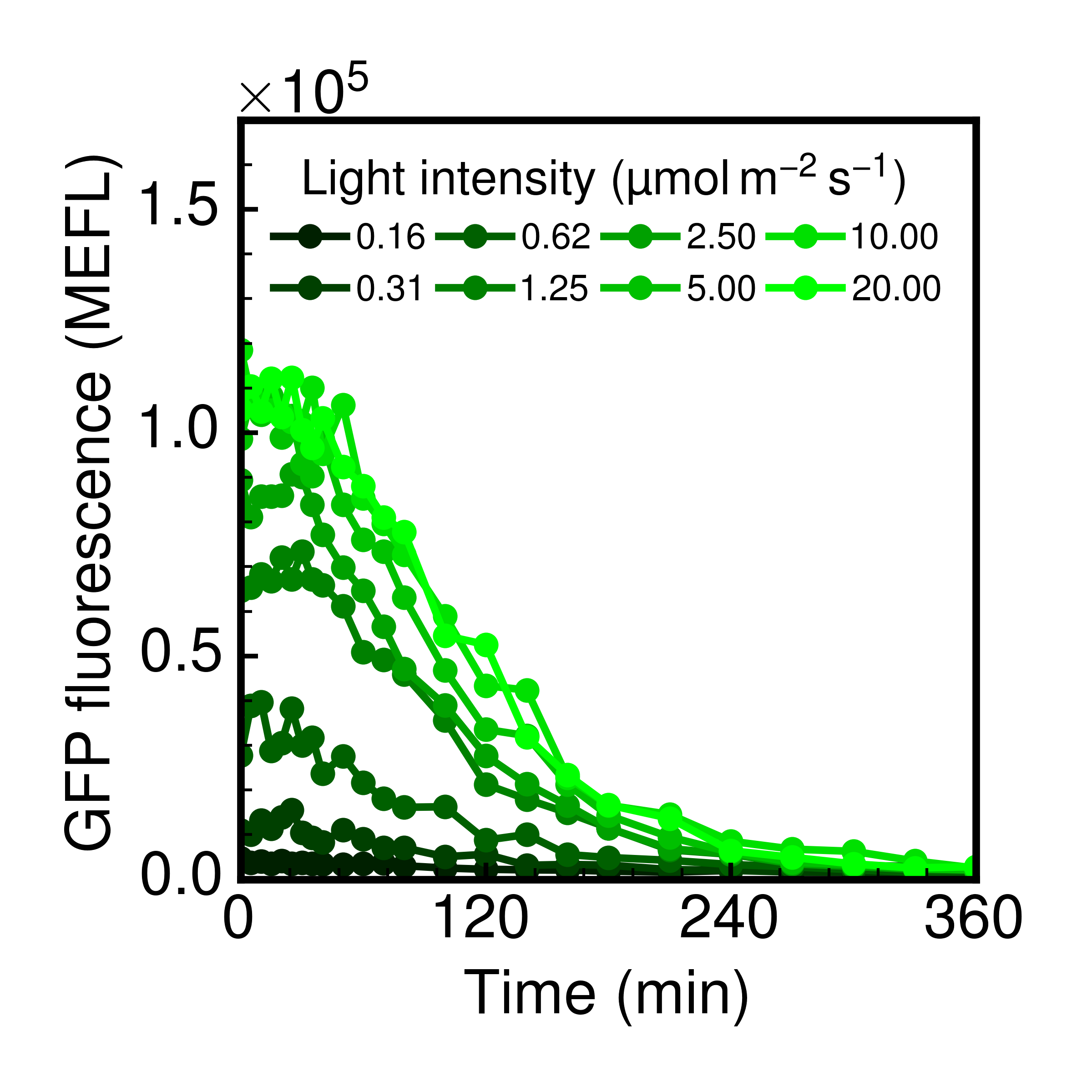

Supplement: Supplementary file 10 — Dataset EV2 [file MSB-13-926-s010.zip › dataset_ev2_ccasr_data_and_analysis/ccasr_analysis/plots/atd_lin_raw.png]

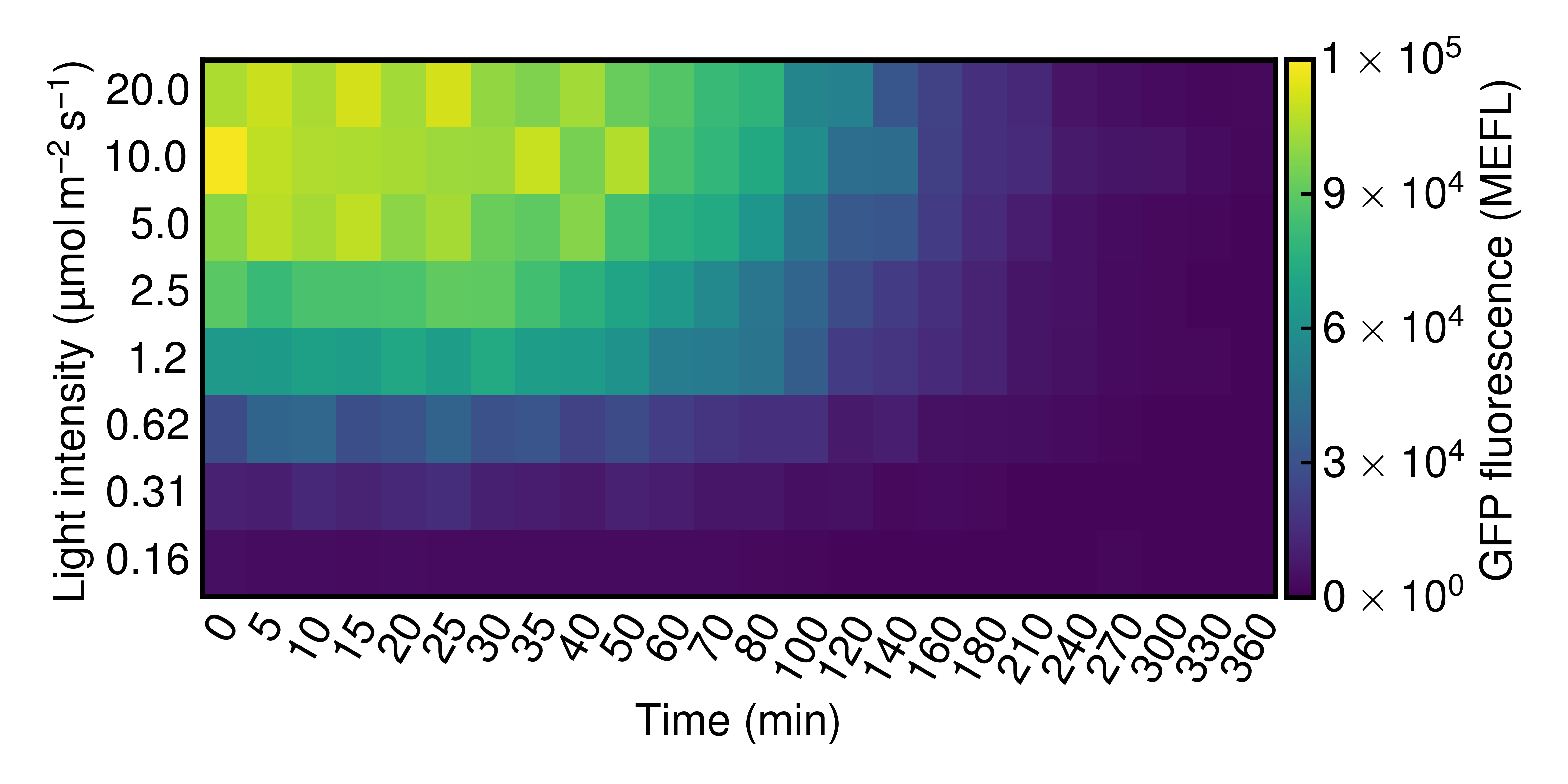

Supplement: Supplementary file 10 — Dataset EV2 [file MSB-13-926-s010.zip › dataset_ev2_ccasr_data_and_analysis/ccasr_analysis/plots/atd_lin_raw_hmap.png]

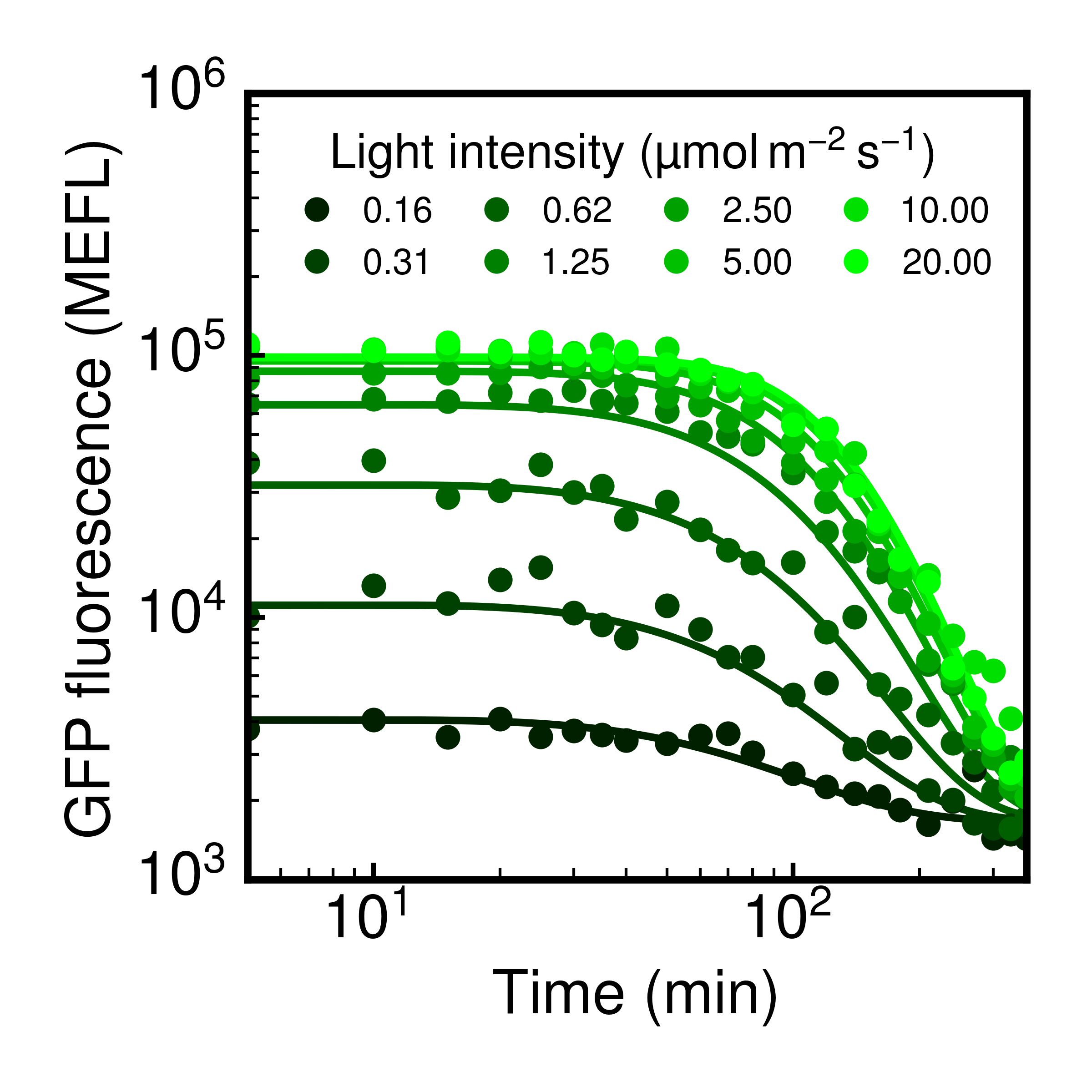

Supplement: Supplementary file 10 — Dataset EV2 [file MSB-13-926-s010.zip › dataset_ev2_ccasr_data_and_analysis/ccasr_analysis/plots/atd_logxy_model.png]

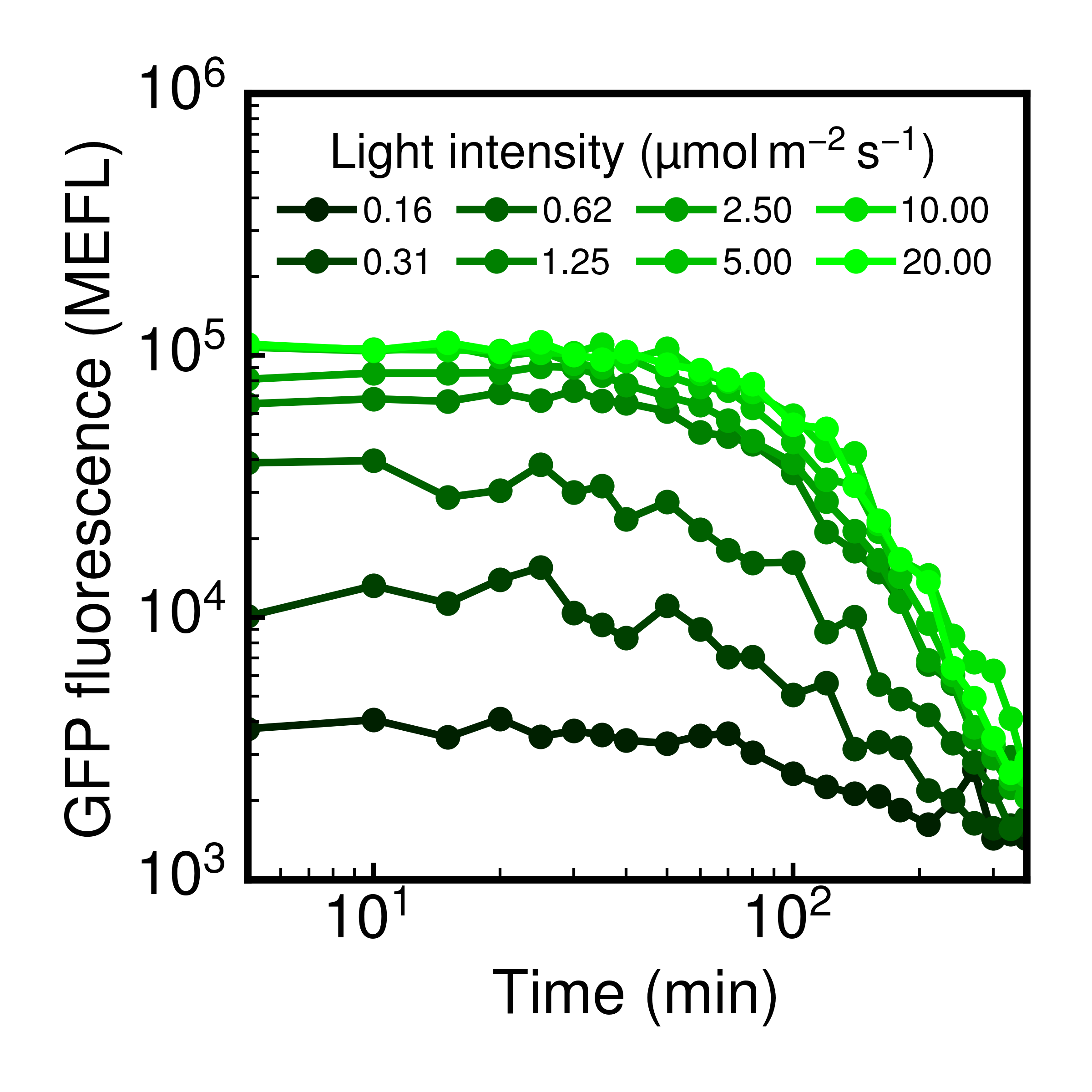

Supplement: Supplementary file 10 — Dataset EV2 [file MSB-13-926-s010.zip › dataset_ev2_ccasr_data_and_analysis/ccasr_analysis/plots/atd_logxy_raw.png]

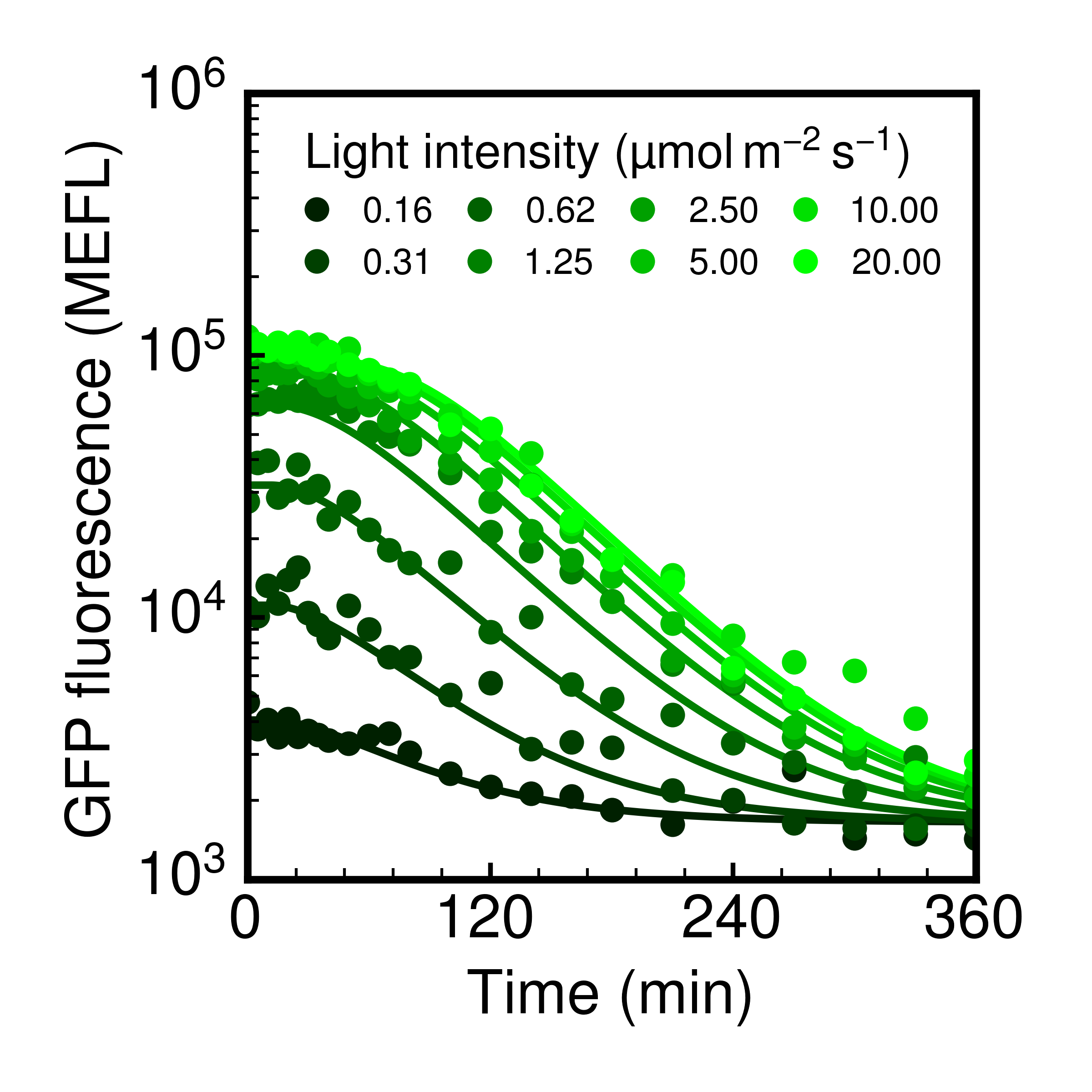

Supplement: Supplementary file 10 — Dataset EV2 [file MSB-13-926-s010.zip › dataset_ev2_ccasr_data_and_analysis/ccasr_analysis/plots/atd_logy_model.png]

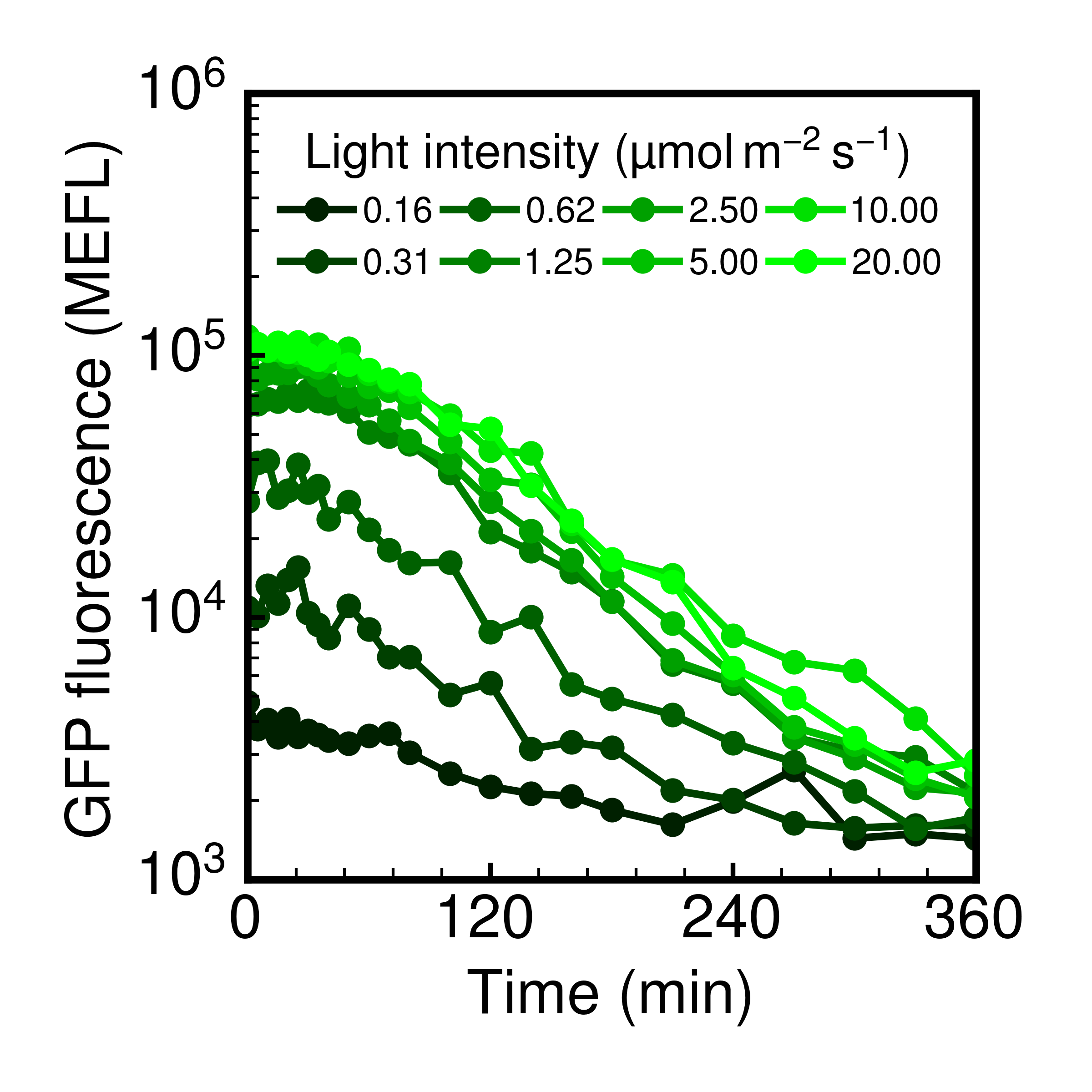

Supplement: Supplementary file 10 — Dataset EV2 [file MSB-13-926-s010.zip › dataset_ev2_ccasr_data_and_analysis/ccasr_analysis/plots/atd_logy_raw.png]

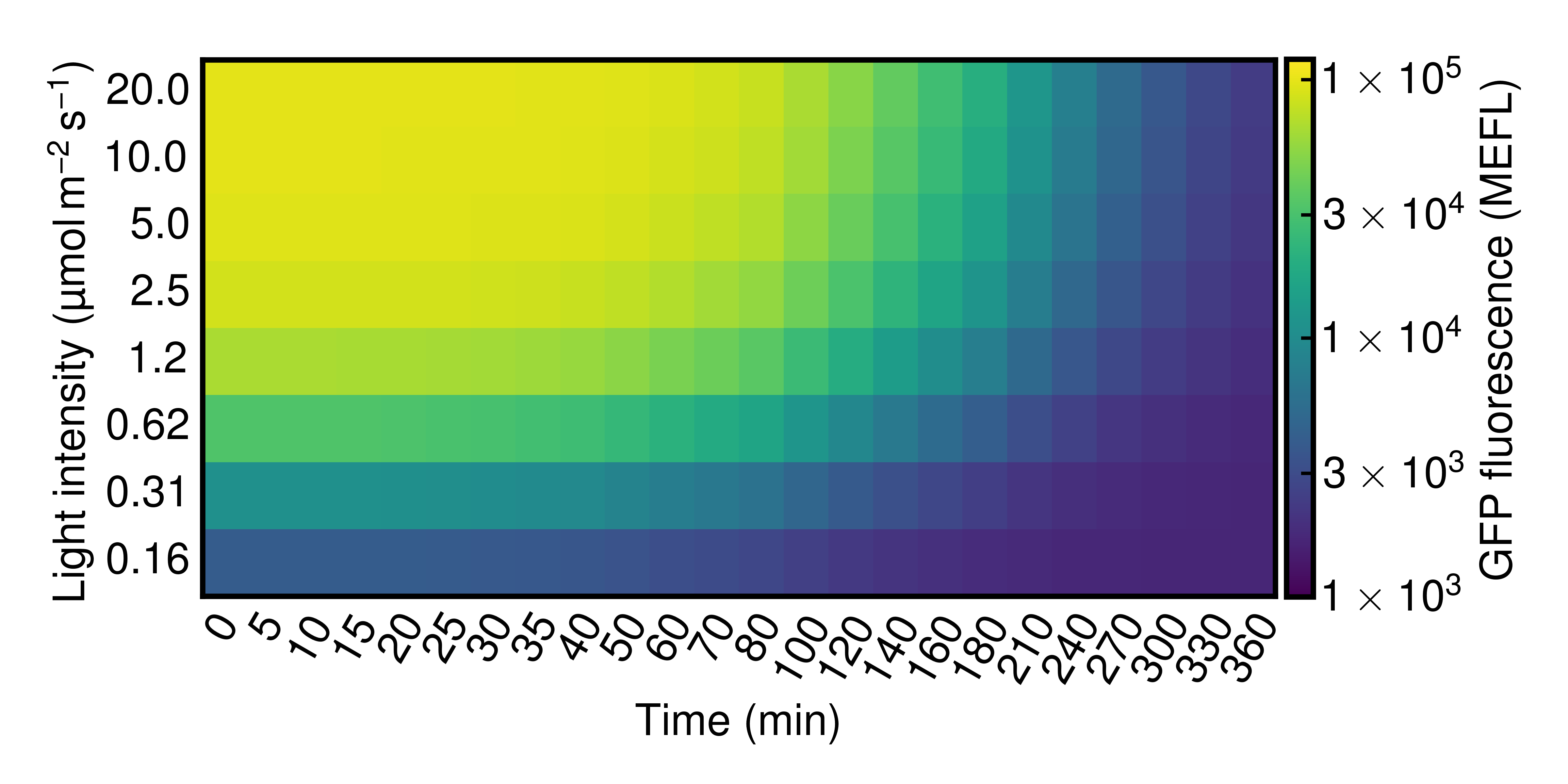

Supplement: Supplementary file 10 — Dataset EV2 [file MSB-13-926-s010.zip › dataset_ev2_ccasr_data_and_analysis/ccasr_analysis/plots/atd_logz_model_hmap.png]

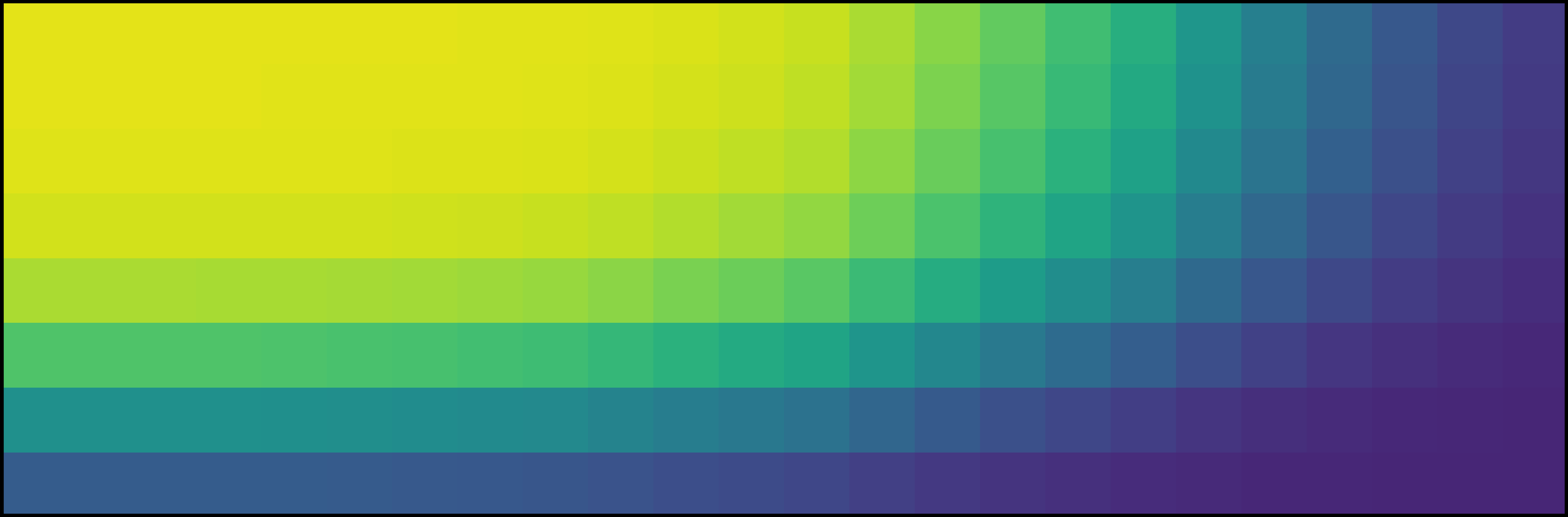

Supplement: Supplementary file 10 — Dataset EV2 [file MSB-13-926-s010.zip › dataset_ev2_ccasr_data_and_analysis/ccasr_analysis/plots/atd_logz_model_nolabel_hmap.png]

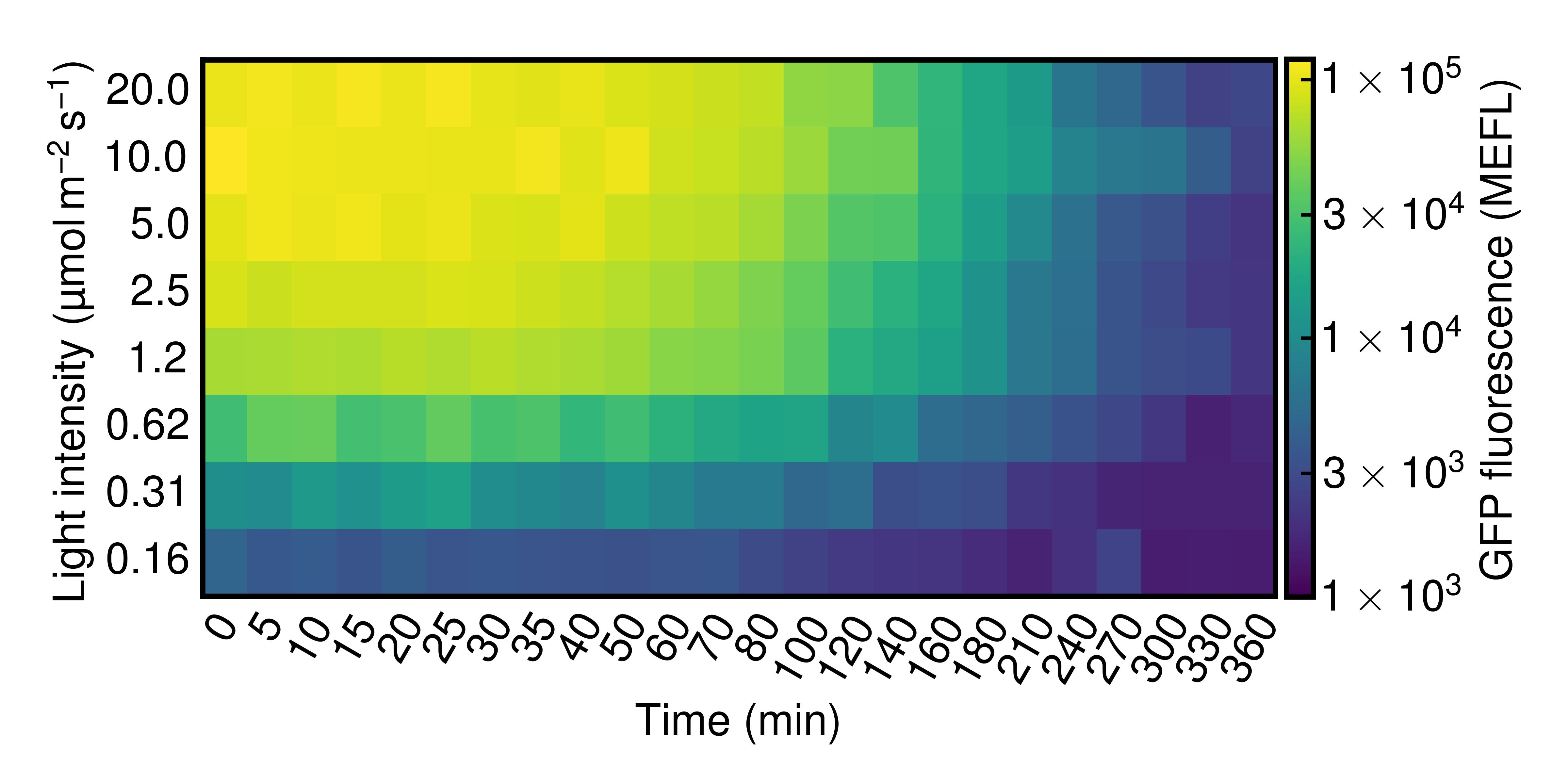

Supplement: Supplementary file 10 — Dataset EV2 [file MSB-13-926-s010.zip › dataset_ev2_ccasr_data_and_analysis/ccasr_analysis/plots/atd_logz_raw_hmap.png]

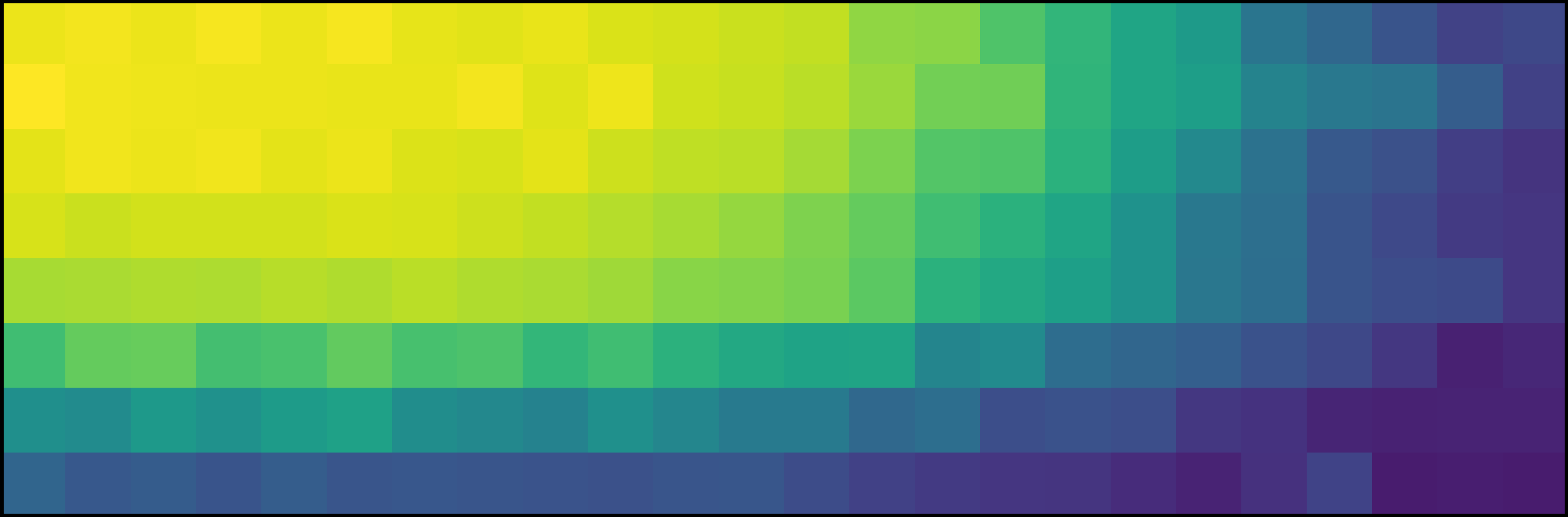

Supplement: Supplementary file 10 — Dataset EV2 [file MSB-13-926-s010.zip › dataset_ev2_ccasr_data_and_analysis/ccasr_analysis/plots/atd_logz_raw_nolabel_hmap.png]

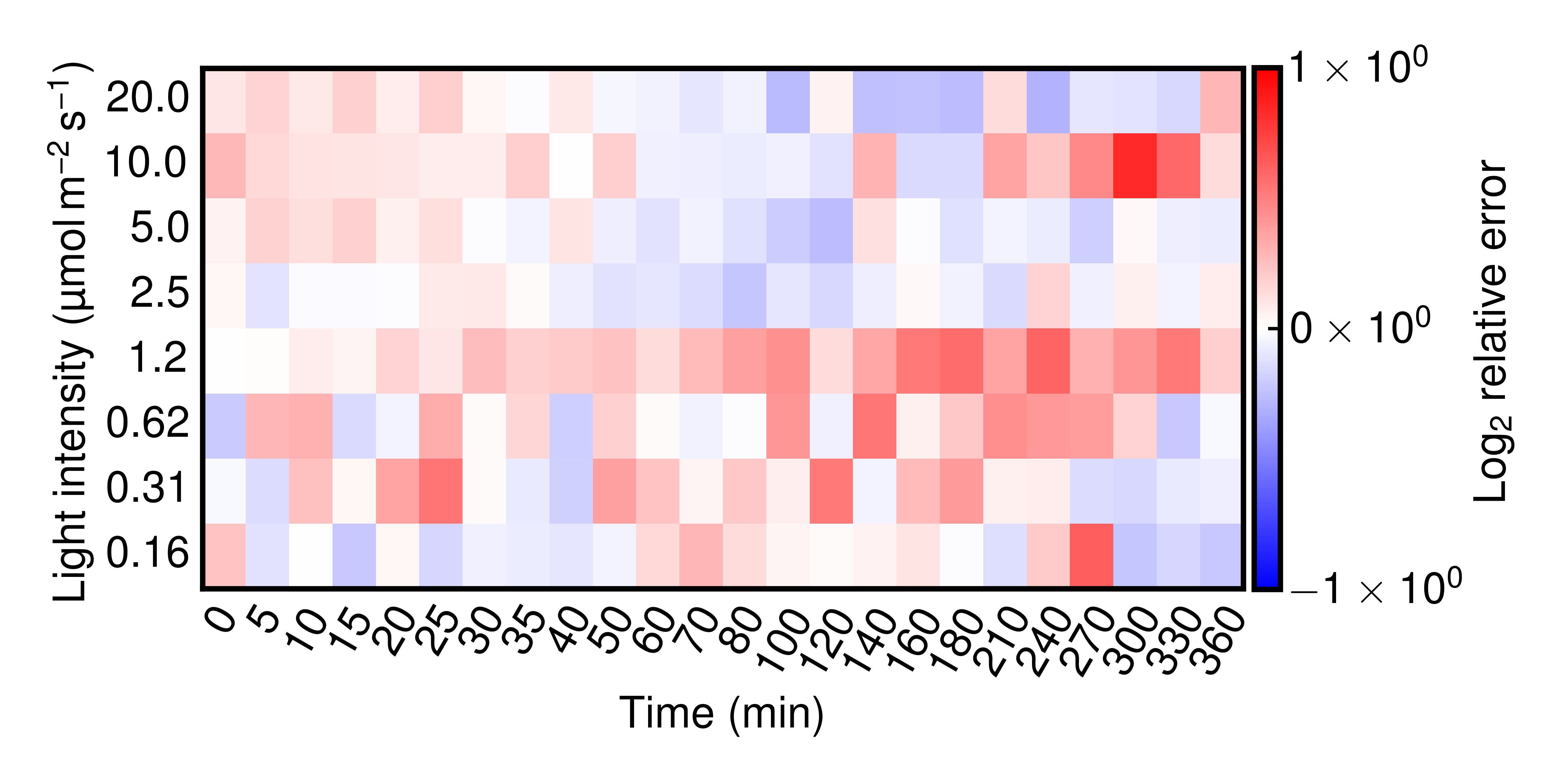

Supplement: Supplementary file 10 — Dataset EV2 [file MSB-13-926-s010.zip › dataset_ev2_ccasr_data_and_analysis/ccasr_analysis/plots/atd_rel_residual_hmap.png]

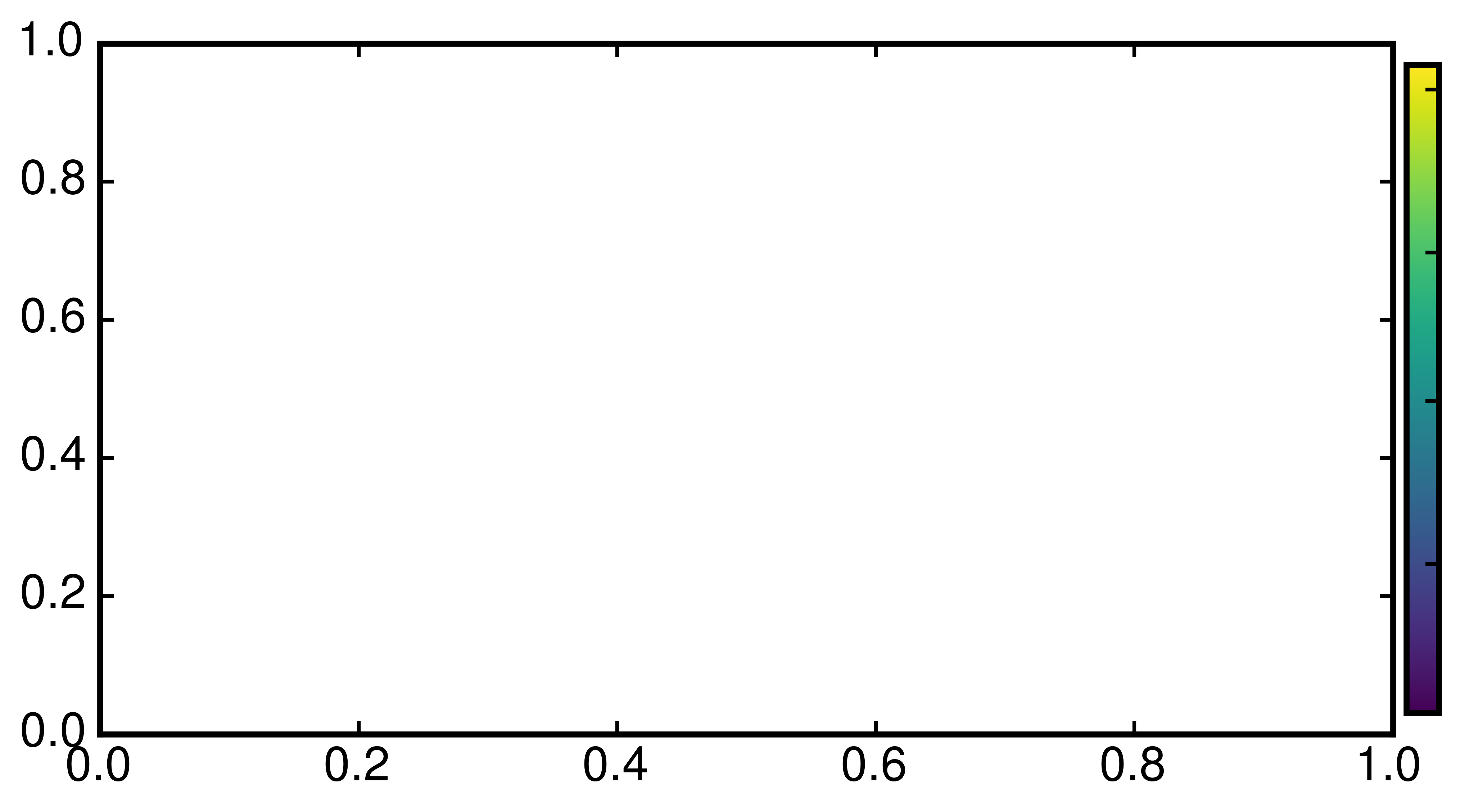

Supplement: Supplementary file 10 — Dataset EV2 [file MSB-13-926-s010.zip › dataset_ev2_ccasr_data_and_analysis/ccasr_analysis/plots/cbar.png]

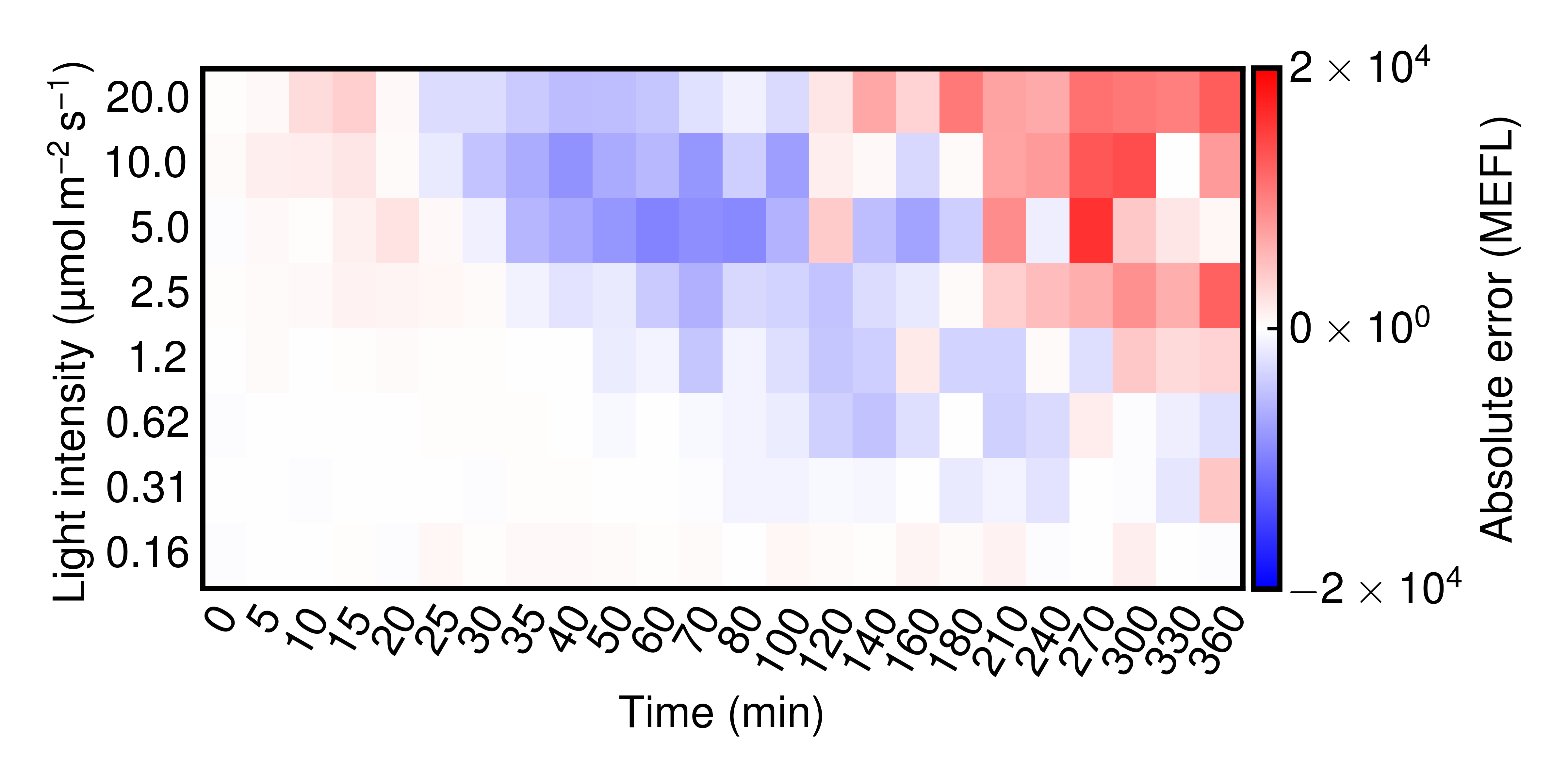

Supplement: Supplementary file 10 — Dataset EV2 [file MSB-13-926-s010.zip › dataset_ev2_ccasr_data_and_analysis/ccasr_analysis/plots/dta_abs_residual_hmap.png]

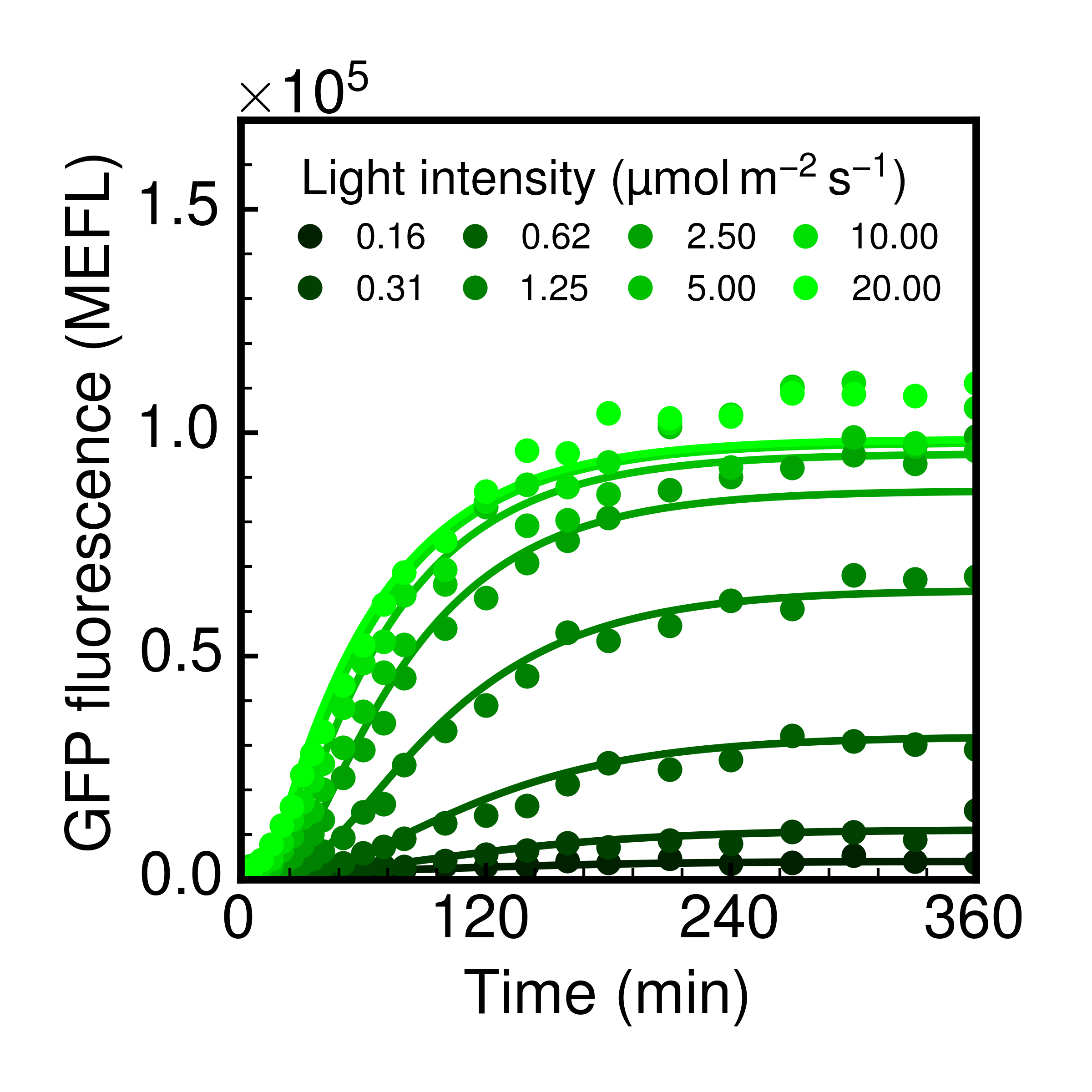

Supplement: Supplementary file 10 — Dataset EV2 [file MSB-13-926-s010.zip › dataset_ev2_ccasr_data_and_analysis/ccasr_analysis/plots/dta_lin_model.png]

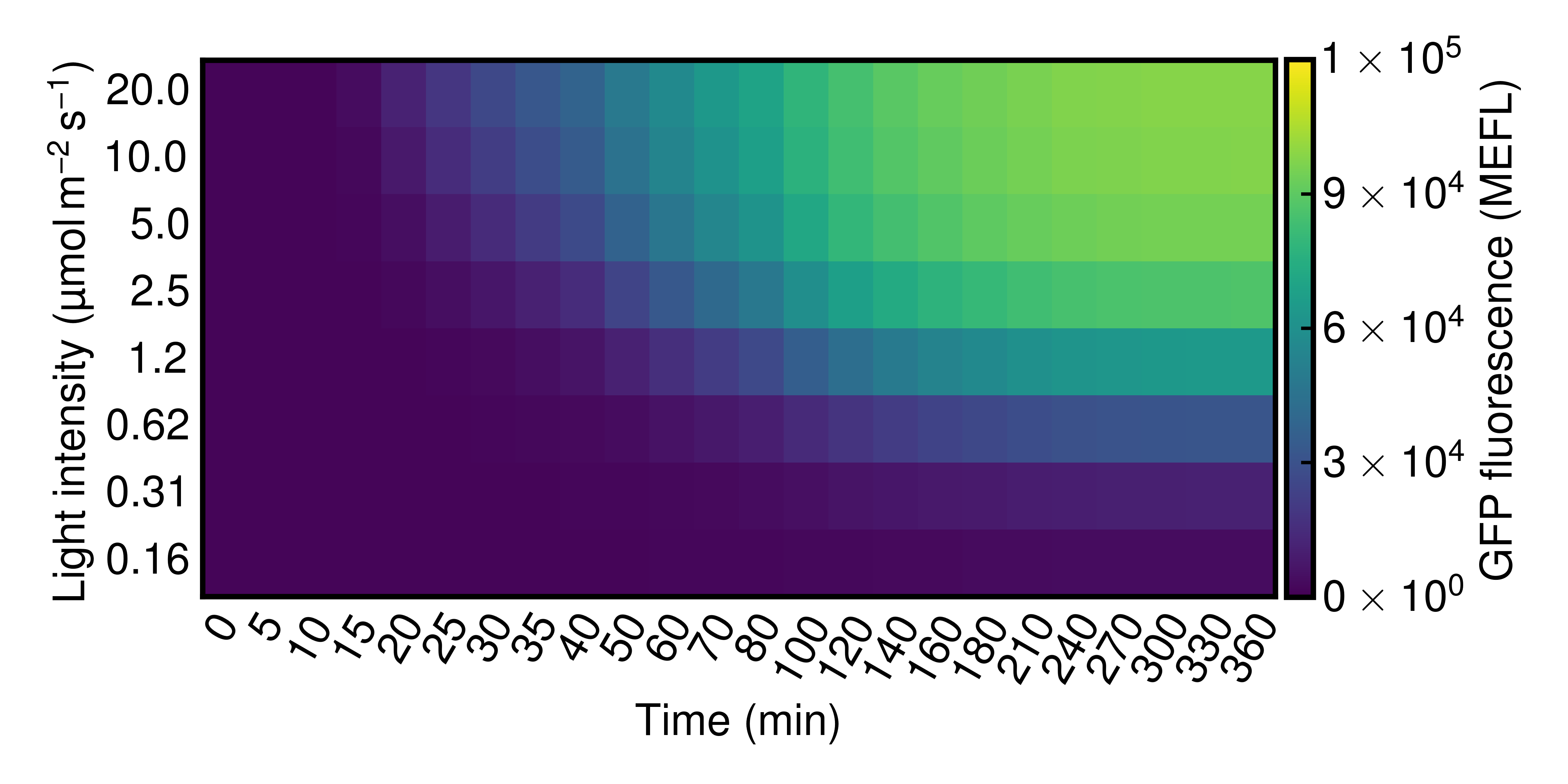

Supplement: Supplementary file 10 — Dataset EV2 [file MSB-13-926-s010.zip › dataset_ev2_ccasr_data_and_analysis/ccasr_analysis/plots/dta_lin_model_hmap.png]

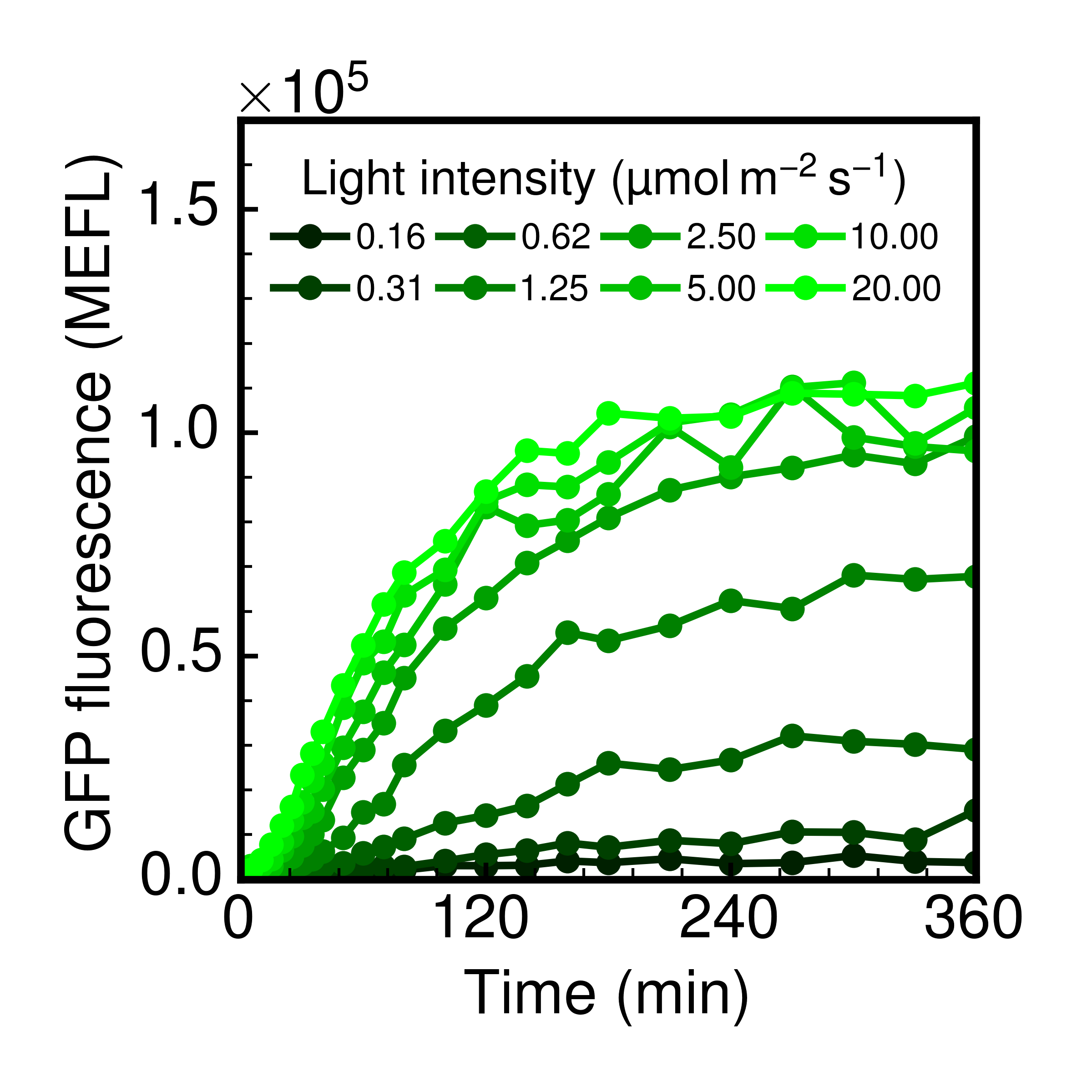

Supplement: Supplementary file 10 — Dataset EV2 [file MSB-13-926-s010.zip › dataset_ev2_ccasr_data_and_analysis/ccasr_analysis/plots/dta_lin_raw.png]

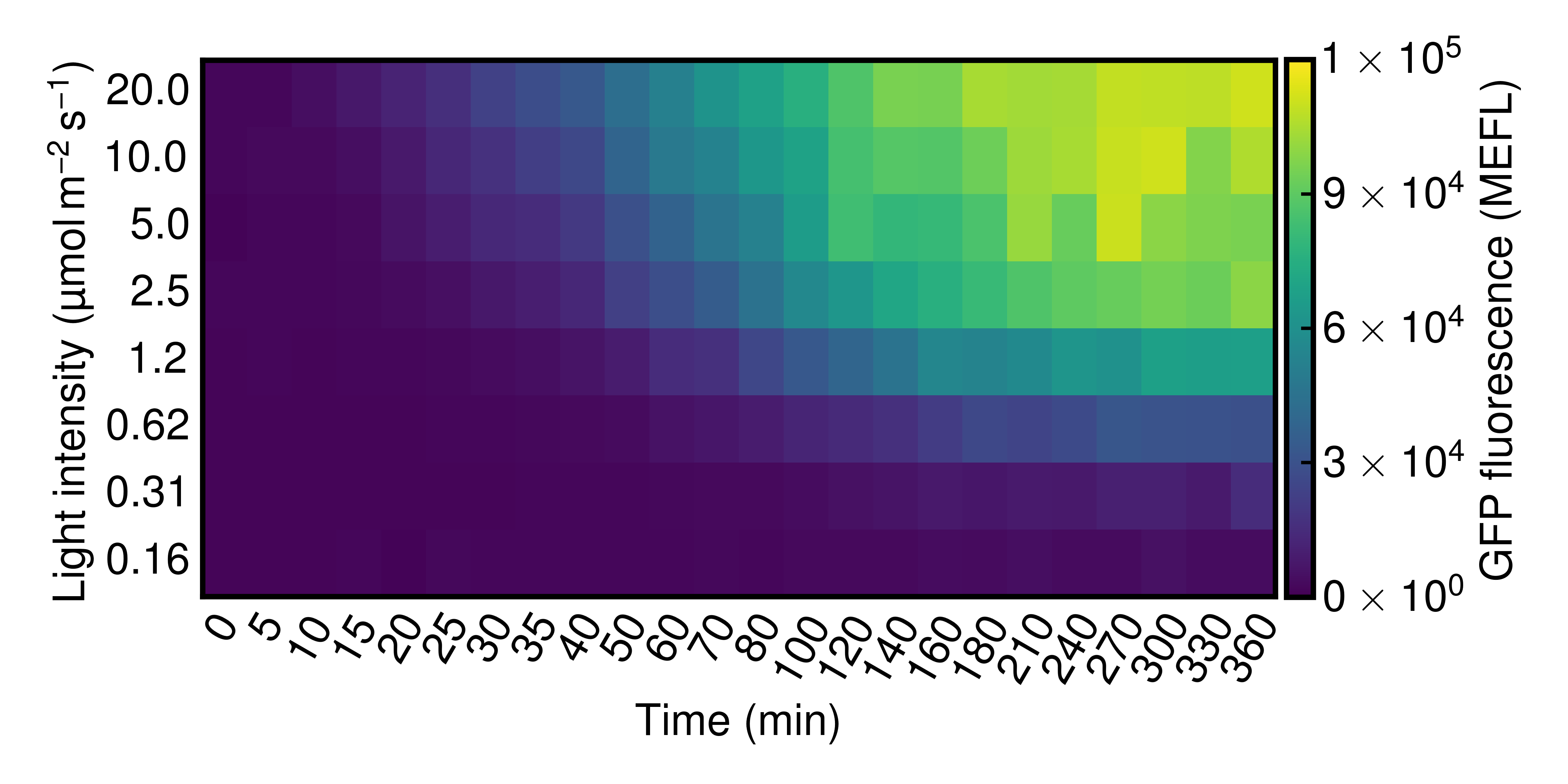

Supplement: Supplementary file 10 — Dataset EV2 [file MSB-13-926-s010.zip › dataset_ev2_ccasr_data_and_analysis/ccasr_analysis/plots/dta_lin_raw_hmap.png]

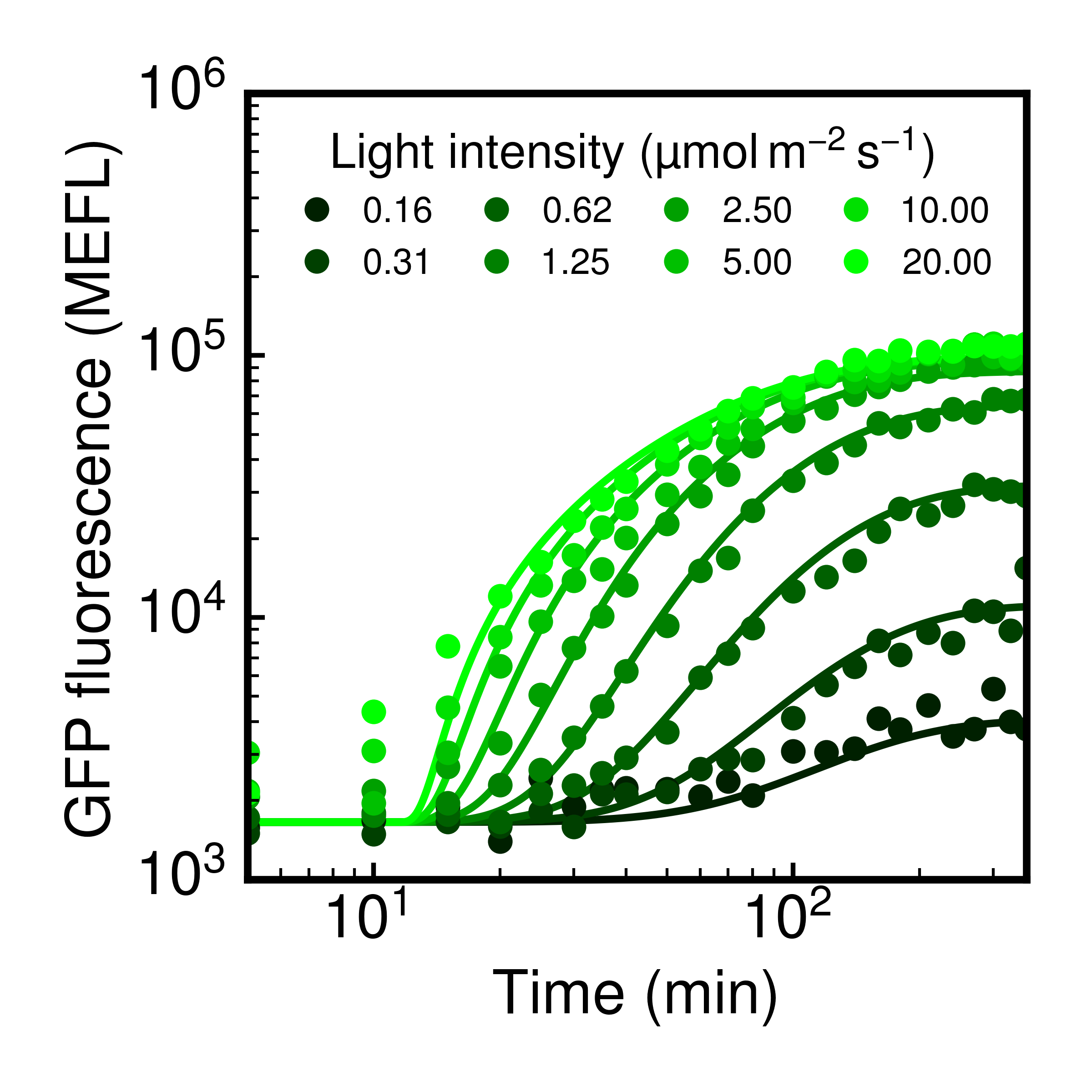

Supplement: Supplementary file 10 — Dataset EV2 [file MSB-13-926-s010.zip › dataset_ev2_ccasr_data_and_analysis/ccasr_analysis/plots/dta_logxy_model.png]

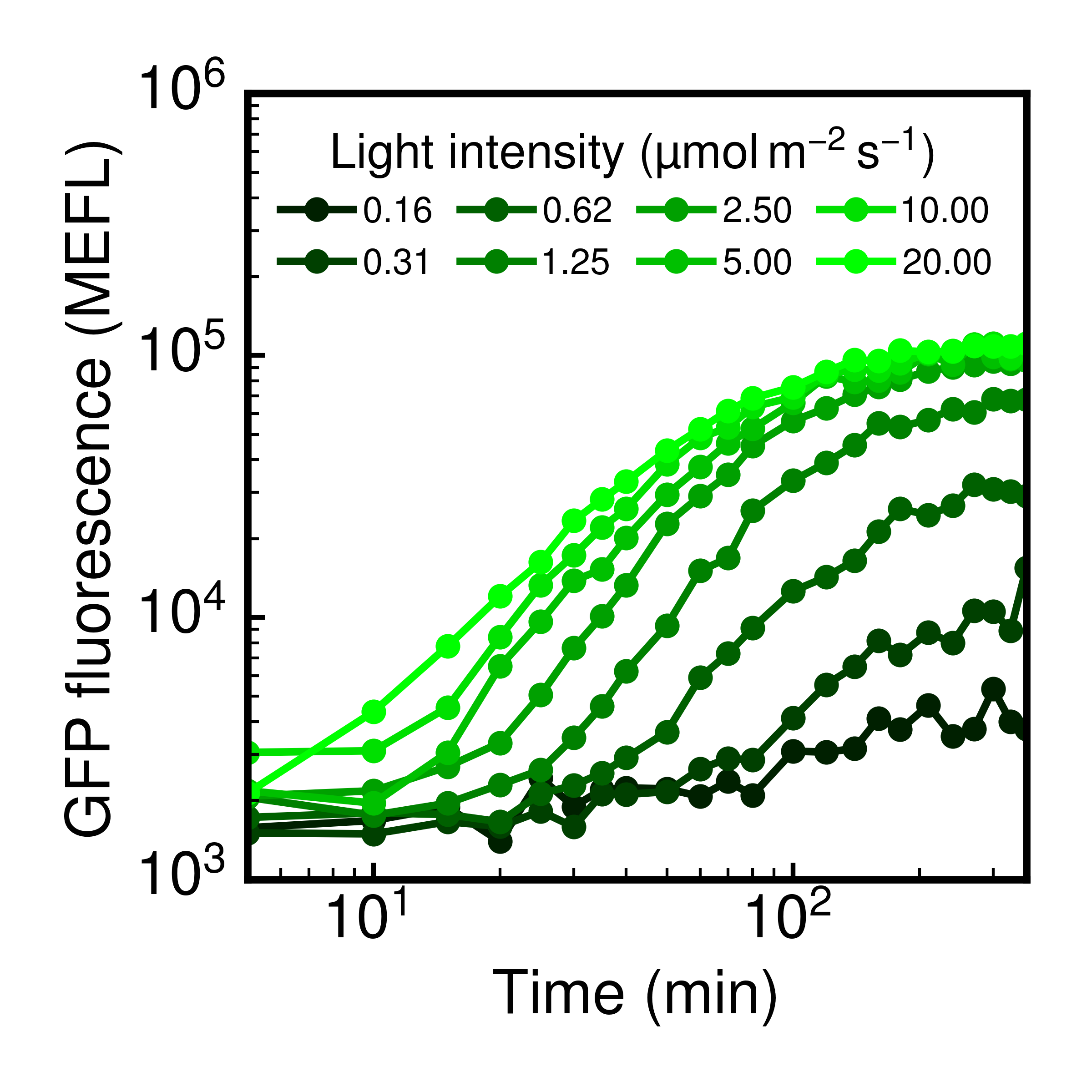

Supplement: Supplementary file 10 — Dataset EV2 [file MSB-13-926-s010.zip › dataset_ev2_ccasr_data_and_analysis/ccasr_analysis/plots/dta_logxy_raw.png]

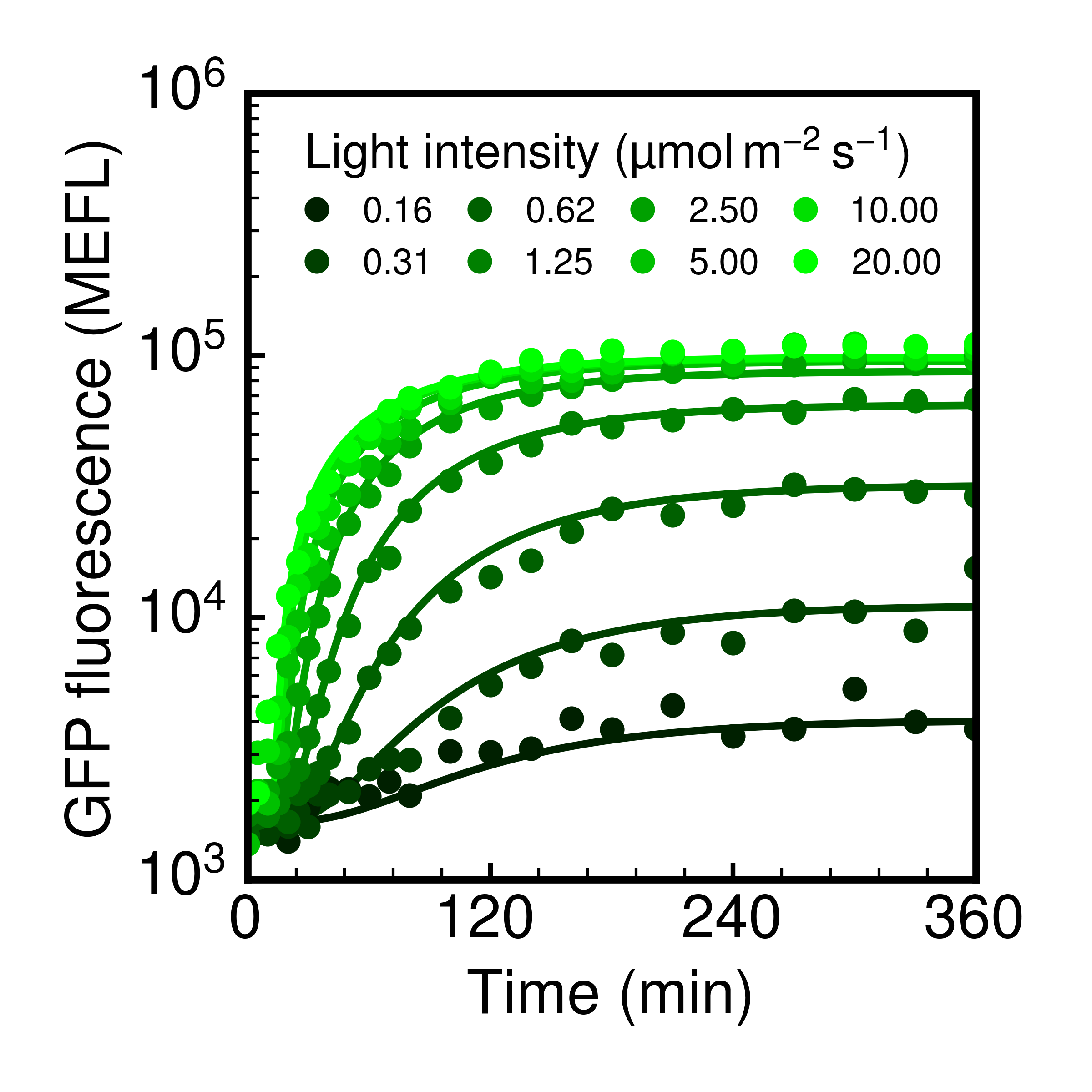

Supplement: Supplementary file 10 — Dataset EV2 [file MSB-13-926-s010.zip › dataset_ev2_ccasr_data_and_analysis/ccasr_analysis/plots/dta_logy_model.png]

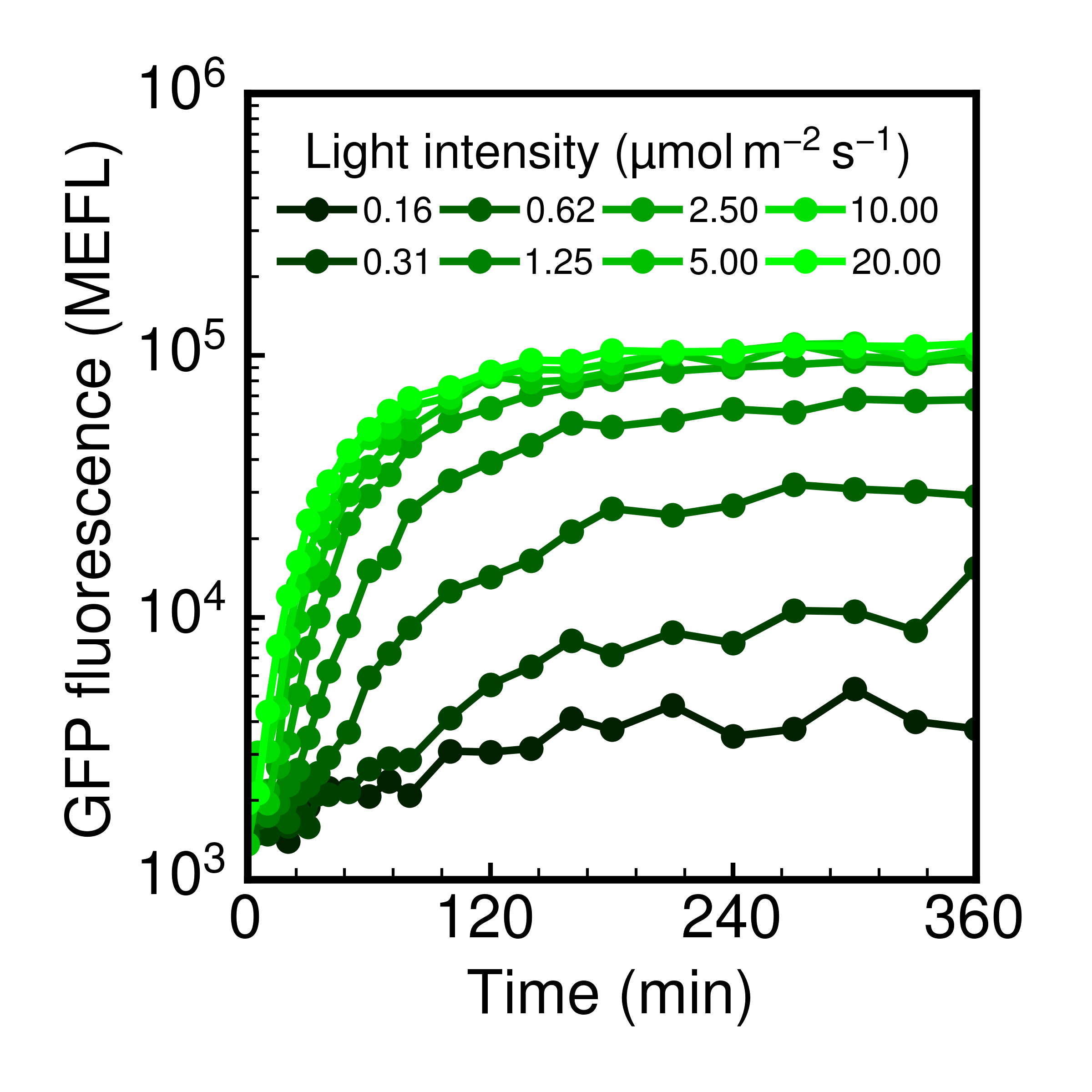

Supplement: Supplementary file 10 — Dataset EV2 [file MSB-13-926-s010.zip › dataset_ev2_ccasr_data_and_analysis/ccasr_analysis/plots/dta_logy_raw.png]

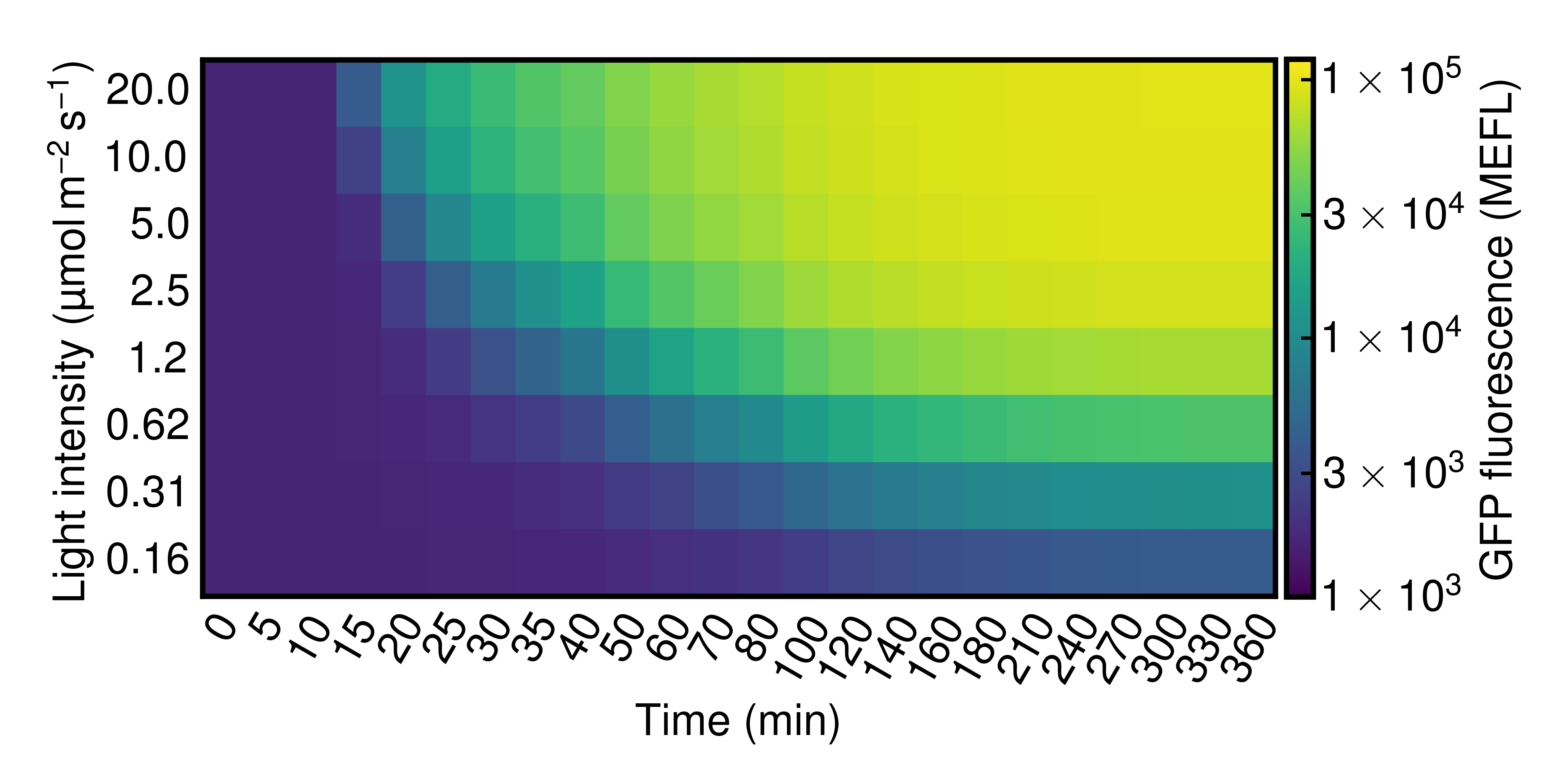

Supplement: Supplementary file 10 — Dataset EV2 [file MSB-13-926-s010.zip › dataset_ev2_ccasr_data_and_analysis/ccasr_analysis/plots/dta_logz_model_hmap.png]

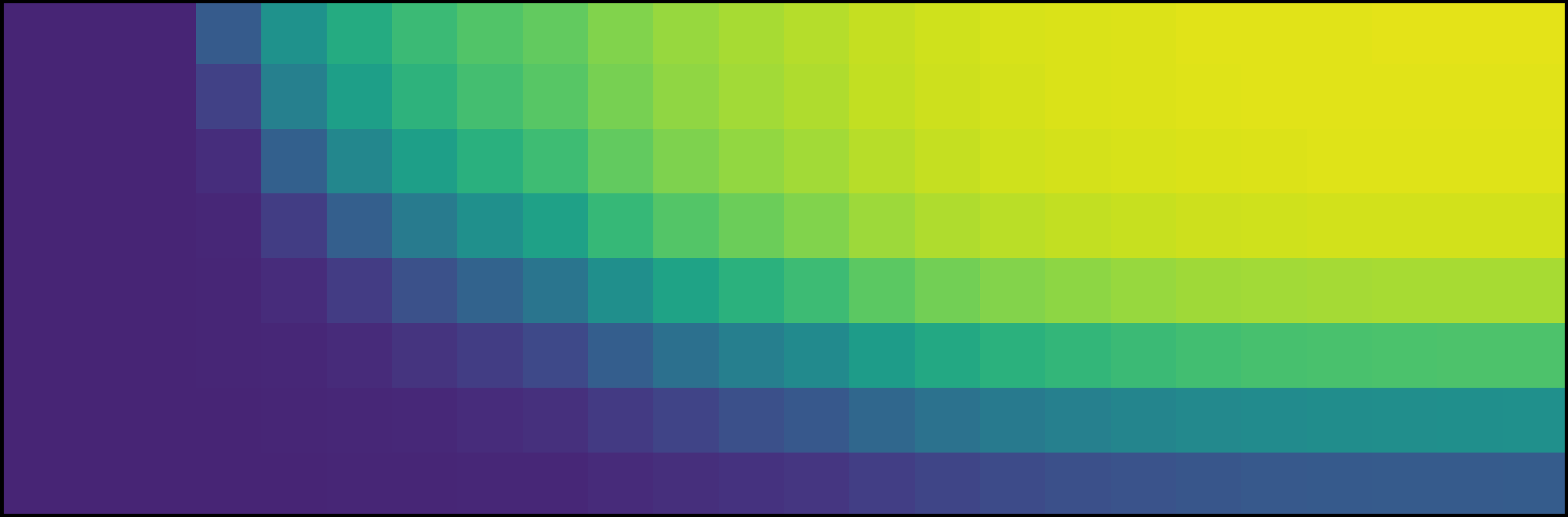

Supplement: Supplementary file 10 — Dataset EV2 [file MSB-13-926-s010.zip › dataset_ev2_ccasr_data_and_analysis/ccasr_analysis/plots/dta_logz_model_nolabel_hmap.png]

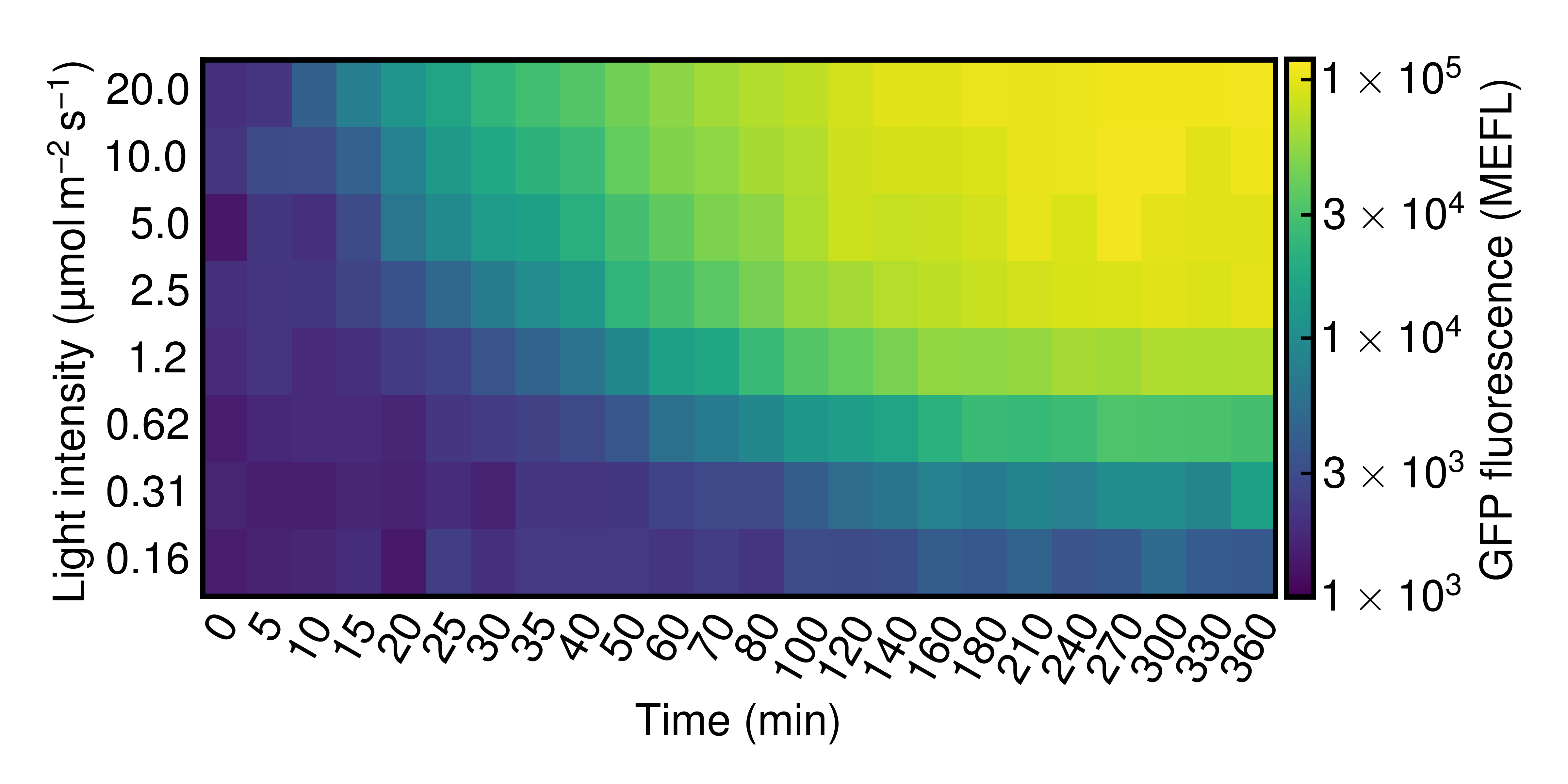

Supplement: Supplementary file 10 — Dataset EV2 [file MSB-13-926-s010.zip › dataset_ev2_ccasr_data_and_analysis/ccasr_analysis/plots/dta_logz_raw_hmap.png]

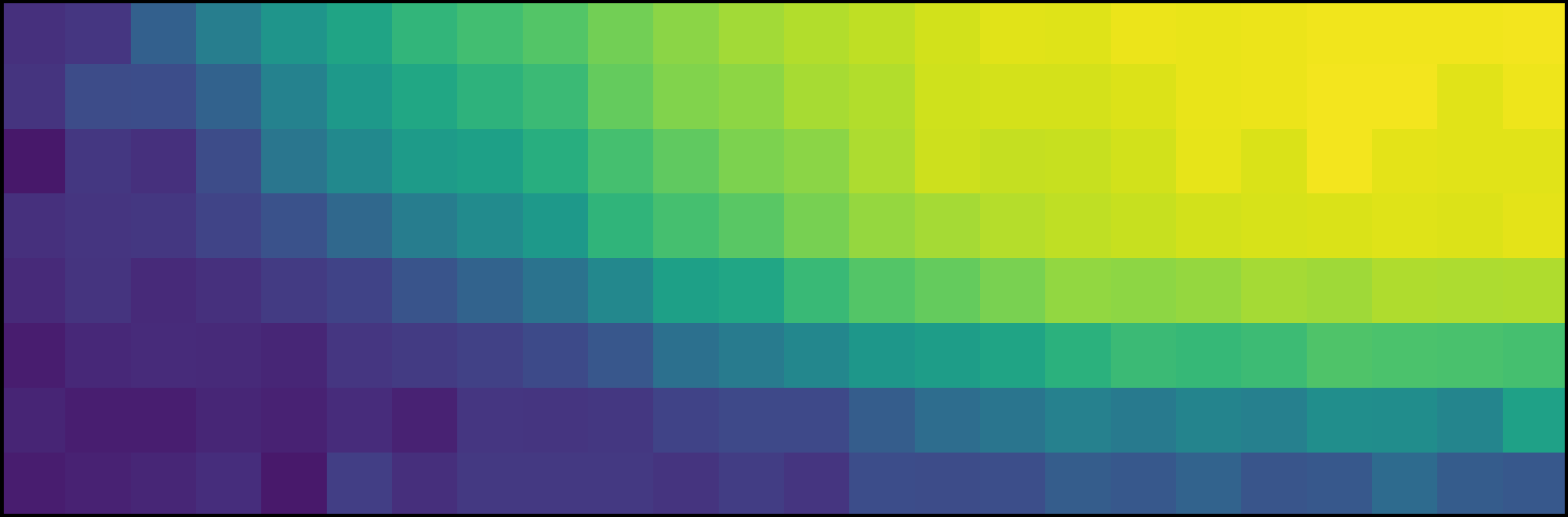

Supplement: Supplementary file 10 — Dataset EV2 [file MSB-13-926-s010.zip › dataset_ev2_ccasr_data_and_analysis/ccasr_analysis/plots/dta_logz_raw_nolabel_hmap.png]

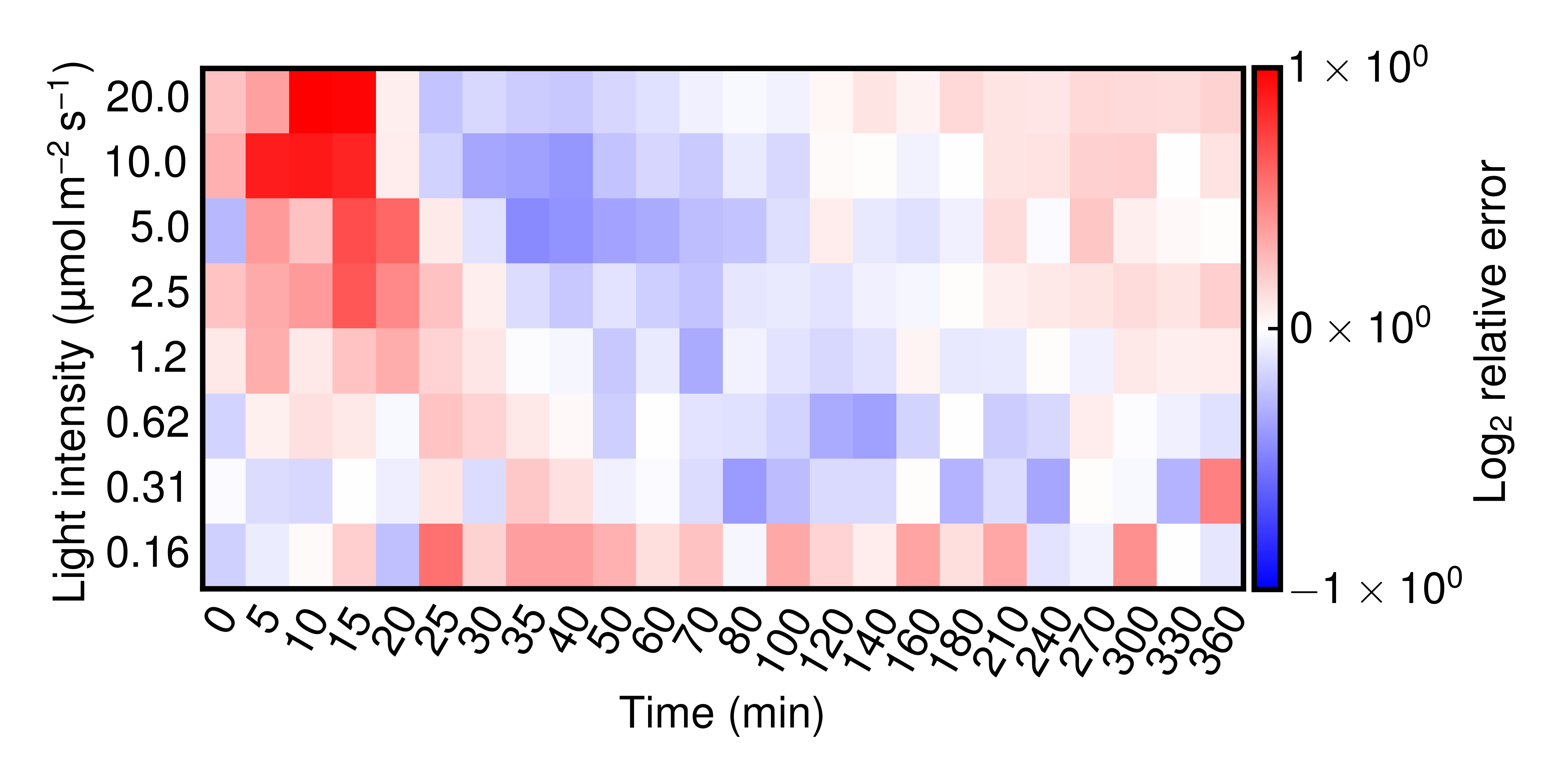

Supplement: Supplementary file 10 — Dataset EV2 [file MSB-13-926-s010.zip › dataset_ev2_ccasr_data_and_analysis/ccasr_analysis/plots/dta_rel_residual_hmap.png]

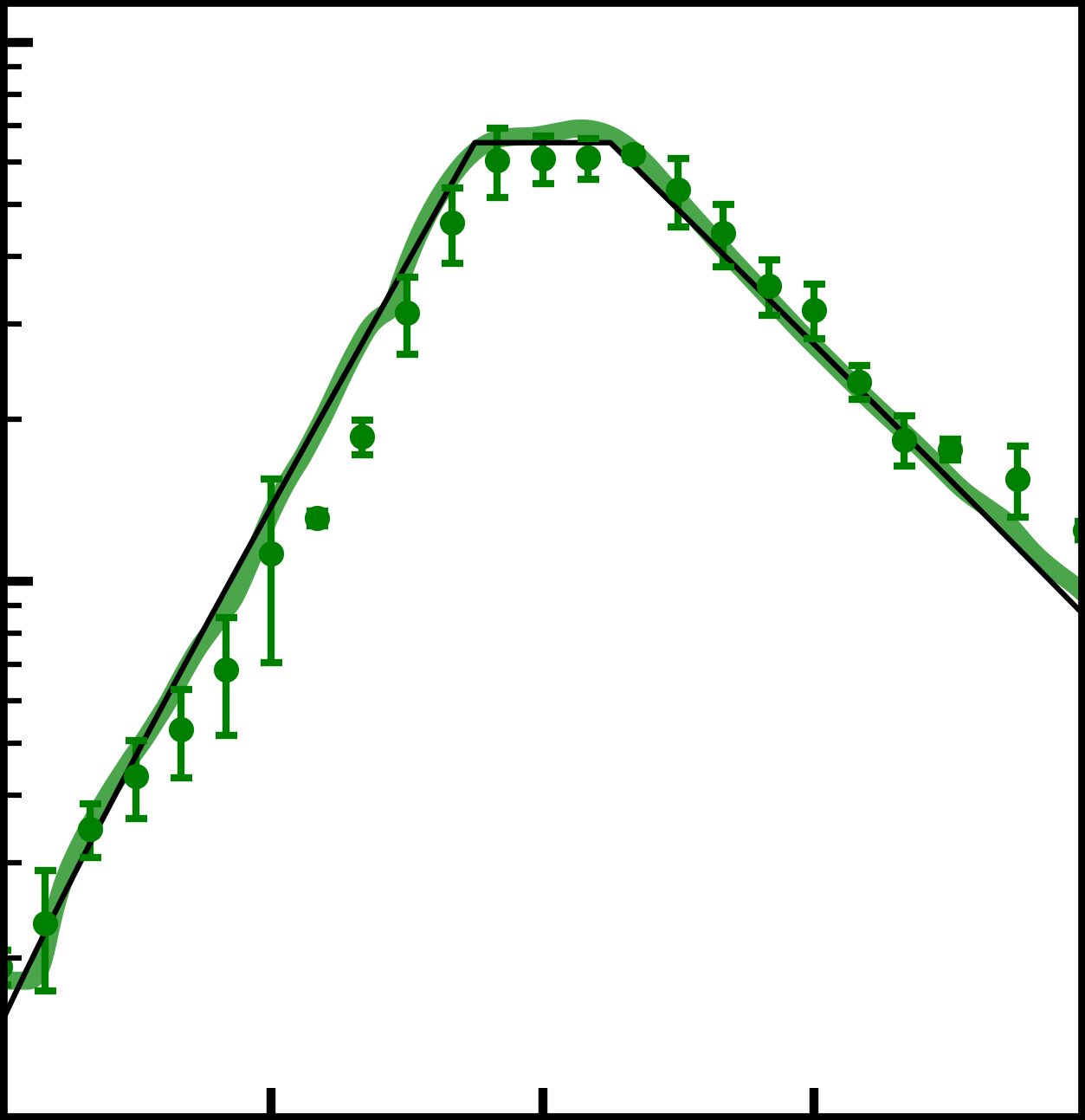

Supplement: Supplementary file 10 — Dataset EV2 [file MSB-13-926-s010.zip › dataset_ev2_ccasr_data_and_analysis/ccasr_analysis/plots/dv-comp_logy_full_data.png]

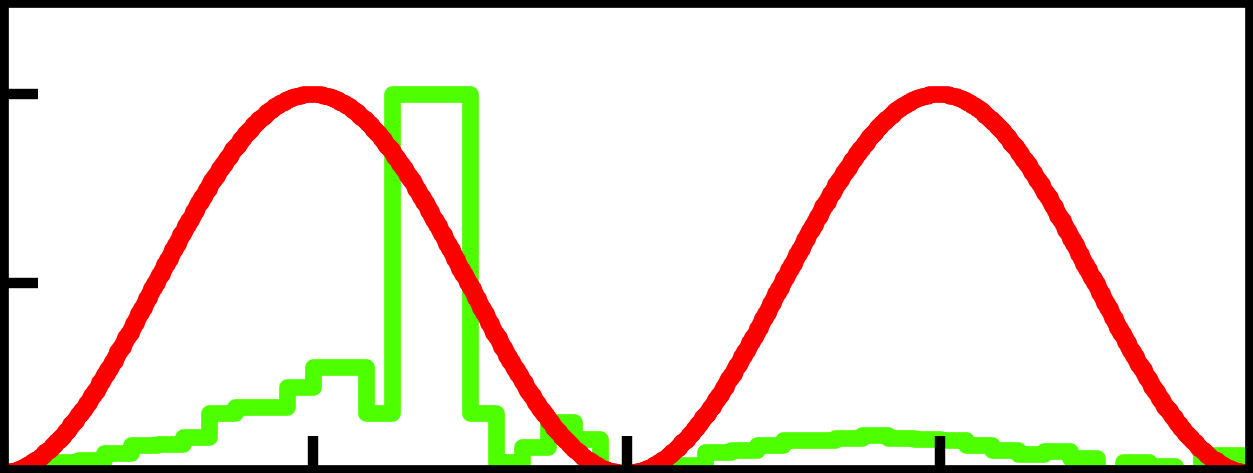

Supplement: Supplementary file 10 — Dataset EV2 [file MSB-13-926-s010.zip › dataset_ev2_ccasr_data_and_analysis/ccasr_analysis/plots/dv-comp_logy_full_intlin.png]

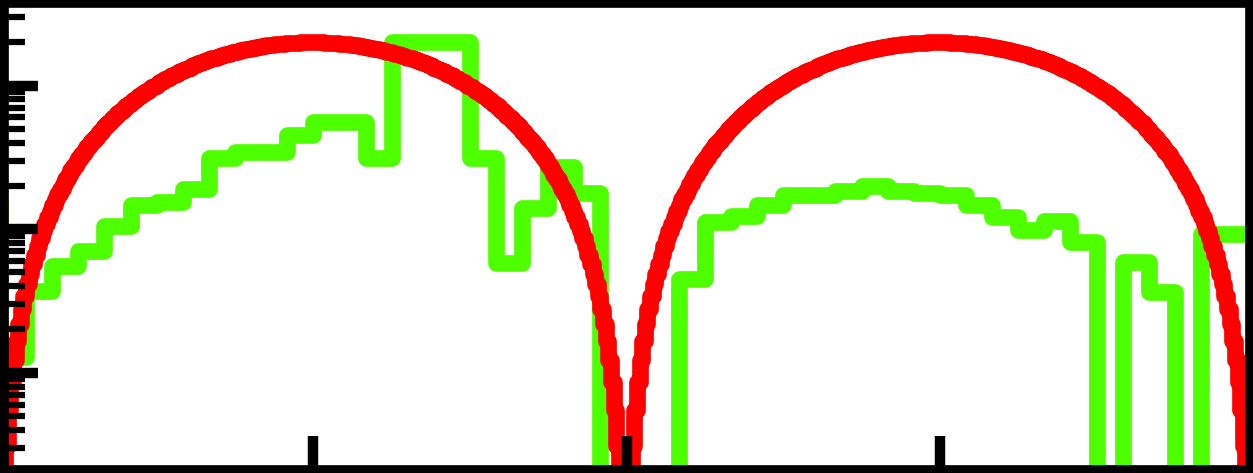

Supplement: Supplementary file 10 — Dataset EV2 [file MSB-13-926-s010.zip › dataset_ev2_ccasr_data_and_analysis/ccasr_analysis/plots/dv-comp_logy_full_intlog.png]

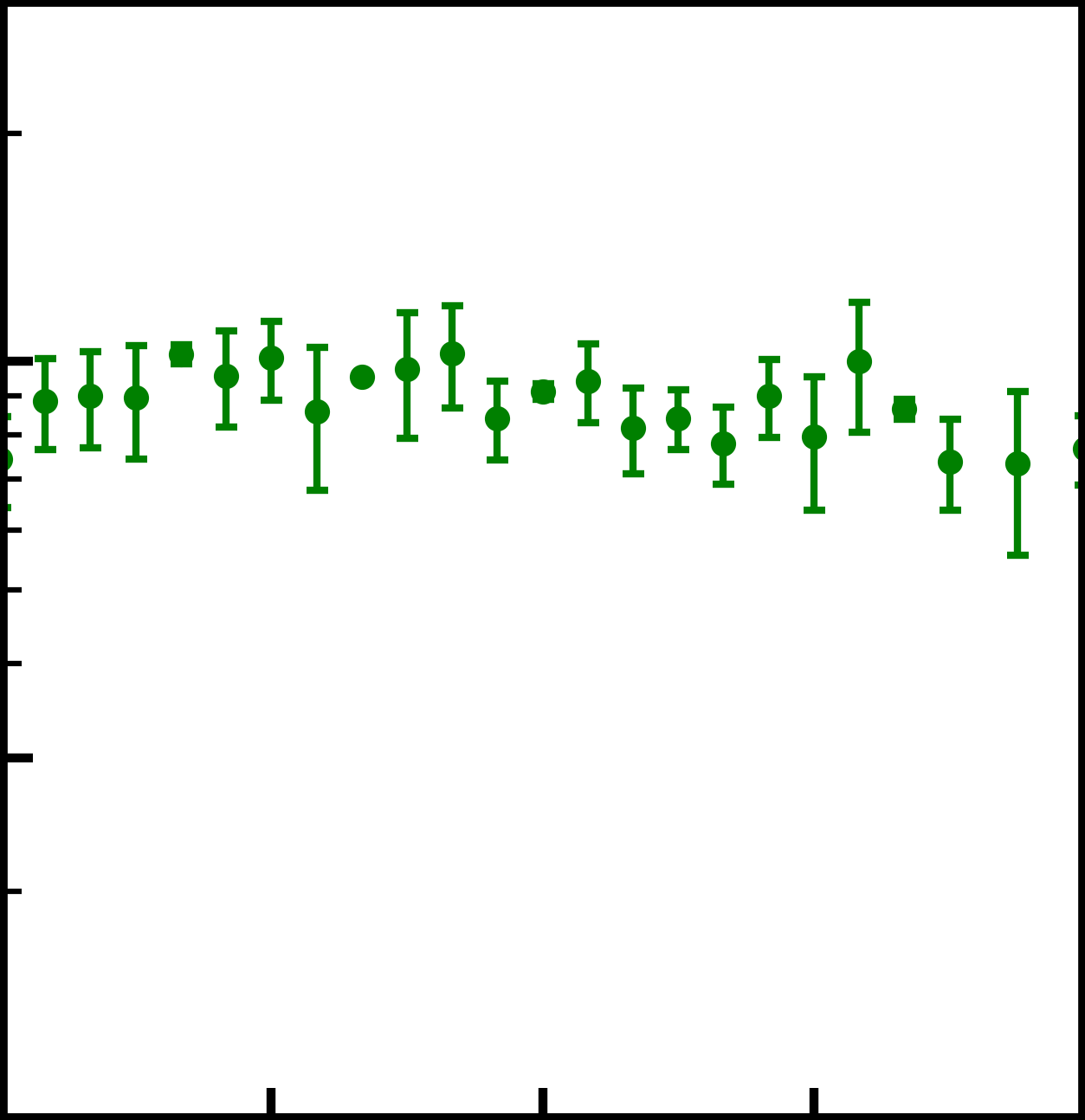

Supplement: Supplementary file 10 — Dataset EV2 [file MSB-13-926-s010.zip › dataset_ev2_ccasr_data_and_analysis/ccasr_analysis/plots/dv-comp_logy_full_od_data.png]

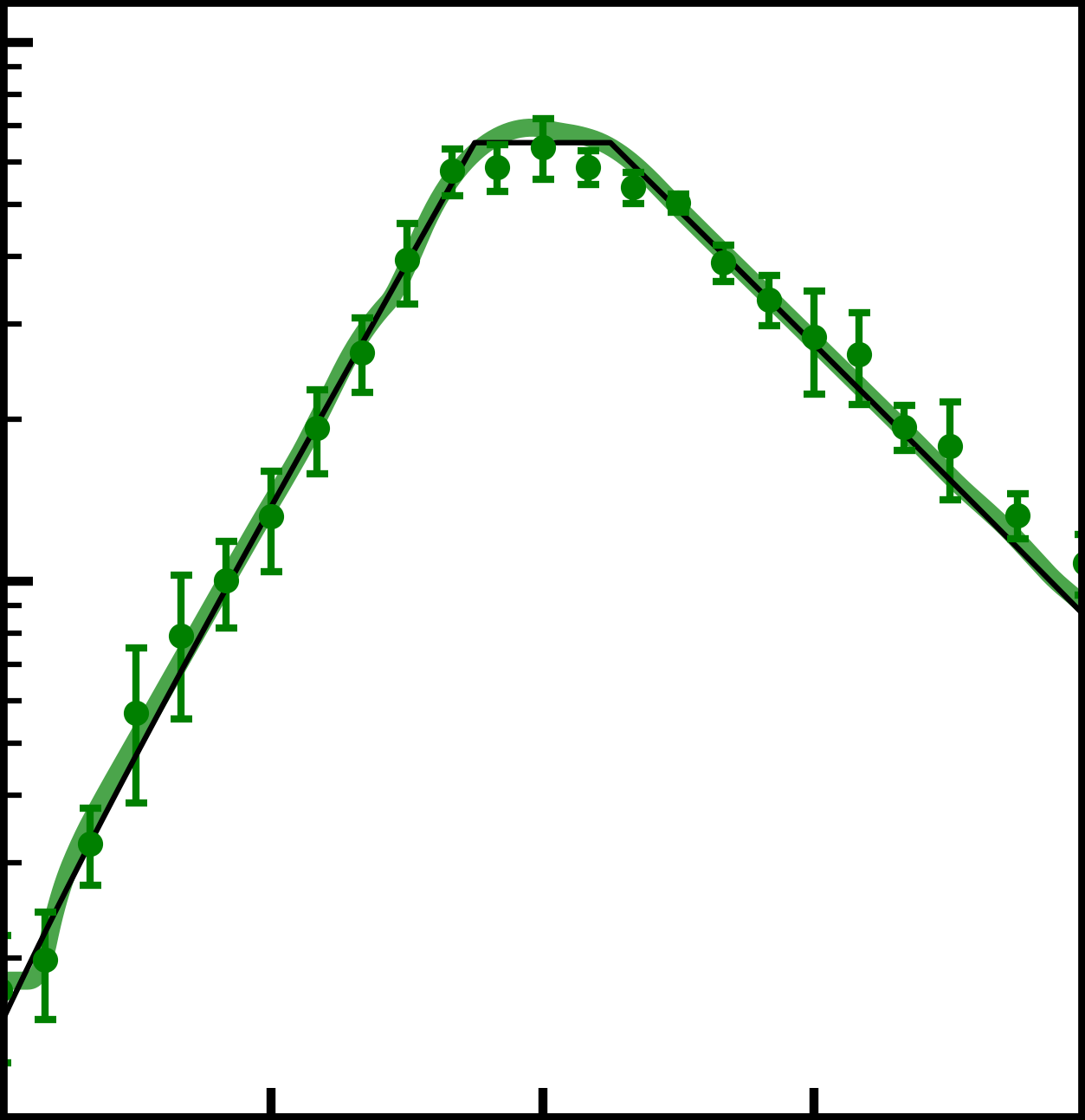

Supplement: Supplementary file 10 — Dataset EV2 [file MSB-13-926-s010.zip › dataset_ev2_ccasr_data_and_analysis/ccasr_analysis/plots/dv-mono-g_logy_full_data.png]

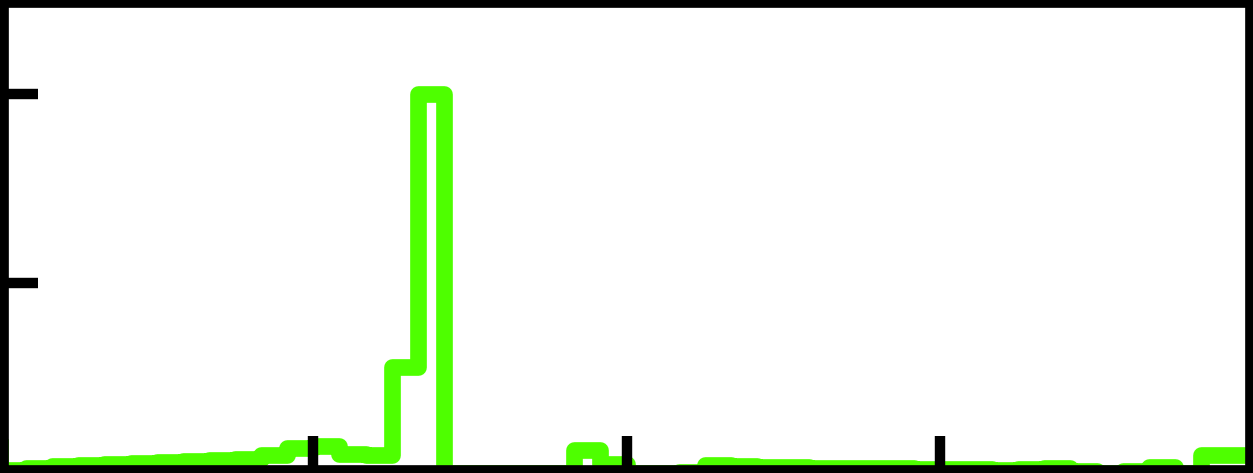

Supplement: Supplementary file 10 — Dataset EV2 [file MSB-13-926-s010.zip › dataset_ev2_ccasr_data_and_analysis/ccasr_analysis/plots/dv-mono-g_logy_full_intlin.png]

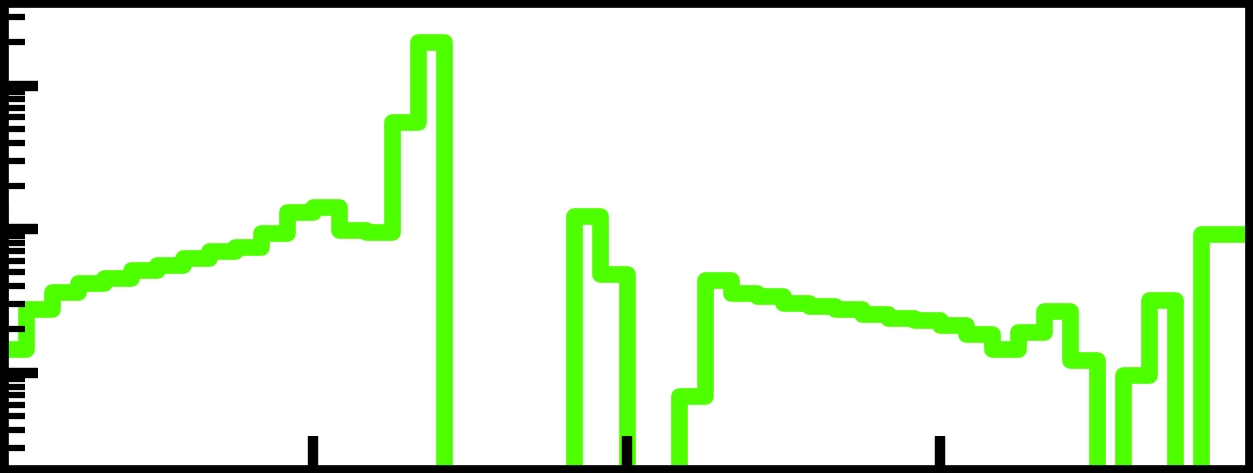

Supplement: Supplementary file 10 — Dataset EV2 [file MSB-13-926-s010.zip › dataset_ev2_ccasr_data_and_analysis/ccasr_analysis/plots/dv-mono-g_logy_full_intlog.png]

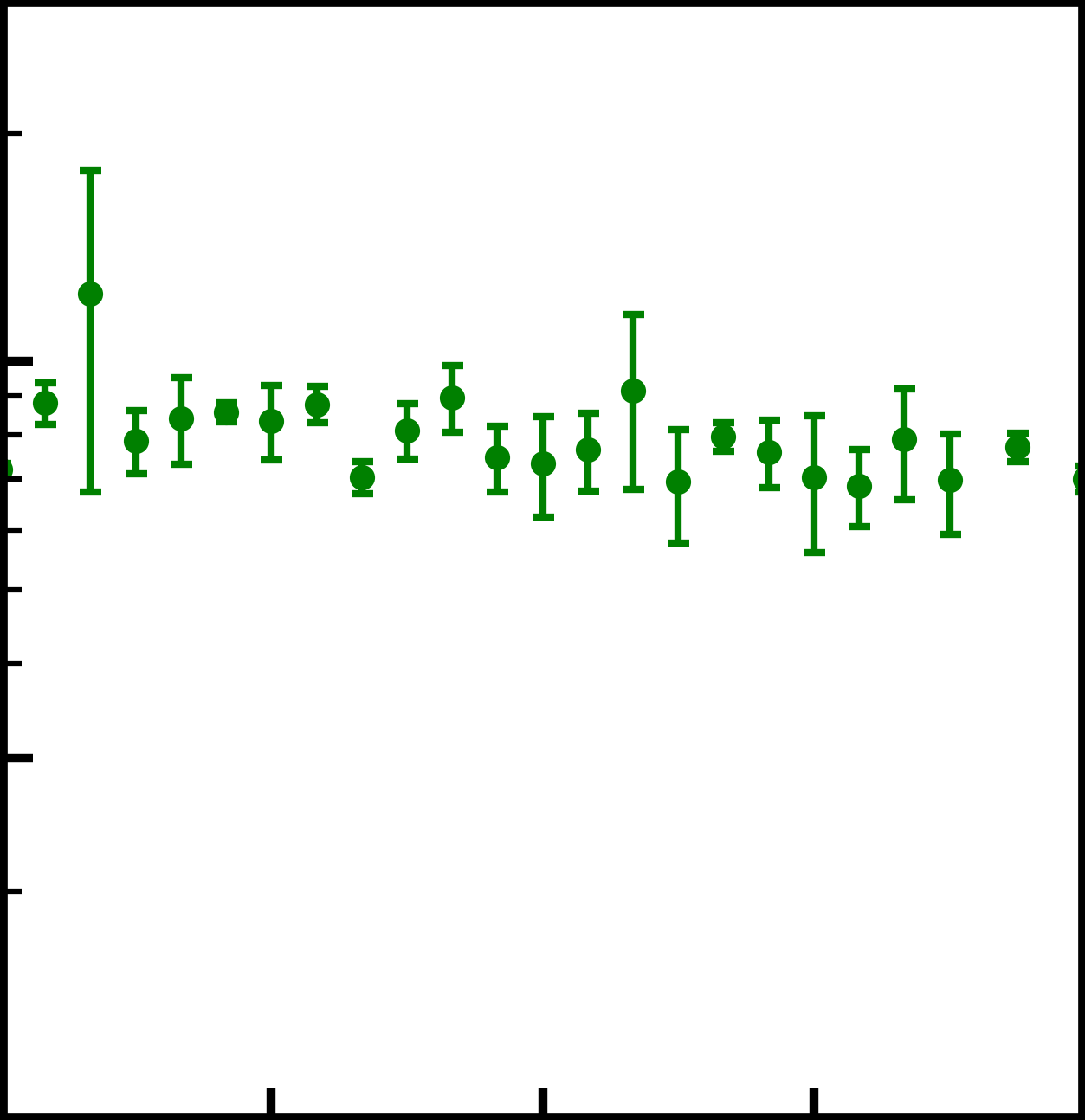

Supplement: Supplementary file 10 — Dataset EV2 [file MSB-13-926-s010.zip › dataset_ev2_ccasr_data_and_analysis/ccasr_analysis/plots/dv-mono-g_logy_full_od_data.png]

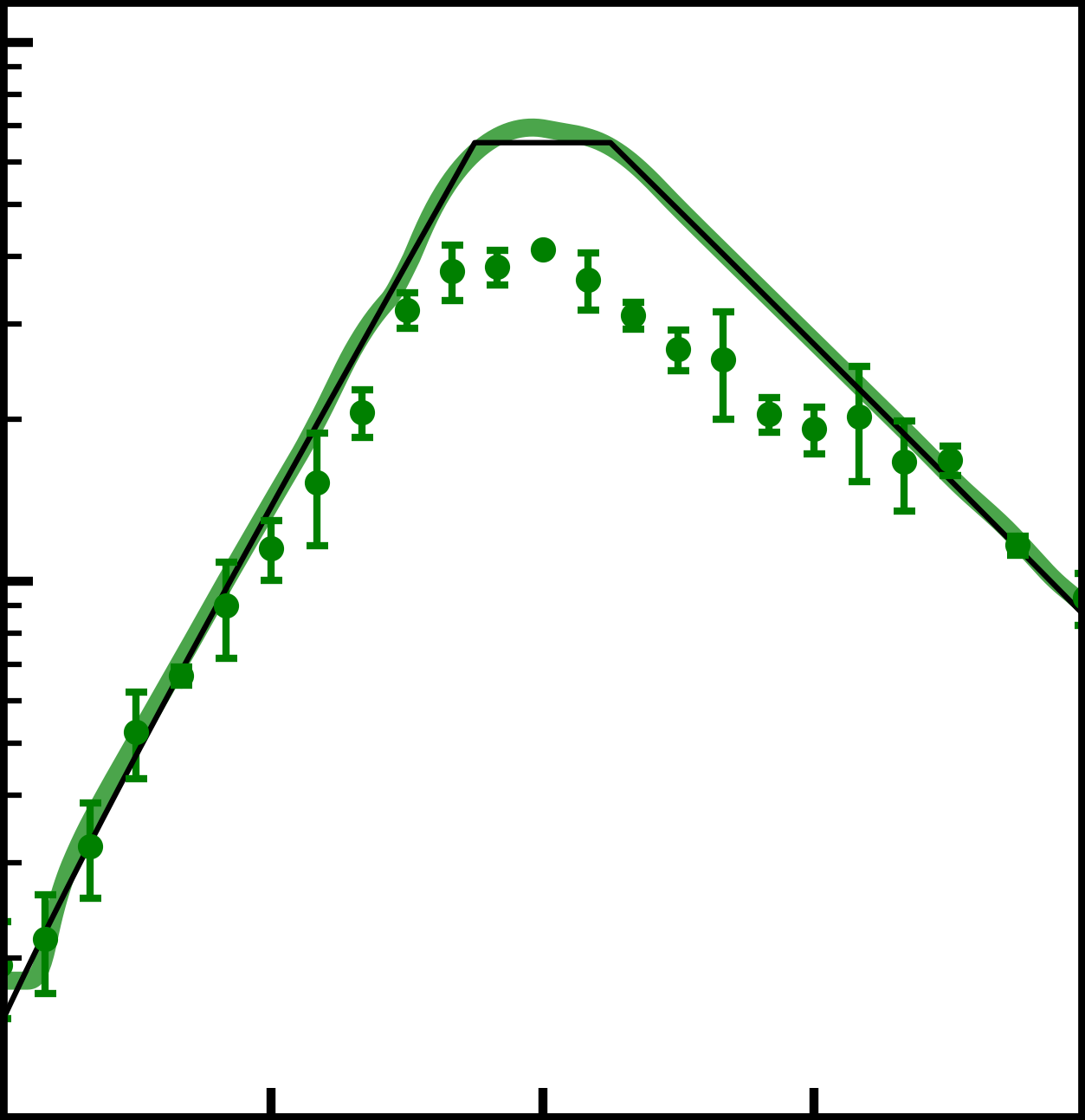

Supplement: Supplementary file 10 — Dataset EV2 [file MSB-13-926-s010.zip › dataset_ev2_ccasr_data_and_analysis/ccasr_analysis/plots/dv-mono-uv_logy_full_data.png]

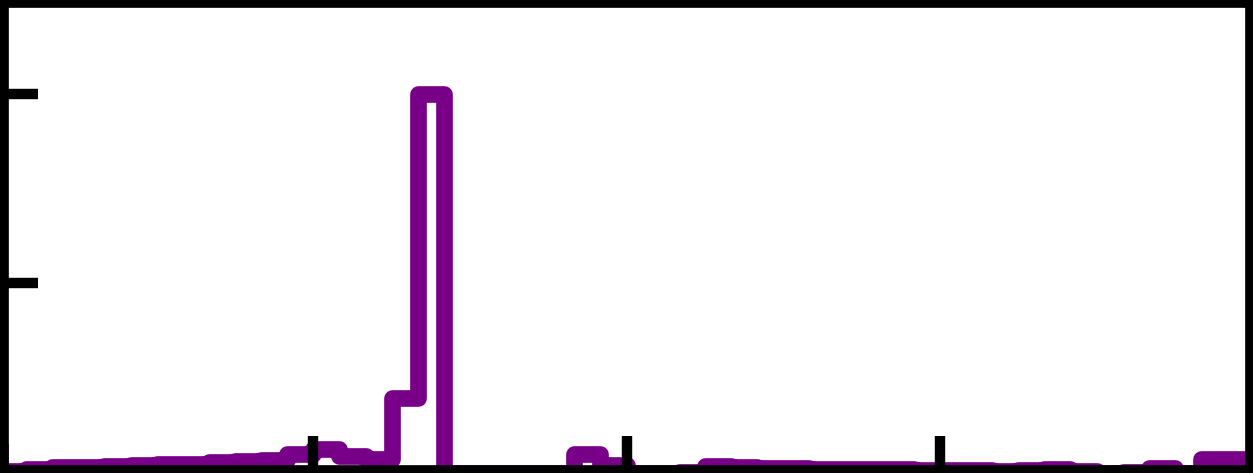

Supplement: Supplementary file 10 — Dataset EV2 [file MSB-13-926-s010.zip › dataset_ev2_ccasr_data_and_analysis/ccasr_analysis/plots/dv-mono-uv_logy_full_intlin.png]

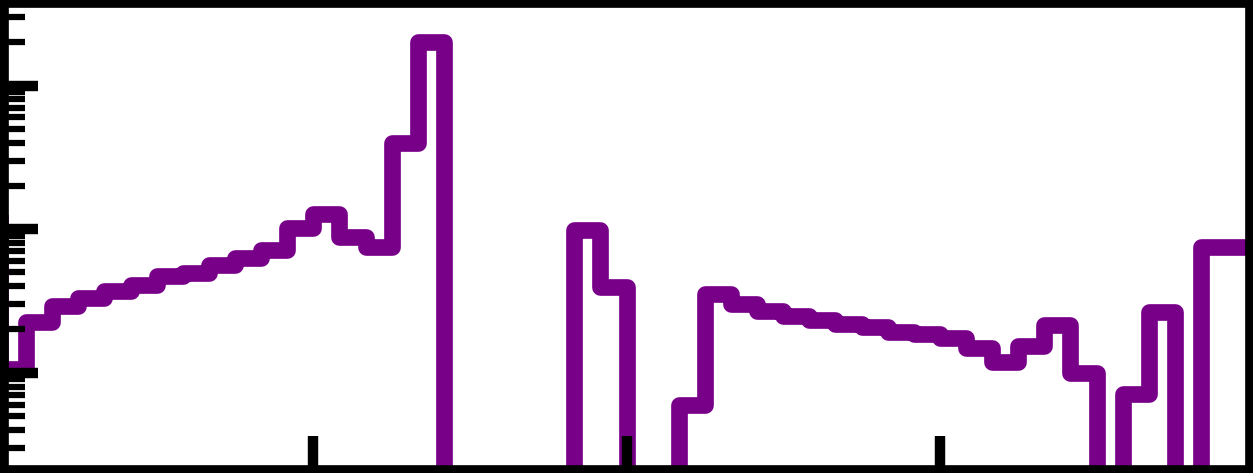

Supplement: Supplementary file 10 — Dataset EV2 [file MSB-13-926-s010.zip › dataset_ev2_ccasr_data_and_analysis/ccasr_analysis/plots/dv-mono-uv_logy_full_intlog.png]

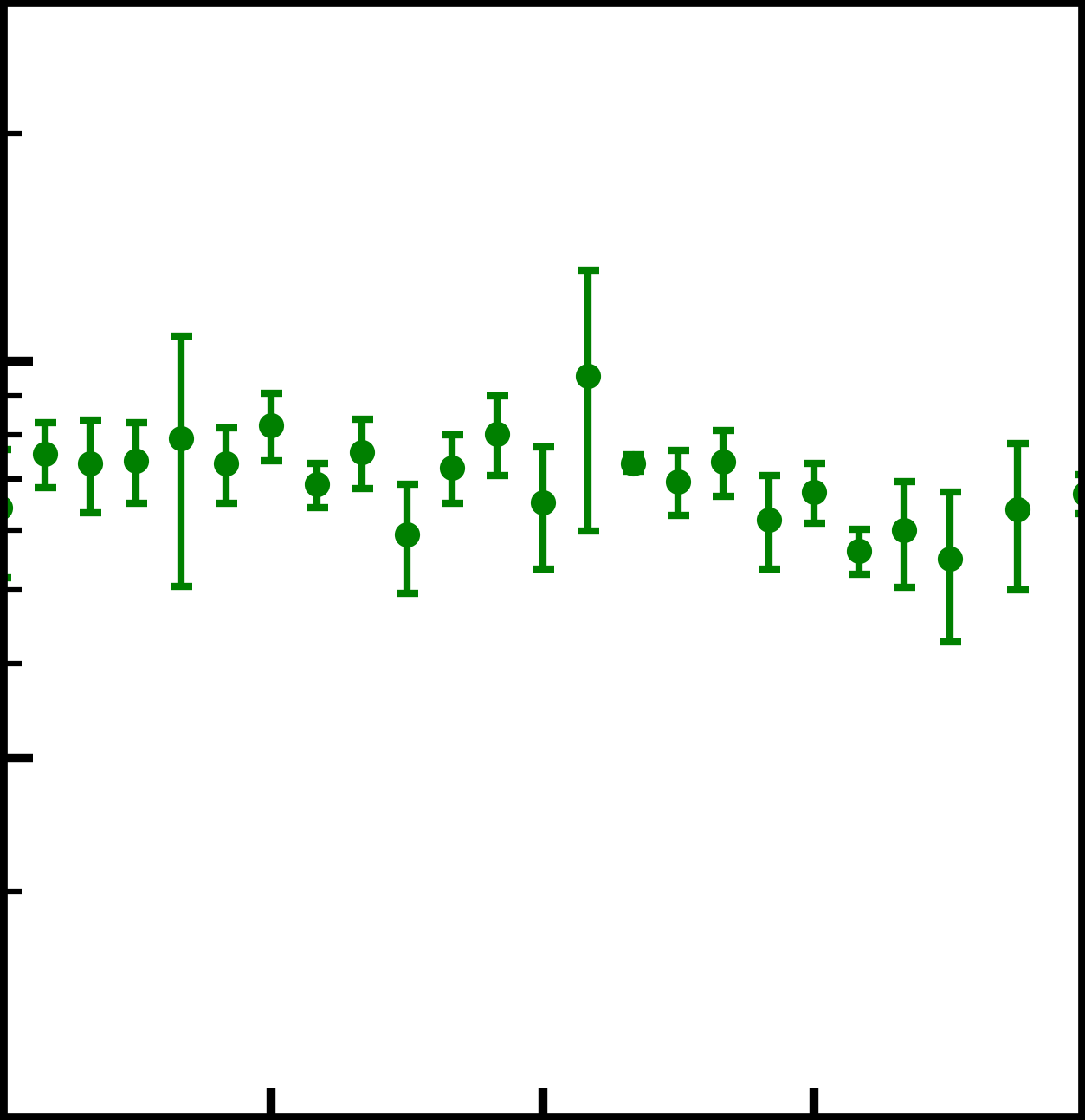

Supplement: Supplementary file 10 — Dataset EV2 [file MSB-13-926-s010.zip › dataset_ev2_ccasr_data_and_analysis/ccasr_analysis/plots/dv-mono-uv_logy_full_od_data.png]

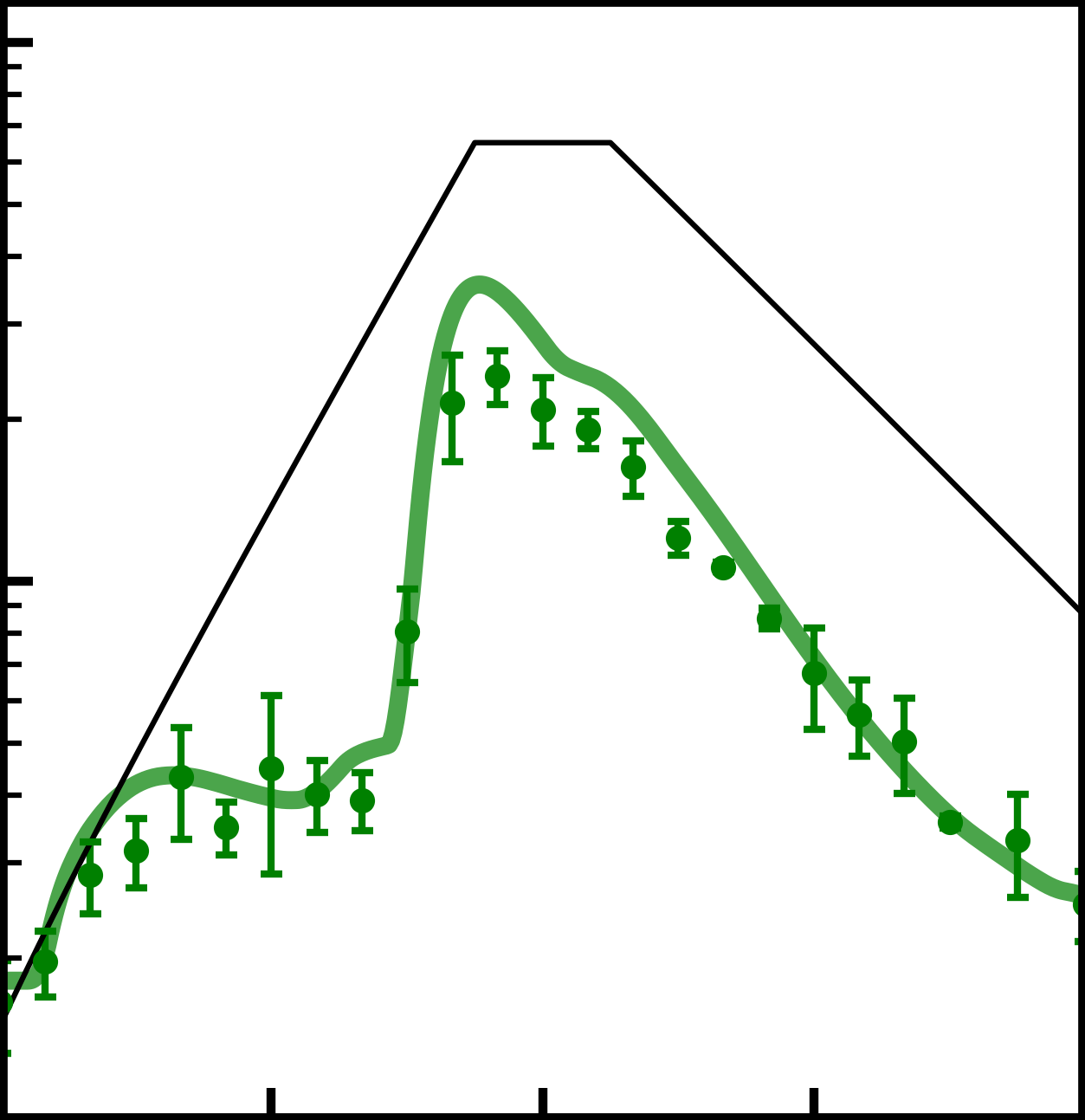

Supplement: Supplementary file 10 — Dataset EV2 [file MSB-13-926-s010.zip › dataset_ev2_ccasr_data_and_analysis/ccasr_analysis/plots/dv-pert_logy_full_data.png]

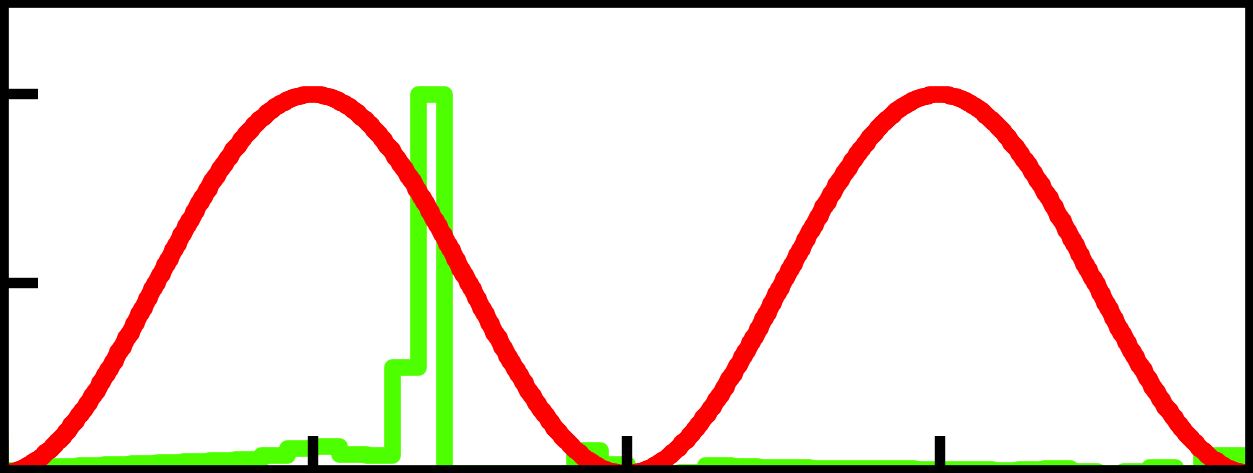

Supplement: Supplementary file 10 — Dataset EV2 [file MSB-13-926-s010.zip › dataset_ev2_ccasr_data_and_analysis/ccasr_analysis/plots/dv-pert_logy_full_intlin.png]

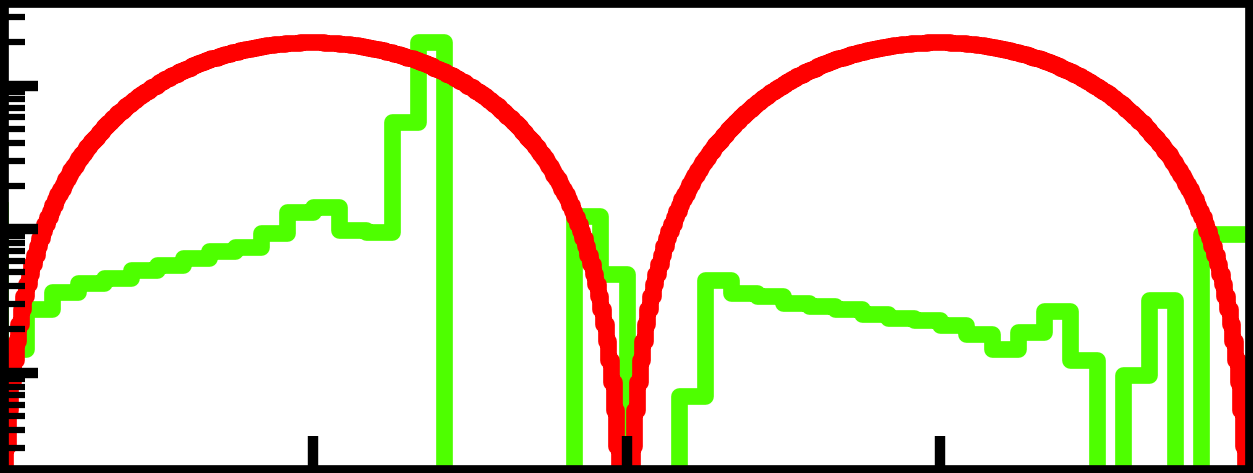

Supplement: Supplementary file 10 — Dataset EV2 [file MSB-13-926-s010.zip › dataset_ev2_ccasr_data_and_analysis/ccasr_analysis/plots/dv-pert_logy_full_intlog.png]

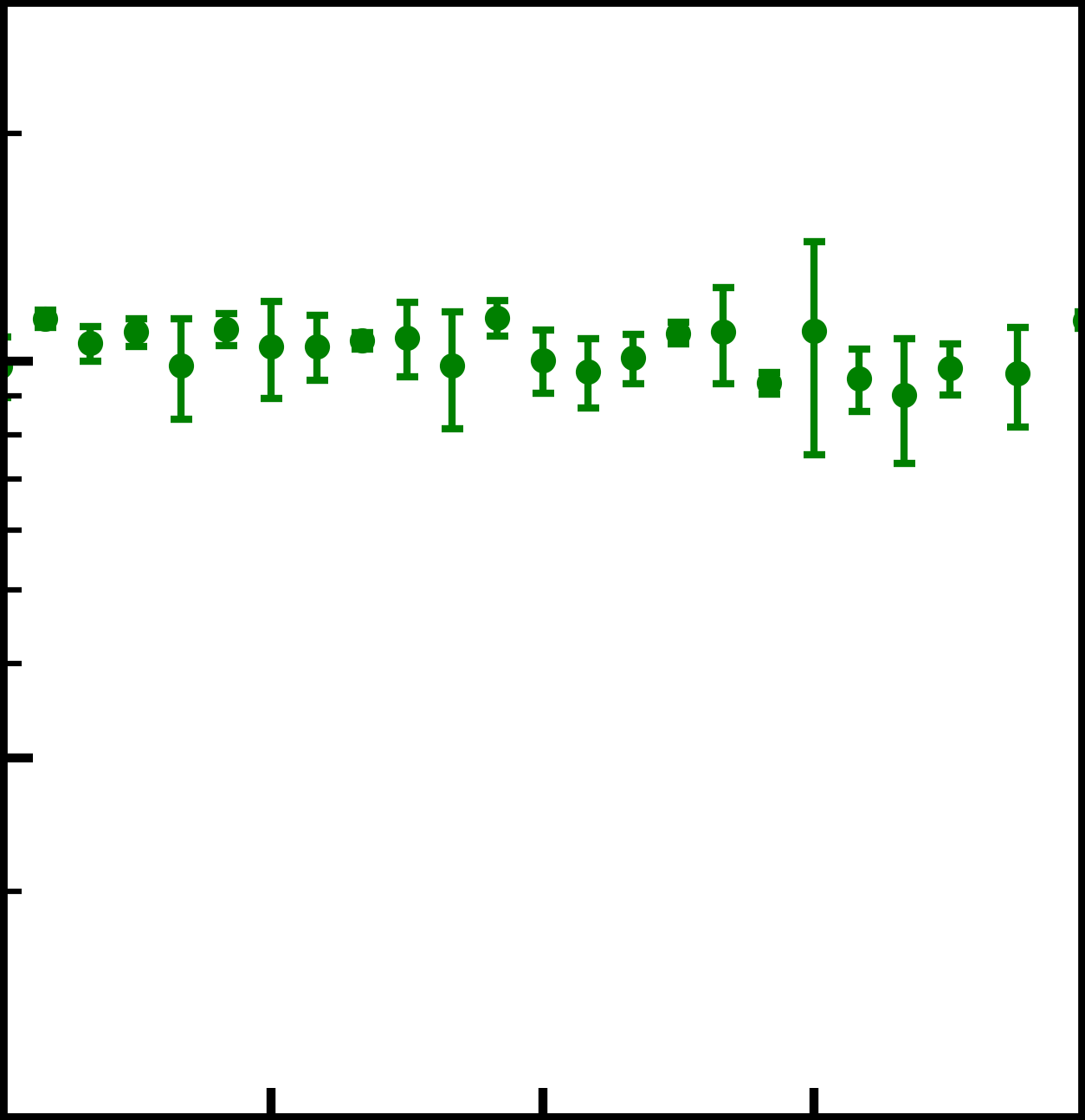

Supplement: Supplementary file 10 — Dataset EV2 [file MSB-13-926-s010.zip › dataset_ev2_ccasr_data_and_analysis/ccasr_analysis/plots/dv-pert_logy_full_od_data.png]

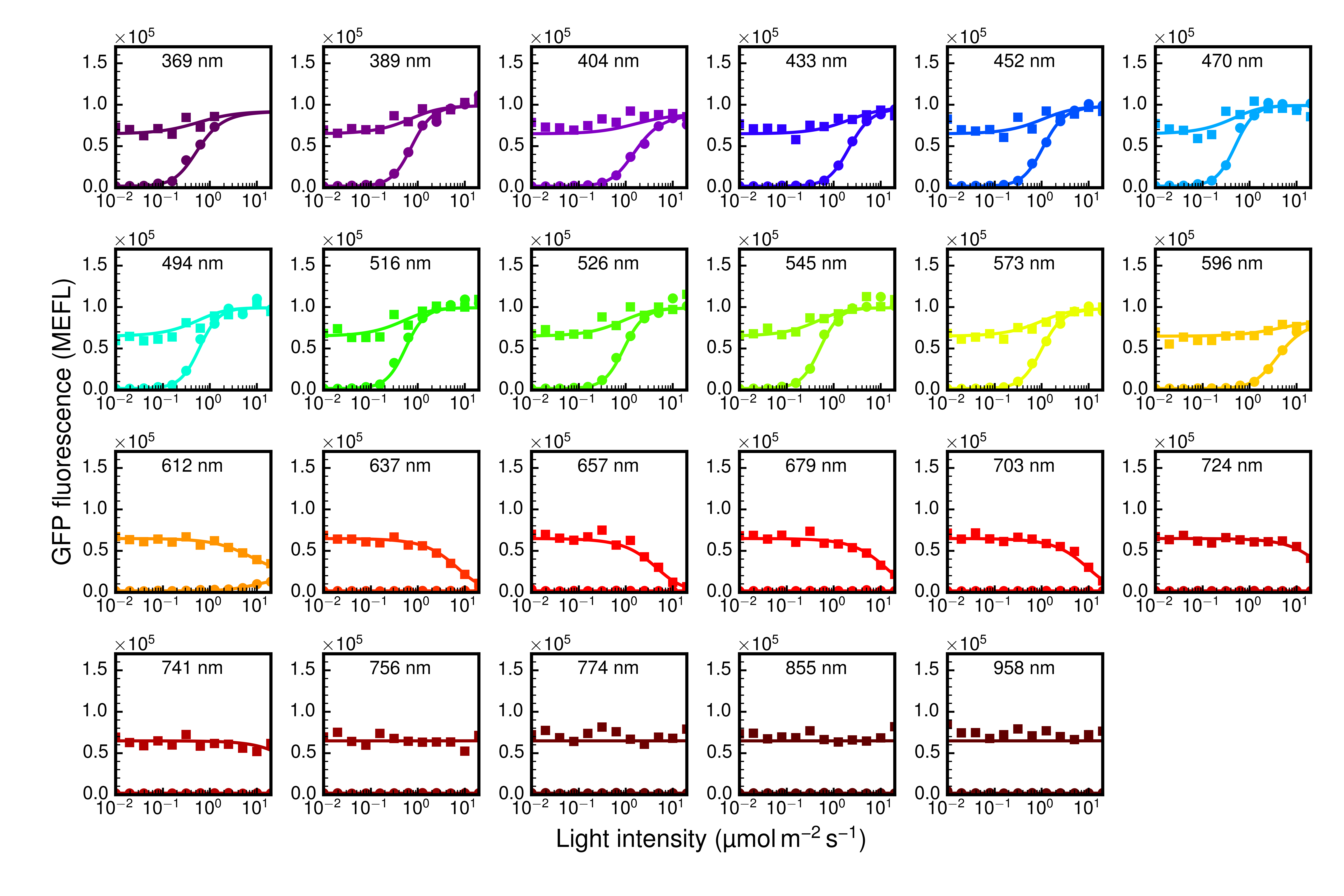

Supplement: Supplementary file 10 — Dataset EV2 [file MSB-13-926-s010.zip › dataset_ev2_ccasr_data_and_analysis/ccasr_analysis/plots/led_fit_lin.png]

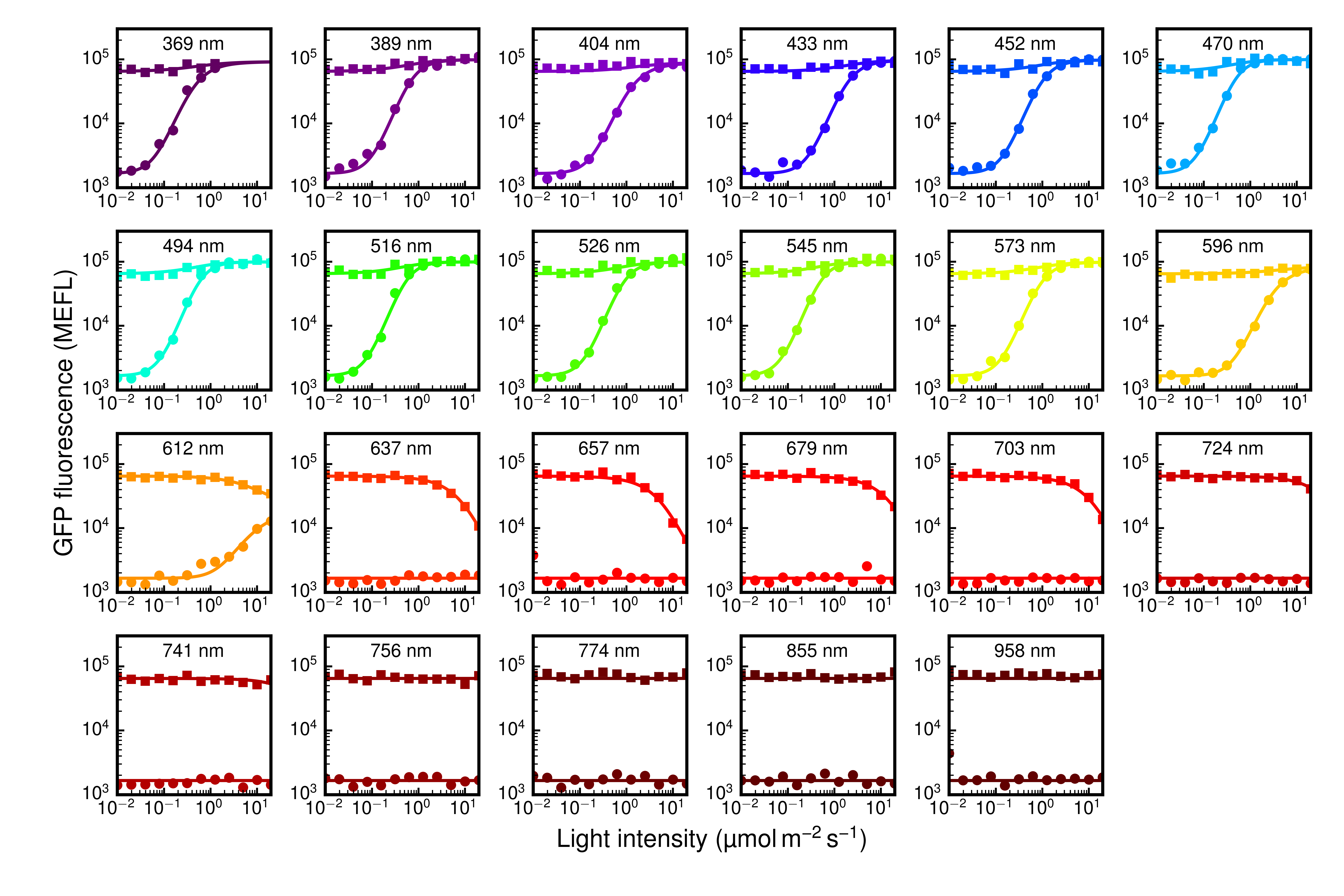

Supplement: Supplementary file 10 — Dataset EV2 [file MSB-13-926-s010.zip › dataset_ev2_ccasr_data_and_analysis/ccasr_analysis/plots/led_fit_log.png]

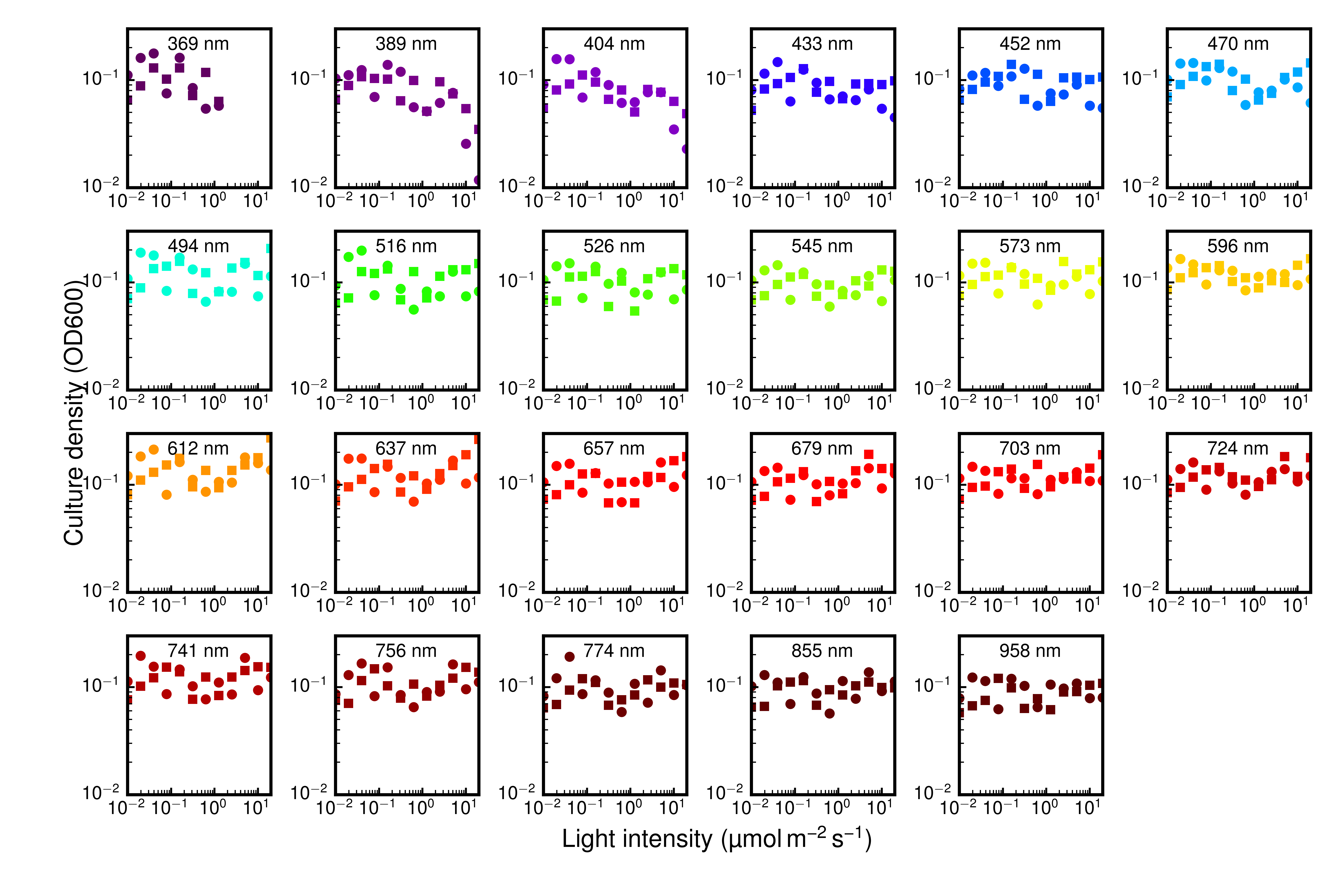

Supplement: Supplementary file 10 — Dataset EV2 [file MSB-13-926-s010.zip › dataset_ev2_ccasr_data_and_analysis/ccasr_analysis/plots/led_od_log.png]

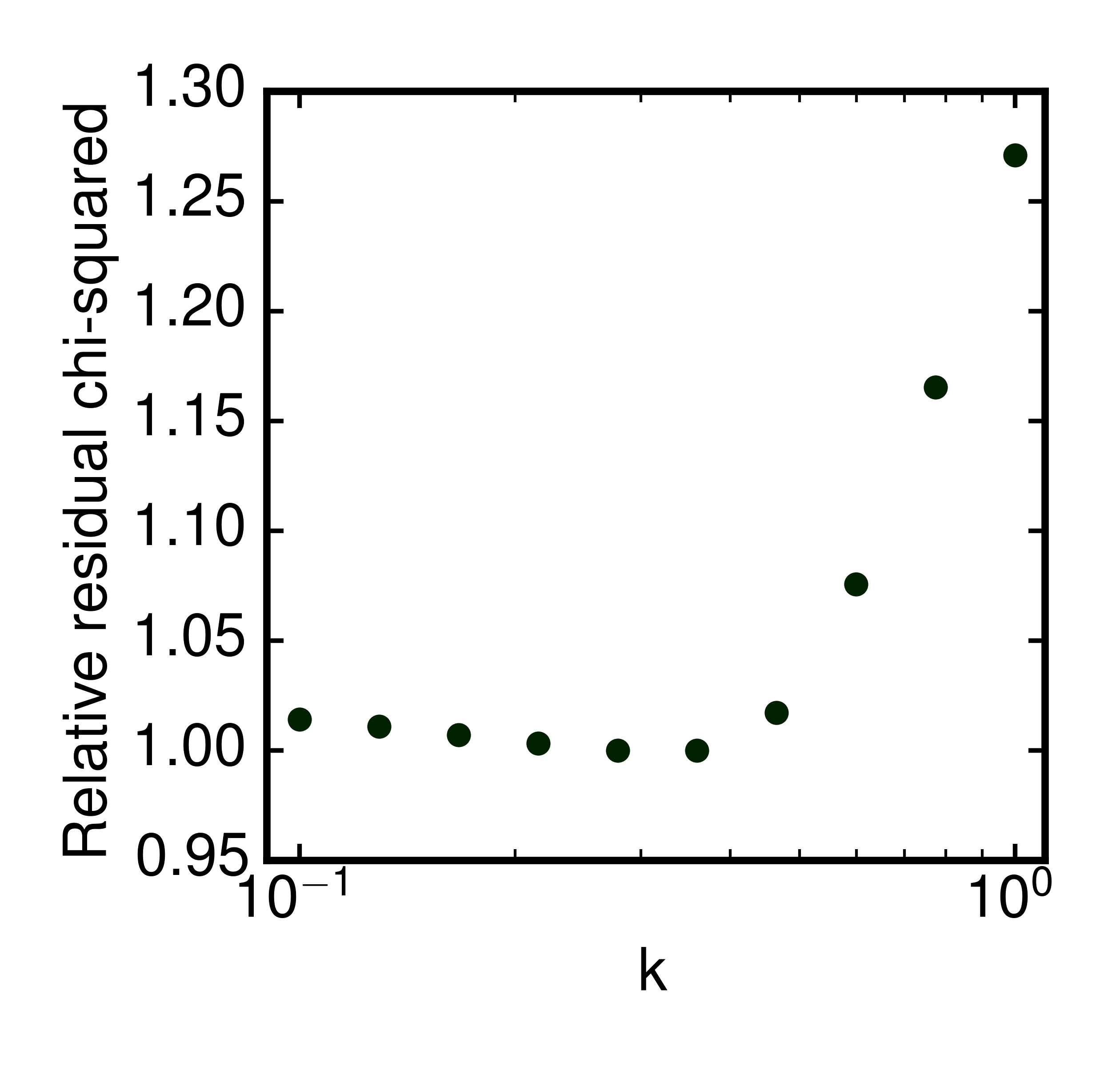

Supplement: Supplementary file 10 — Dataset EV2 [file MSB-13-926-s010.zip › dataset_ev2_ccasr_data_and_analysis/ccasr_analysis/plots/multicollinearity_chisq.png]

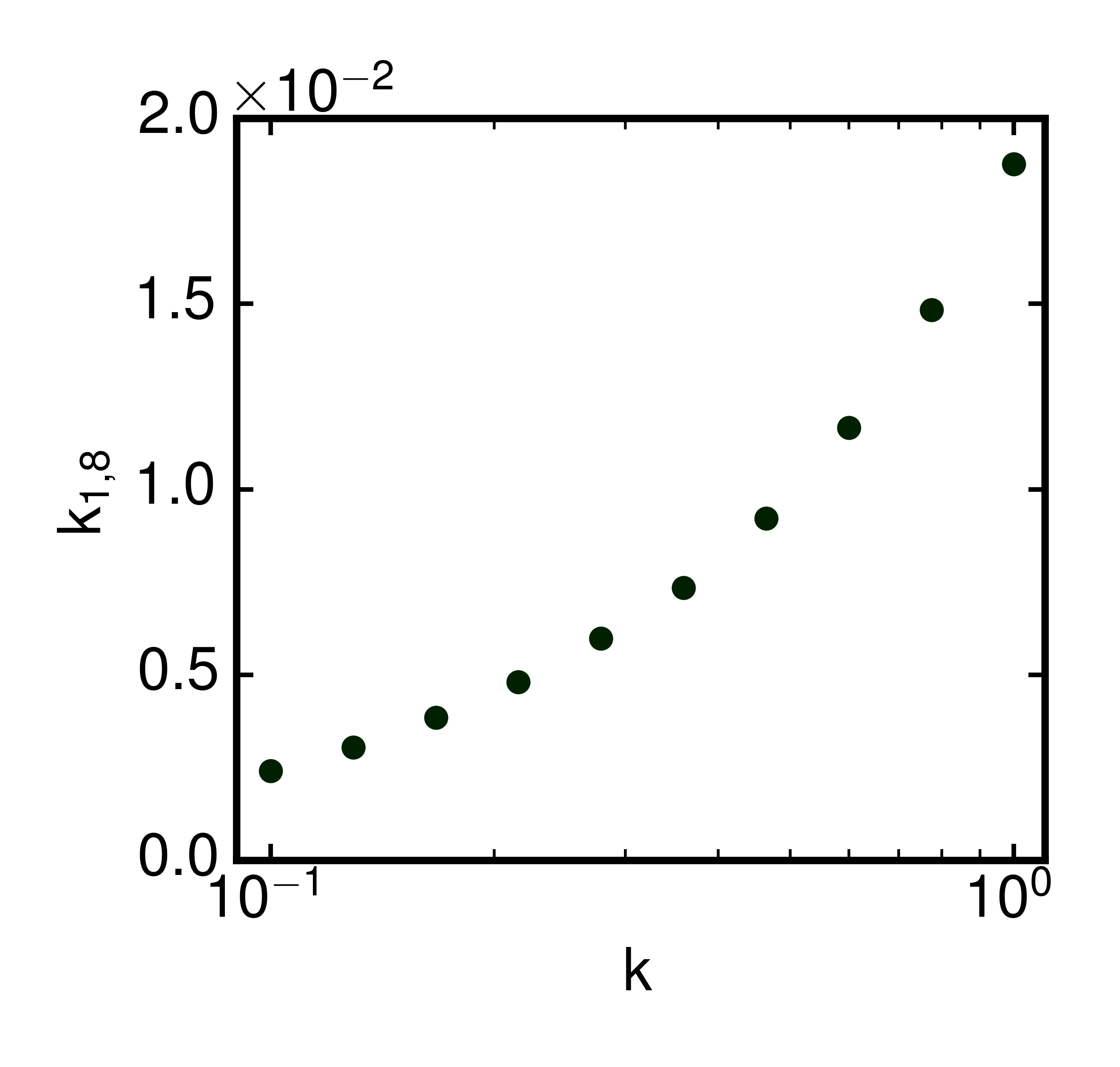

Supplement: Supplementary file 10 — Dataset EV2 [file MSB-13-926-s010.zip › dataset_ev2_ccasr_data_and_analysis/ccasr_analysis/plots/multicollinearity_k1.png]

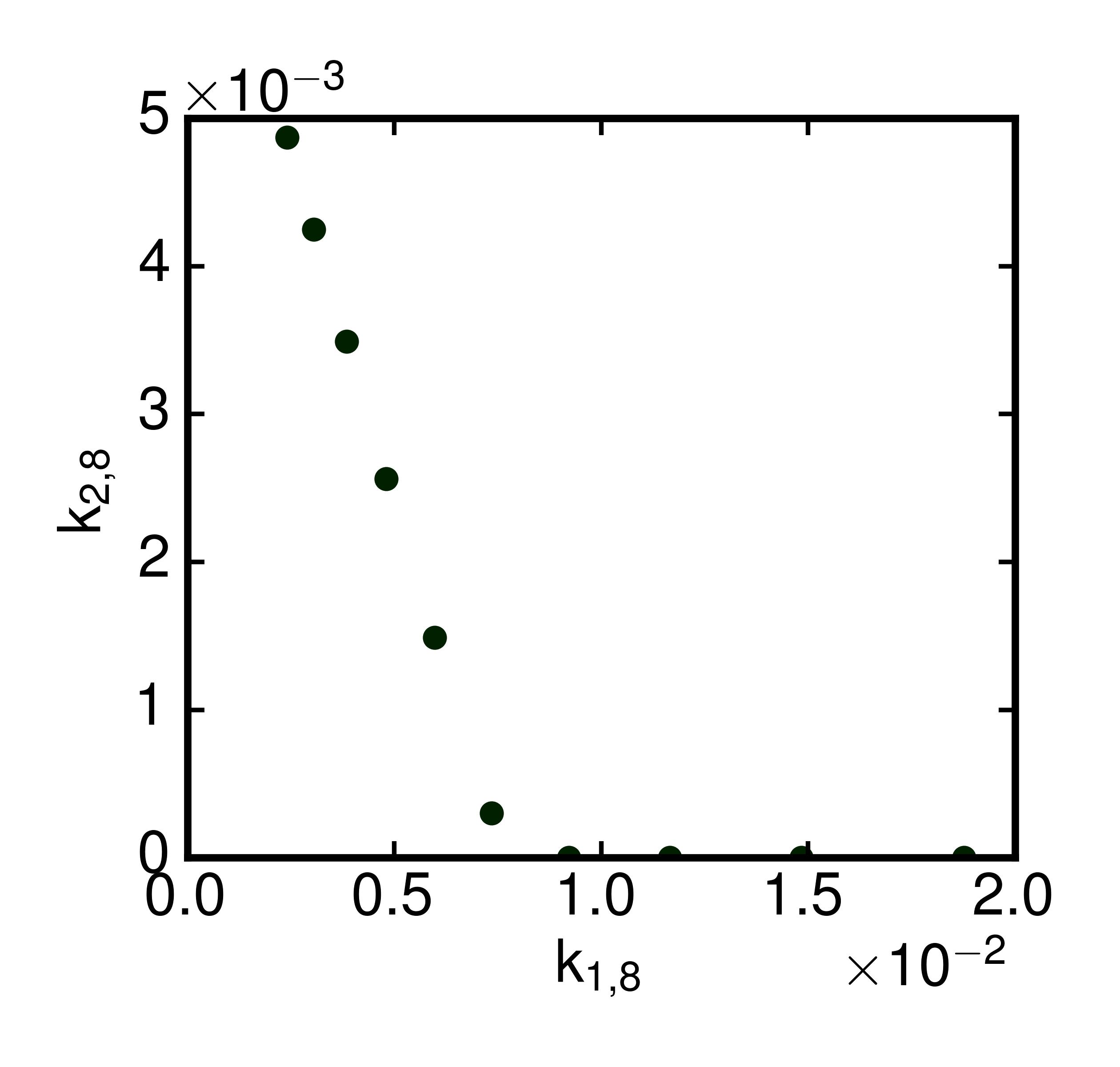

Supplement: Supplementary file 10 — Dataset EV2 [file MSB-13-926-s010.zip › dataset_ev2_ccasr_data_and_analysis/ccasr_analysis/plots/multicollinearity_k1k2.png]

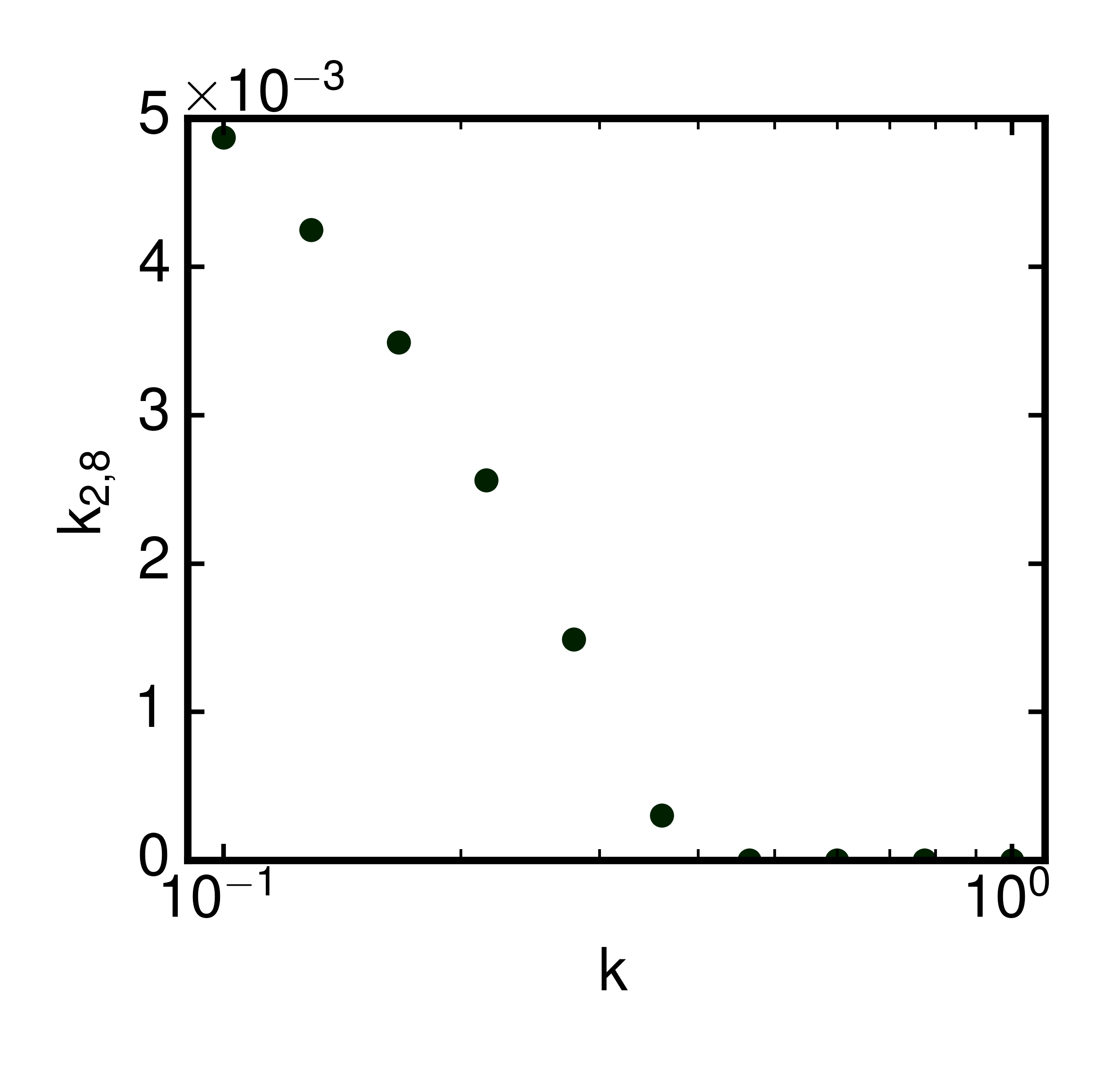

Supplement: Supplementary file 10 — Dataset EV2 [file MSB-13-926-s010.zip › dataset_ev2_ccasr_data_and_analysis/ccasr_analysis/plots/multicollinearity_k2.png]

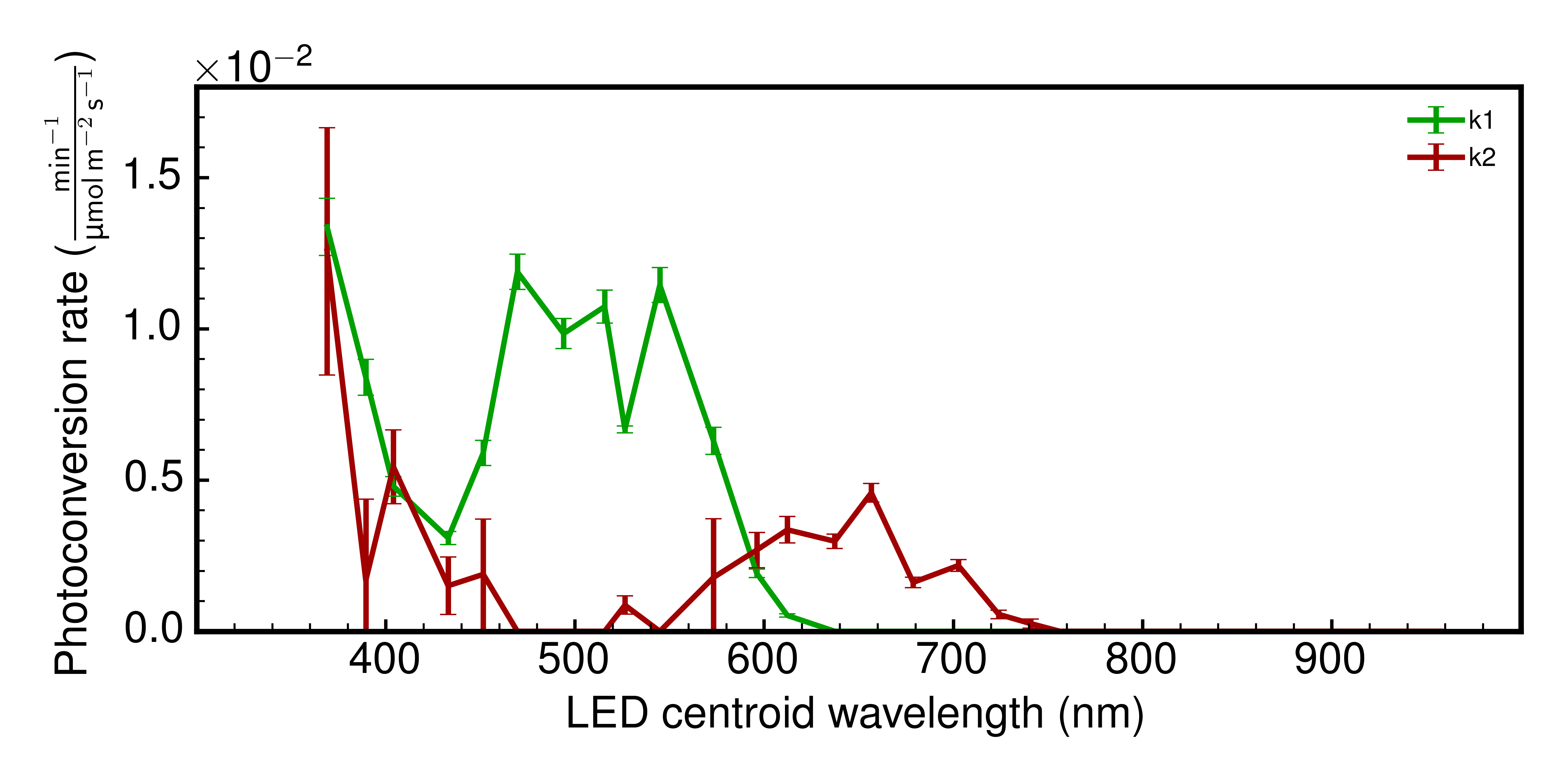

Supplement: Supplementary file 10 — Dataset EV2 [file MSB-13-926-s010.zip › dataset_ev2_ccasr_data_and_analysis/ccasr_analysis/plots/pcs.png]

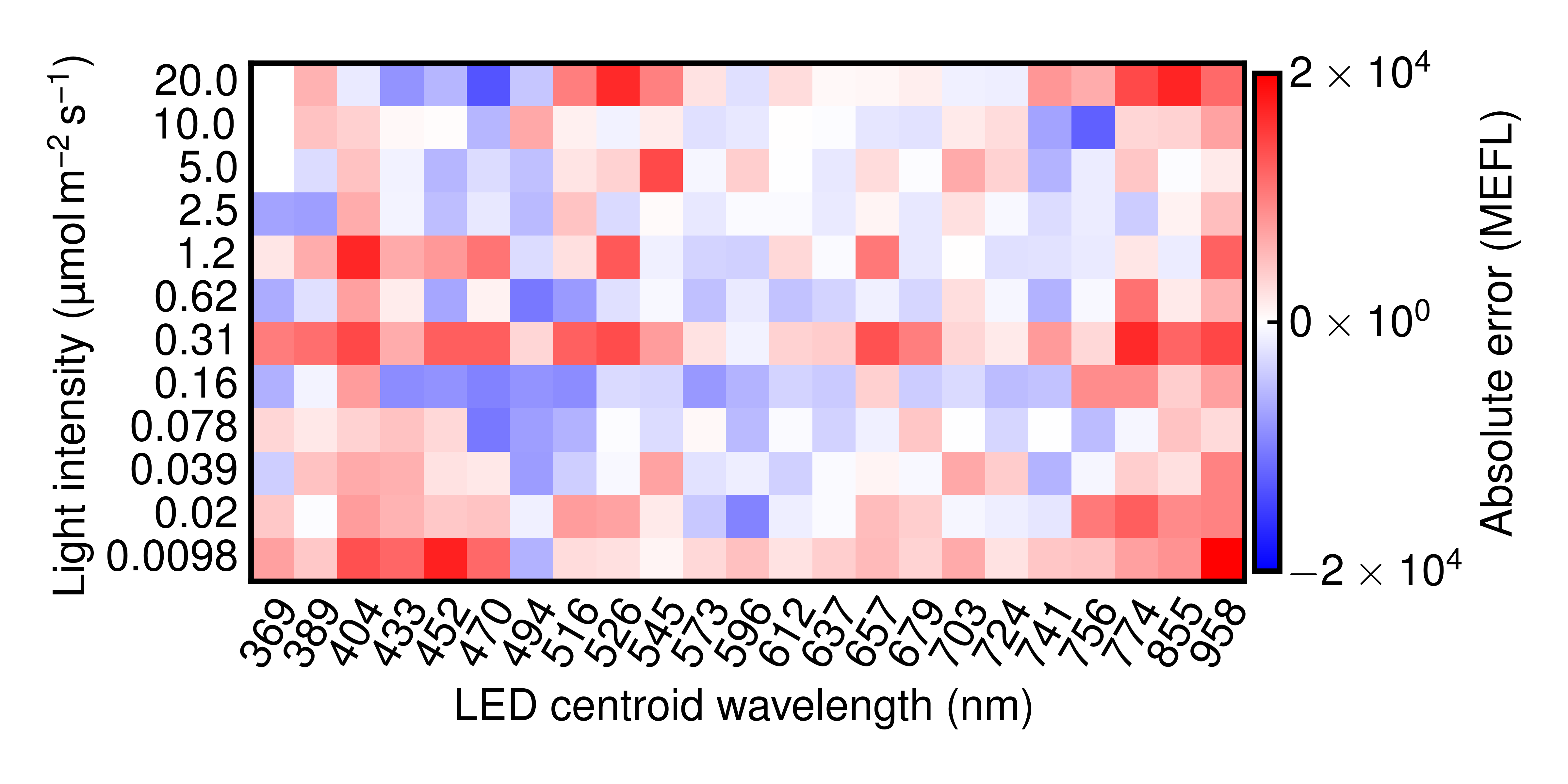

Supplement: Supplementary file 10 — Dataset EV2 [file MSB-13-926-s010.zip › dataset_ev2_ccasr_data_and_analysis/ccasr_analysis/plots/ras_abs_residual_heatmap.png]

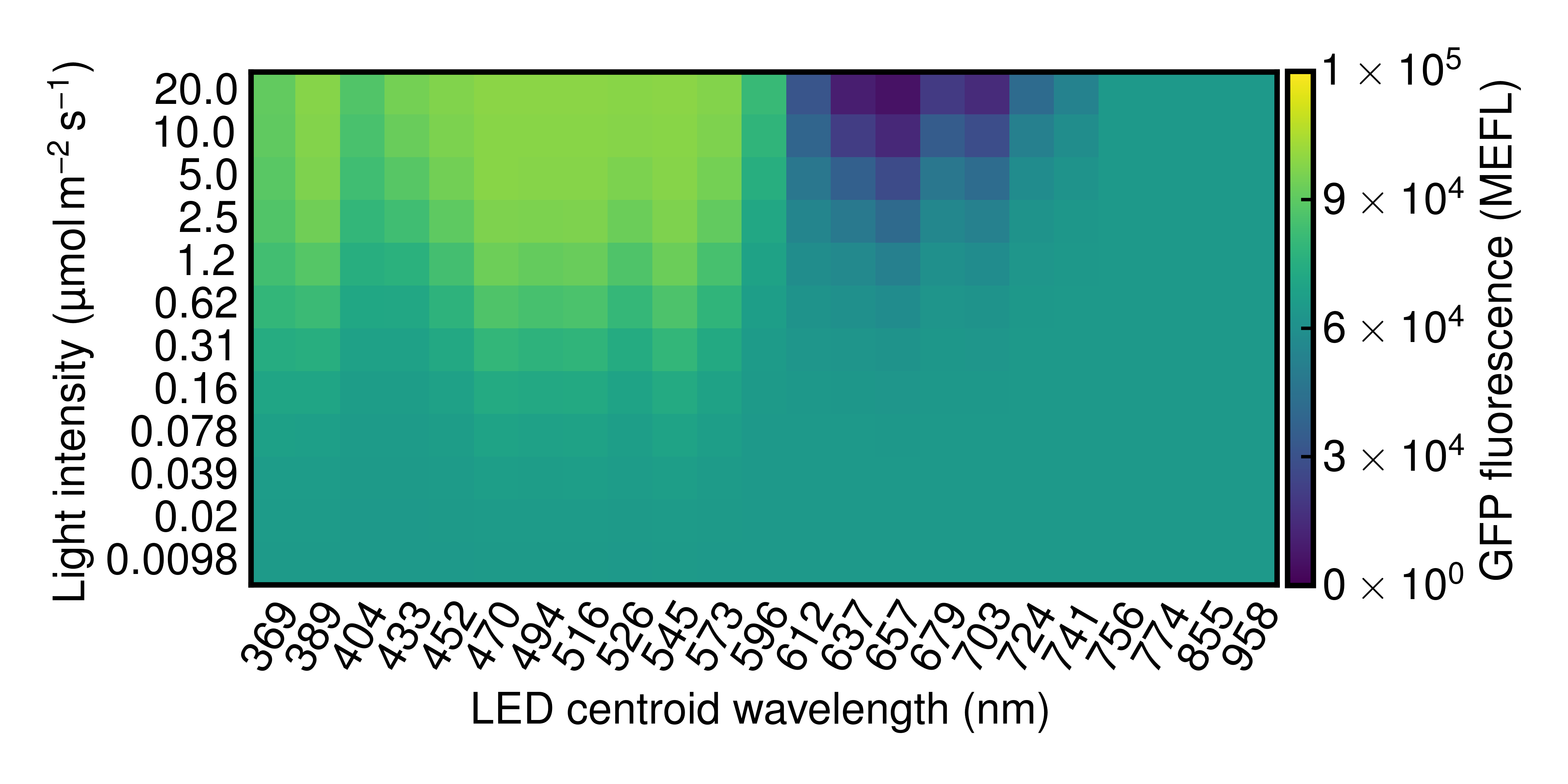

Supplement: Supplementary file 10 — Dataset EV2 [file MSB-13-926-s010.zip › dataset_ev2_ccasr_data_and_analysis/ccasr_analysis/plots/ras_lin_model_heatmap.png]

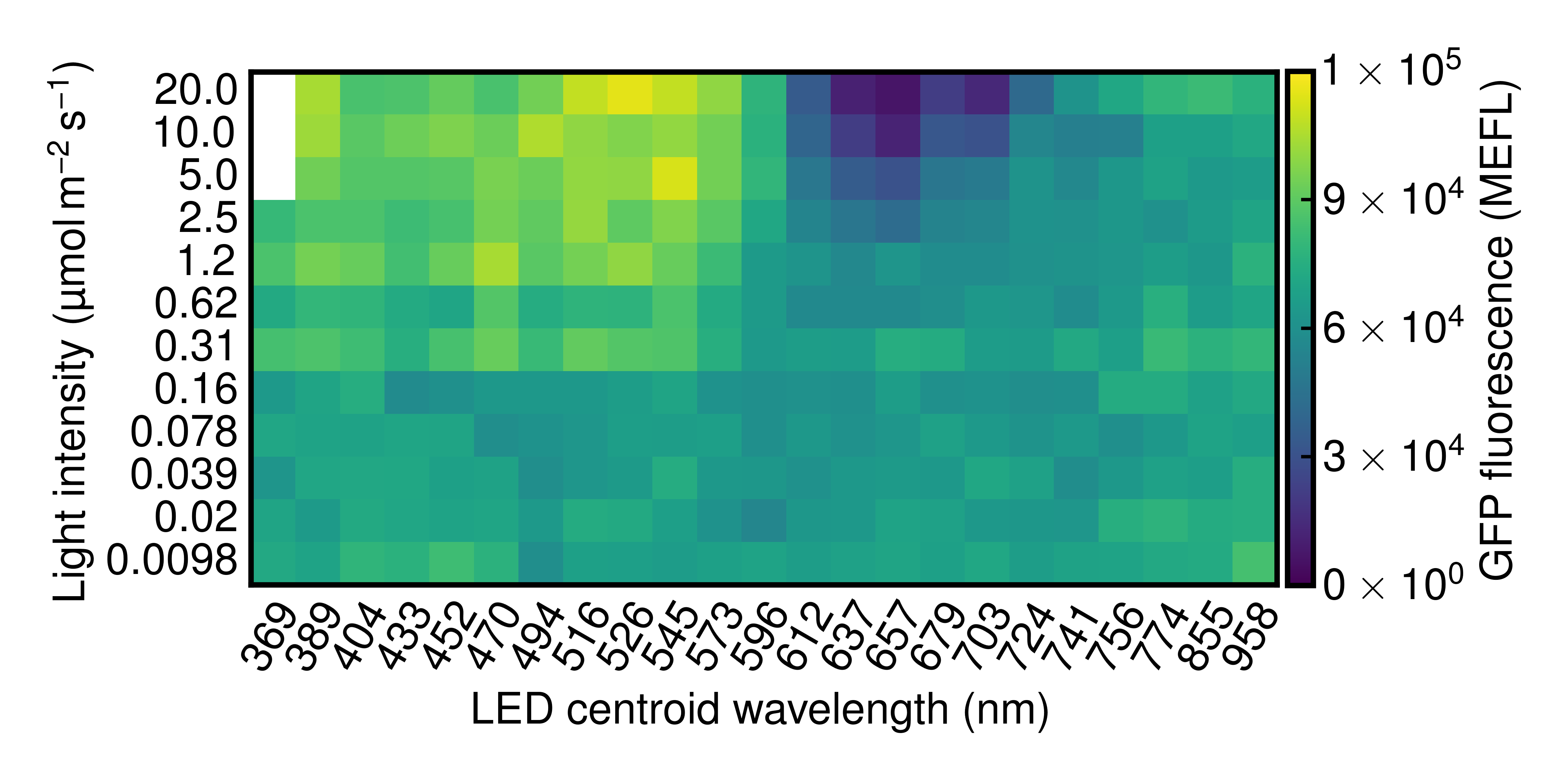

Supplement: Supplementary file 10 — Dataset EV2 [file MSB-13-926-s010.zip › dataset_ev2_ccasr_data_and_analysis/ccasr_analysis/plots/ras_lin_raw_heatmap.png]

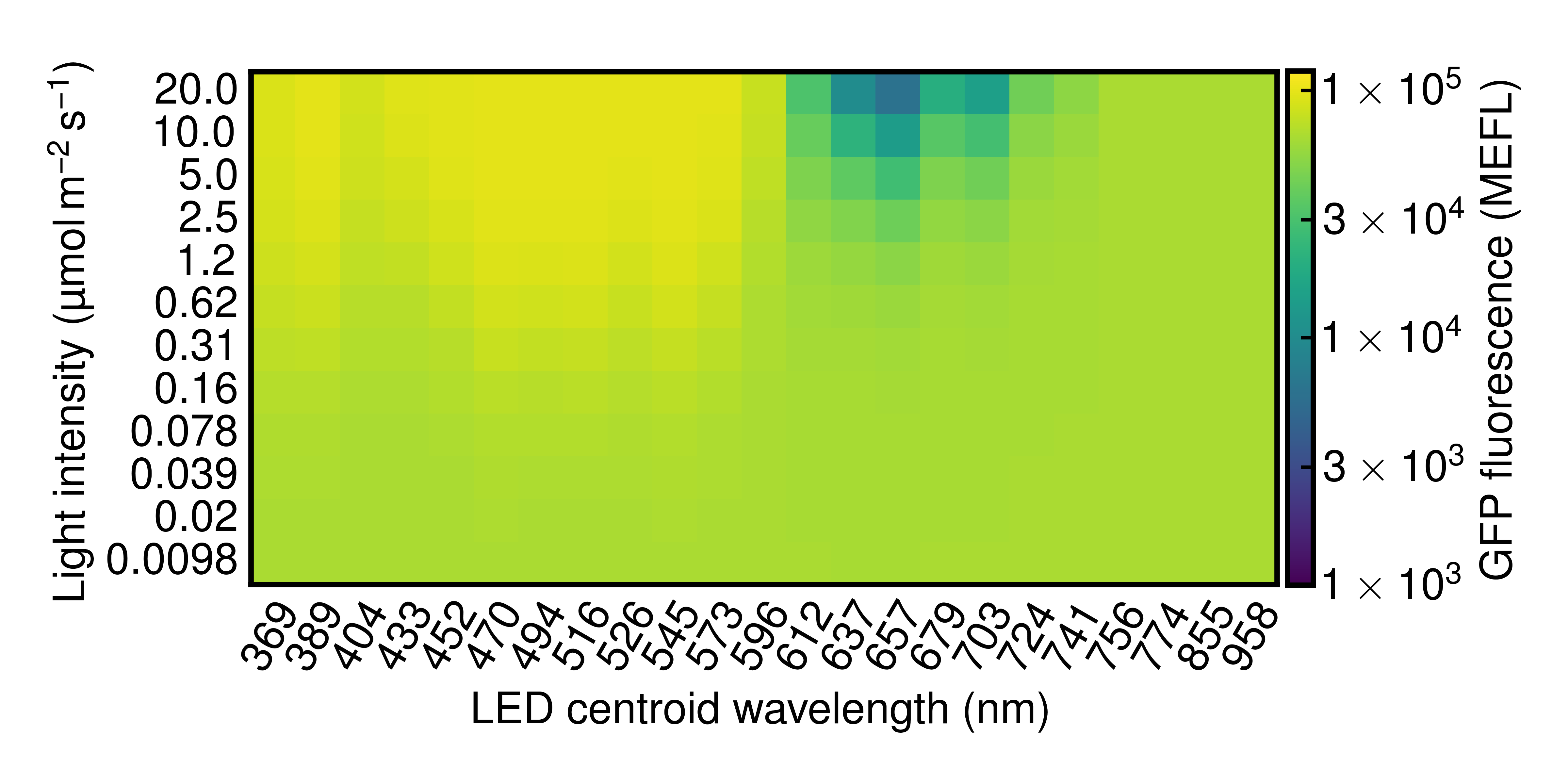

Supplement: Supplementary file 10 — Dataset EV2 [file MSB-13-926-s010.zip › dataset_ev2_ccasr_data_and_analysis/ccasr_analysis/plots/ras_logz_model_heatmap.png]

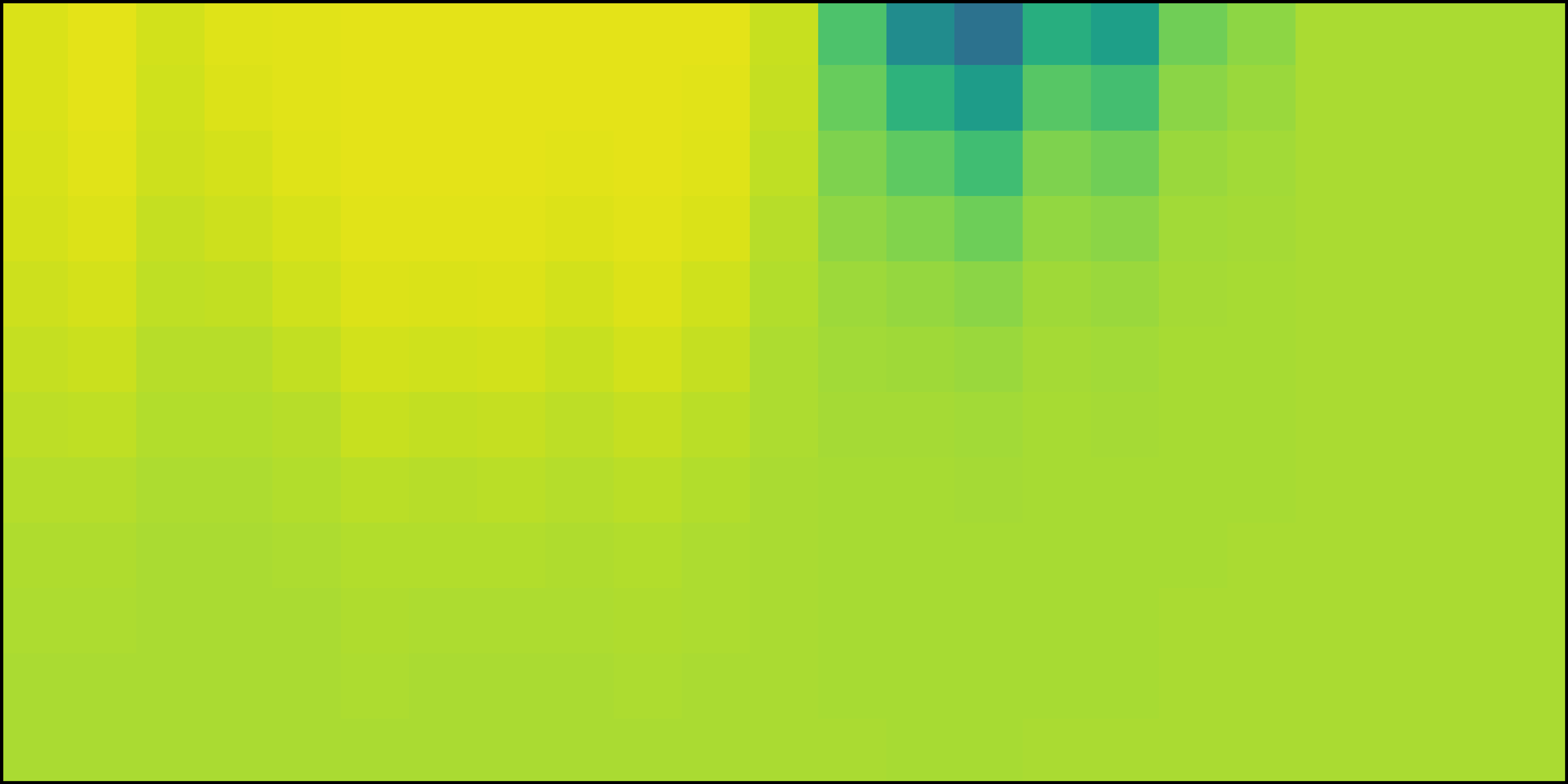

Supplement: Supplementary file 10 — Dataset EV2 [file MSB-13-926-s010.zip › dataset_ev2_ccasr_data_and_analysis/ccasr_analysis/plots/ras_logz_model_nolabel_heatmap.png]

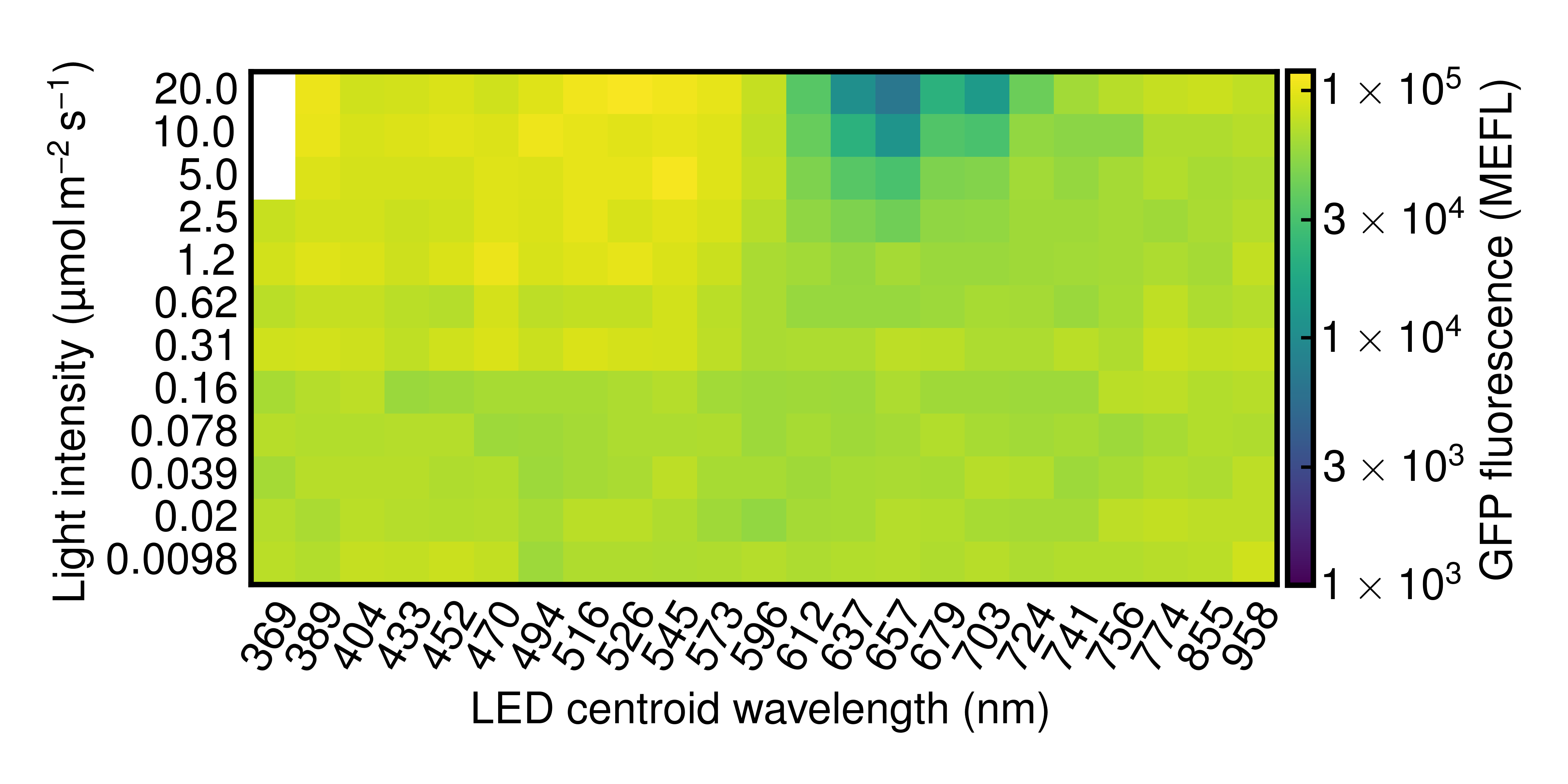

Supplement: Supplementary file 10 — Dataset EV2 [file MSB-13-926-s010.zip › dataset_ev2_ccasr_data_and_analysis/ccasr_analysis/plots/ras_logz_raw_heatmap.png]

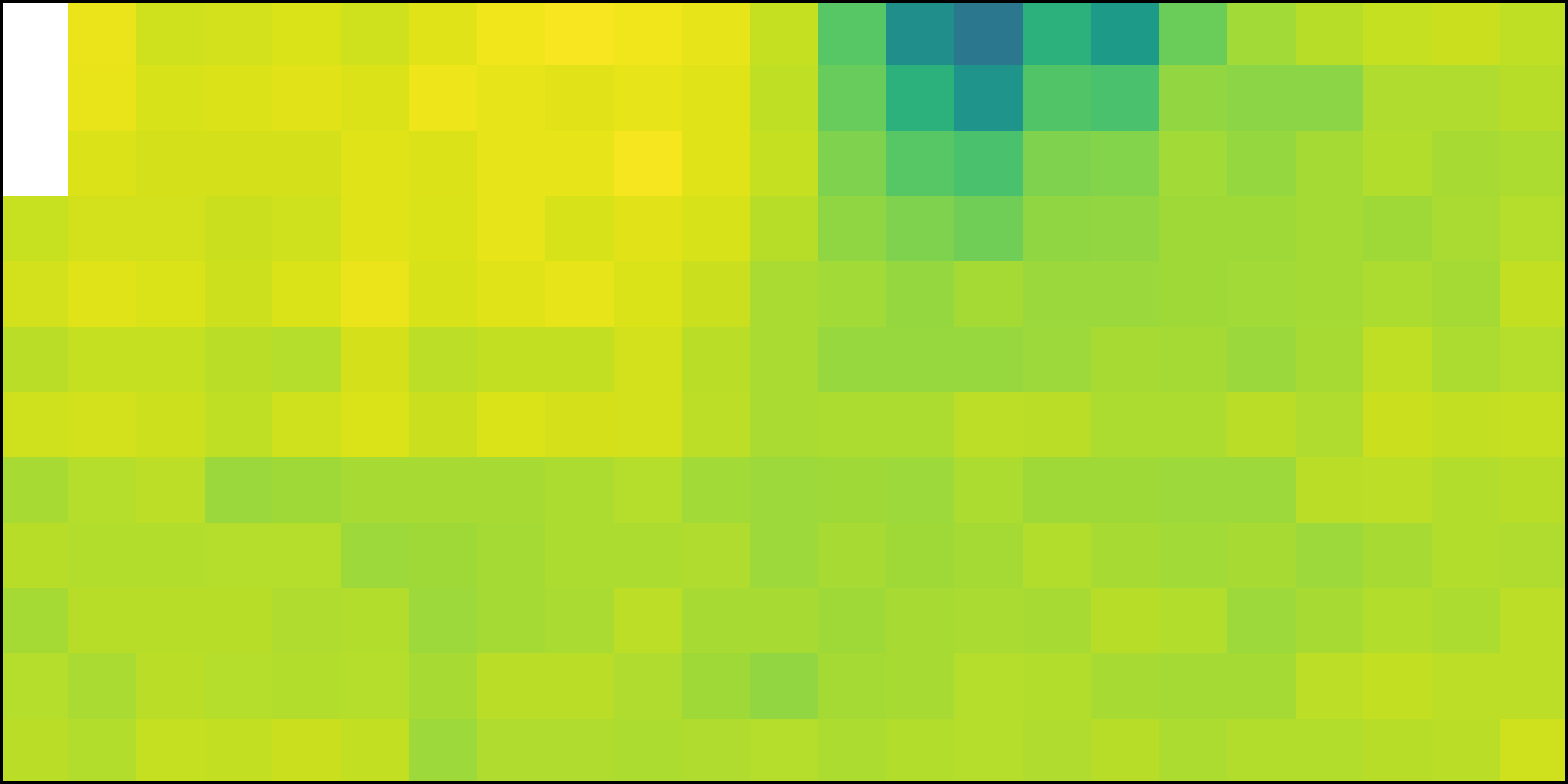

Supplement: Supplementary file 10 — Dataset EV2 [file MSB-13-926-s010.zip › dataset_ev2_ccasr_data_and_analysis/ccasr_analysis/plots/ras_logz_raw_nolabel_heatmap.png]

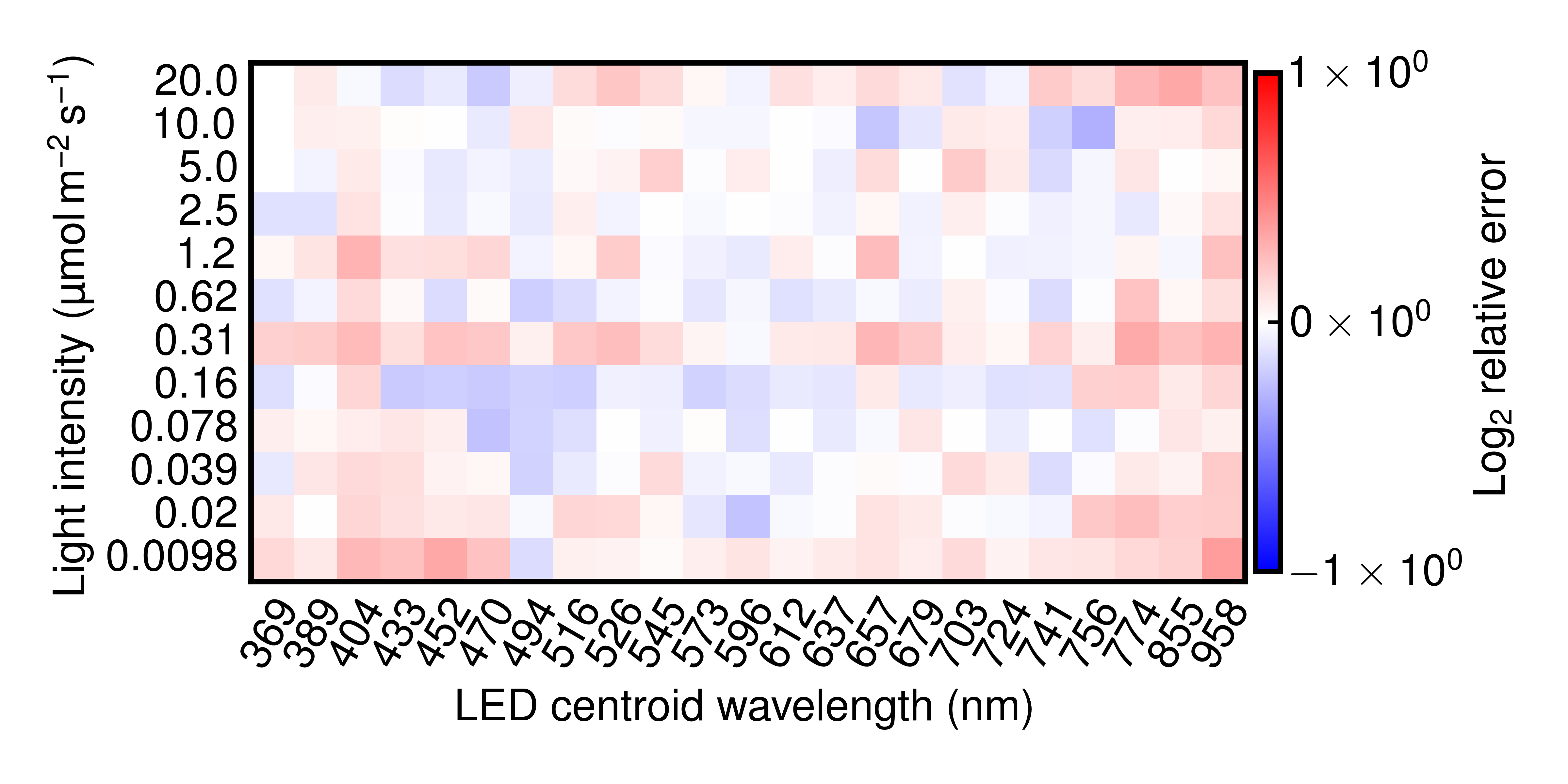

Supplement: Supplementary file 10 — Dataset EV2 [file MSB-13-926-s010.zip › dataset_ev2_ccasr_data_and_analysis/ccasr_analysis/plots/ras_rel_residual_heatmap.png]

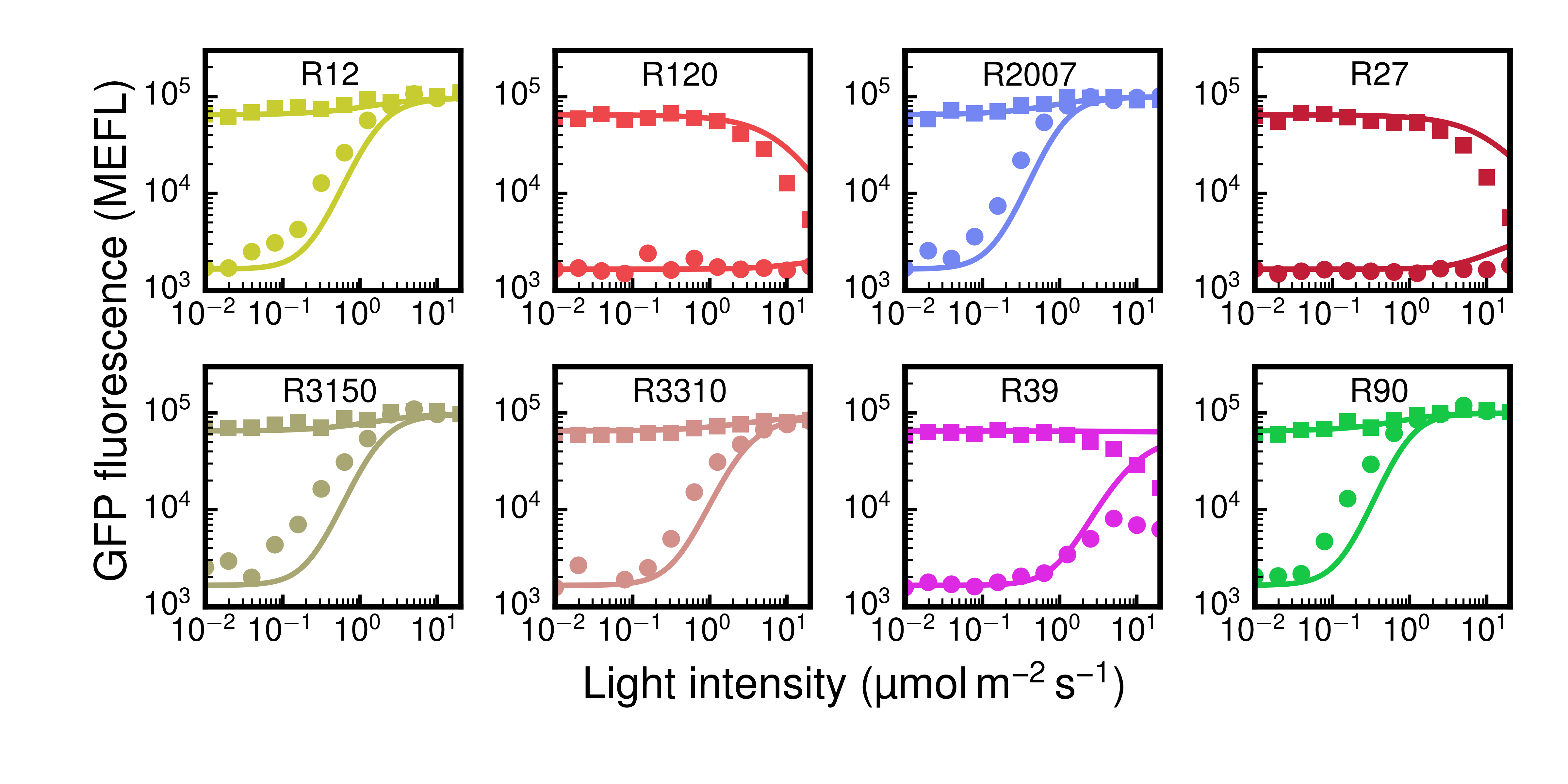

Supplement: Supplementary file 10 — Dataset EV2 [file MSB-13-926-s010.zip › dataset_ev2_ccasr_data_and_analysis/ccasr_analysis/plots/sv_led_fit_log.png]

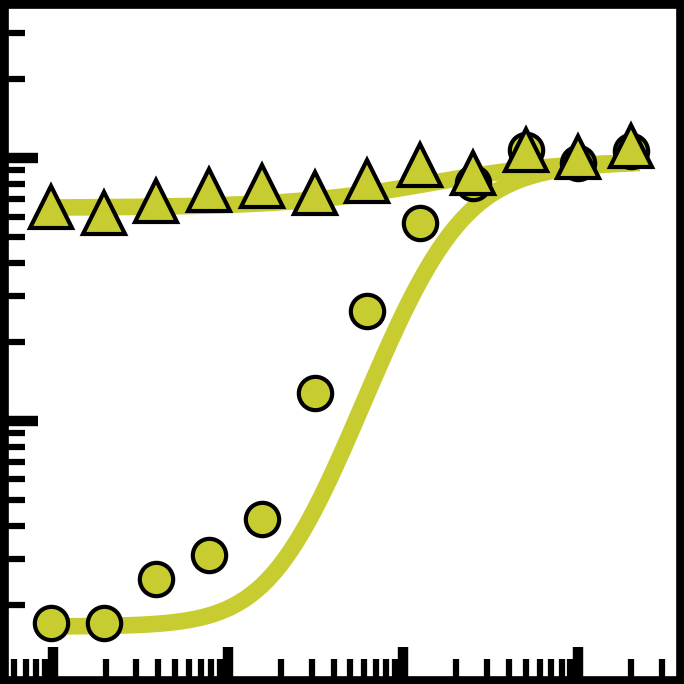

Supplement: Supplementary file 10 — Dataset EV2 [file MSB-13-926-s010.zip › dataset_ev2_ccasr_data_and_analysis/ccasr_analysis/plots/sv_led_fit_R12.png]

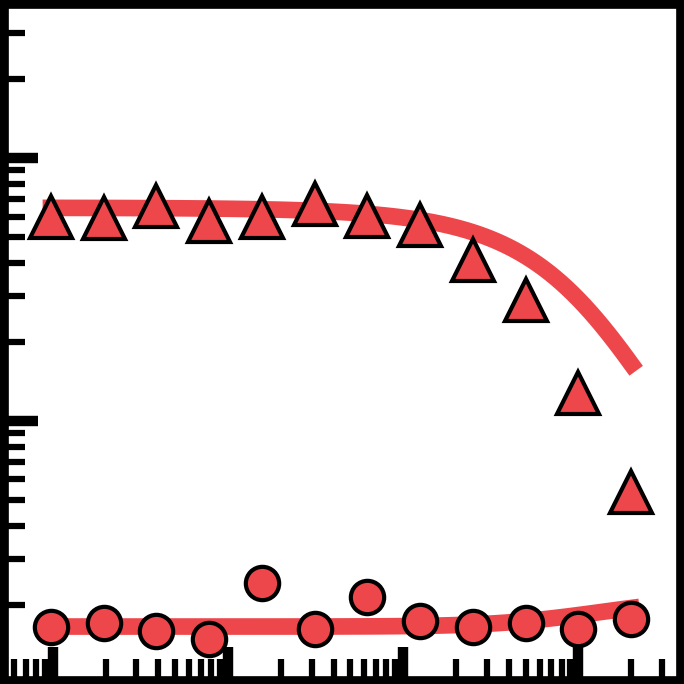

Supplement: Supplementary file 10 — Dataset EV2 [file MSB-13-926-s010.zip › dataset_ev2_ccasr_data_and_analysis/ccasr_analysis/plots/sv_led_fit_R120.png]

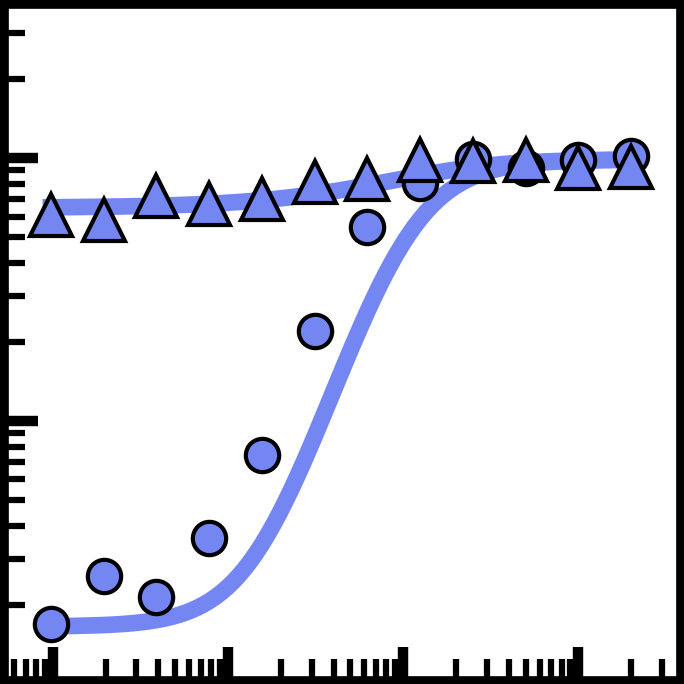

Supplement: Supplementary file 10 — Dataset EV2 [file MSB-13-926-s010.zip › dataset_ev2_ccasr_data_and_analysis/ccasr_analysis/plots/sv_led_fit_R2007.png]

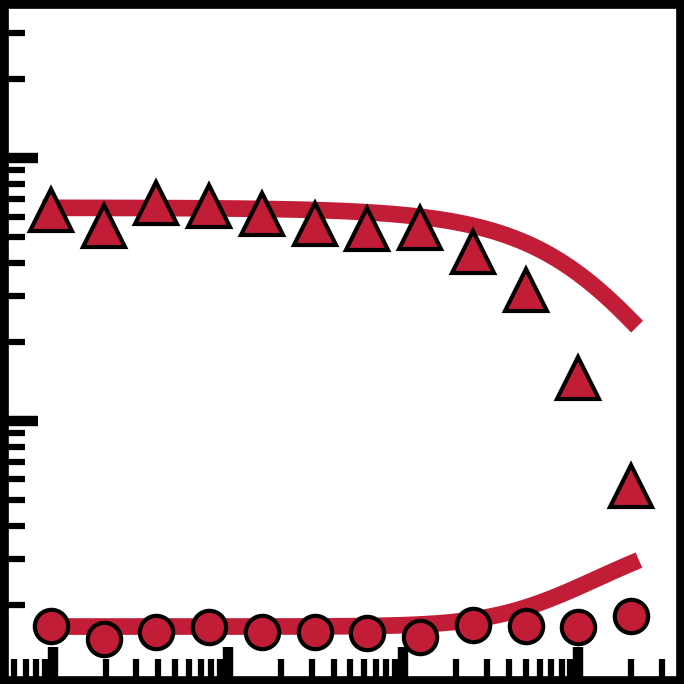

Supplement: Supplementary file 10 — Dataset EV2 [file MSB-13-926-s010.zip › dataset_ev2_ccasr_data_and_analysis/ccasr_analysis/plots/sv_led_fit_R27.png]

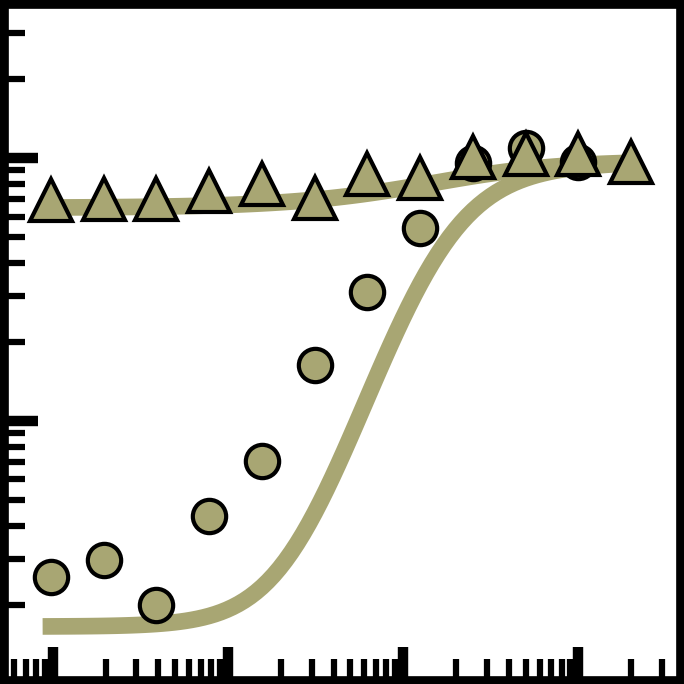

Supplement: Supplementary file 10 — Dataset EV2 [file MSB-13-926-s010.zip › dataset_ev2_ccasr_data_and_analysis/ccasr_analysis/plots/sv_led_fit_R3150.png]

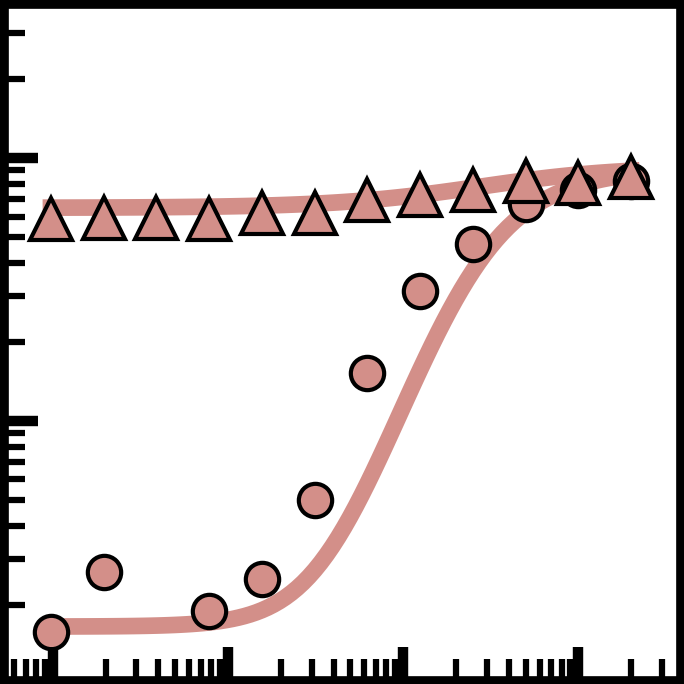

Supplement: Supplementary file 10 — Dataset EV2 [file MSB-13-926-s010.zip › dataset_ev2_ccasr_data_and_analysis/ccasr_analysis/plots/sv_led_fit_R3310.png]

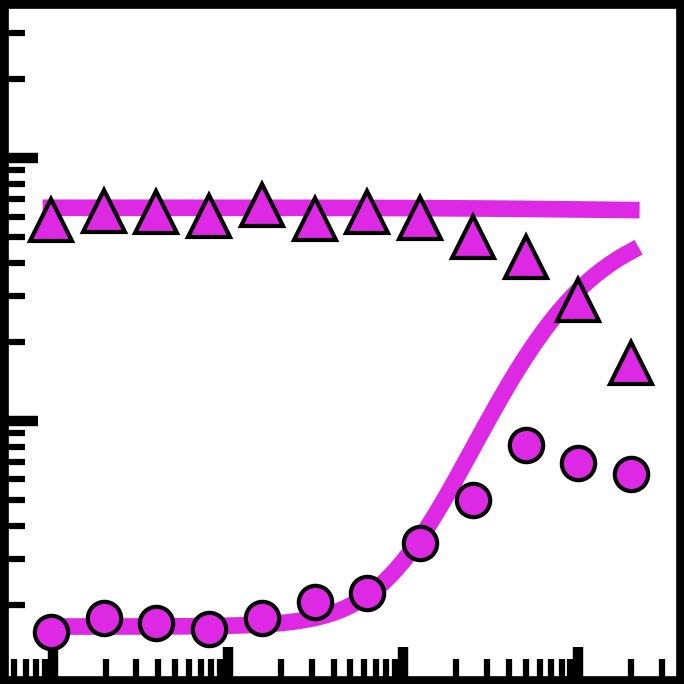

Supplement: Supplementary file 10 — Dataset EV2 [file MSB-13-926-s010.zip › dataset_ev2_ccasr_data_and_analysis/ccasr_analysis/plots/sv_led_fit_R39.png]

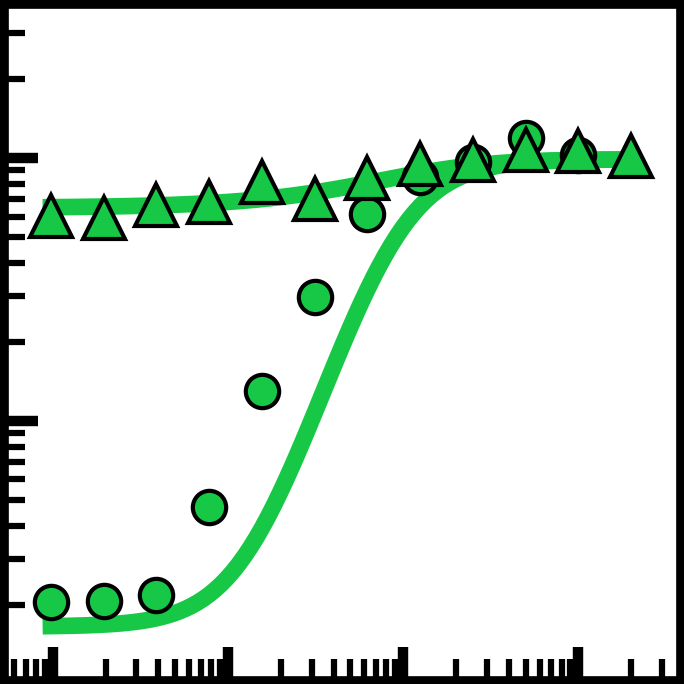

Supplement: Supplementary file 10 — Dataset EV2 [file MSB-13-926-s010.zip › dataset_ev2_ccasr_data_and_analysis/ccasr_analysis/plots/sv_led_fit_R90.png]

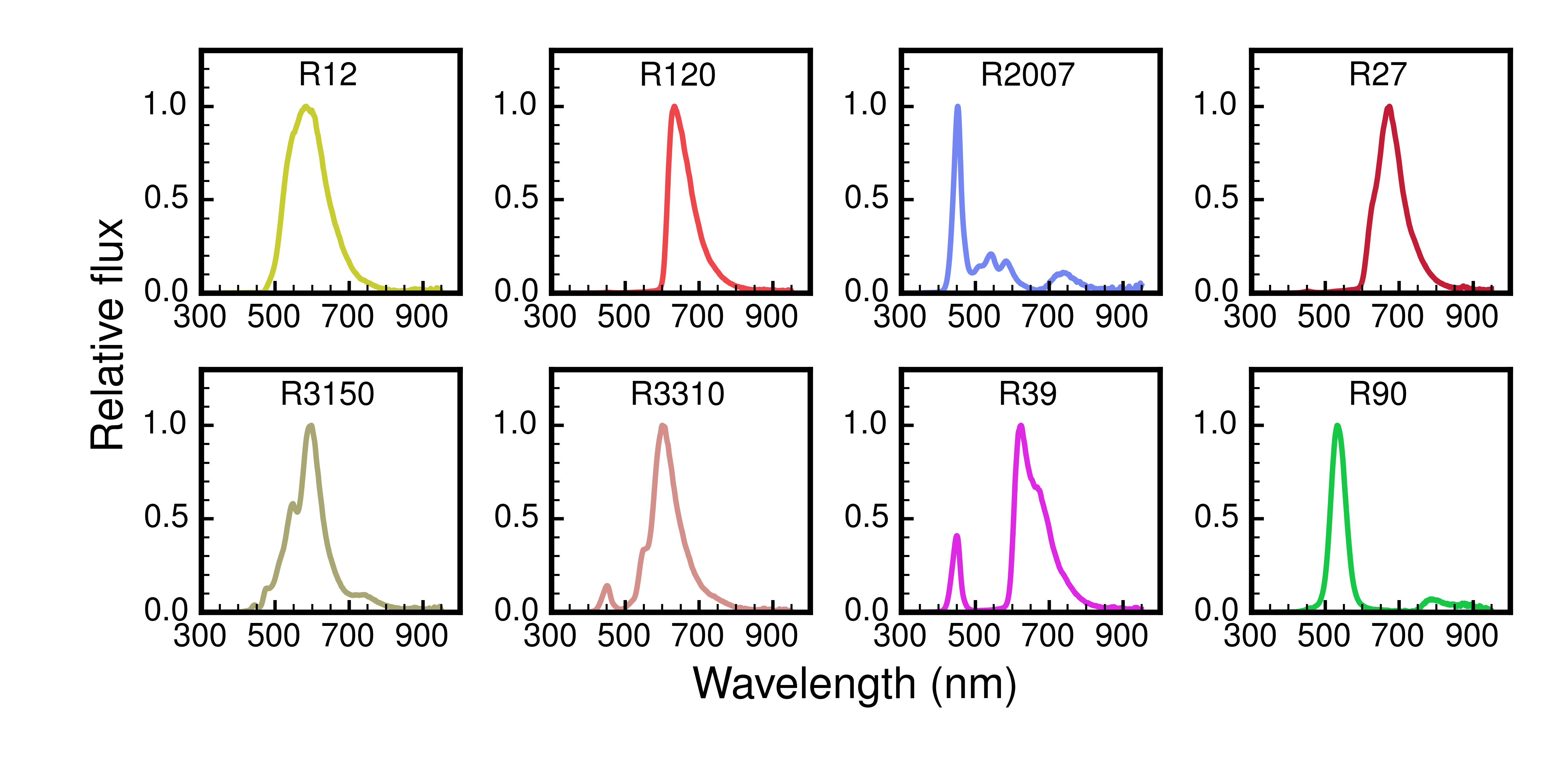

Supplement: Supplementary file 10 — Dataset EV2 [file MSB-13-926-s010.zip › dataset_ev2_ccasr_data_and_analysis/ccasr_analysis/plots/sv_led_spectra.png]

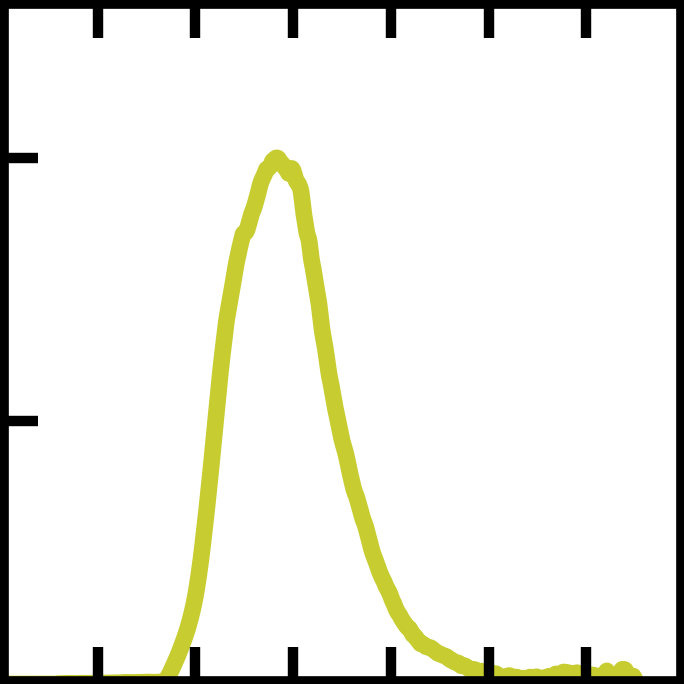

Supplement: Supplementary file 10 — Dataset EV2 [file MSB-13-926-s010.zip › dataset_ev2_ccasr_data_and_analysis/ccasr_analysis/plots/sv_led_spectra_R12.png]

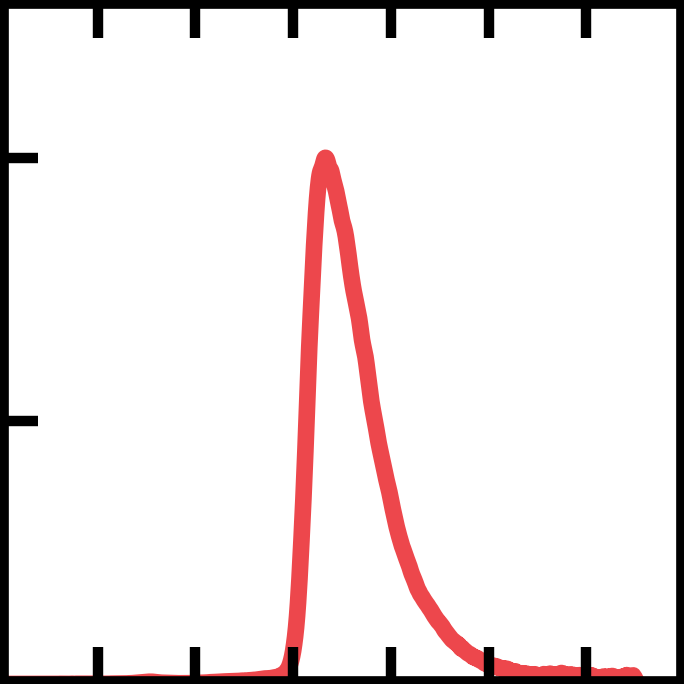

Supplement: Supplementary file 10 — Dataset EV2 [file MSB-13-926-s010.zip › dataset_ev2_ccasr_data_and_analysis/ccasr_analysis/plots/sv_led_spectra_R120.png]

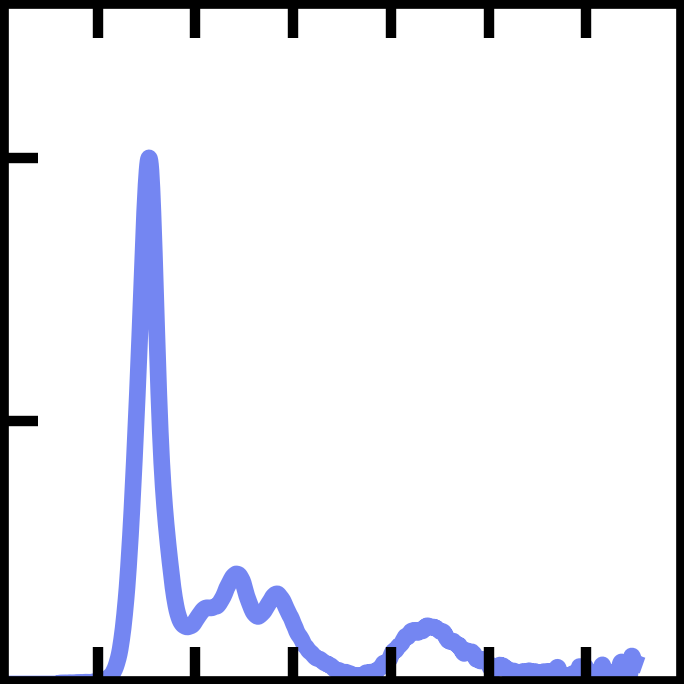

Supplement: Supplementary file 10 — Dataset EV2 [file MSB-13-926-s010.zip › dataset_ev2_ccasr_data_and_analysis/ccasr_analysis/plots/sv_led_spectra_R2007.png]

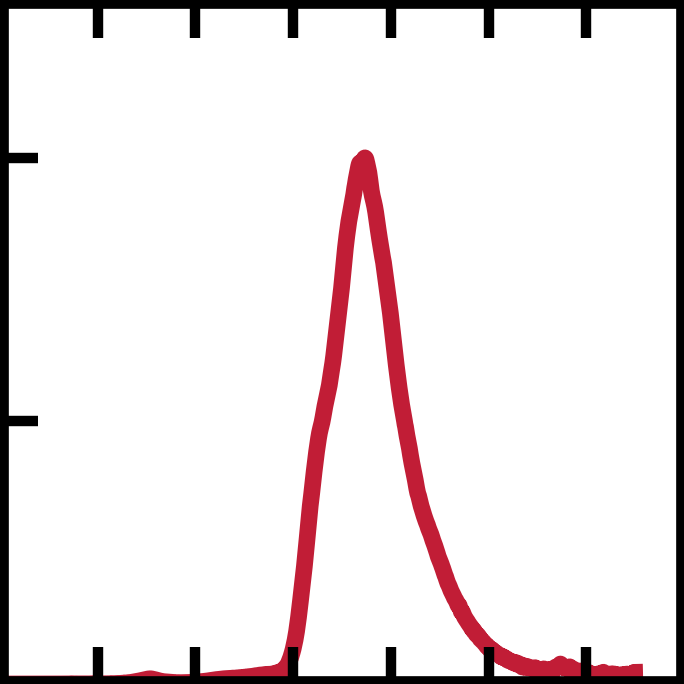

Supplement: Supplementary file 10 — Dataset EV2 [file MSB-13-926-s010.zip › dataset_ev2_ccasr_data_and_analysis/ccasr_analysis/plots/sv_led_spectra_R27.png]

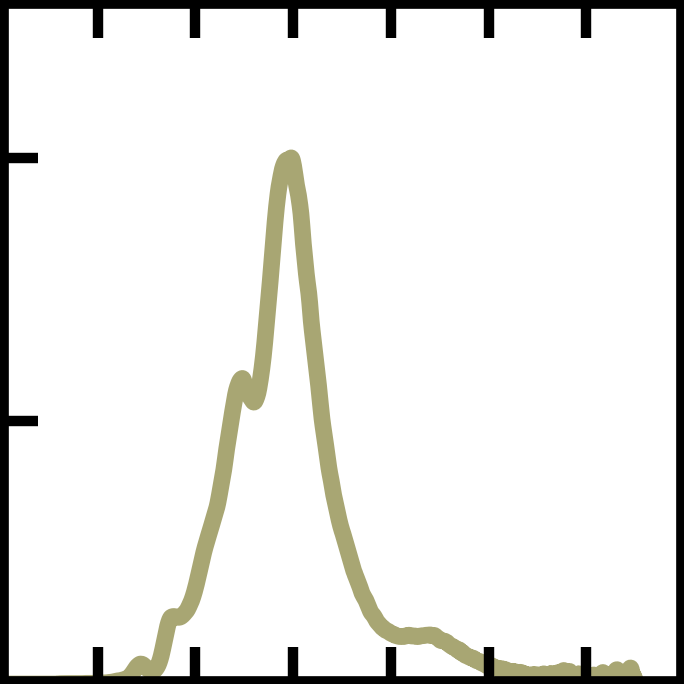

Supplement: Supplementary file 10 — Dataset EV2 [file MSB-13-926-s010.zip › dataset_ev2_ccasr_data_and_analysis/ccasr_analysis/plots/sv_led_spectra_R3150.png]

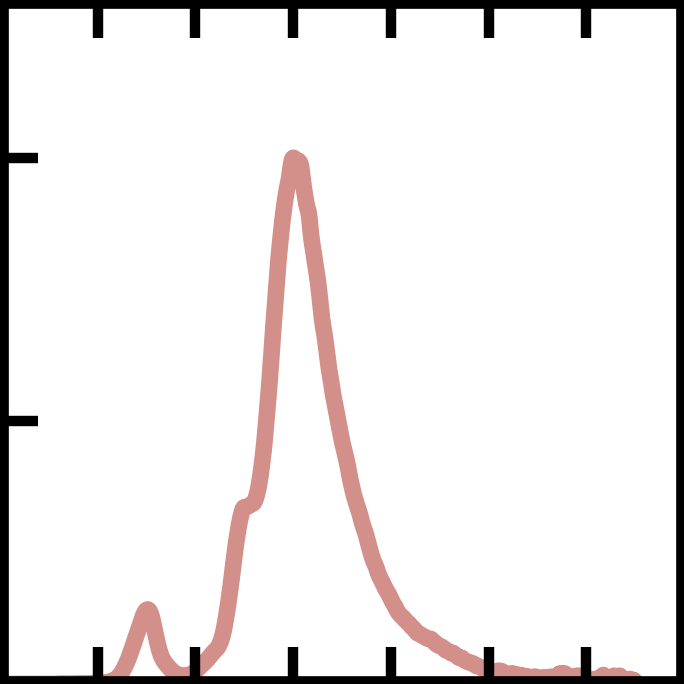

Supplement: Supplementary file 10 — Dataset EV2 [file MSB-13-926-s010.zip › dataset_ev2_ccasr_data_and_analysis/ccasr_analysis/plots/sv_led_spectra_R3310.png]

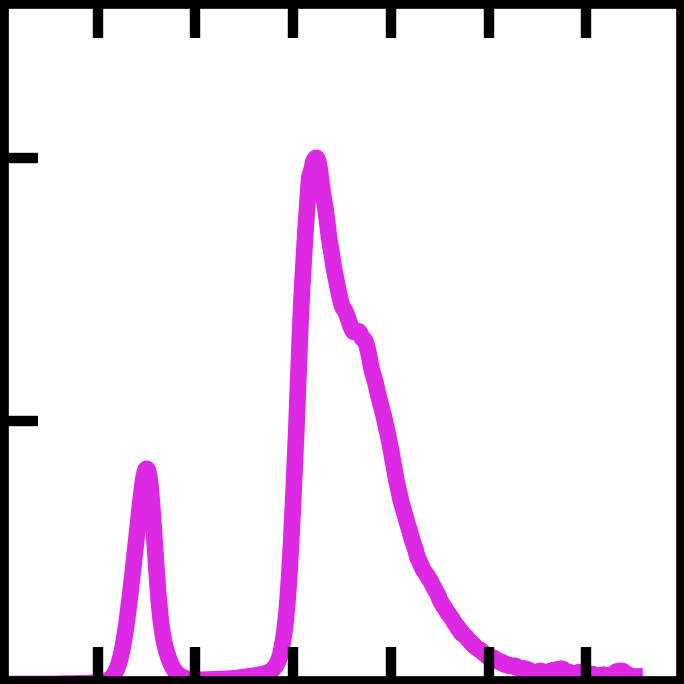

Supplement: Supplementary file 10 — Dataset EV2 [file MSB-13-926-s010.zip › dataset_ev2_ccasr_data_and_analysis/ccasr_analysis/plots/sv_led_spectra_R39.png]

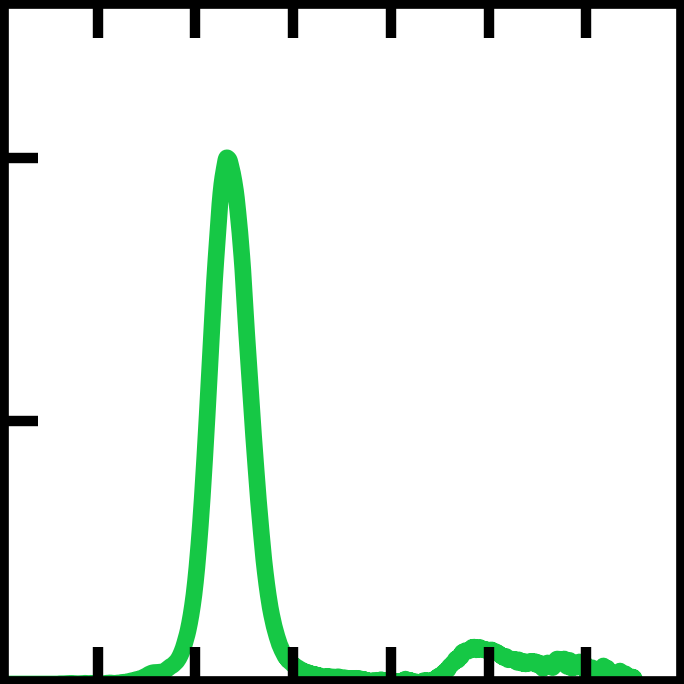

Supplement: Supplementary file 10 — Dataset EV2 [file MSB-13-926-s010.zip › dataset_ev2_ccasr_data_and_analysis/ccasr_analysis/plots/sv_led_spectra_R90.png]

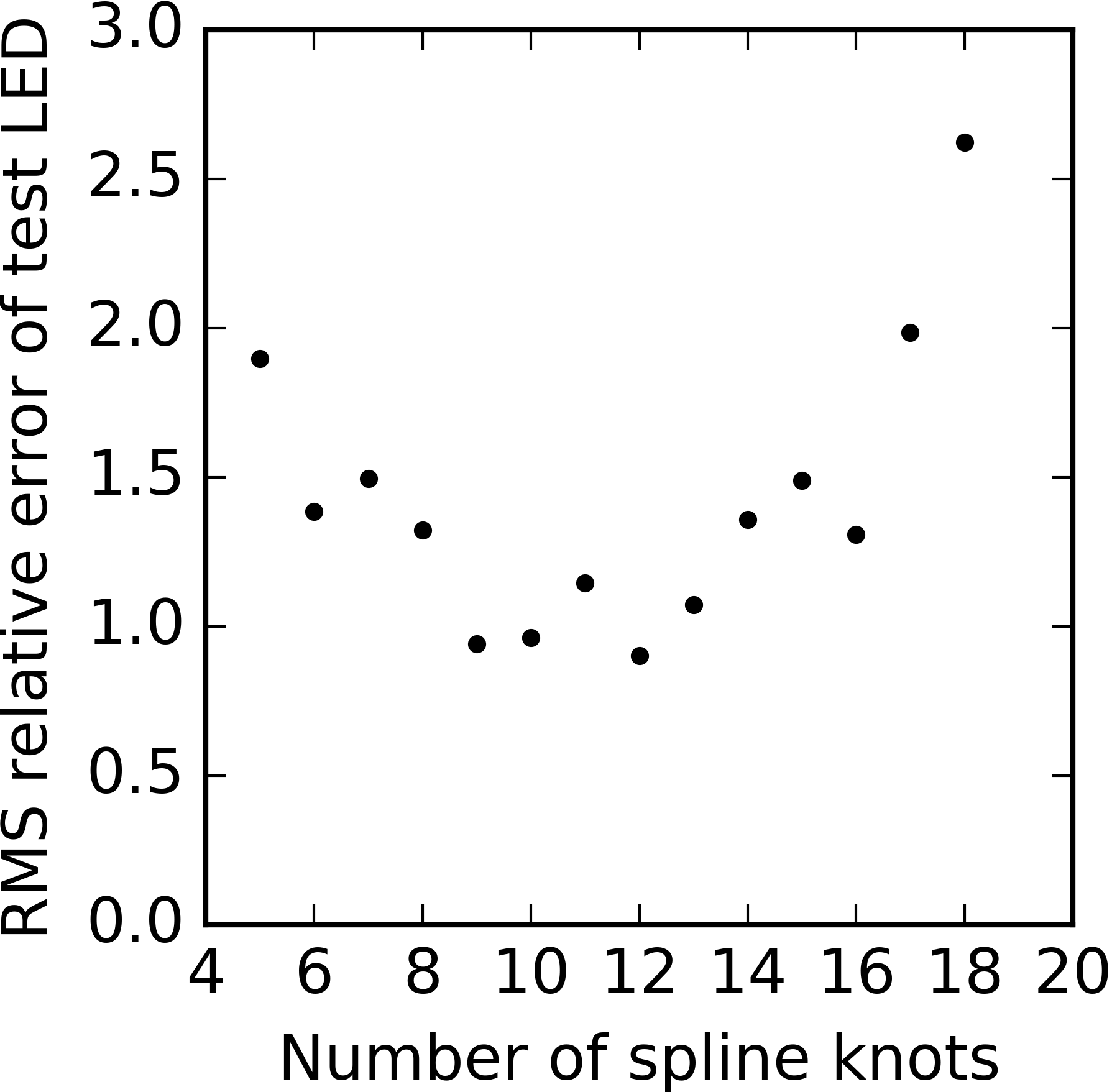

Supplement: Supplementary file 13 — Dataset EV5 [file MSB-13-926-s013.zip › dataset_ev5_pcs_spline_construction/plots/loocv_rdv2_k1.png]

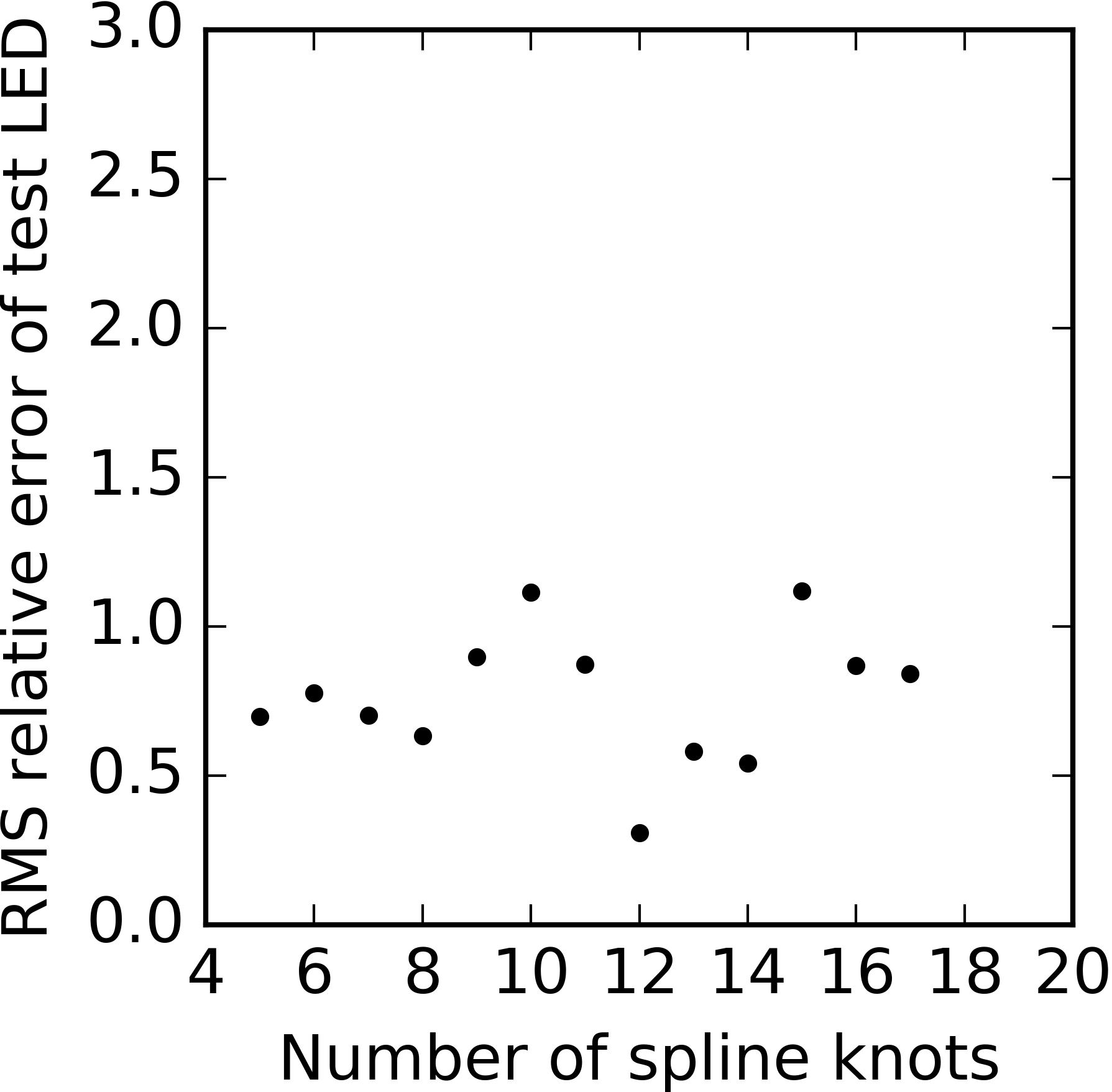

Supplement: Supplementary file 13 — Dataset EV5 [file MSB-13-926-s013.zip › dataset_ev5_pcs_spline_construction/plots/loocv_rdv2_k2.png]

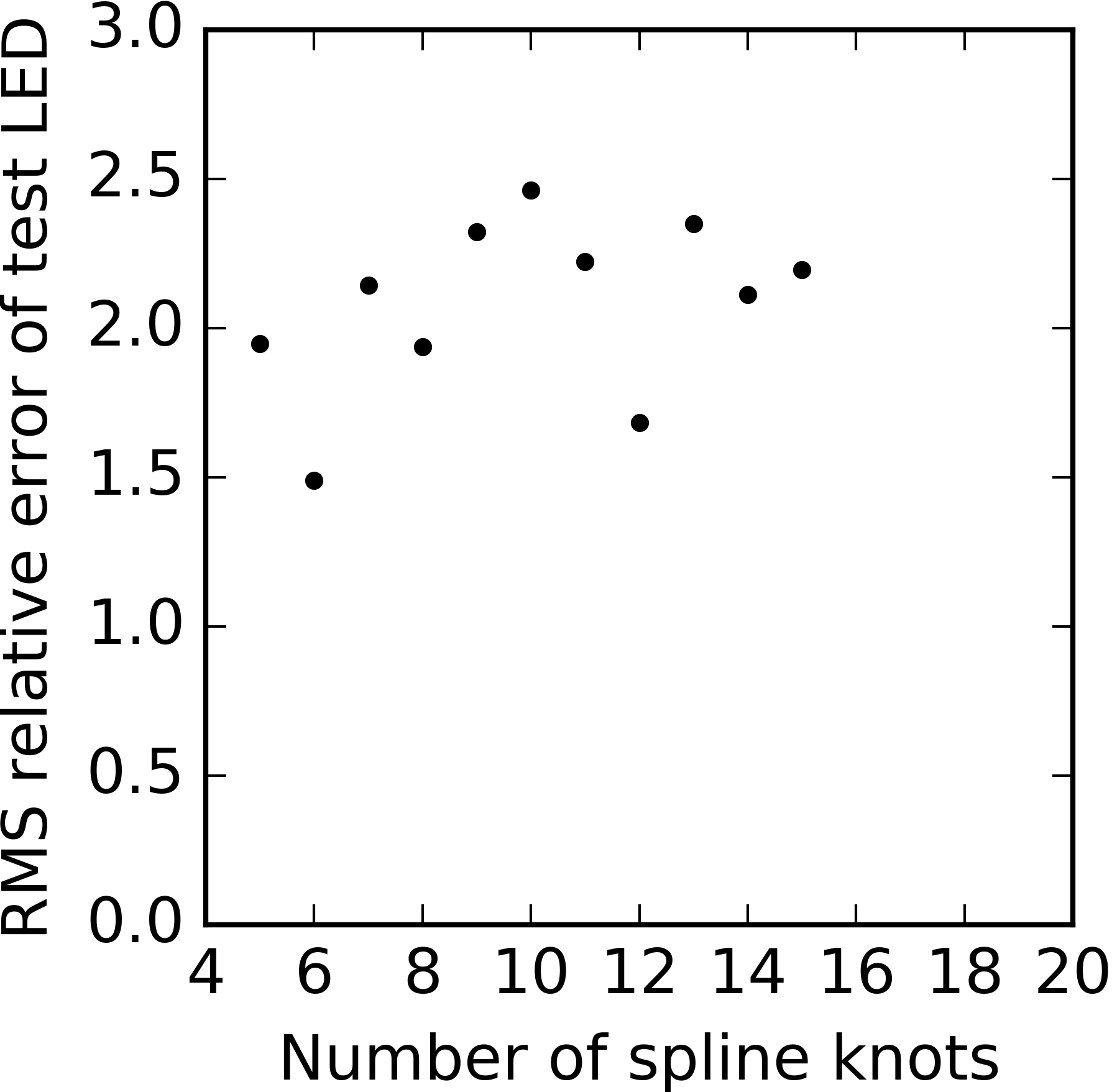

Supplement: Supplementary file 13 — Dataset EV5 [file MSB-13-926-s013.zip › dataset_ev5_pcs_spline_construction/plots/loocv_rgv2_k1.png]

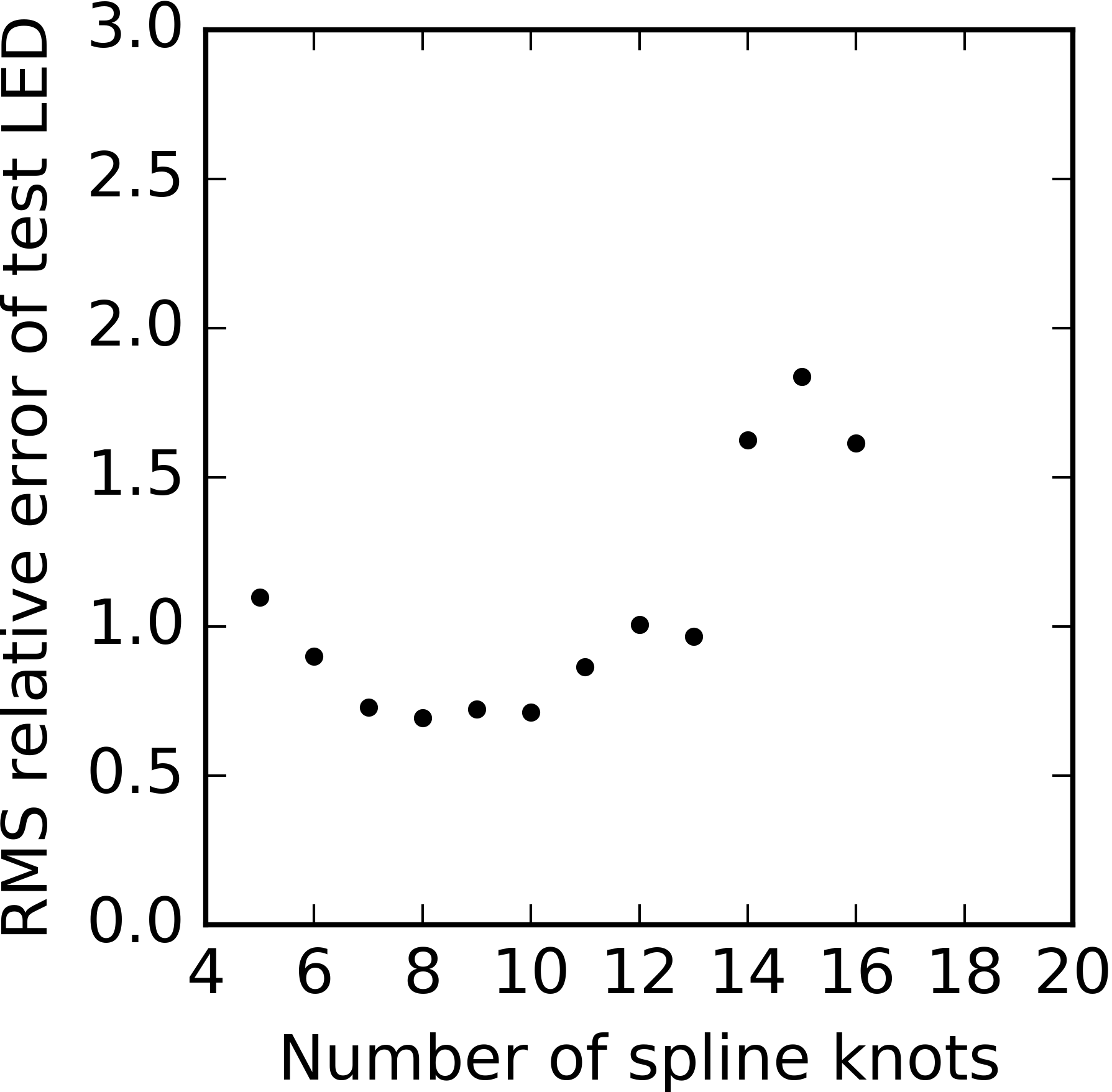

Supplement: Supplementary file 13 — Dataset EV5 [file MSB-13-926-s013.zip › dataset_ev5_pcs_spline_construction/plots/loocv_rgv2_k2.png]

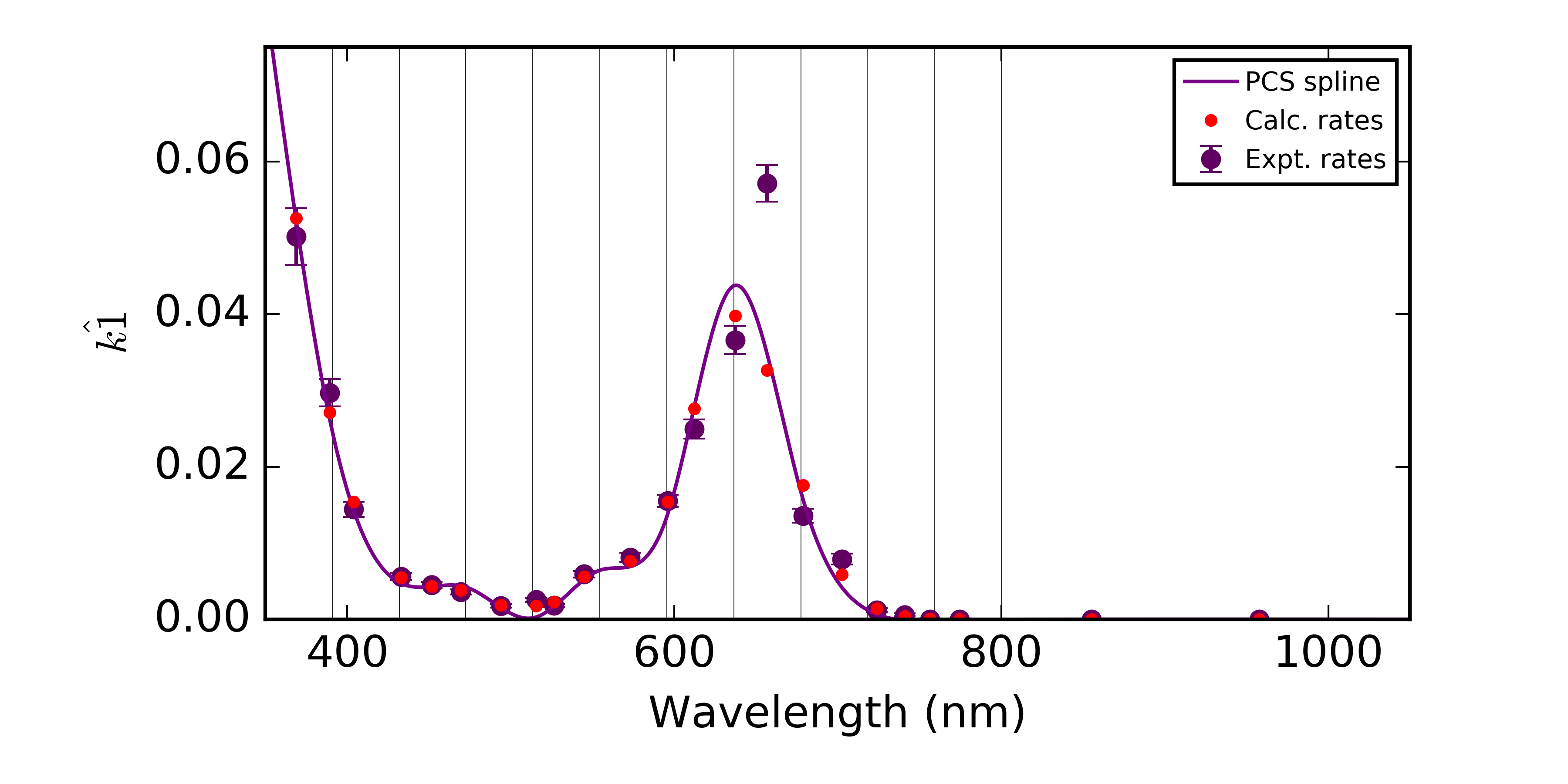

Supplement: Supplementary file 13 — Dataset EV5 [file MSB-13-926-s013.zip › dataset_ev5_pcs_spline_construction/plots/pcs_spline_rdv2_k1.png]

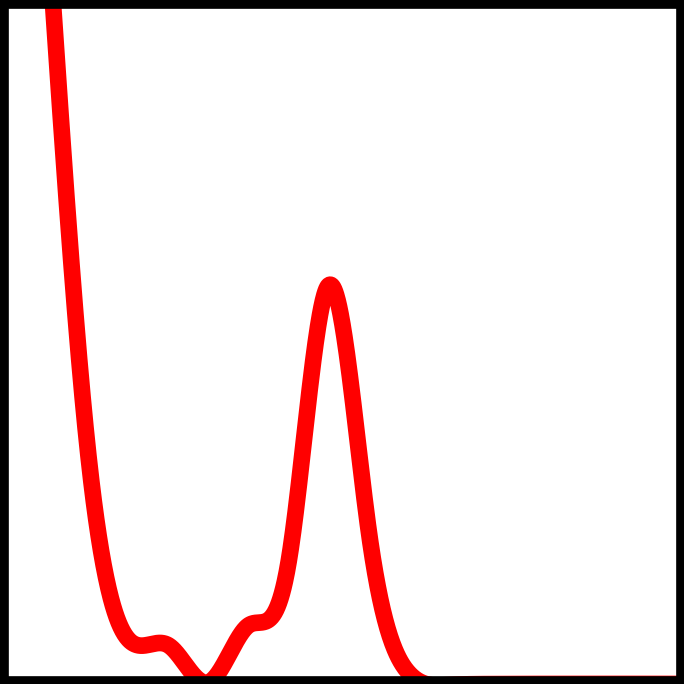

Supplement: Supplementary file 13 — Dataset EV5 [file MSB-13-926-s013.zip › dataset_ev5_pcs_spline_construction/plots/pcs_spline_rdv2_k1_cartoon.png]
