# Supplementary figures and images for: A photoconversion model for full spectral programming and multiplexing of optogenetic systems (part 2 of 3)
Source: Mol Syst Biol. 2017 Apr 24;13(4):926. doi: 10.15252/msb.20167456 (PMC5408778; doi:10.15252/msb.20167456)

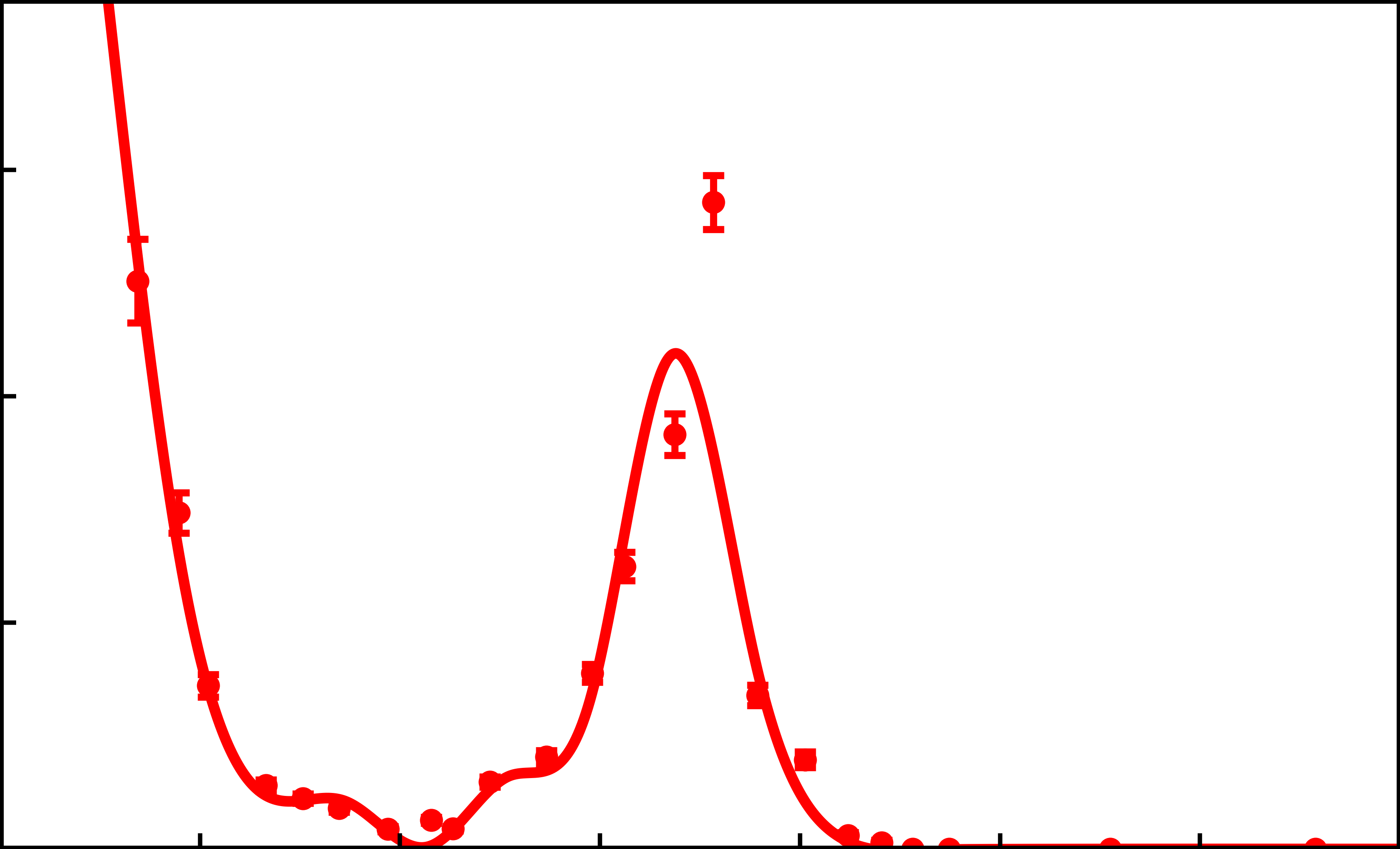

Supplement: Supplementary file 13 — Dataset EV5 [file MSB-13-926-s013.zip › dataset_ev5_pcs_spline_construction/plots/pcs_spline_rdv2_k1_square.png]

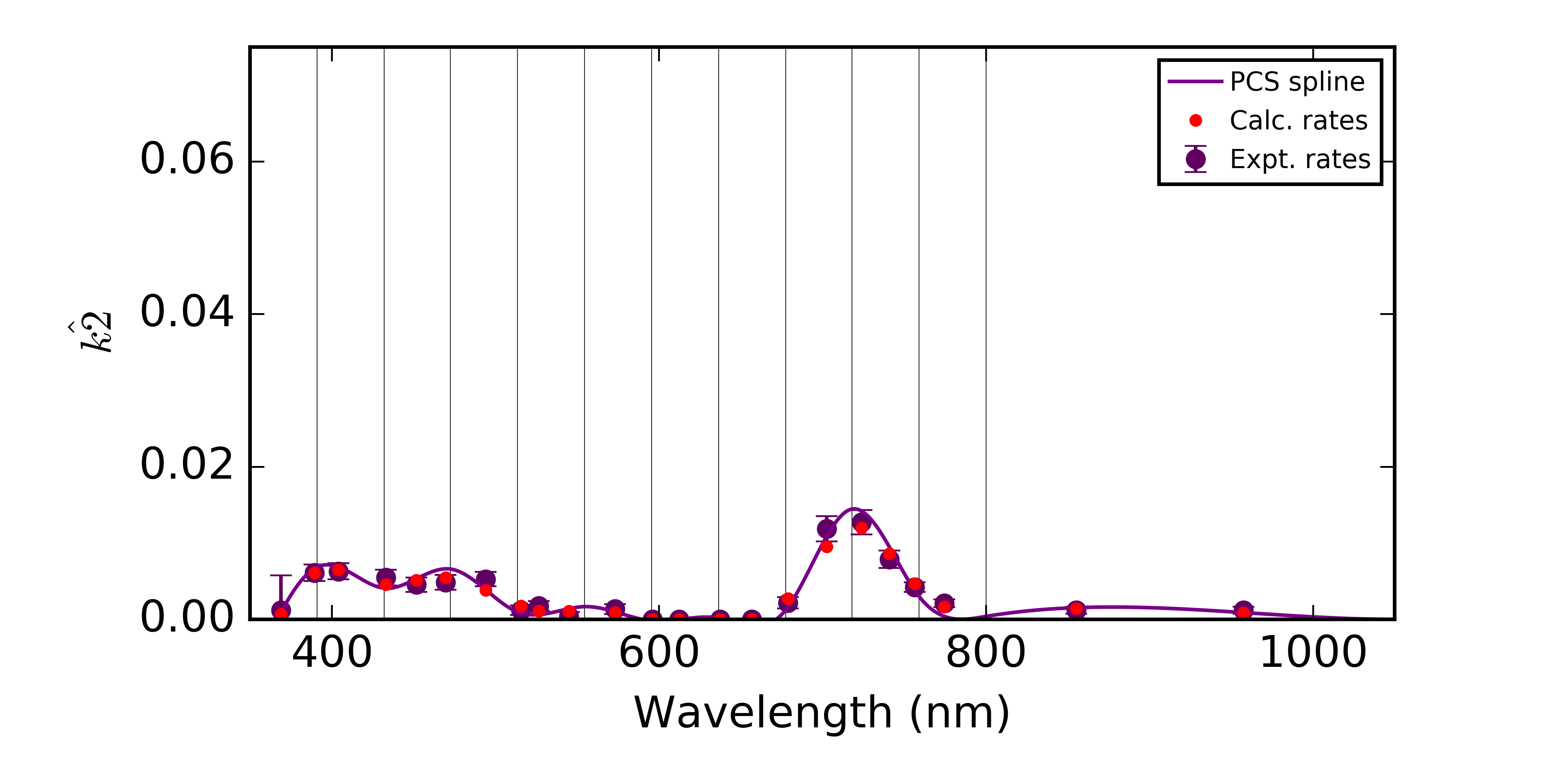

Supplement: Supplementary file 13 — Dataset EV5 [file MSB-13-926-s013.zip › dataset_ev5_pcs_spline_construction/plots/pcs_spline_rdv2_k2.png]

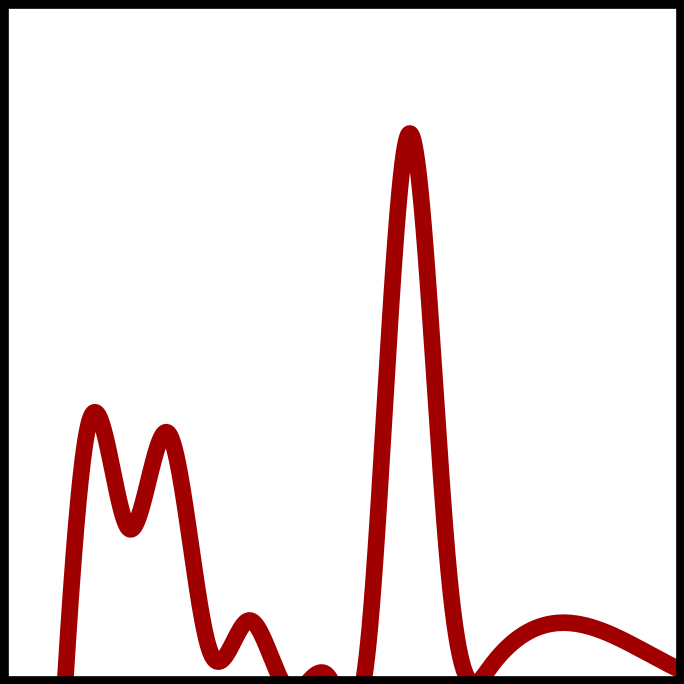

Supplement: Supplementary file 13 — Dataset EV5 [file MSB-13-926-s013.zip › dataset_ev5_pcs_spline_construction/plots/pcs_spline_rdv2_k2_cartoon.png]

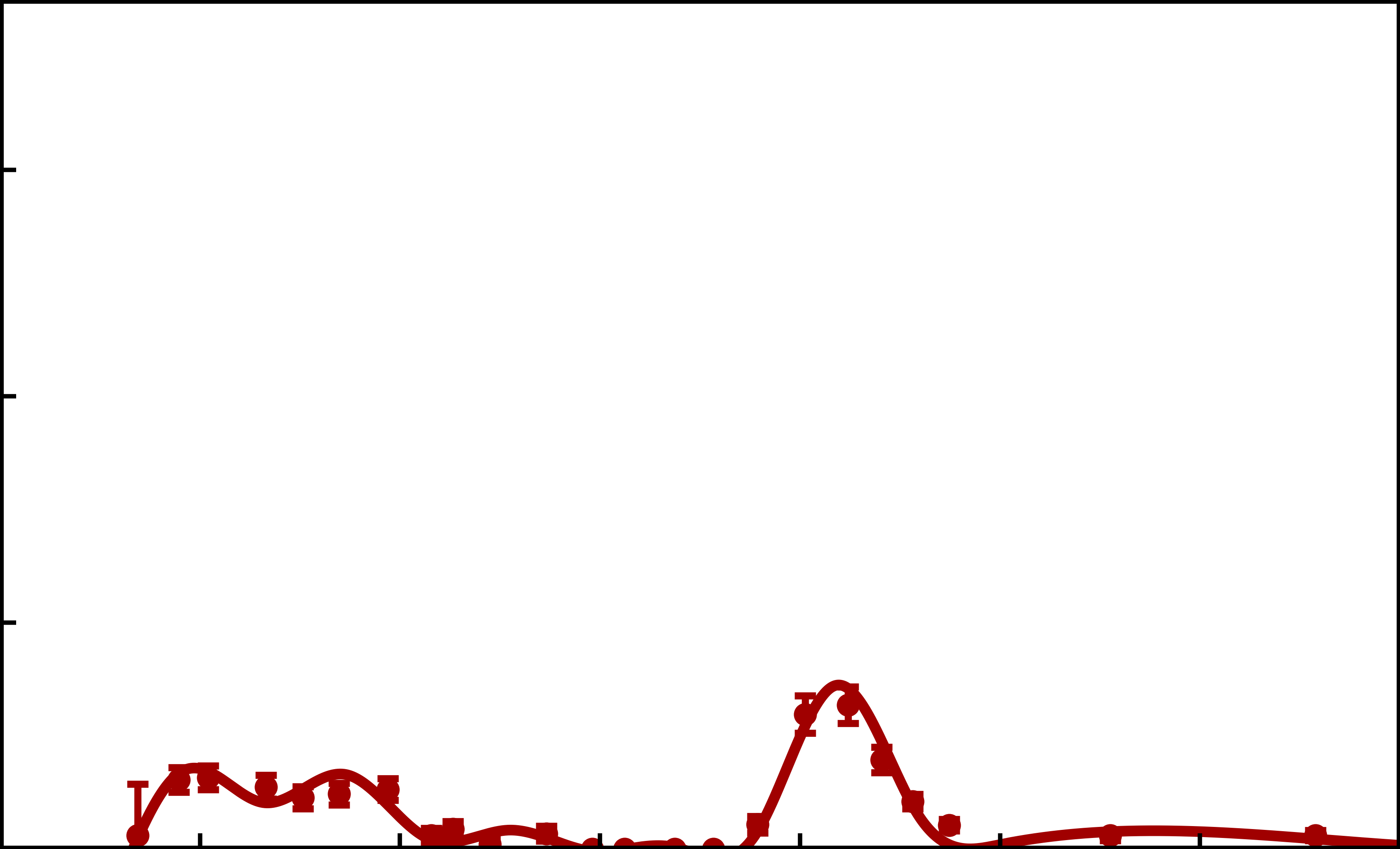

Supplement: Supplementary file 13 — Dataset EV5 [file MSB-13-926-s013.zip › dataset_ev5_pcs_spline_construction/plots/pcs_spline_rdv2_k2_square.png]

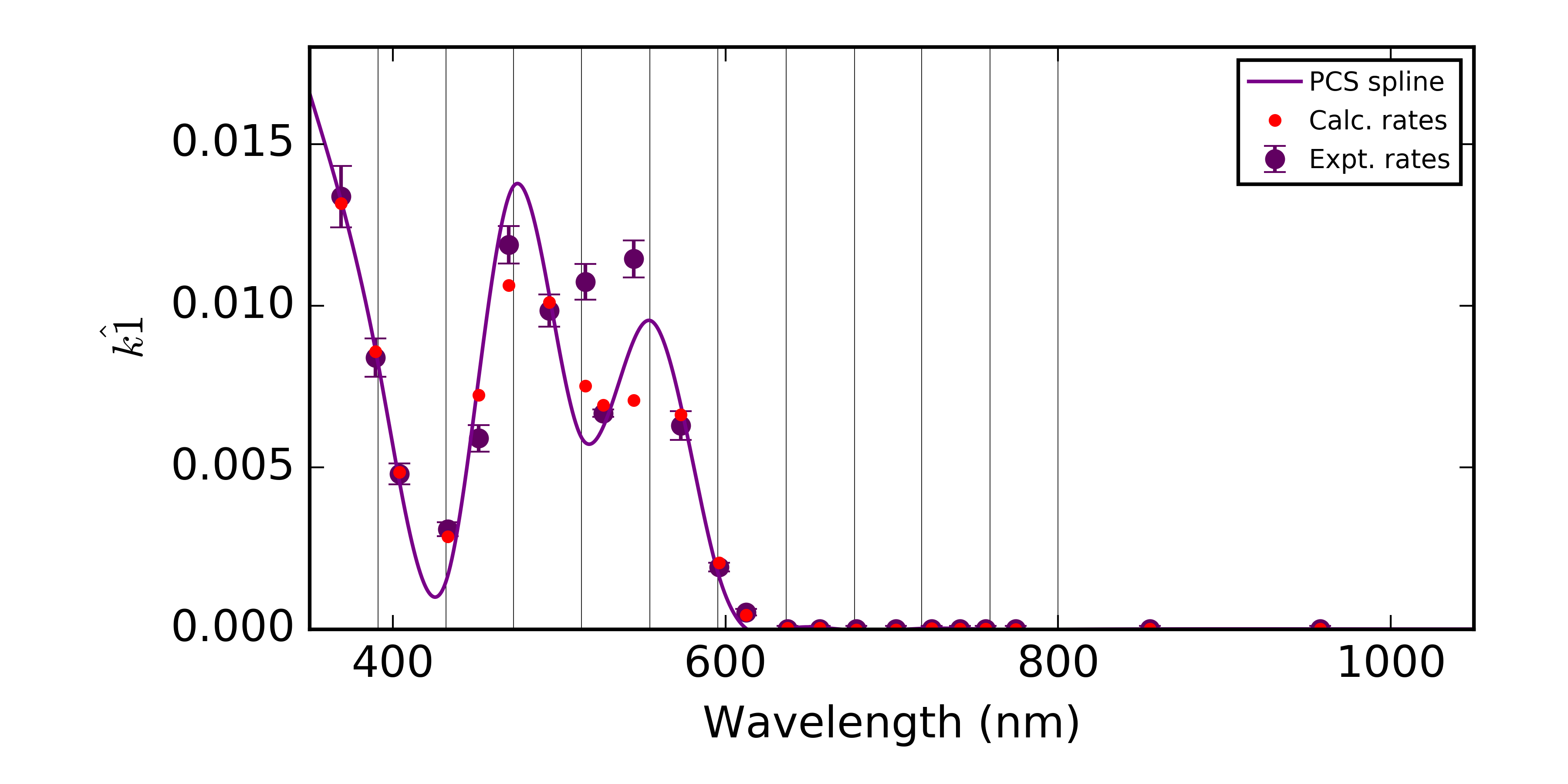

Supplement: Supplementary file 13 — Dataset EV5 [file MSB-13-926-s013.zip › dataset_ev5_pcs_spline_construction/plots/pcs_spline_rgv2_k1.png]

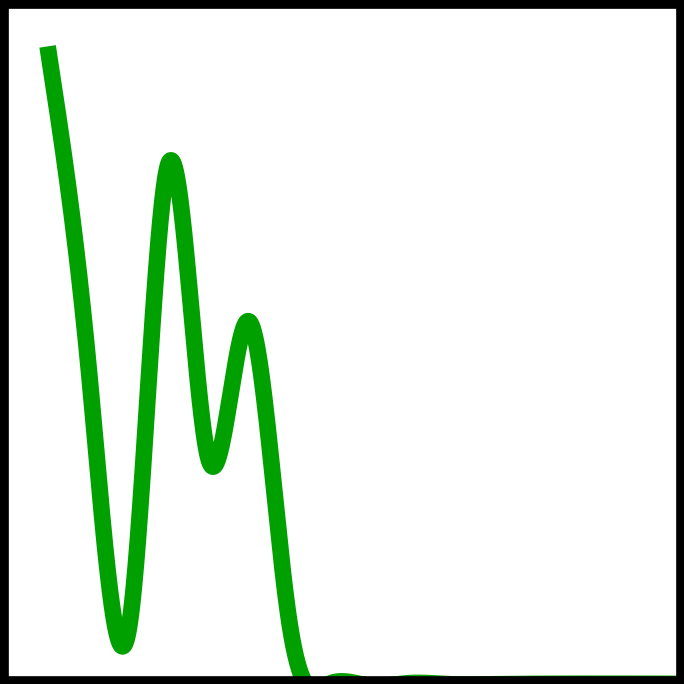

Supplement: Supplementary file 13 — Dataset EV5 [file MSB-13-926-s013.zip › dataset_ev5_pcs_spline_construction/plots/pcs_spline_rgv2_k1_cartoon.png]

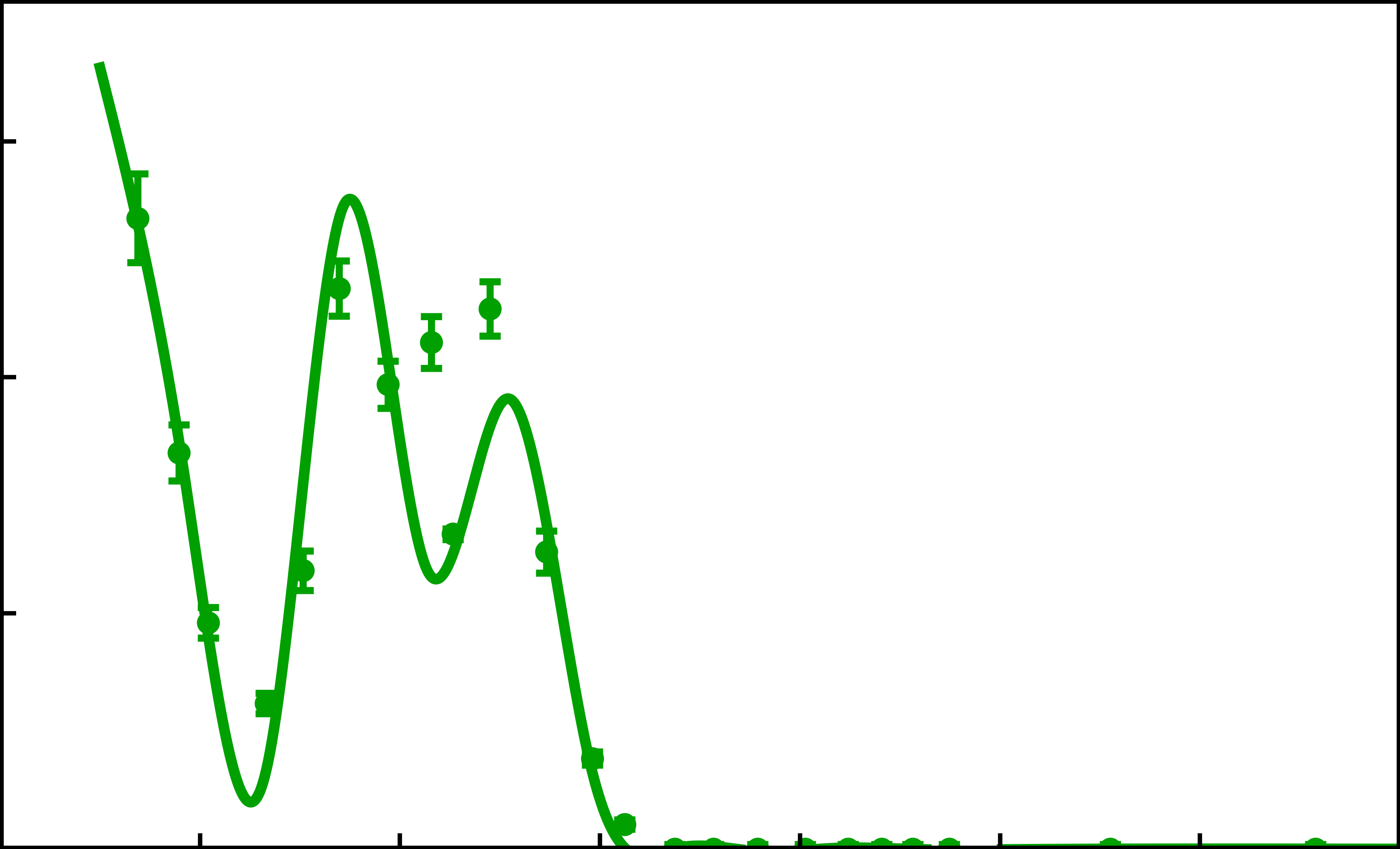

Supplement: Supplementary file 13 — Dataset EV5 [file MSB-13-926-s013.zip › dataset_ev5_pcs_spline_construction/plots/pcs_spline_rgv2_k1_square.png]

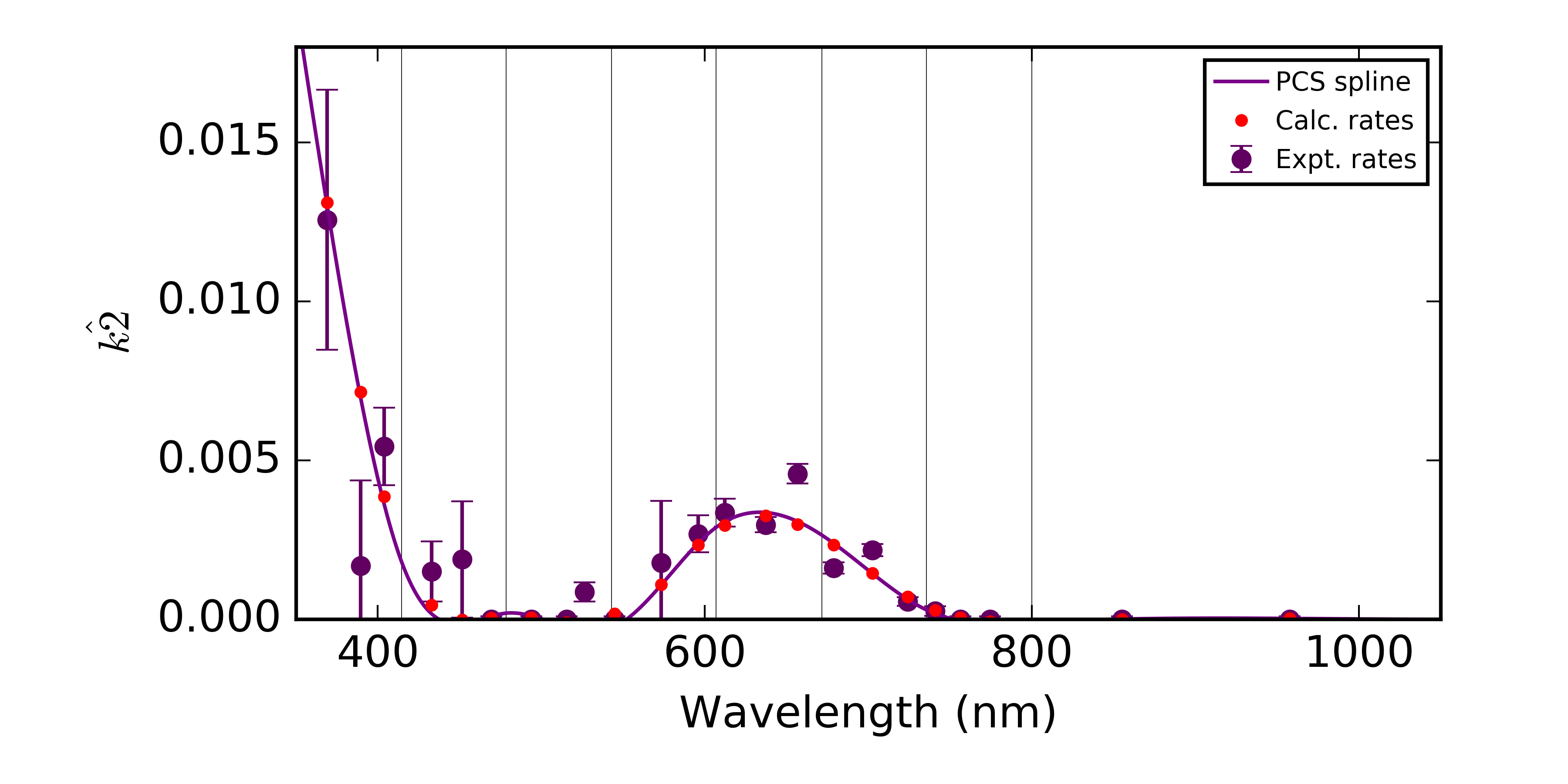

Supplement: Supplementary file 13 — Dataset EV5 [file MSB-13-926-s013.zip › dataset_ev5_pcs_spline_construction/plots/pcs_spline_rgv2_k2.png]

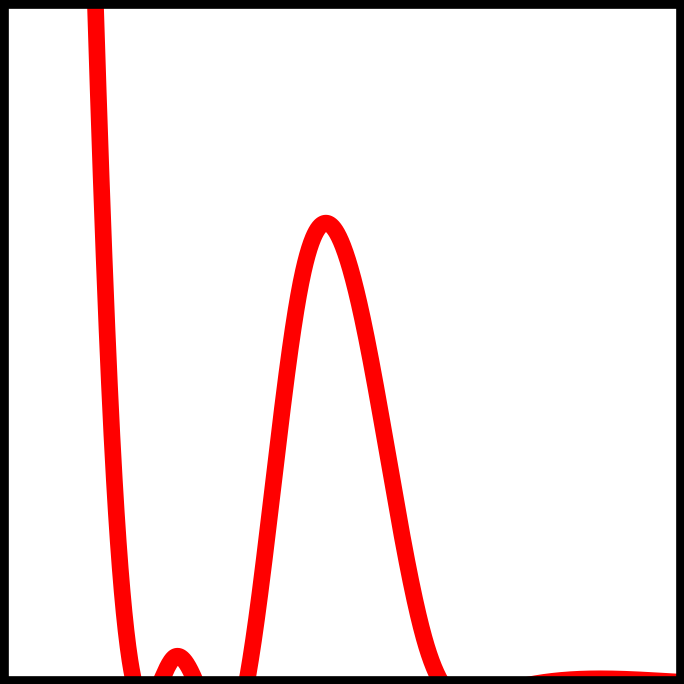

Supplement: Supplementary file 13 — Dataset EV5 [file MSB-13-926-s013.zip › dataset_ev5_pcs_spline_construction/plots/pcs_spline_rgv2_k2_cartoon.png]

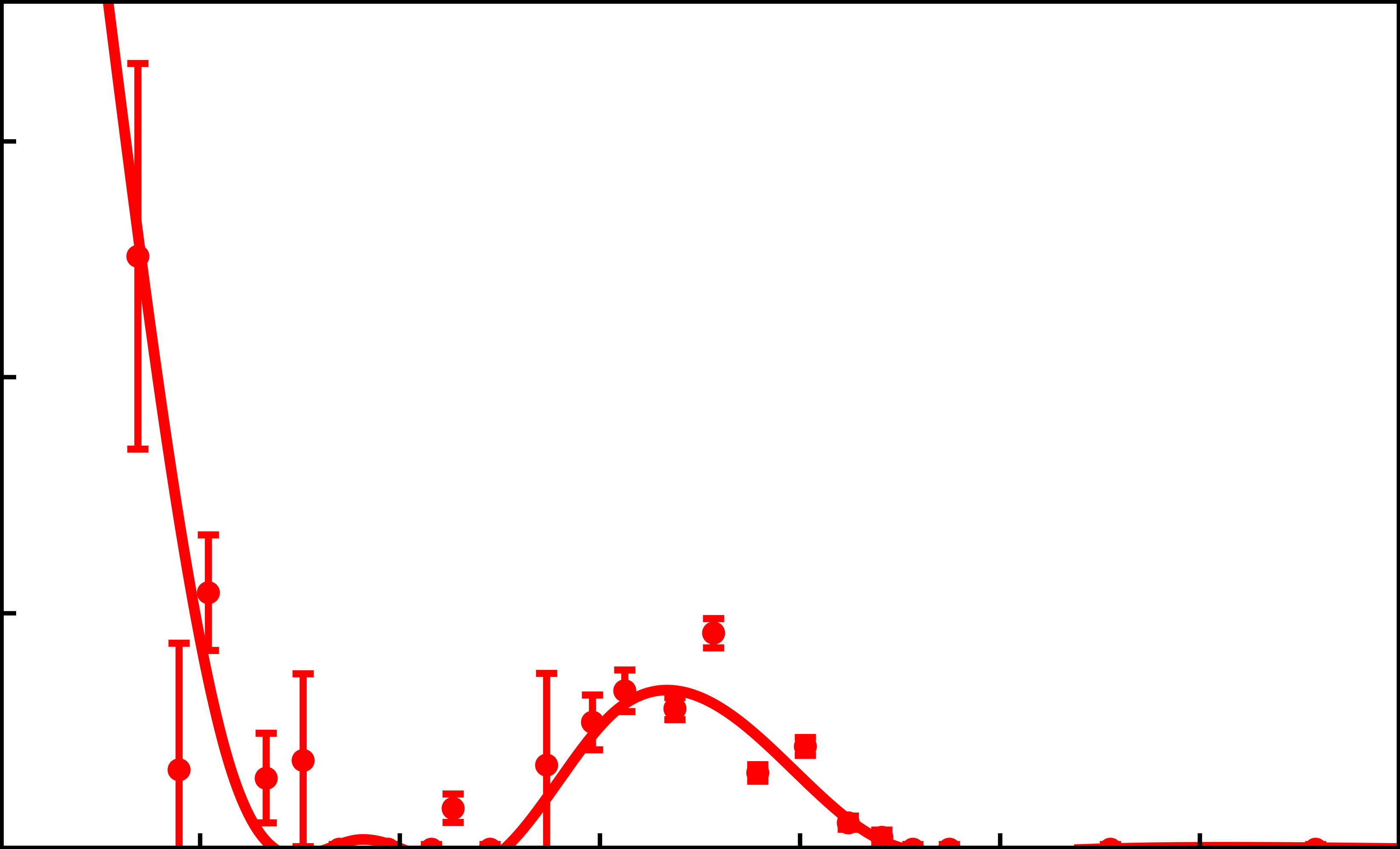

Supplement: Supplementary file 13 — Dataset EV5 [file MSB-13-926-s013.zip › dataset_ev5_pcs_spline_construction/plots/pcs_spline_rgv2_k2_square.png]

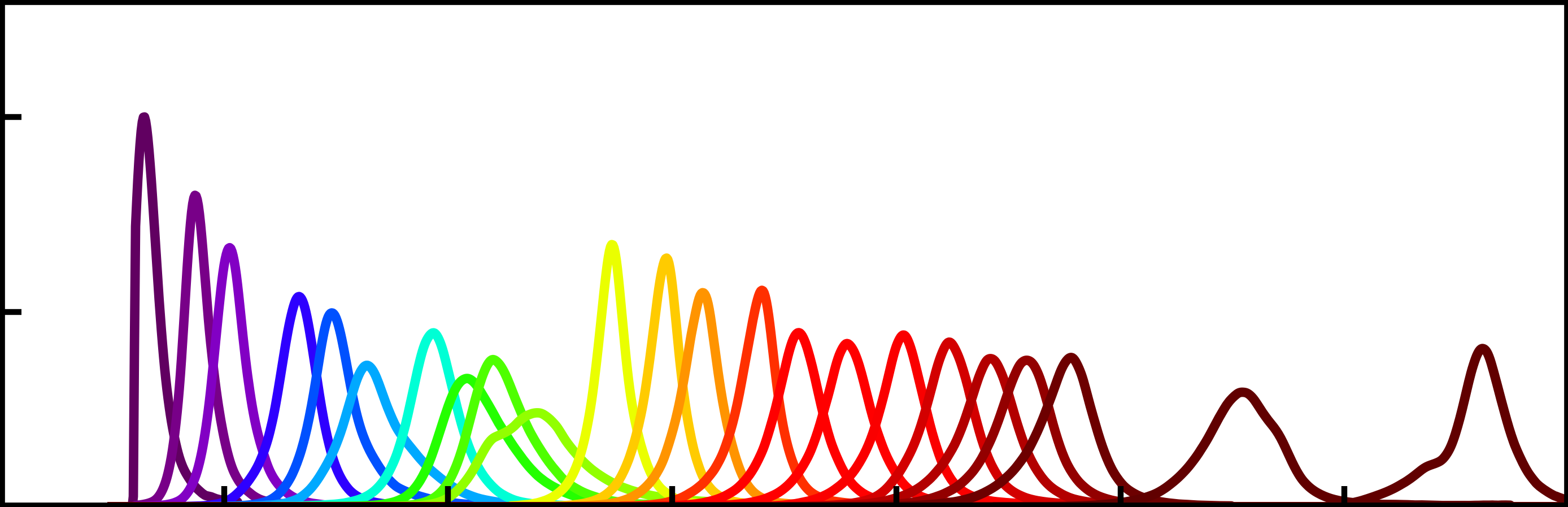

Supplement: Supplementary file 13 — Dataset EV5 [file MSB-13-926-s013.zip › dataset_ev5_pcs_spline_construction/plots/scleds_normalized.png]

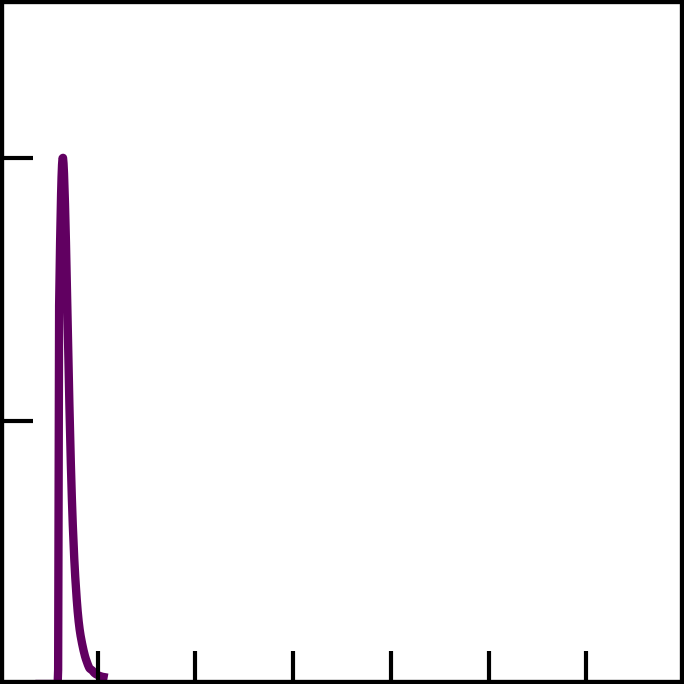

Supplement: Supplementary file 13 — Dataset EV5 [file MSB-13-926-s013.zip › dataset_ev5_pcs_spline_construction/plots/sc_led_spectra_361-LS.png]

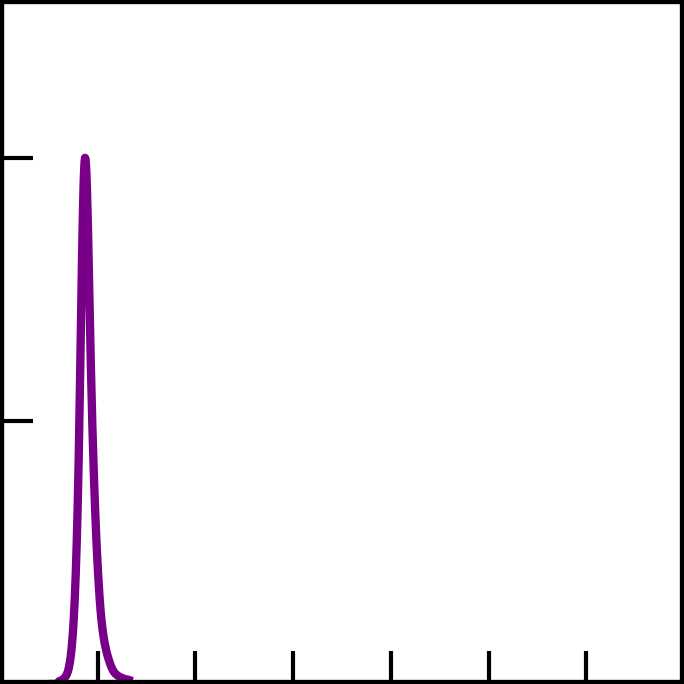

Supplement: Supplementary file 13 — Dataset EV5 [file MSB-13-926-s013.zip › dataset_ev5_pcs_spline_construction/plots/sc_led_spectra_380-SB.png]

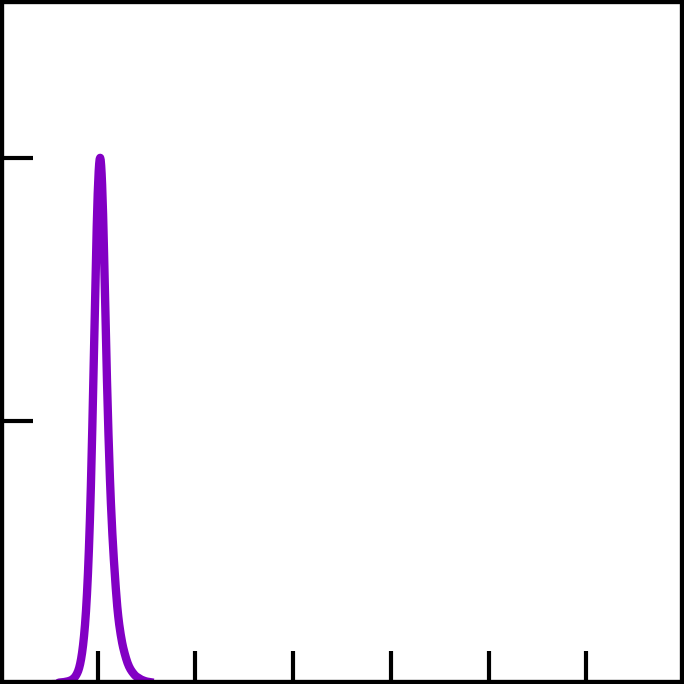

Supplement: Supplementary file 13 — Dataset EV5 [file MSB-13-926-s013.zip › dataset_ev5_pcs_spline_construction/plots/sc_led_spectra_405-SB.png]

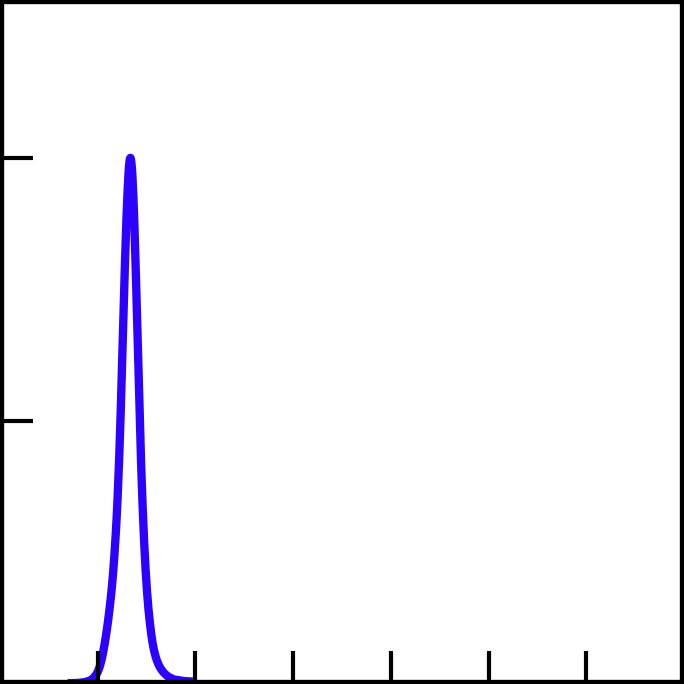

Supplement: Supplementary file 13 — Dataset EV5 [file MSB-13-926-s013.zip › dataset_ev5_pcs_spline_construction/plots/sc_led_spectra_430-MB.png]

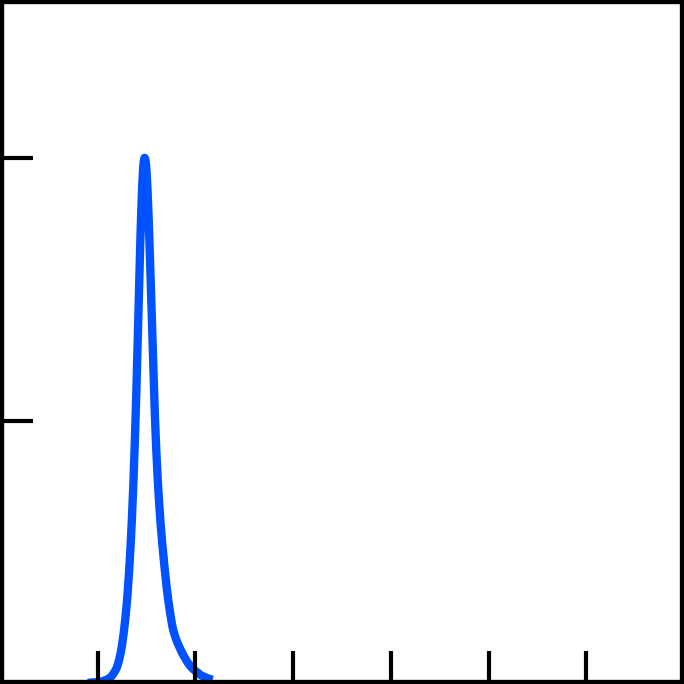

Supplement: Supplementary file 13 — Dataset EV5 [file MSB-13-926-s013.zip › dataset_ev5_pcs_spline_construction/plots/sc_led_spectra_450-MB.png]

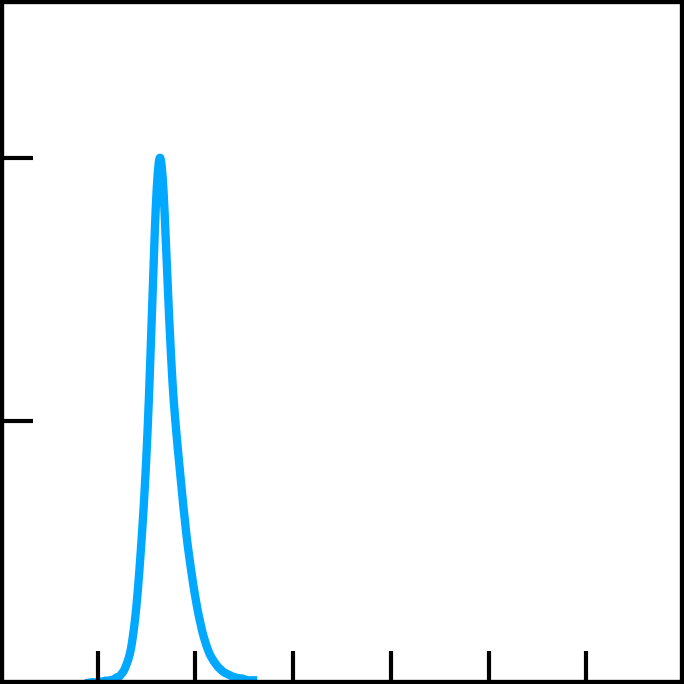

Supplement: Supplementary file 13 — Dataset EV5 [file MSB-13-926-s013.zip › dataset_ev5_pcs_spline_construction/plots/sc_led_spectra_470-SB.png]

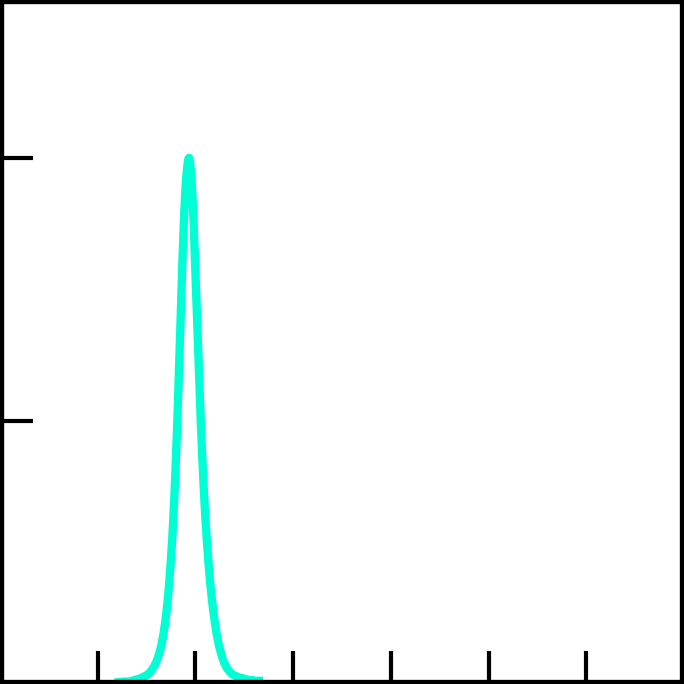

Supplement: Supplementary file 13 — Dataset EV5 [file MSB-13-926-s013.zip › dataset_ev5_pcs_spline_construction/plots/sc_led_spectra_490-MB_R383.png]

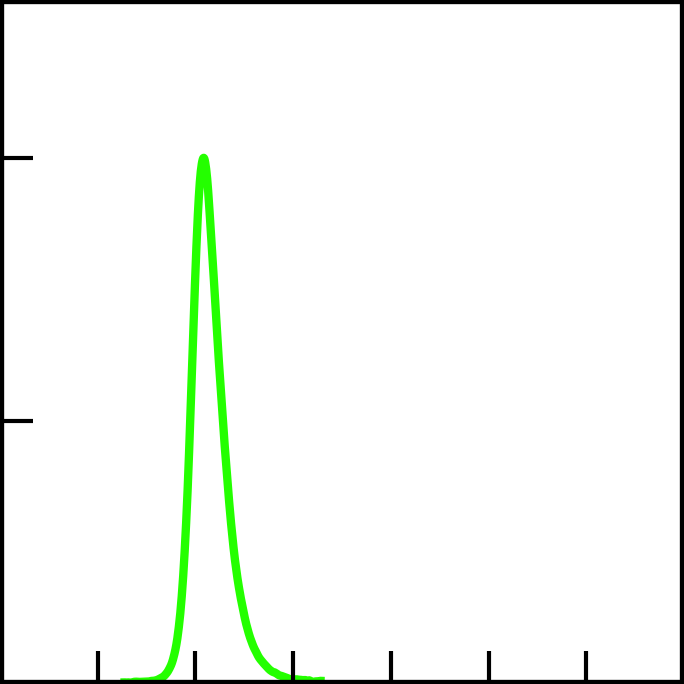

Supplement: Supplementary file 13 — Dataset EV5 [file MSB-13-926-s013.zip › dataset_ev5_pcs_spline_construction/plots/sc_led_spectra_505-SB.png]

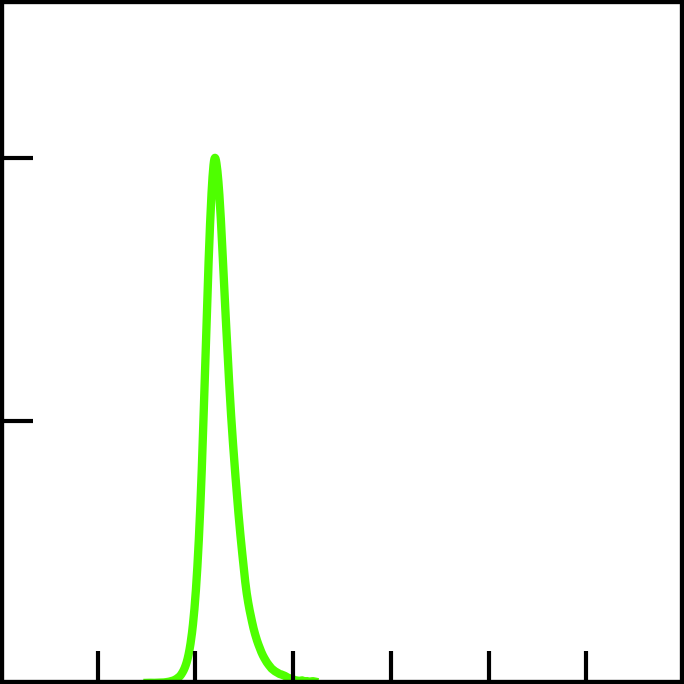

Supplement: Supplementary file 13 — Dataset EV5 [file MSB-13-926-s013.zip › dataset_ev5_pcs_spline_construction/plots/sc_led_spectra_520-2-KB.png]

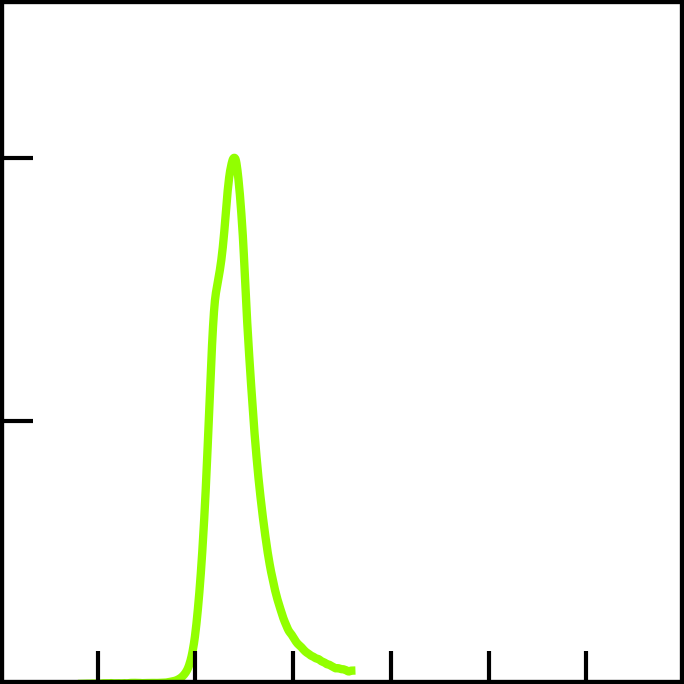

Supplement: Supplementary file 13 — Dataset EV5 [file MSB-13-926-s013.zip › dataset_ev5_pcs_spline_construction/plots/sc_led_spectra_525-SB_R2003.png]

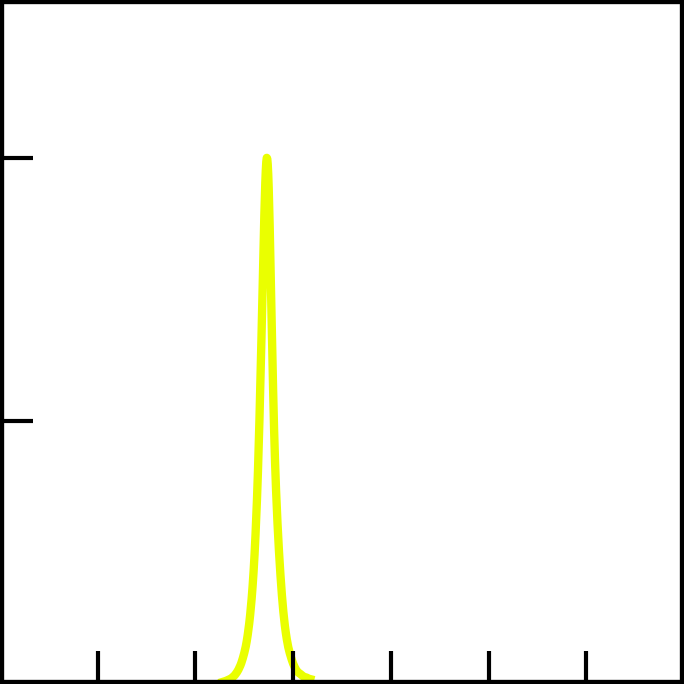

Supplement: Supplementary file 13 — Dataset EV5 [file MSB-13-926-s013.zip › dataset_ev5_pcs_spline_construction/plots/sc_led_spectra_570-KB.png]

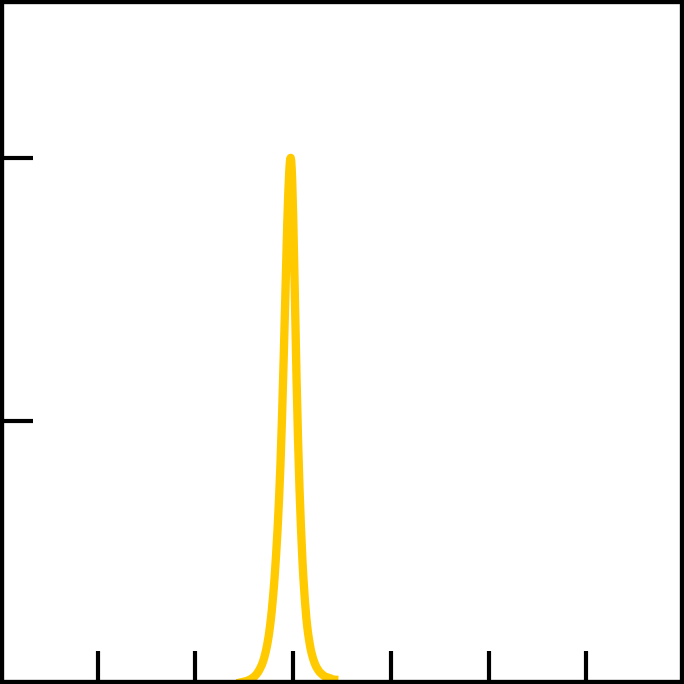

Supplement: Supplementary file 13 — Dataset EV5 [file MSB-13-926-s013.zip › dataset_ev5_pcs_spline_construction/plots/sc_led_spectra_590-SB.png]

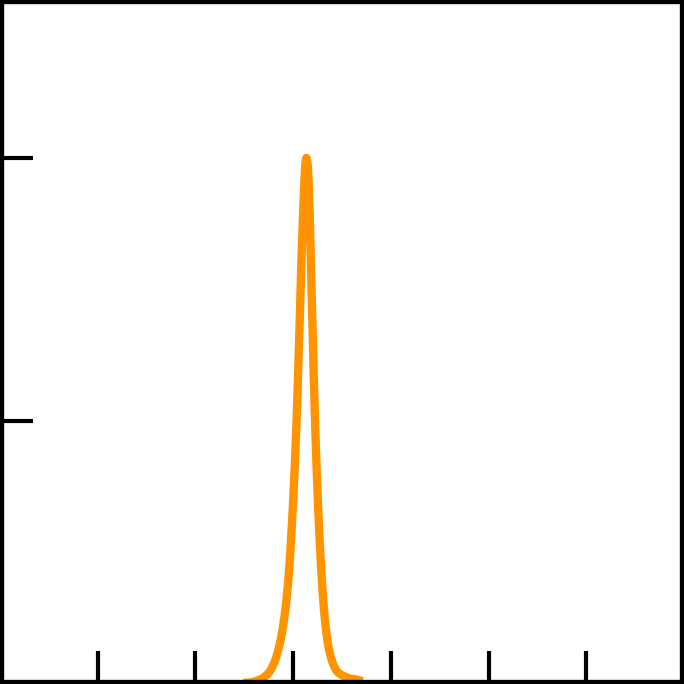

Supplement: Supplementary file 13 — Dataset EV5 [file MSB-13-926-s013.zip › dataset_ev5_pcs_spline_construction/plots/sc_led_spectra_605-SB.png]

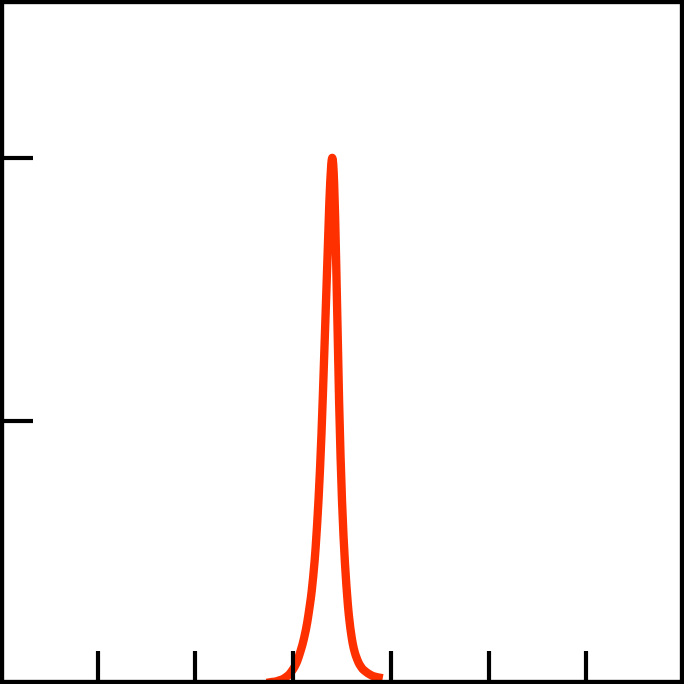

Supplement: Supplementary file 13 — Dataset EV5 [file MSB-13-926-s013.zip › dataset_ev5_pcs_spline_construction/plots/sc_led_spectra_630-SB.png]

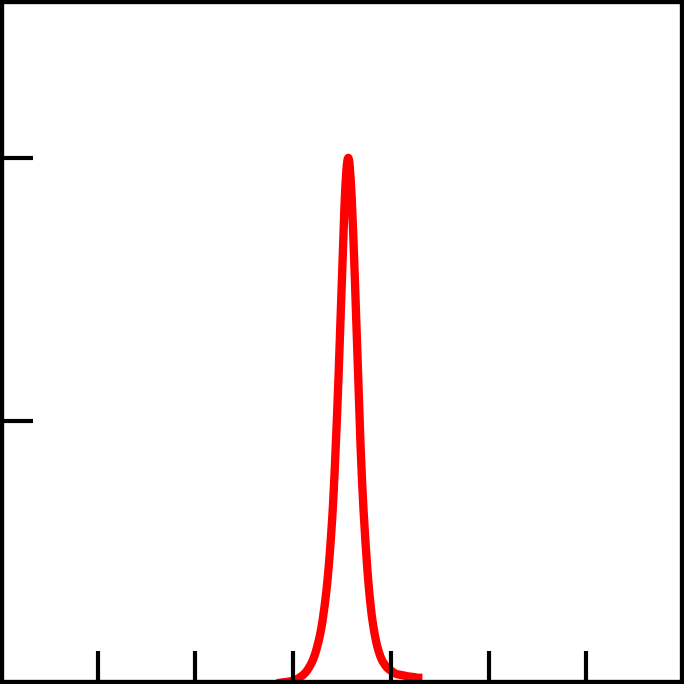

Supplement: Supplementary file 13 — Dataset EV5 [file MSB-13-926-s013.zip › dataset_ev5_pcs_spline_construction/plots/sc_led_spectra_660-LS.png]

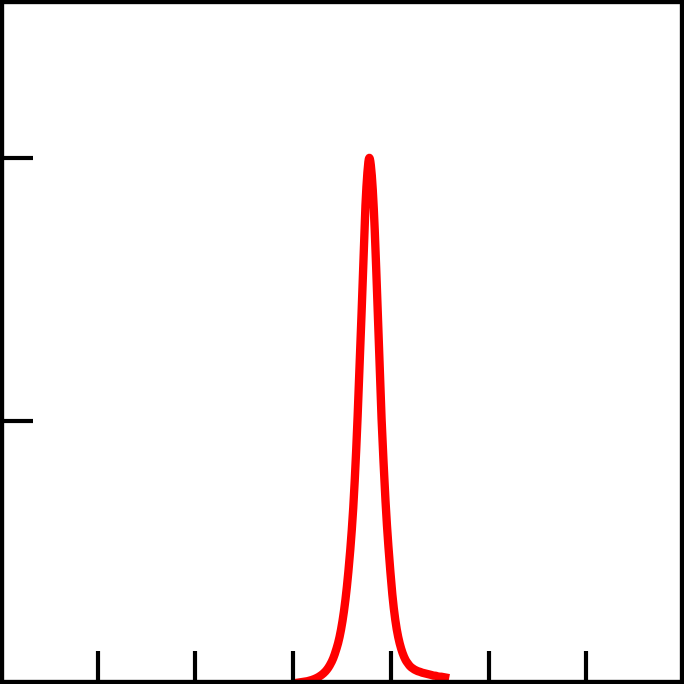

Supplement: Supplementary file 13 — Dataset EV5 [file MSB-13-926-s013.zip › dataset_ev5_pcs_spline_construction/plots/sc_led_spectra_680-MT.png]

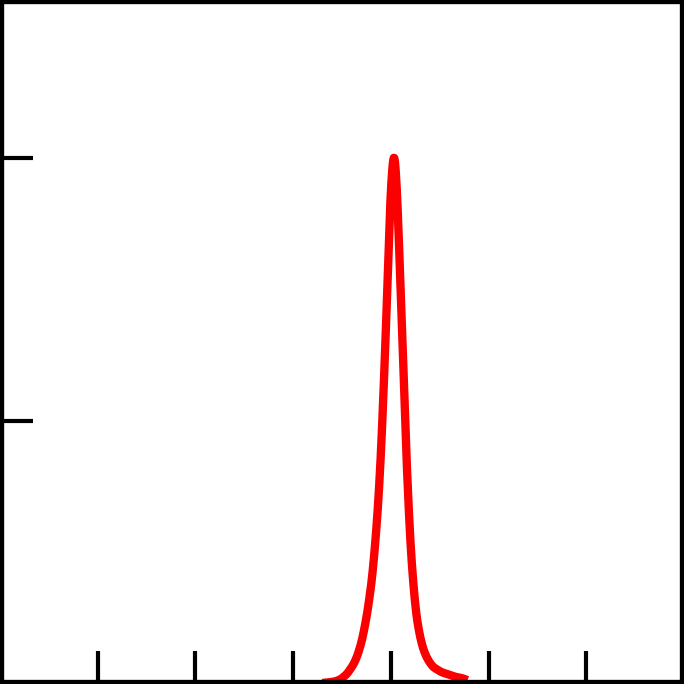

Supplement: Supplementary file 13 — Dataset EV5 [file MSB-13-926-s013.zip › dataset_ev5_pcs_spline_construction/plots/sc_led_spectra_700-MB.png]

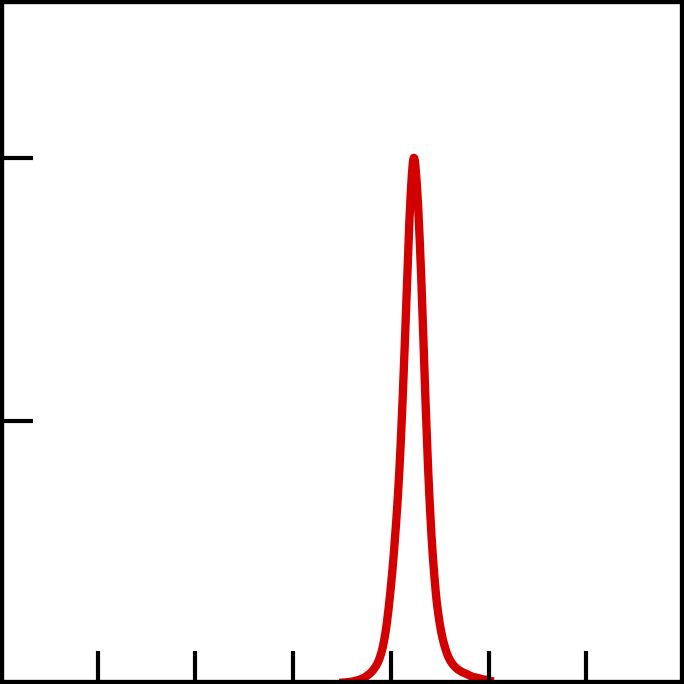

Supplement: Supplementary file 13 — Dataset EV5 [file MSB-13-926-s013.zip › dataset_ev5_pcs_spline_construction/plots/sc_led_spectra_720-MB.png]

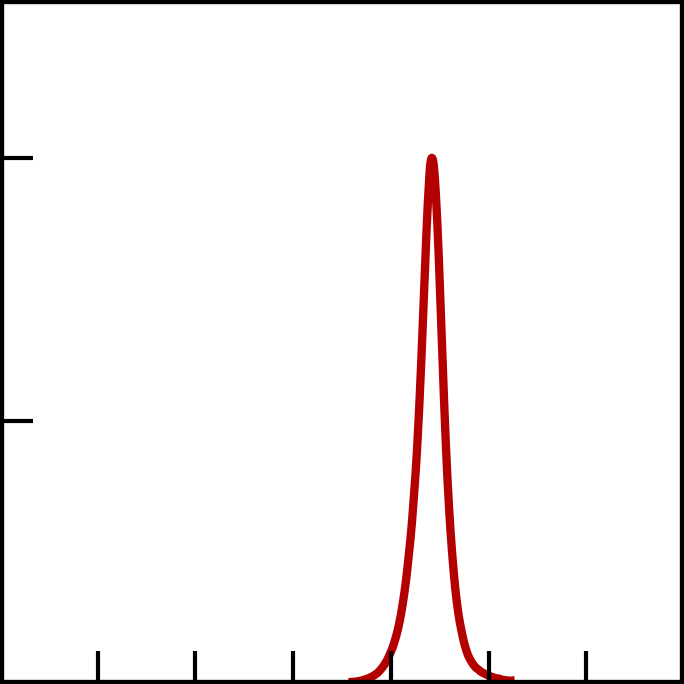

Supplement: Supplementary file 13 — Dataset EV5 [file MSB-13-926-s013.zip › dataset_ev5_pcs_spline_construction/plots/sc_led_spectra_740-MT.png]

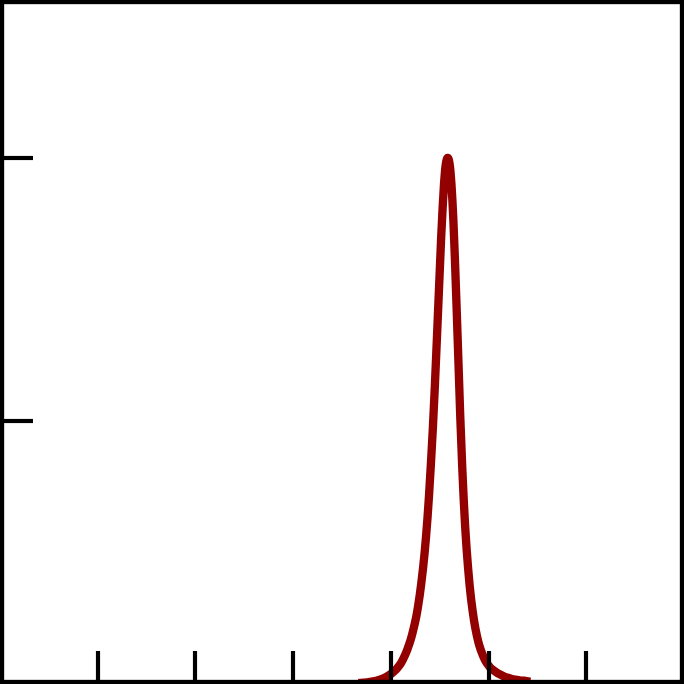

Supplement: Supplementary file 13 — Dataset EV5 [file MSB-13-926-s013.zip › dataset_ev5_pcs_spline_construction/plots/sc_led_spectra_760-MB.png]

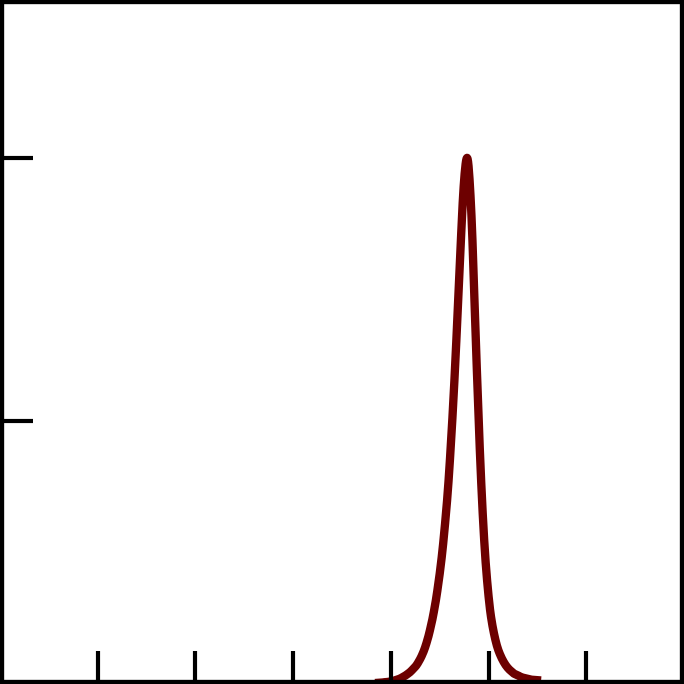

Supplement: Supplementary file 13 — Dataset EV5 [file MSB-13-926-s013.zip › dataset_ev5_pcs_spline_construction/plots/sc_led_spectra_780-MB.png]

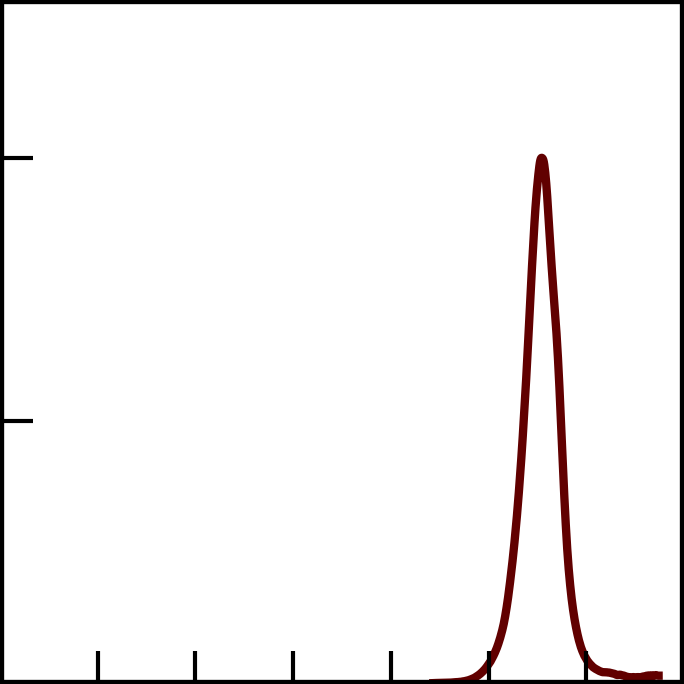

Supplement: Supplementary file 13 — Dataset EV5 [file MSB-13-926-s013.zip › dataset_ev5_pcs_spline_construction/plots/sc_led_spectra_850-VI.png]

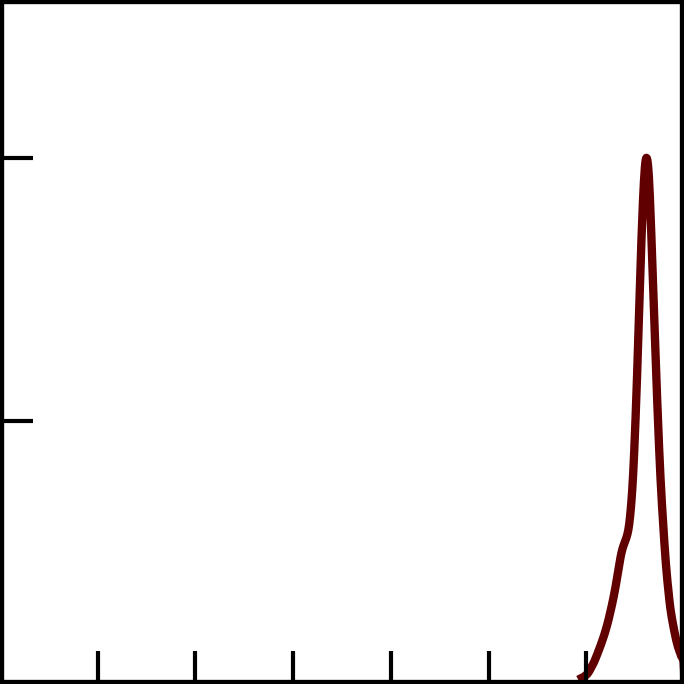

Supplement: Supplementary file 13 — Dataset EV5 [file MSB-13-926-s013.zip › dataset_ev5_pcs_spline_construction/plots/sc_led_spectra_940-VI.png]

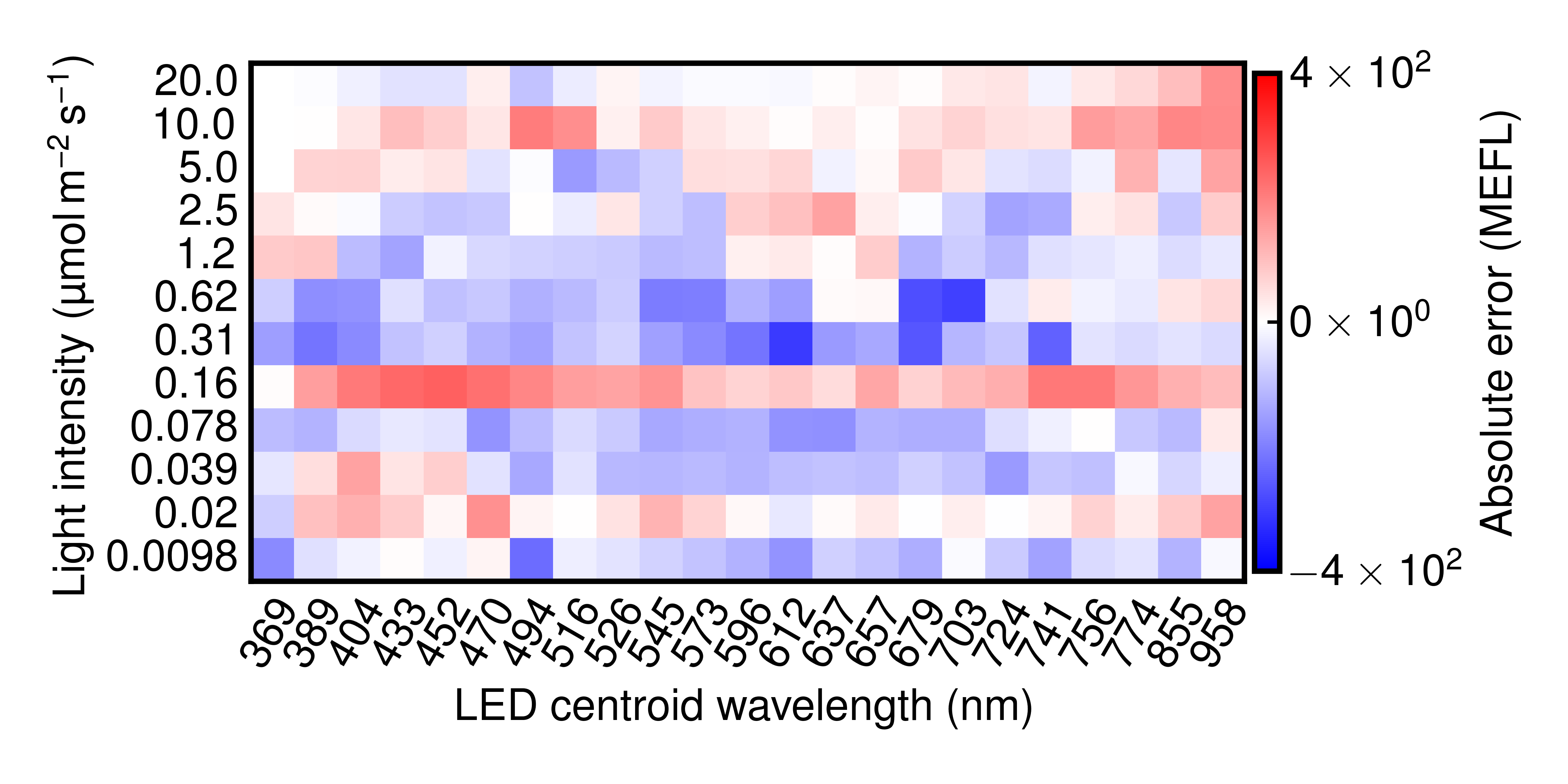

Supplement: Supplementary file 15 — Dataset EV7 [file MSB-13-926-s015.zip › dataset_ev7_cph8-ompr_data_and_analysis/cph8-ompr_analysis/plots/aas_abs_residual_heatmap.png]

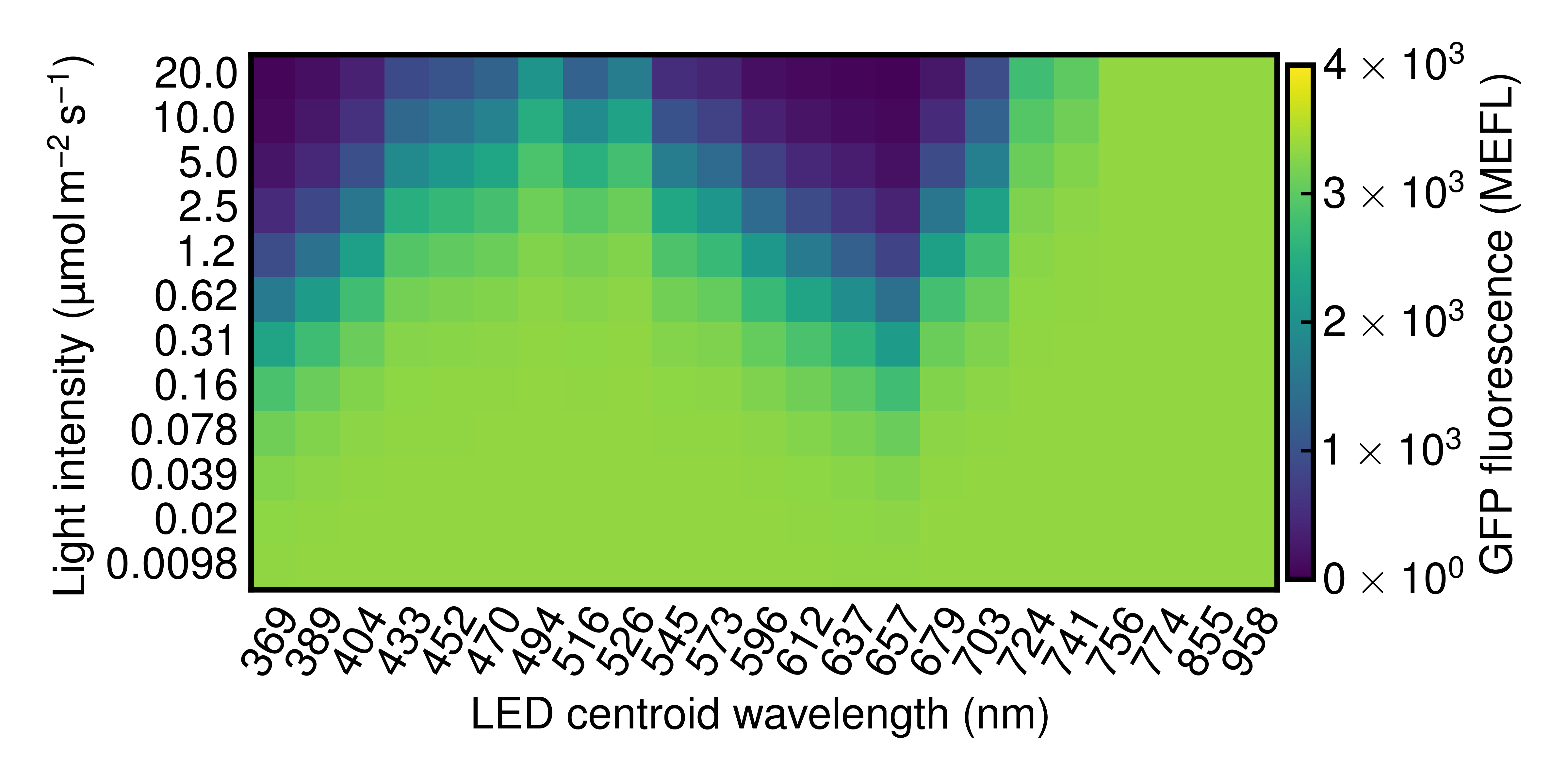

Supplement: Supplementary file 15 — Dataset EV7 [file MSB-13-926-s015.zip › dataset_ev7_cph8-ompr_data_and_analysis/cph8-ompr_analysis/plots/aas_lin_model_heatmap.png]

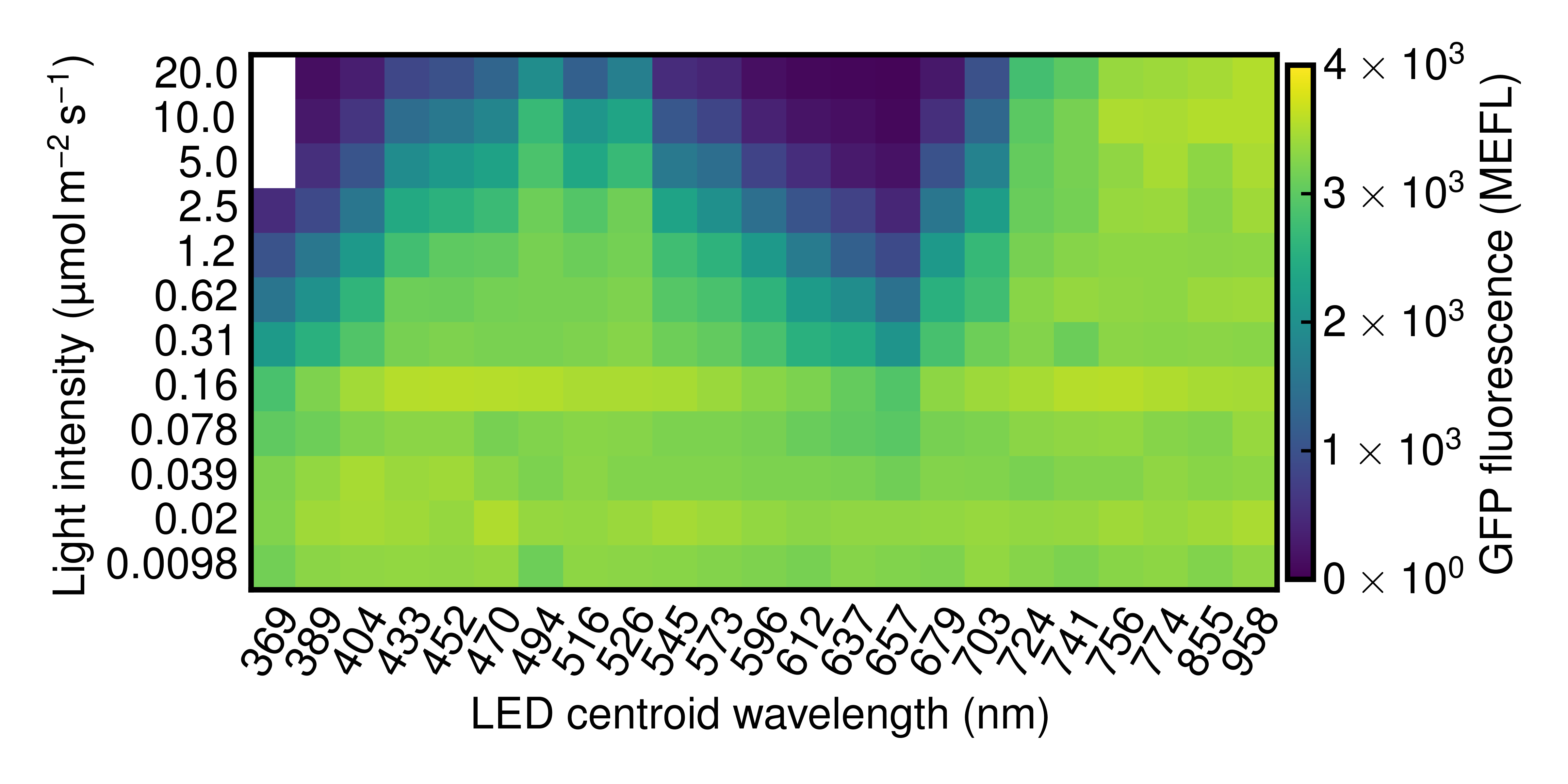

Supplement: Supplementary file 15 — Dataset EV7 [file MSB-13-926-s015.zip › dataset_ev7_cph8-ompr_data_and_analysis/cph8-ompr_analysis/plots/aas_lin_raw_heatmap.png]

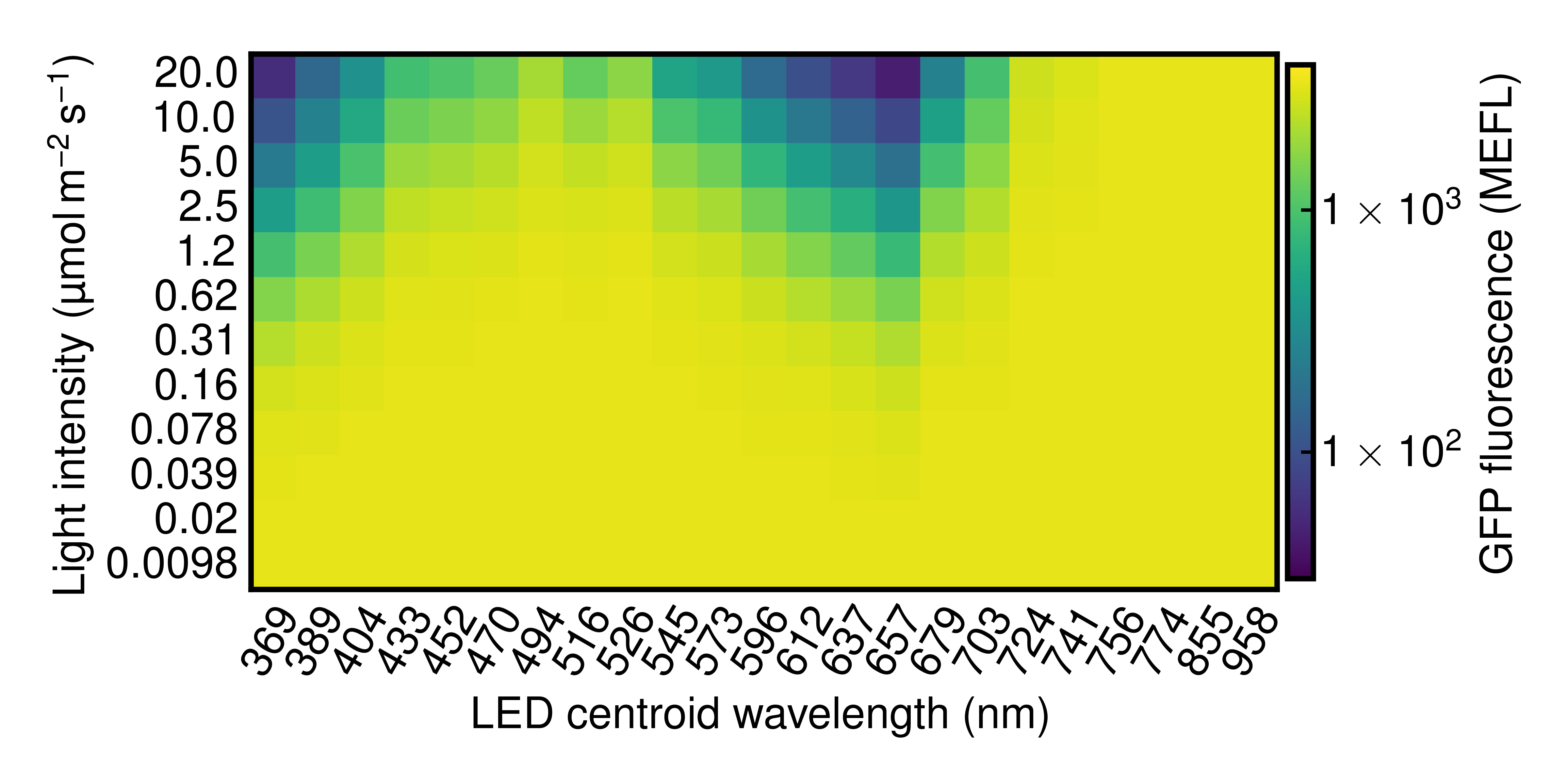

Supplement: Supplementary file 15 — Dataset EV7 [file MSB-13-926-s015.zip › dataset_ev7_cph8-ompr_data_and_analysis/cph8-ompr_analysis/plots/aas_logz_model_heatmap.png]

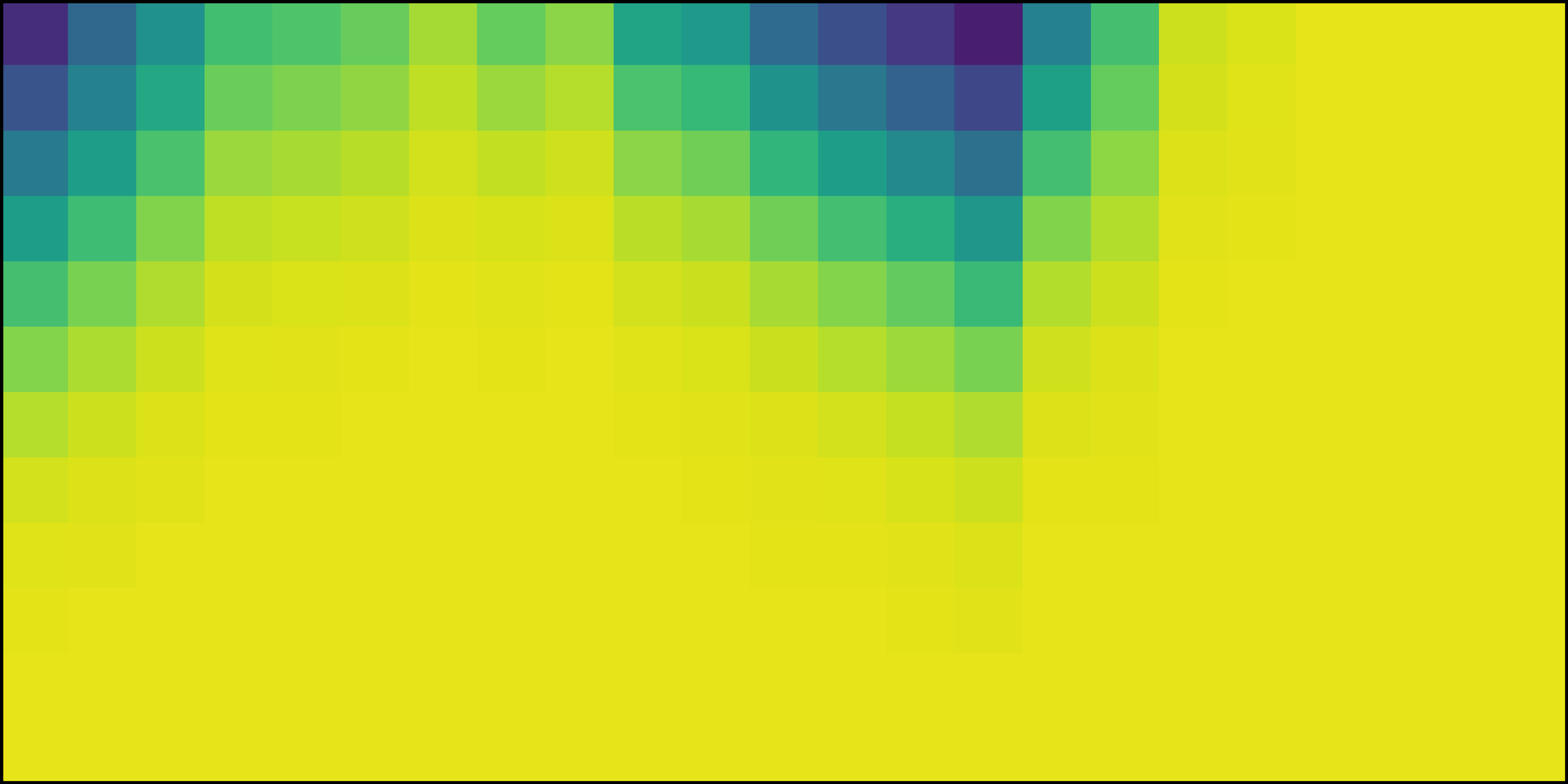

Supplement: Supplementary file 15 — Dataset EV7 [file MSB-13-926-s015.zip › dataset_ev7_cph8-ompr_data_and_analysis/cph8-ompr_analysis/plots/aas_logz_model_nolabel_heatmap.png]

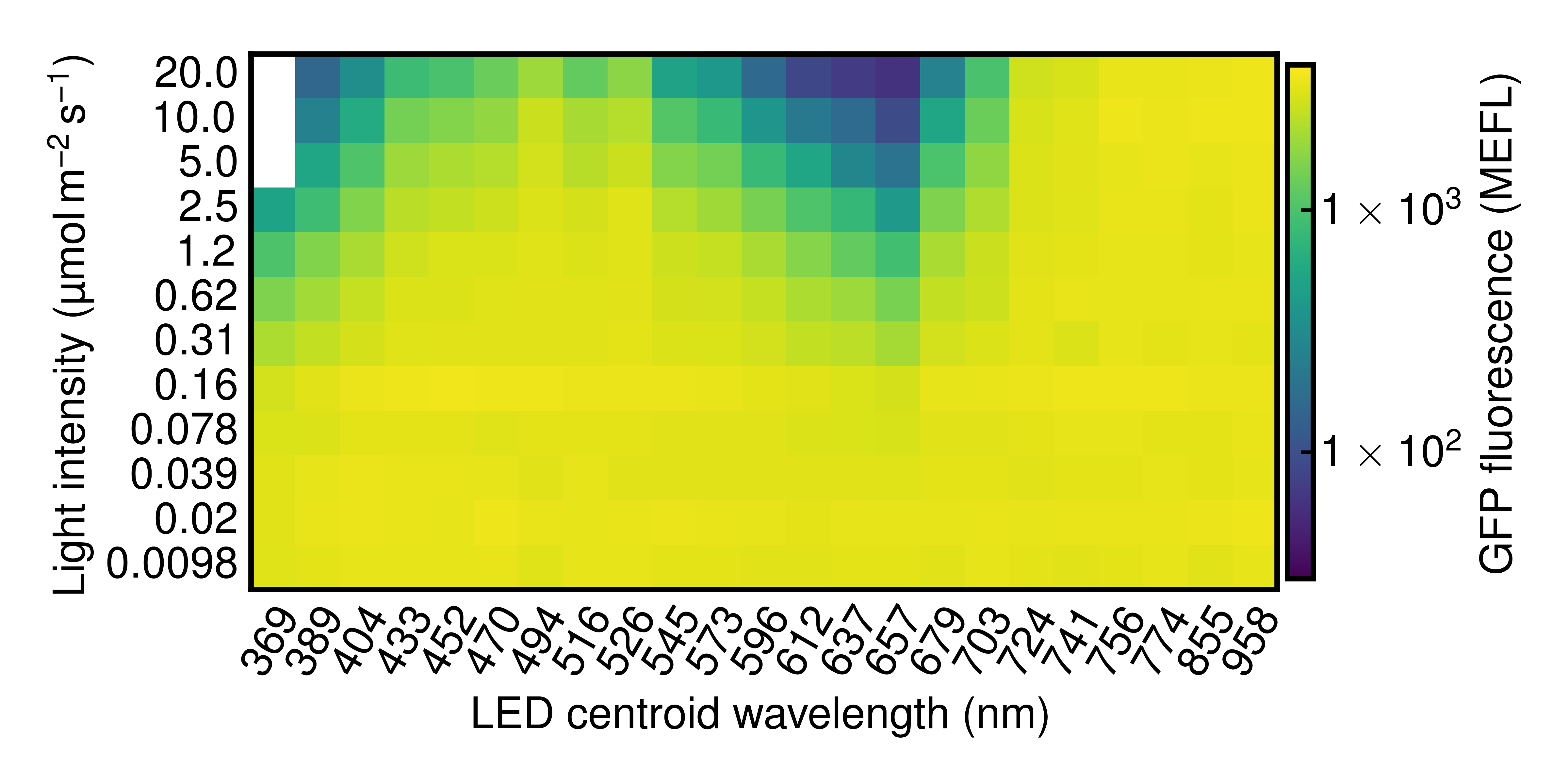

Supplement: Supplementary file 15 — Dataset EV7 [file MSB-13-926-s015.zip › dataset_ev7_cph8-ompr_data_and_analysis/cph8-ompr_analysis/plots/aas_logz_raw_heatmap.png]

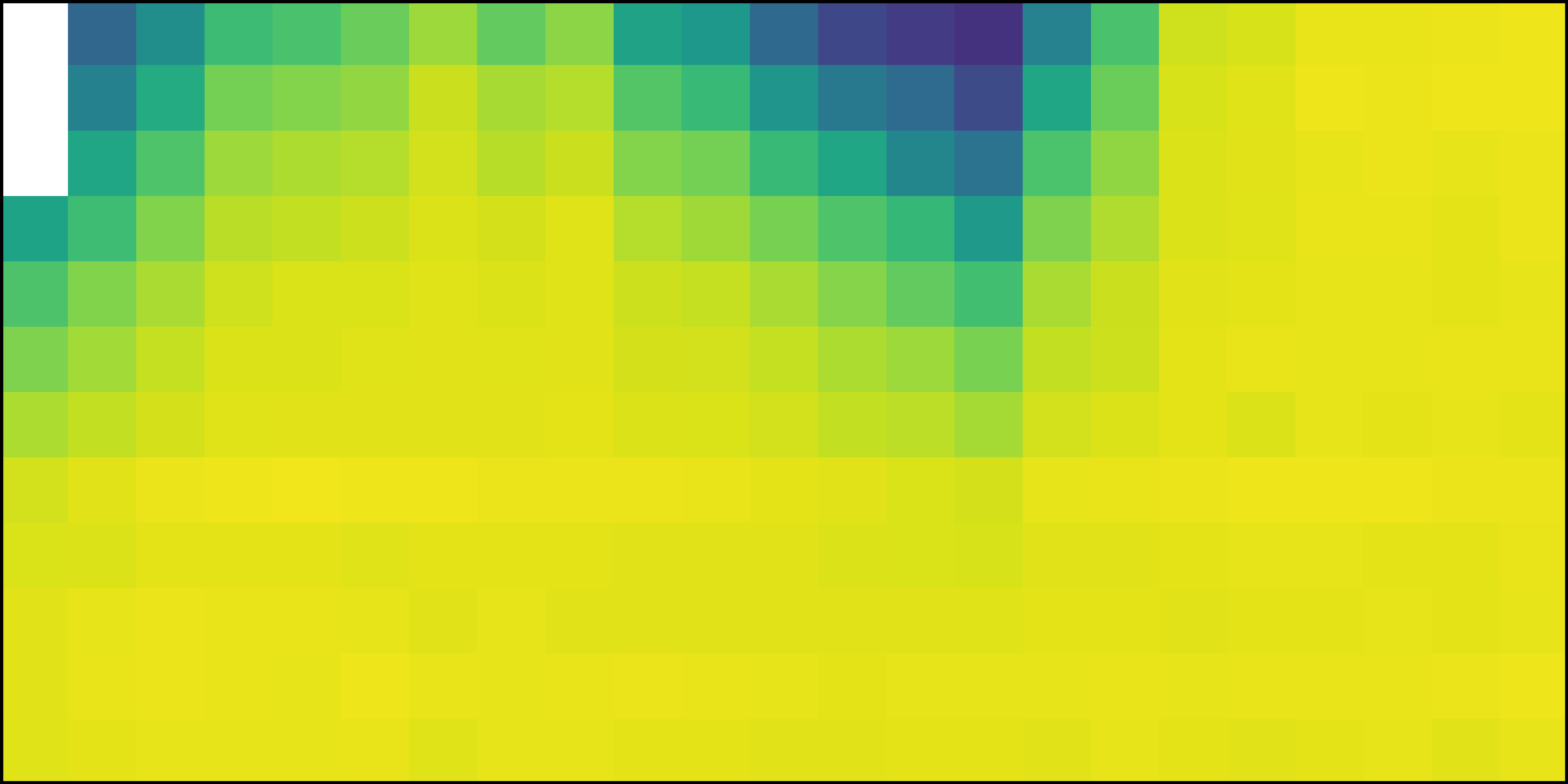

Supplement: Supplementary file 15 — Dataset EV7 [file MSB-13-926-s015.zip › dataset_ev7_cph8-ompr_data_and_analysis/cph8-ompr_analysis/plots/aas_logz_raw_nolabel_heatmap.png]

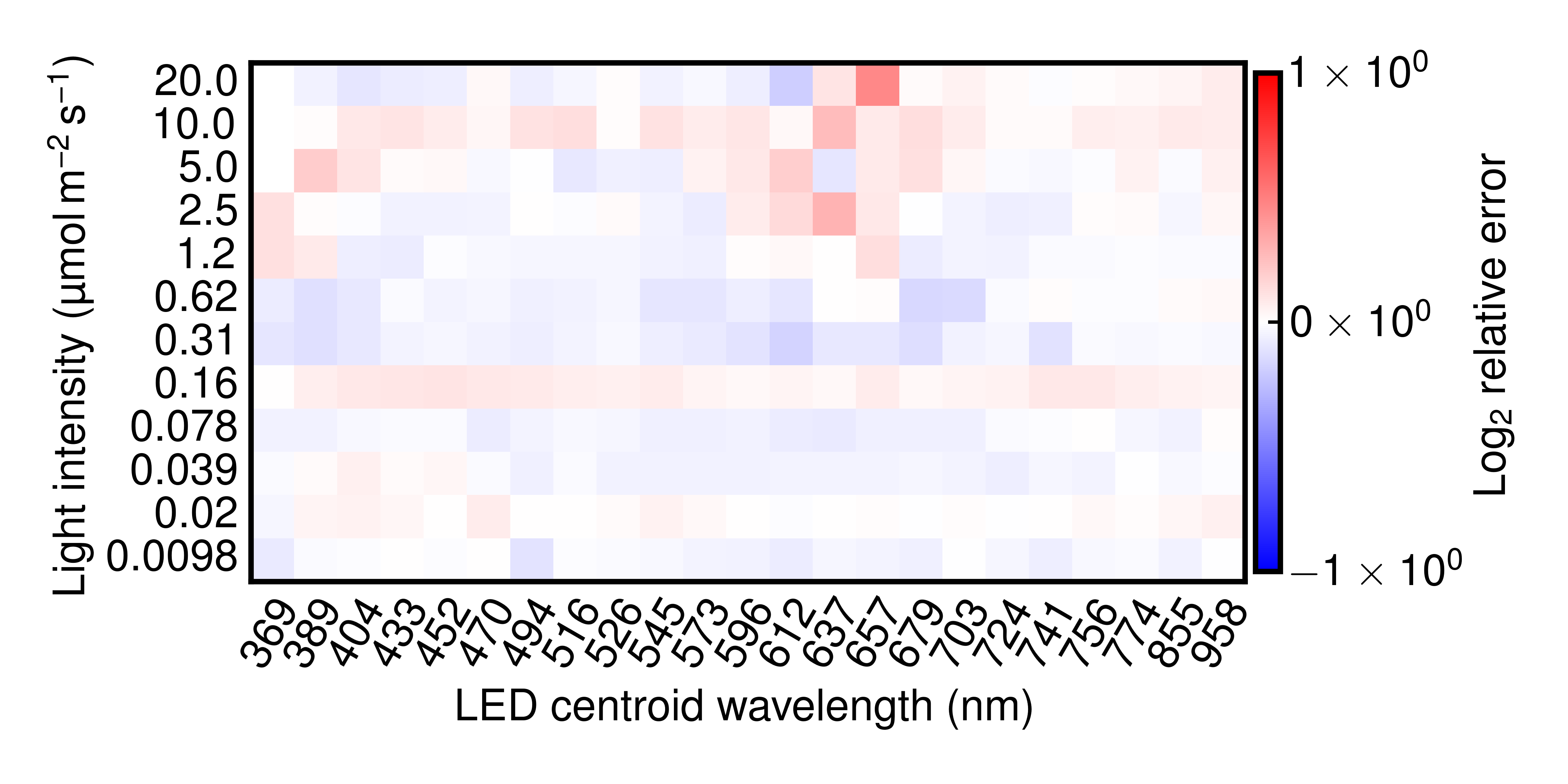

Supplement: Supplementary file 15 — Dataset EV7 [file MSB-13-926-s015.zip › dataset_ev7_cph8-ompr_data_and_analysis/cph8-ompr_analysis/plots/aas_rel_residual_heatmap.png]

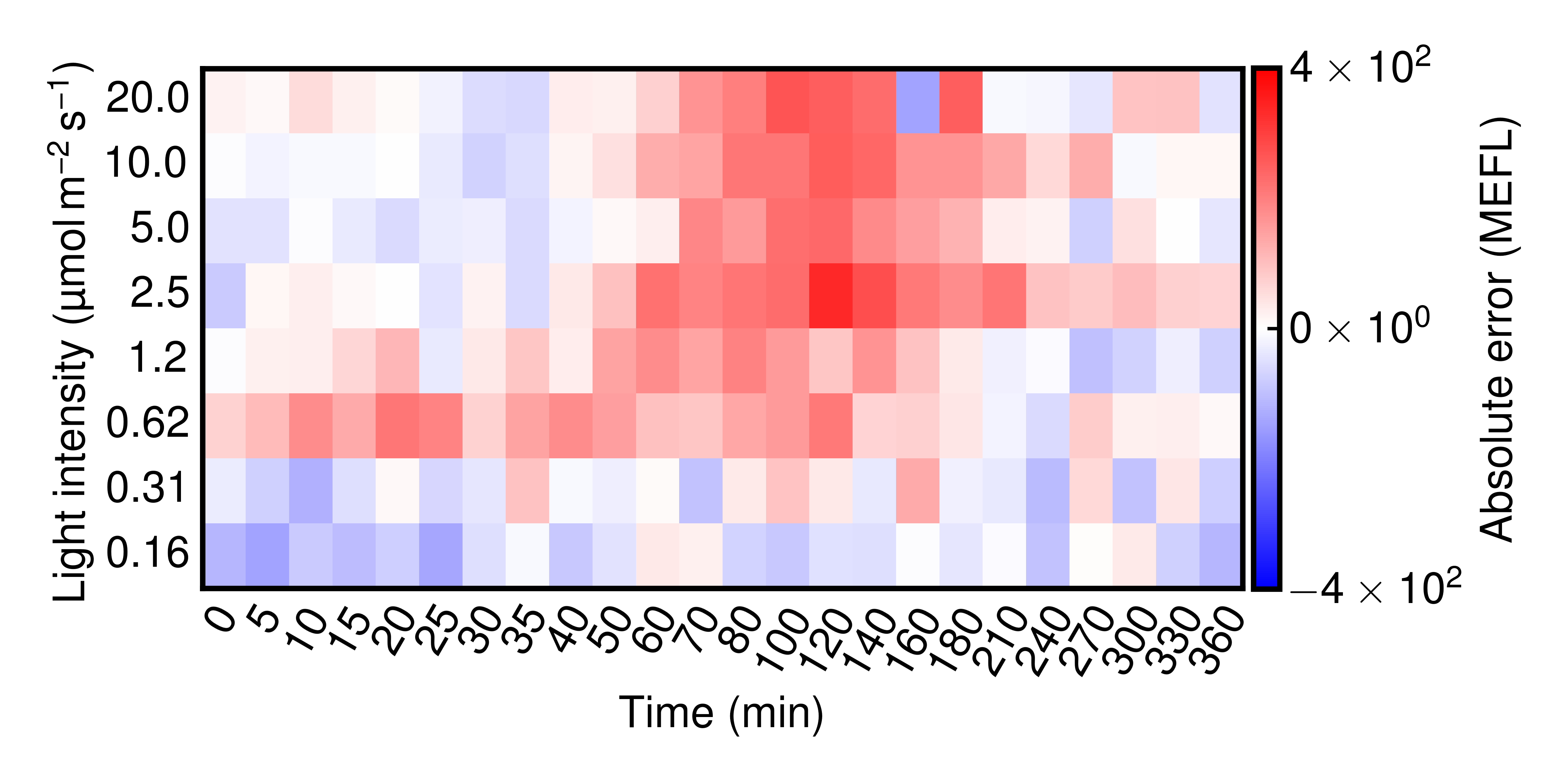

Supplement: Supplementary file 15 — Dataset EV7 [file MSB-13-926-s015.zip › dataset_ev7_cph8-ompr_data_and_analysis/cph8-ompr_analysis/plots/atd_abs_residual_hmap.png]

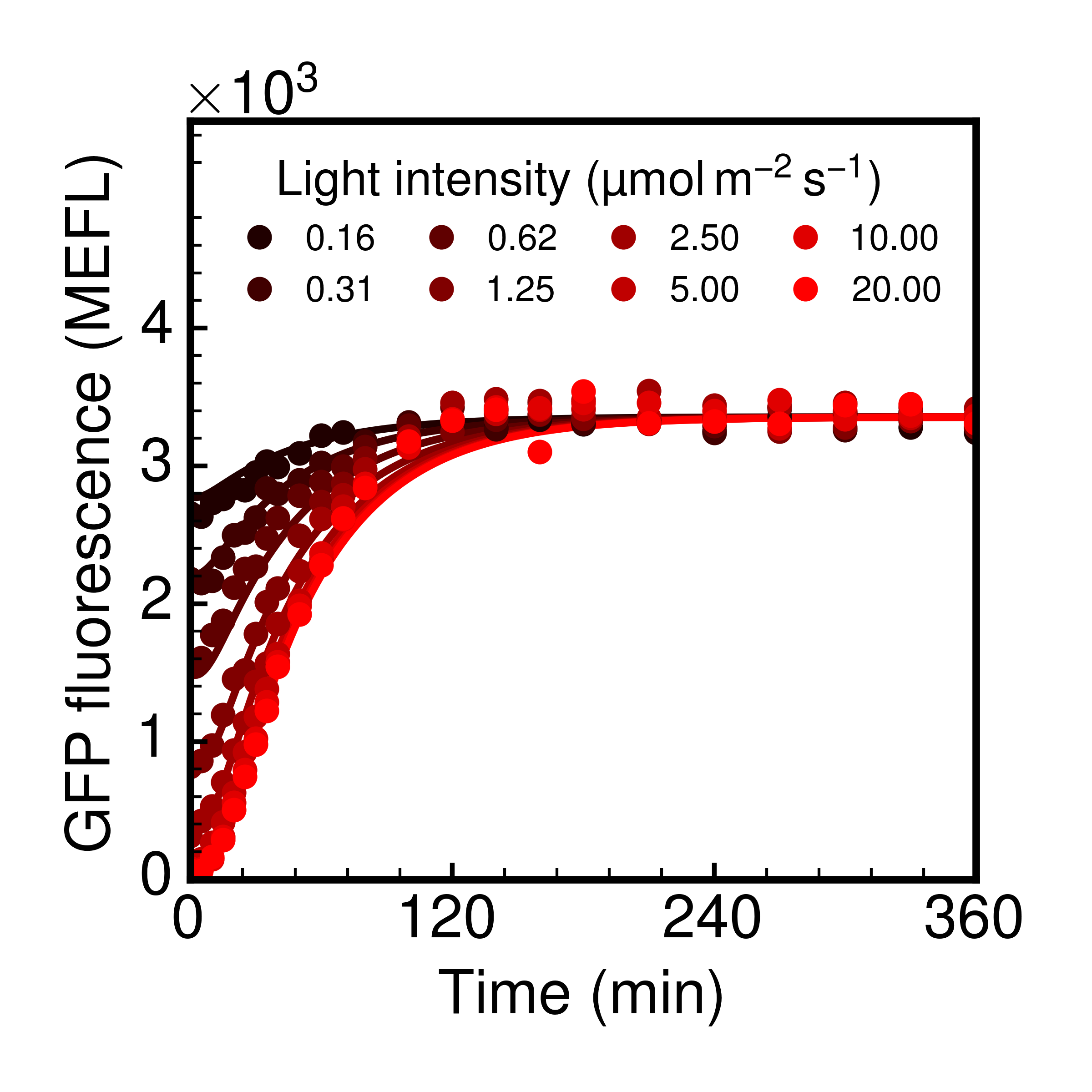

Supplement: Supplementary file 15 — Dataset EV7 [file MSB-13-926-s015.zip › dataset_ev7_cph8-ompr_data_and_analysis/cph8-ompr_analysis/plots/atd_lin_model.png]

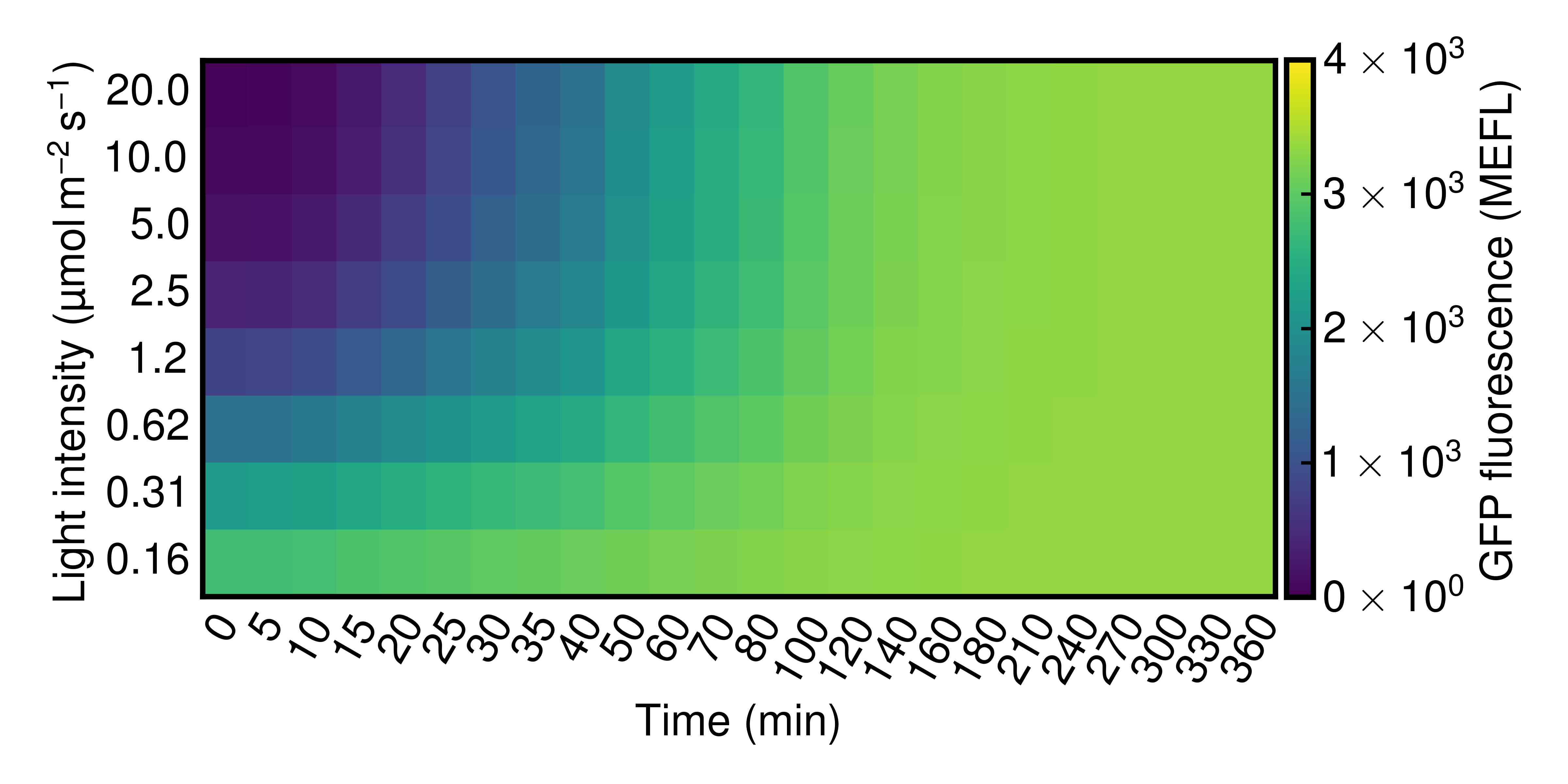

Supplement: Supplementary file 15 — Dataset EV7 [file MSB-13-926-s015.zip › dataset_ev7_cph8-ompr_data_and_analysis/cph8-ompr_analysis/plots/atd_lin_model_hmap.png]

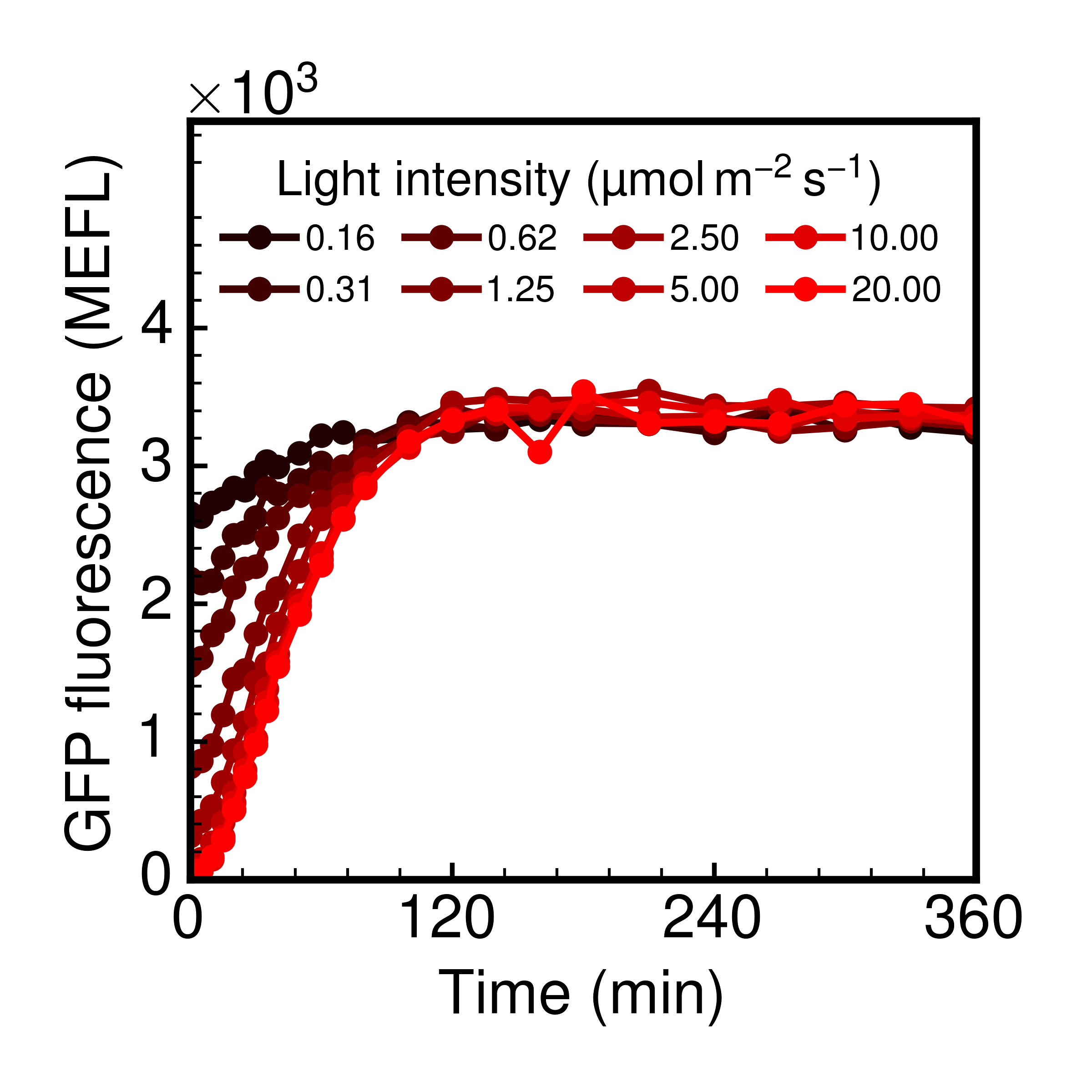

Supplement: Supplementary file 15 — Dataset EV7 [file MSB-13-926-s015.zip › dataset_ev7_cph8-ompr_data_and_analysis/cph8-ompr_analysis/plots/atd_lin_raw.png]

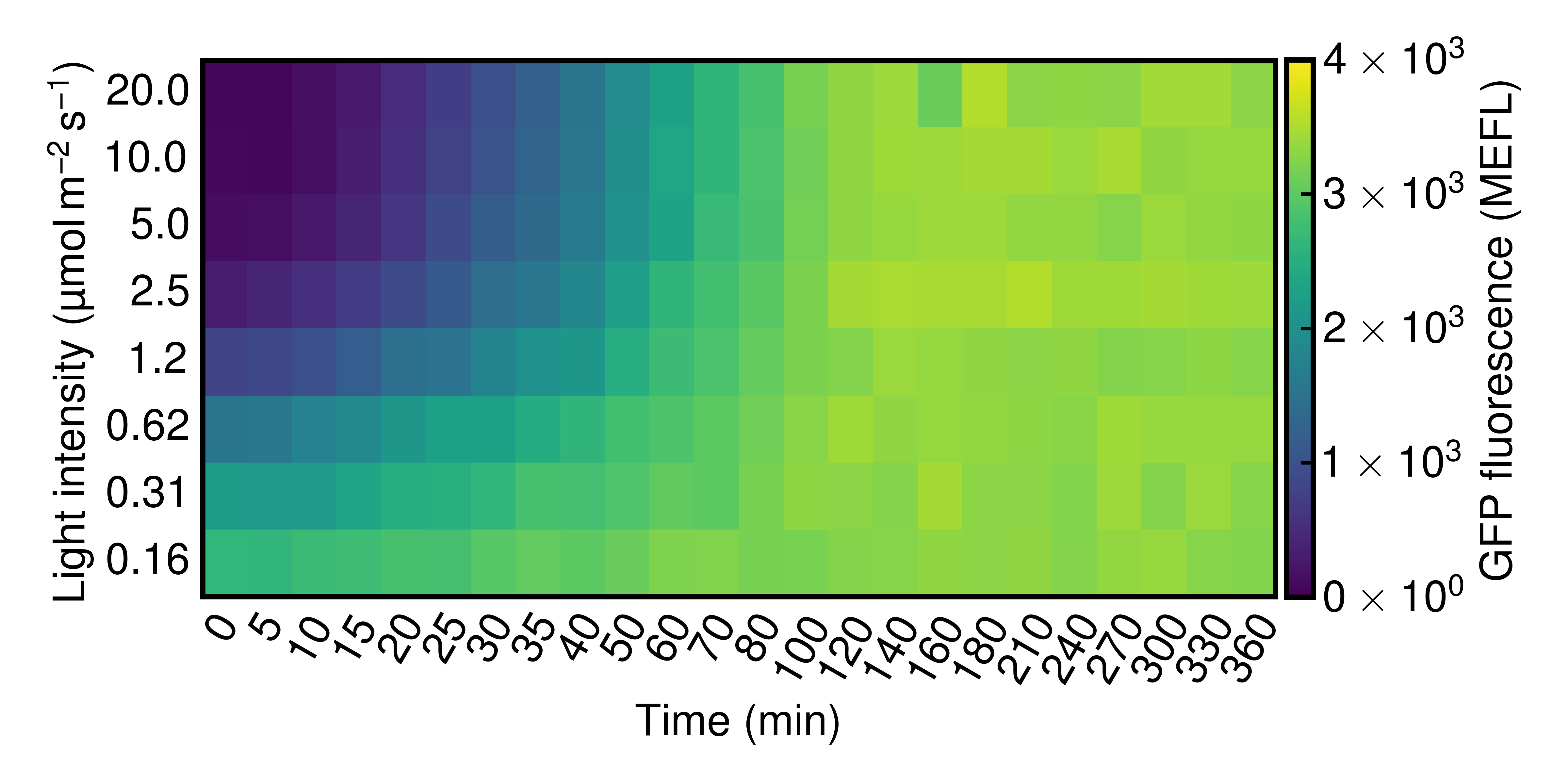

Supplement: Supplementary file 15 — Dataset EV7 [file MSB-13-926-s015.zip › dataset_ev7_cph8-ompr_data_and_analysis/cph8-ompr_analysis/plots/atd_lin_raw_hmap.png]

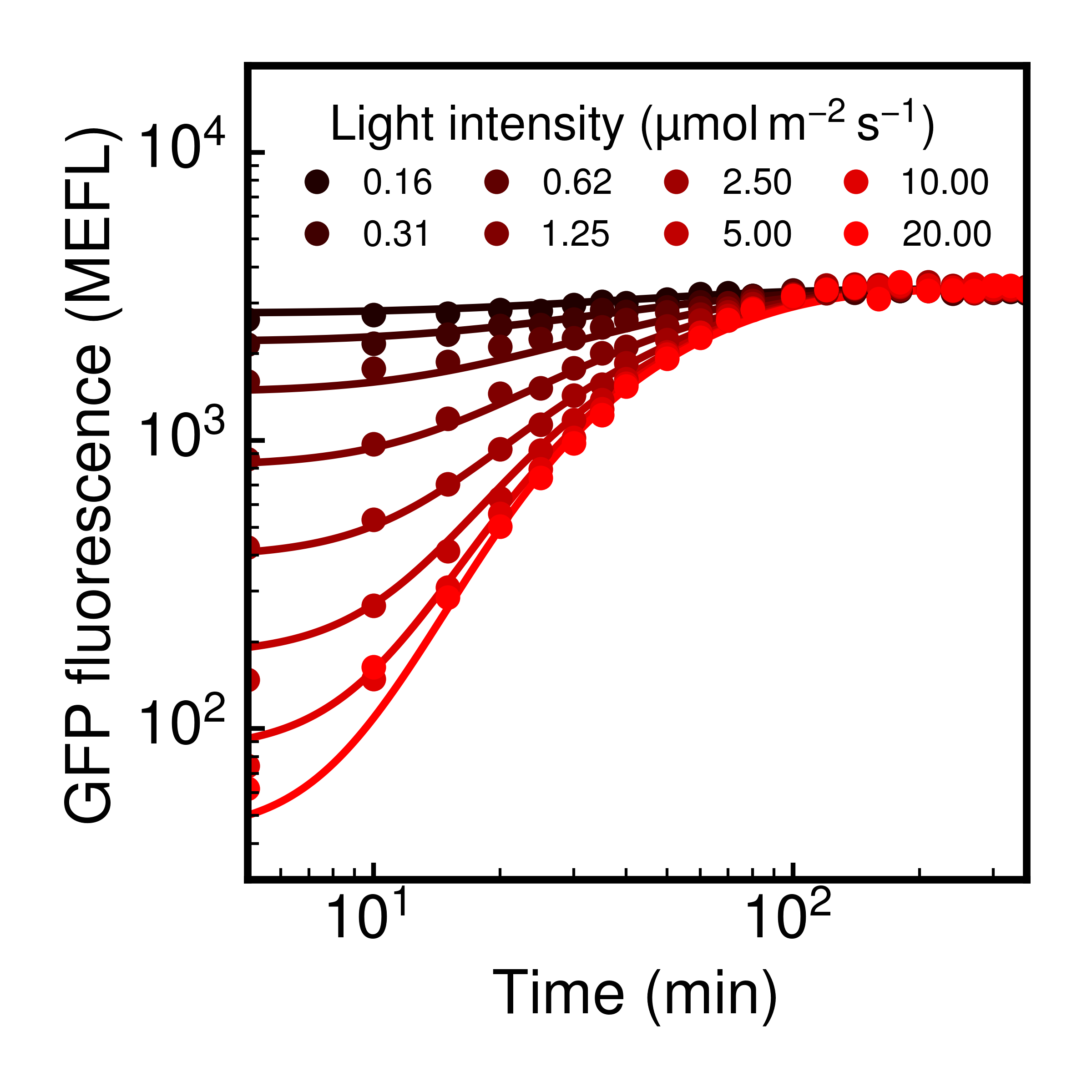

Supplement: Supplementary file 15 — Dataset EV7 [file MSB-13-926-s015.zip › dataset_ev7_cph8-ompr_data_and_analysis/cph8-ompr_analysis/plots/atd_logxy_model.png]

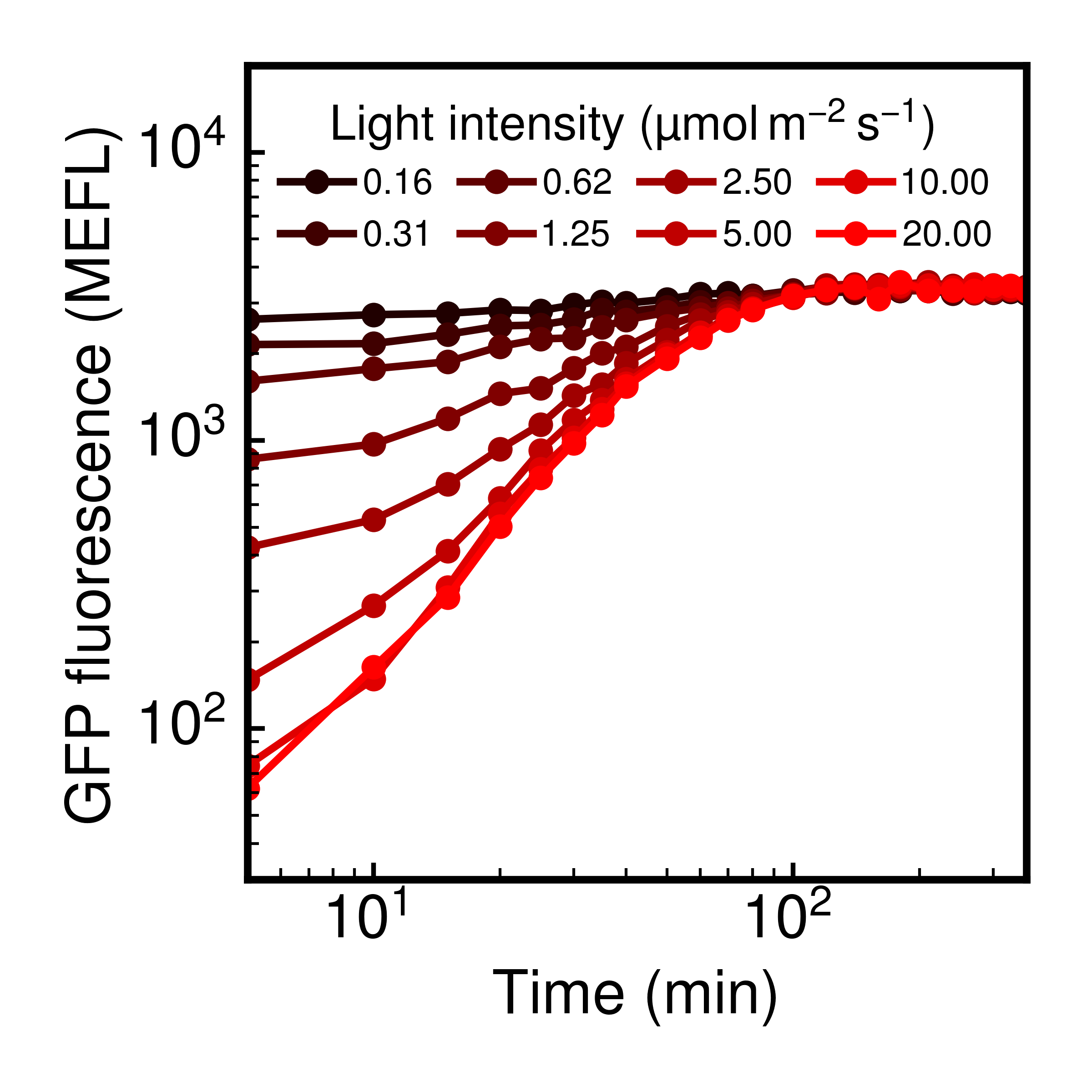

Supplement: Supplementary file 15 — Dataset EV7 [file MSB-13-926-s015.zip › dataset_ev7_cph8-ompr_data_and_analysis/cph8-ompr_analysis/plots/atd_logxy_raw.png]

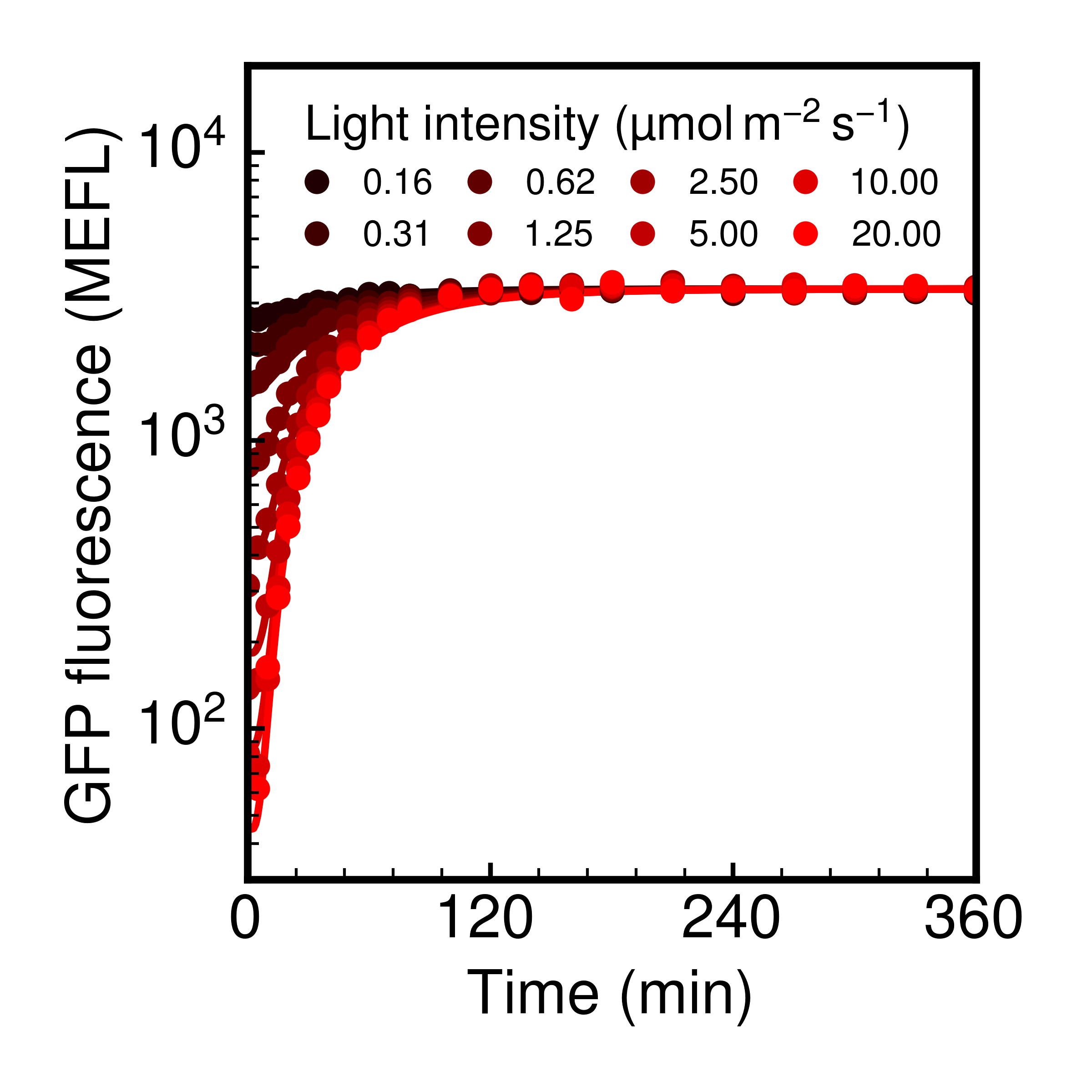

Supplement: Supplementary file 15 — Dataset EV7 [file MSB-13-926-s015.zip › dataset_ev7_cph8-ompr_data_and_analysis/cph8-ompr_analysis/plots/atd_logy_model.png]

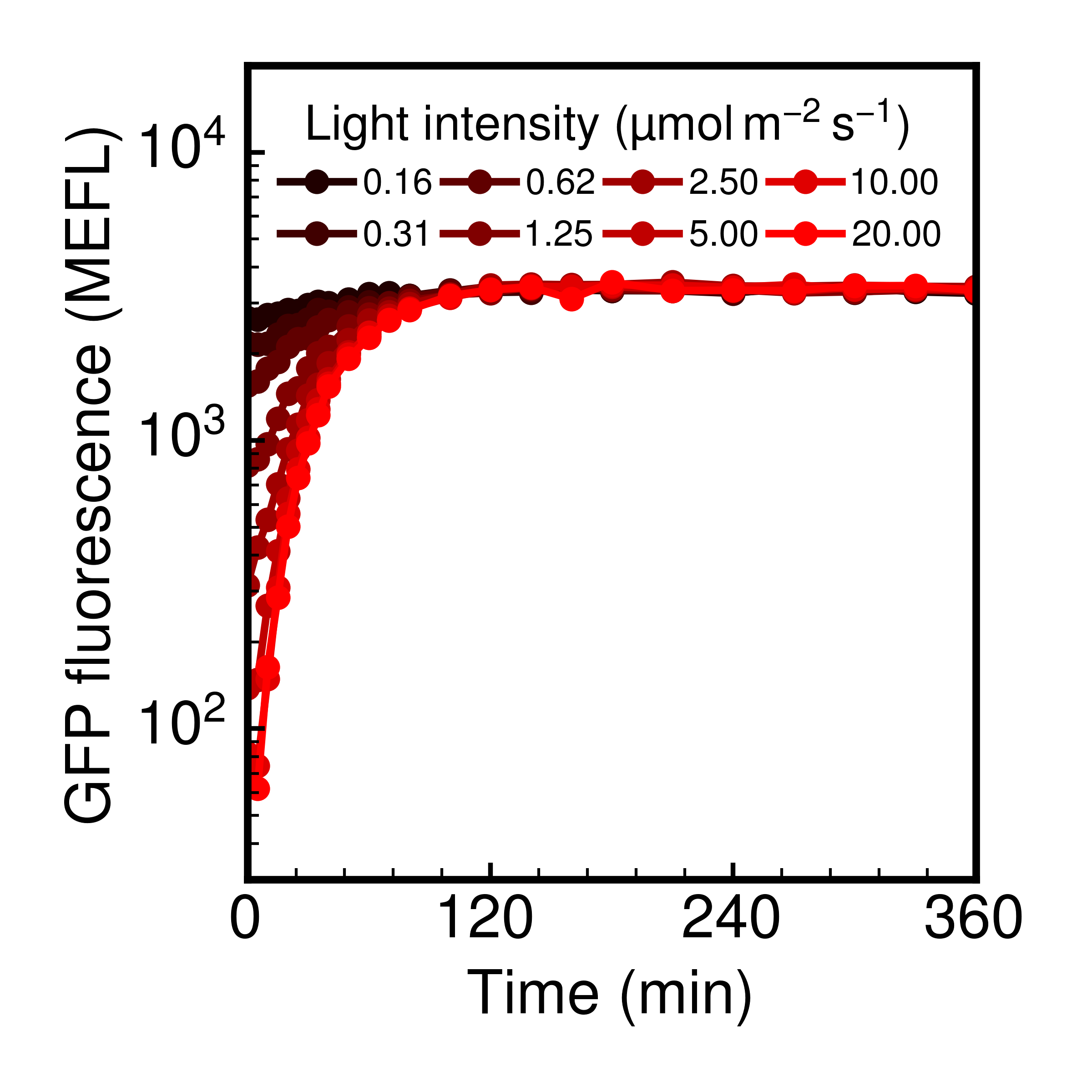

Supplement: Supplementary file 15 — Dataset EV7 [file MSB-13-926-s015.zip › dataset_ev7_cph8-ompr_data_and_analysis/cph8-ompr_analysis/plots/atd_logy_raw.png]

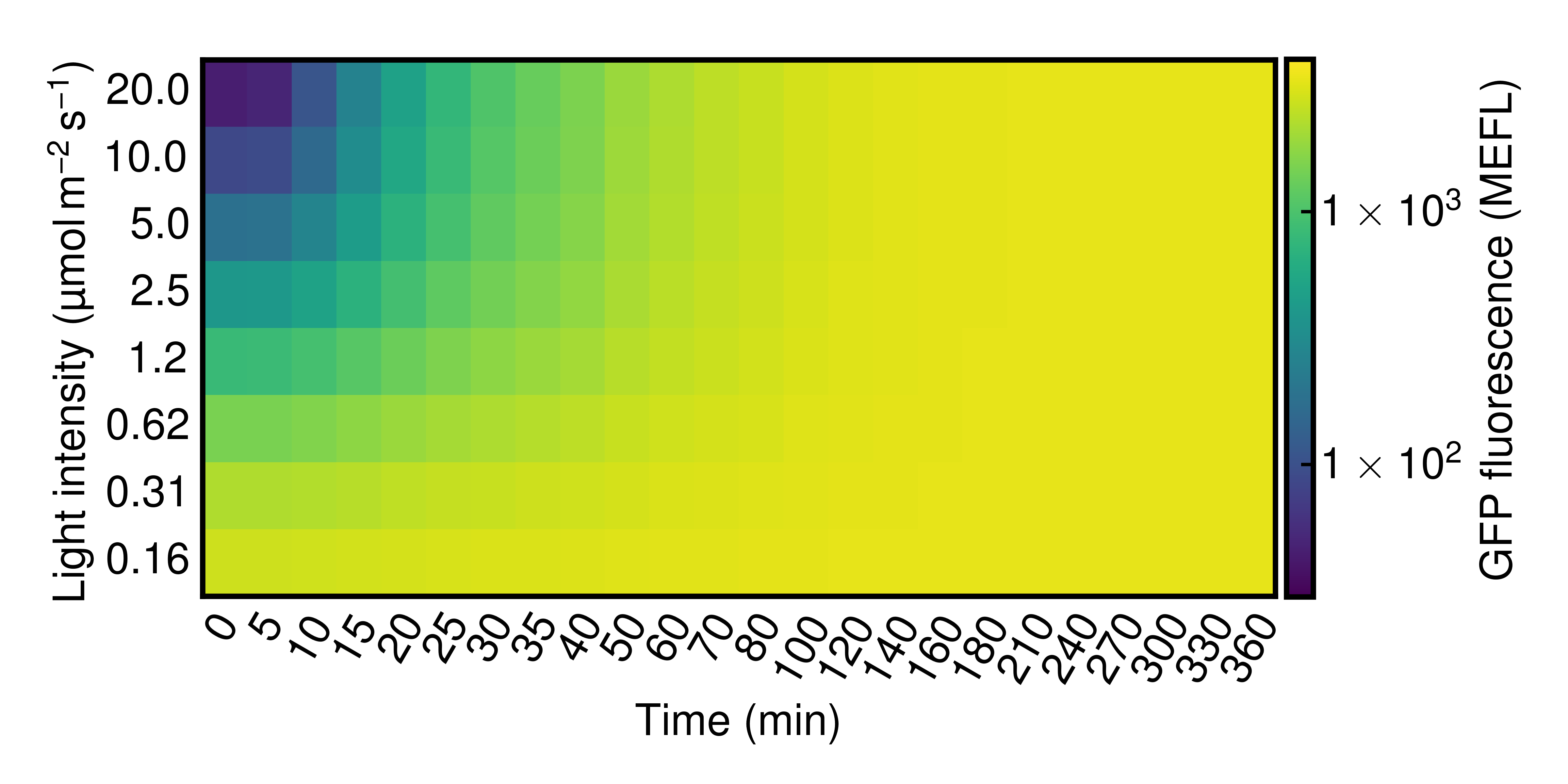

Supplement: Supplementary file 15 — Dataset EV7 [file MSB-13-926-s015.zip › dataset_ev7_cph8-ompr_data_and_analysis/cph8-ompr_analysis/plots/atd_logz_model_hmap.png]

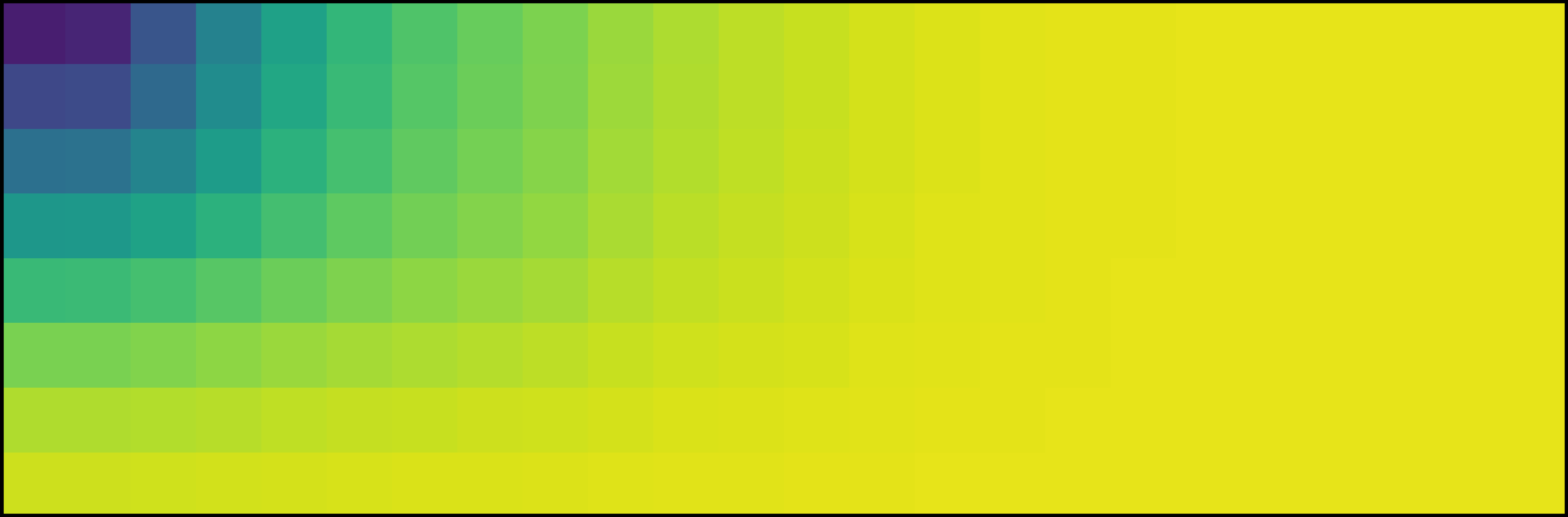

Supplement: Supplementary file 15 — Dataset EV7 [file MSB-13-926-s015.zip › dataset_ev7_cph8-ompr_data_and_analysis/cph8-ompr_analysis/plots/atd_logz_model_nolabel_hmap.png]

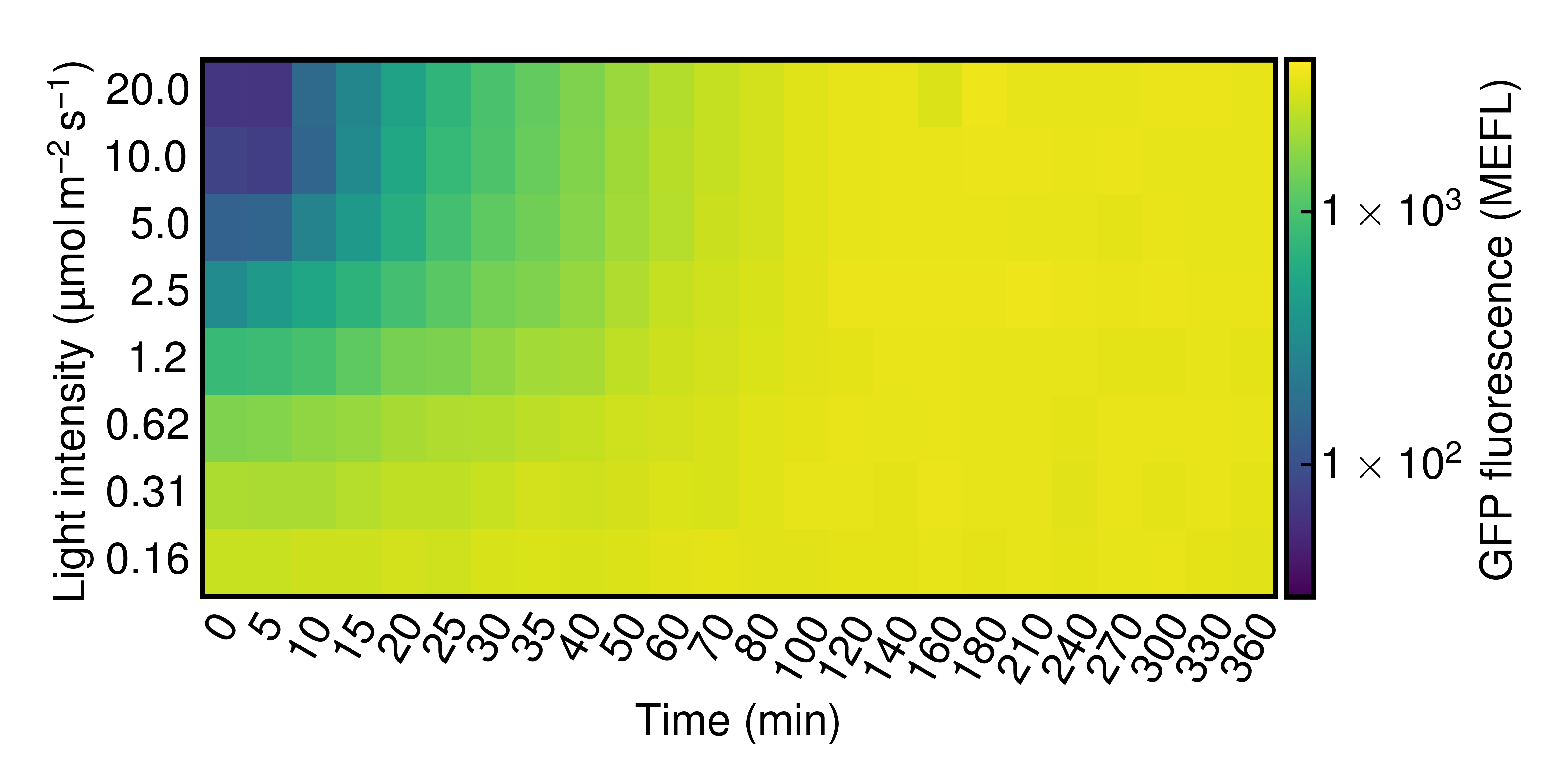

Supplement: Supplementary file 15 — Dataset EV7 [file MSB-13-926-s015.zip › dataset_ev7_cph8-ompr_data_and_analysis/cph8-ompr_analysis/plots/atd_logz_raw_hmap.png]

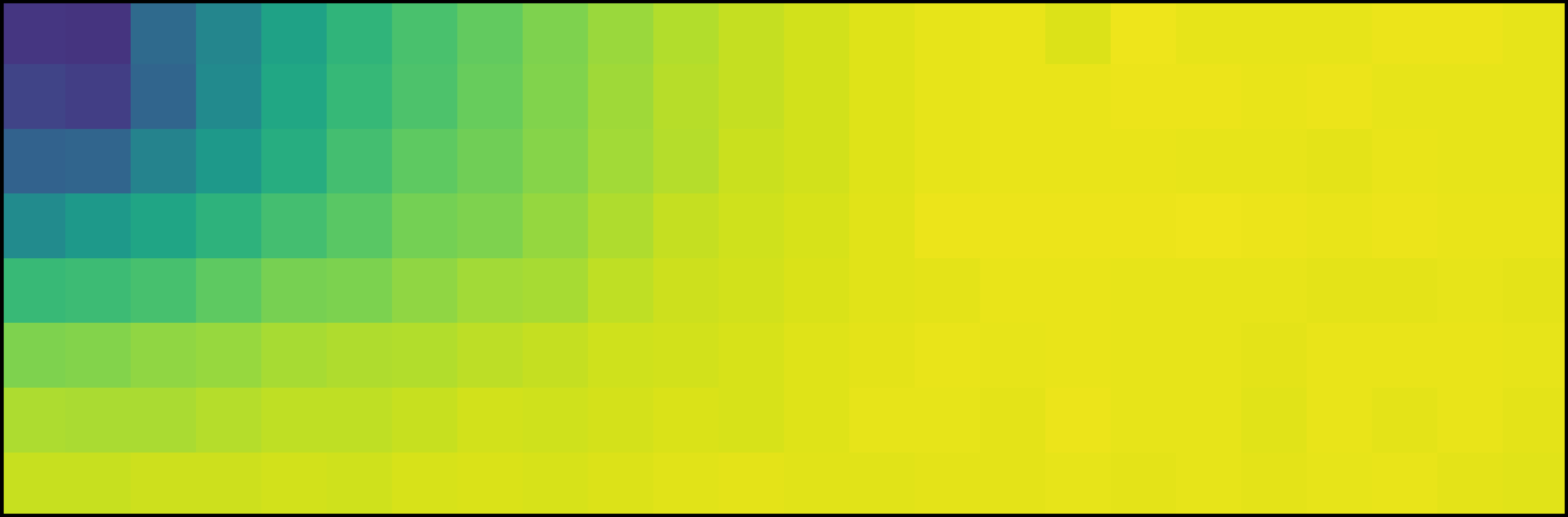

Supplement: Supplementary file 15 — Dataset EV7 [file MSB-13-926-s015.zip › dataset_ev7_cph8-ompr_data_and_analysis/cph8-ompr_analysis/plots/atd_logz_raw_nolabel_hmap.png]

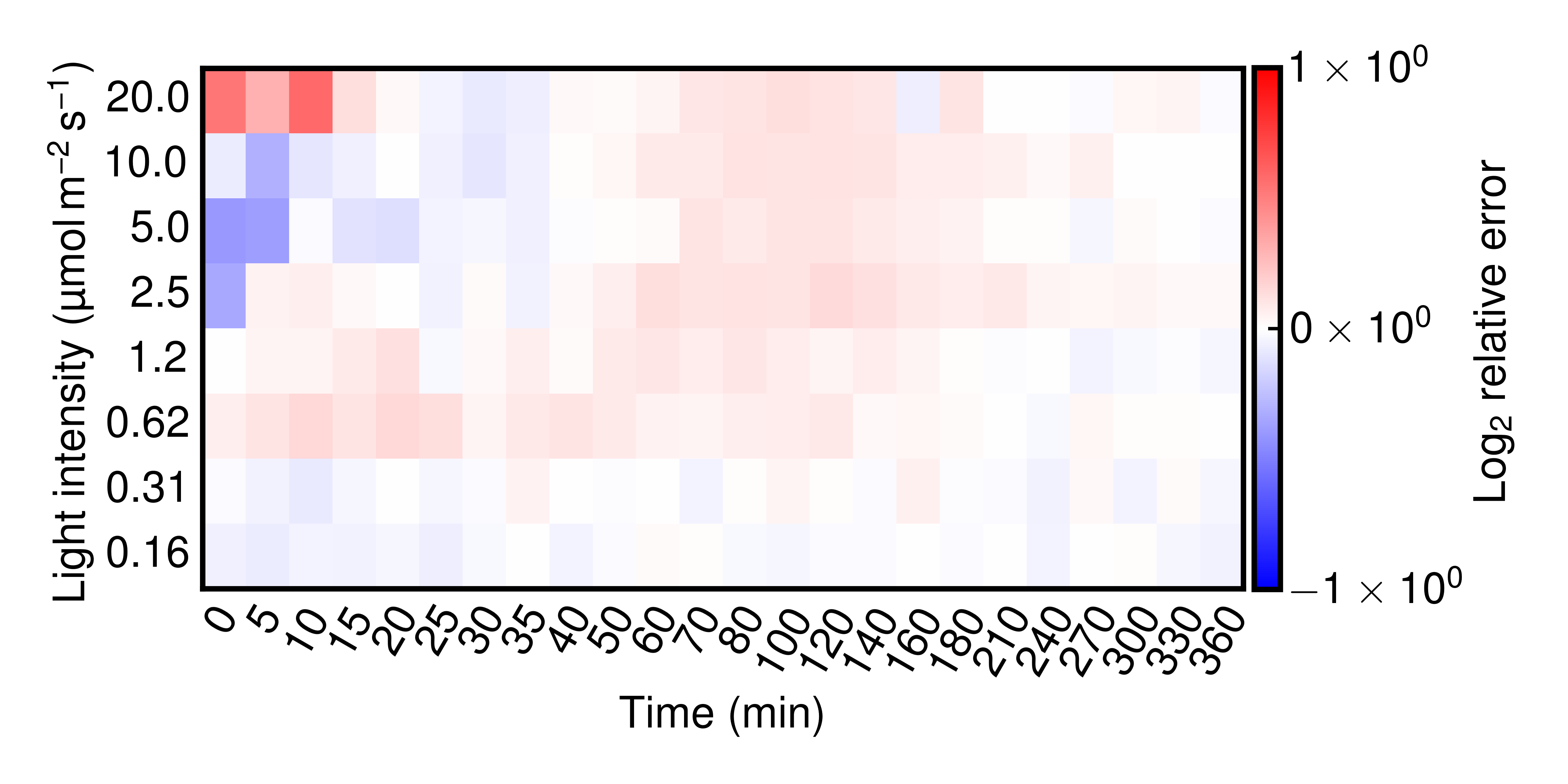

Supplement: Supplementary file 15 — Dataset EV7 [file MSB-13-926-s015.zip › dataset_ev7_cph8-ompr_data_and_analysis/cph8-ompr_analysis/plots/atd_rel_residual_hmap.png]

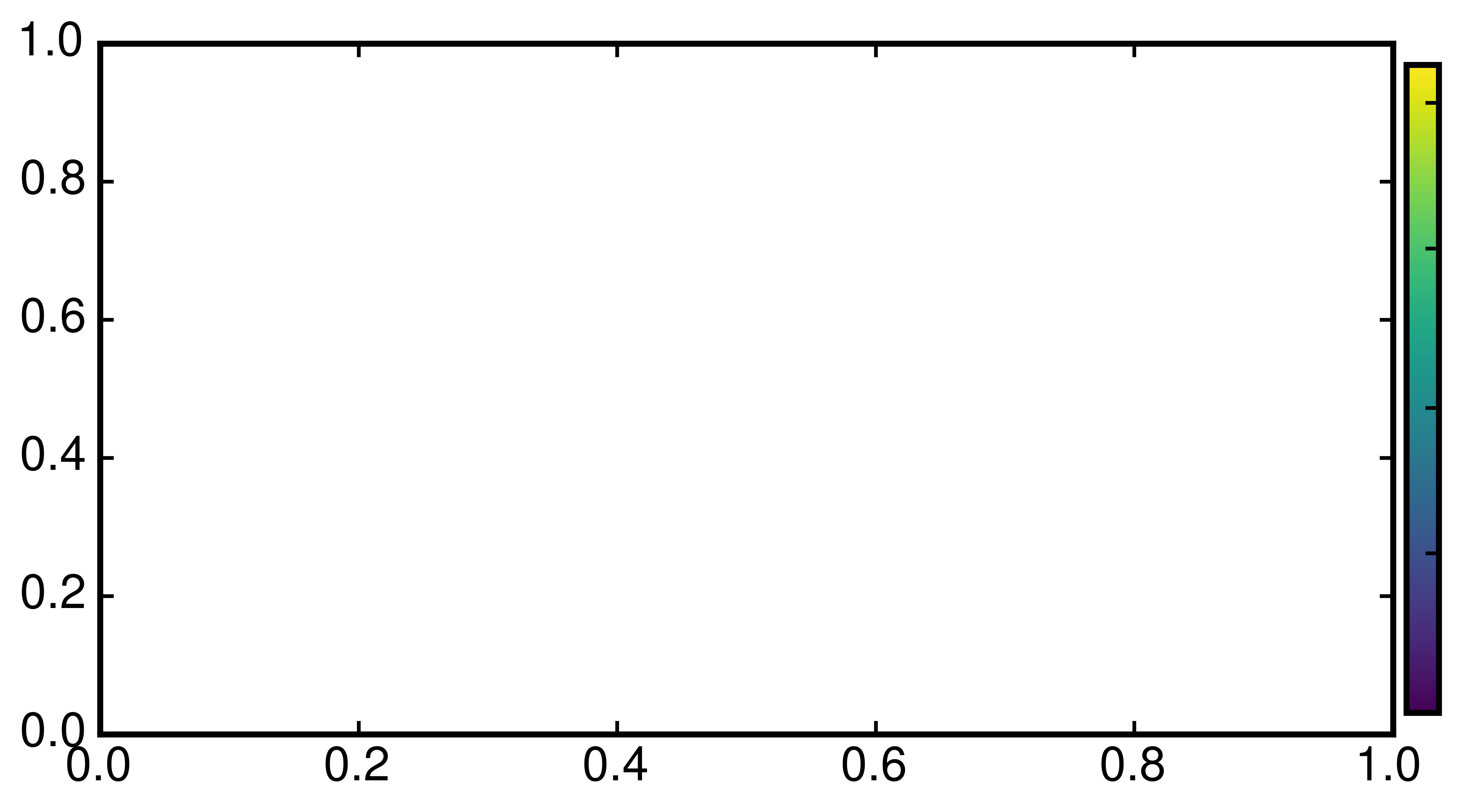

Supplement: Supplementary file 15 — Dataset EV7 [file MSB-13-926-s015.zip › dataset_ev7_cph8-ompr_data_and_analysis/cph8-ompr_analysis/plots/cbar.png]

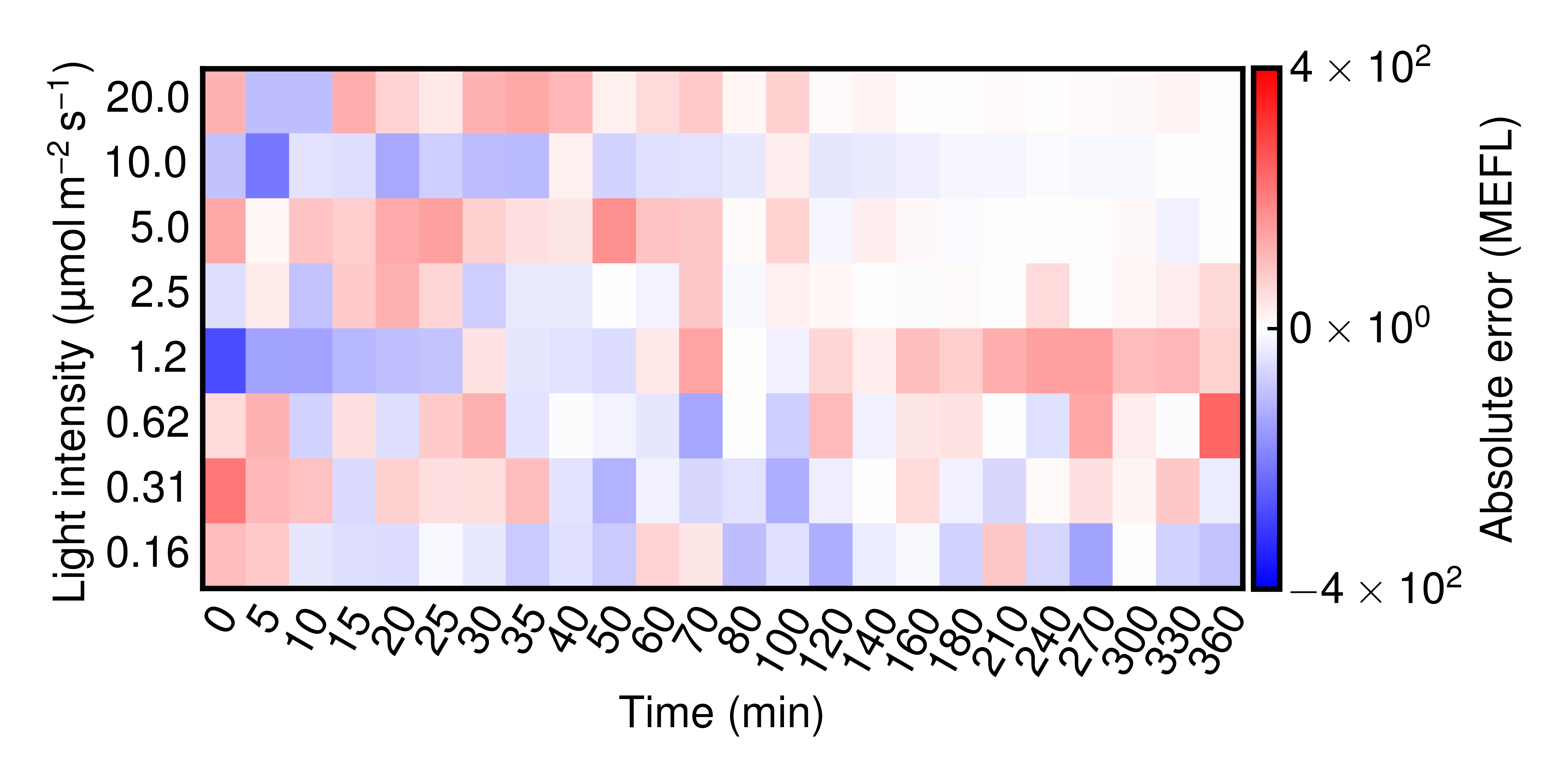

Supplement: Supplementary file 15 — Dataset EV7 [file MSB-13-926-s015.zip › dataset_ev7_cph8-ompr_data_and_analysis/cph8-ompr_analysis/plots/dta_abs_residual_hmap.png]

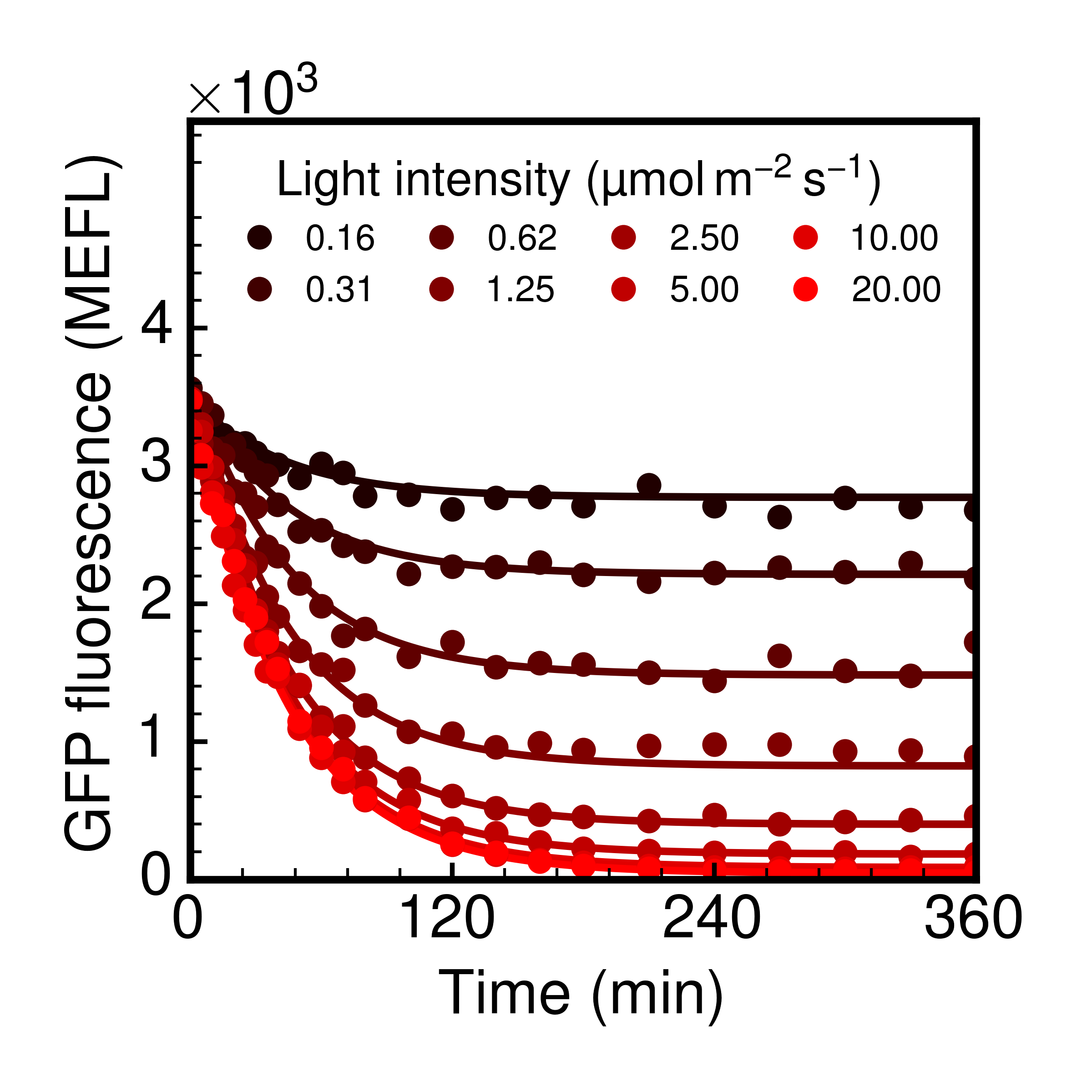

Supplement: Supplementary file 15 — Dataset EV7 [file MSB-13-926-s015.zip › dataset_ev7_cph8-ompr_data_and_analysis/cph8-ompr_analysis/plots/dta_lin_model.png]

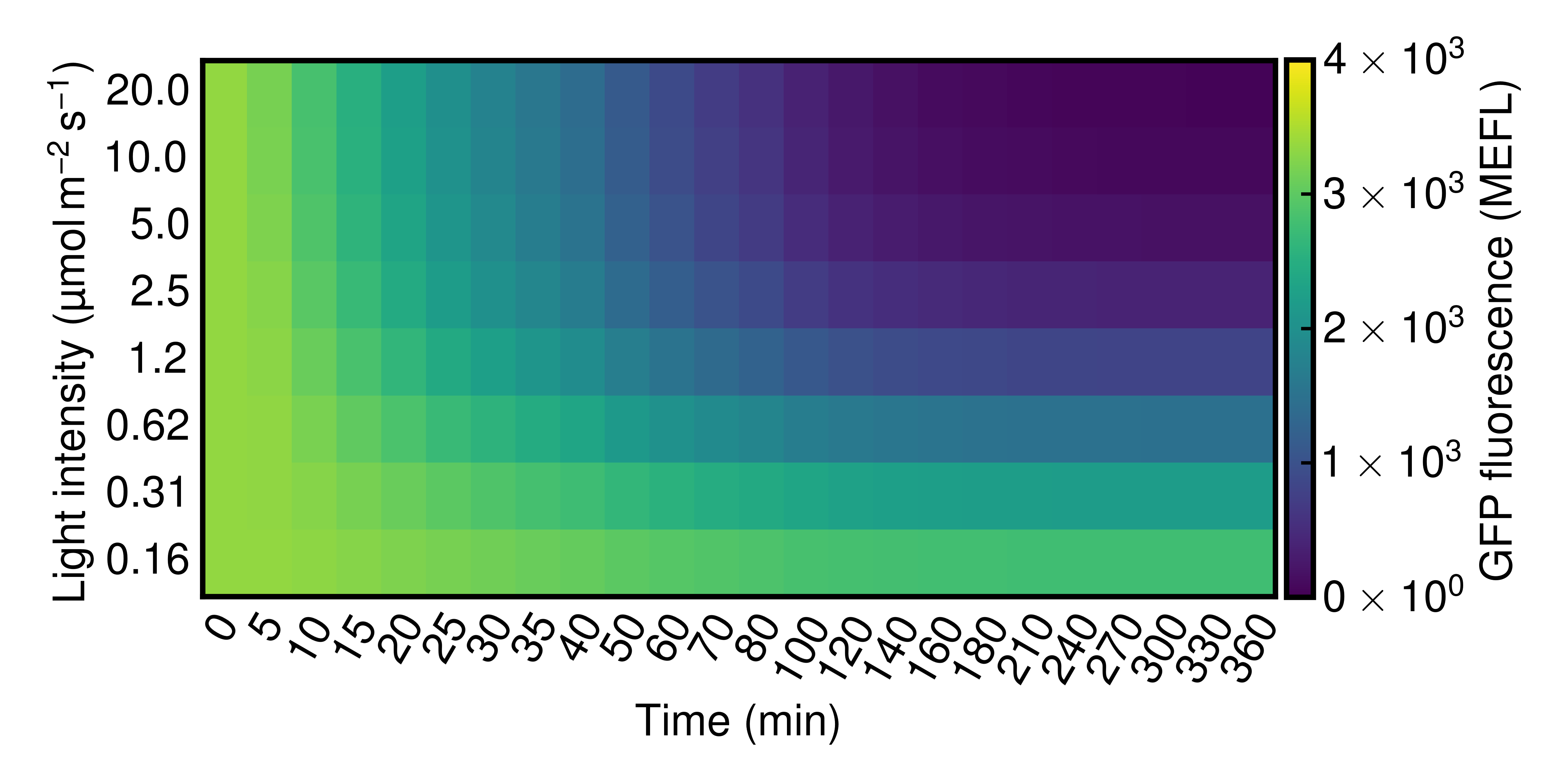

Supplement: Supplementary file 15 — Dataset EV7 [file MSB-13-926-s015.zip › dataset_ev7_cph8-ompr_data_and_analysis/cph8-ompr_analysis/plots/dta_lin_model_hmap.png]

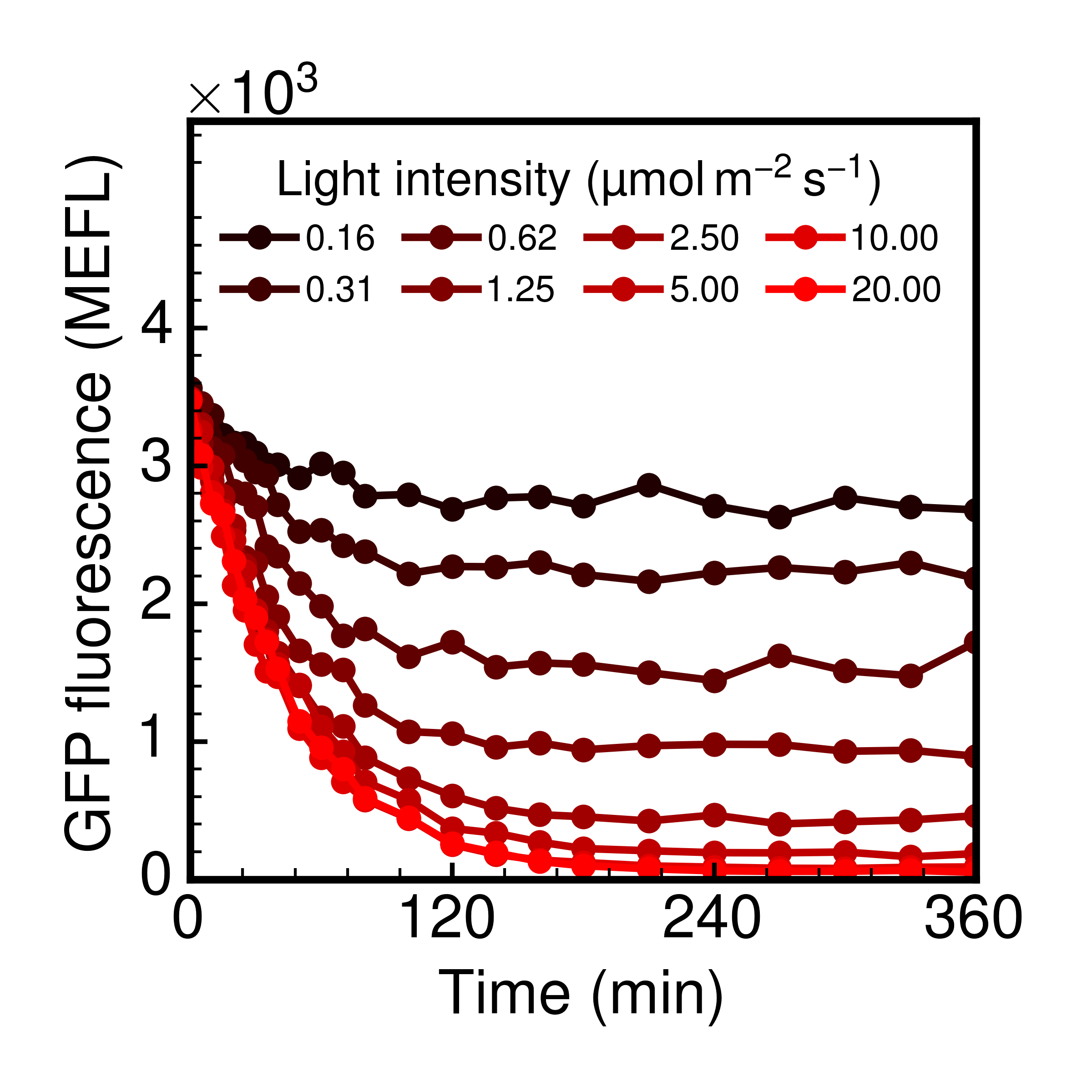

Supplement: Supplementary file 15 — Dataset EV7 [file MSB-13-926-s015.zip › dataset_ev7_cph8-ompr_data_and_analysis/cph8-ompr_analysis/plots/dta_lin_raw.png]

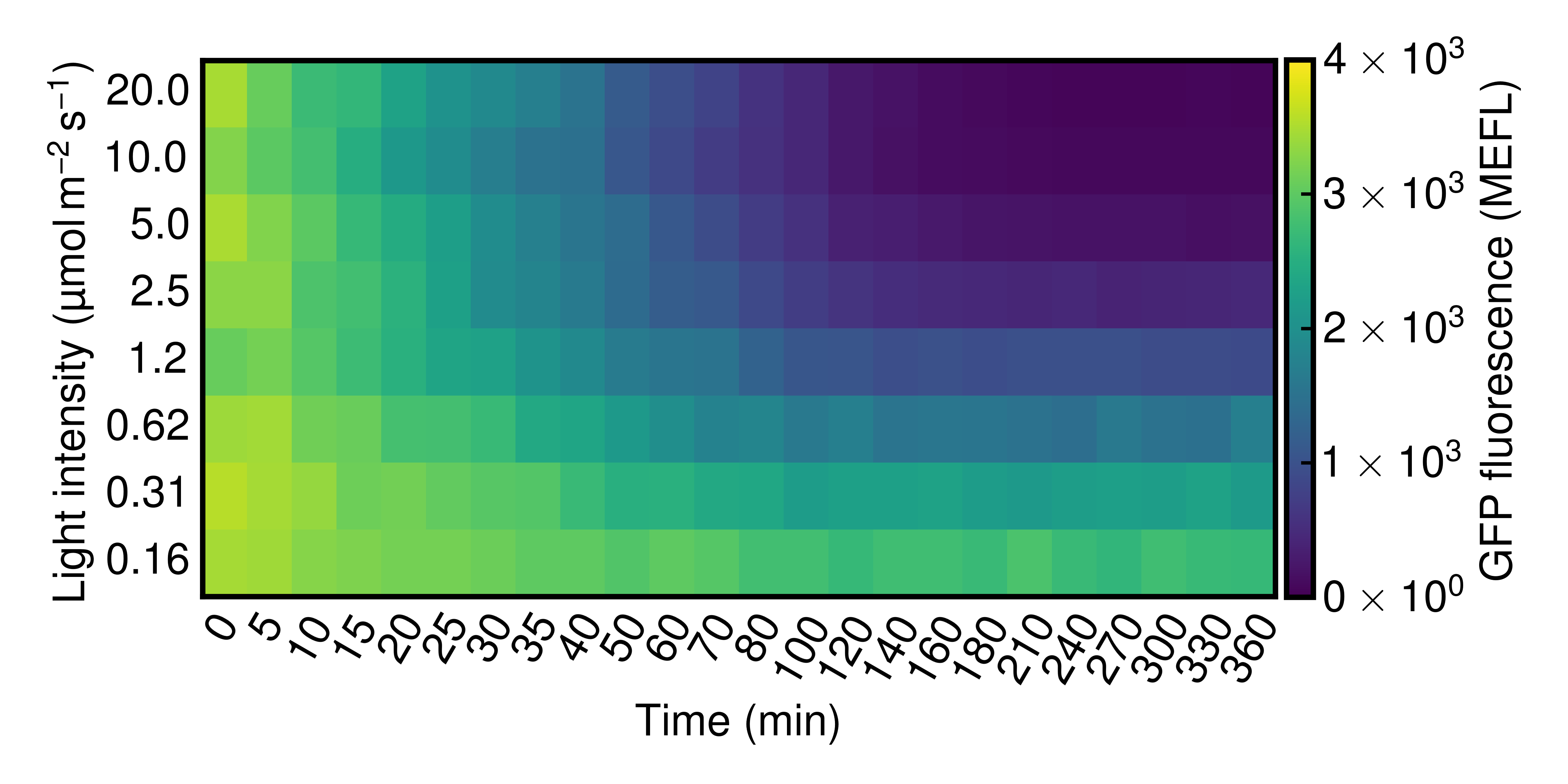

Supplement: Supplementary file 15 — Dataset EV7 [file MSB-13-926-s015.zip › dataset_ev7_cph8-ompr_data_and_analysis/cph8-ompr_analysis/plots/dta_lin_raw_hmap.png]

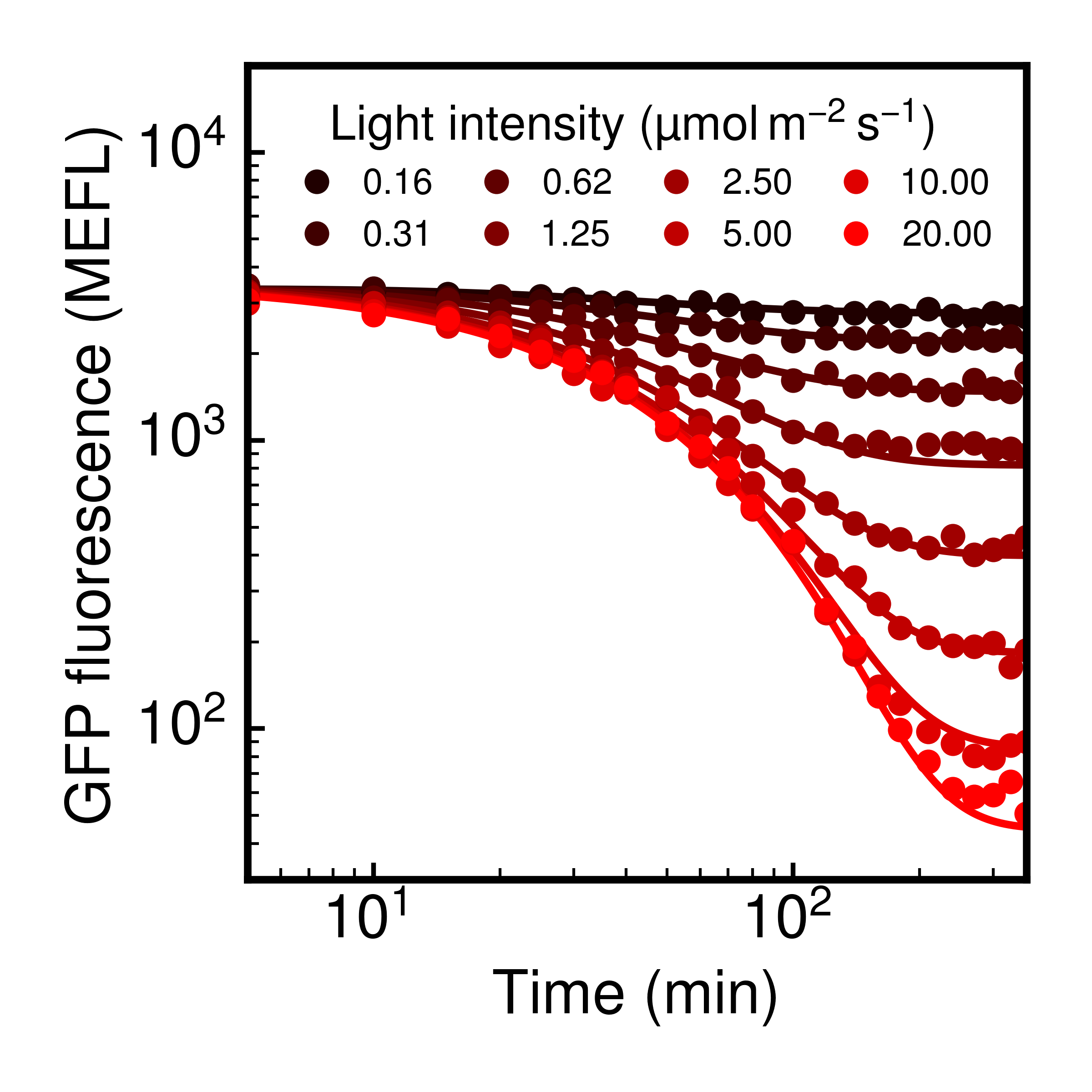

Supplement: Supplementary file 15 — Dataset EV7 [file MSB-13-926-s015.zip › dataset_ev7_cph8-ompr_data_and_analysis/cph8-ompr_analysis/plots/dta_logxy_model.png]

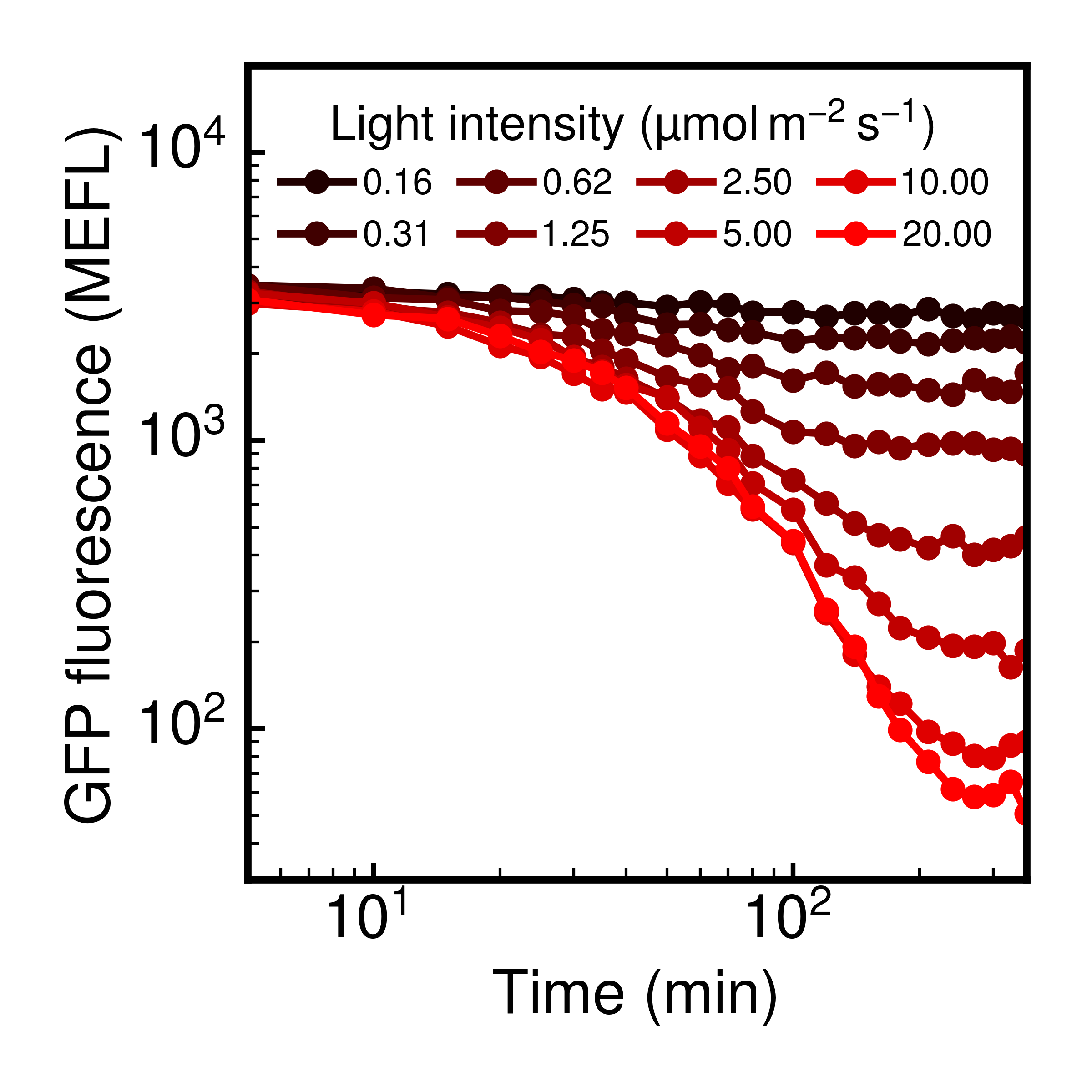

Supplement: Supplementary file 15 — Dataset EV7 [file MSB-13-926-s015.zip › dataset_ev7_cph8-ompr_data_and_analysis/cph8-ompr_analysis/plots/dta_logxy_raw.png]

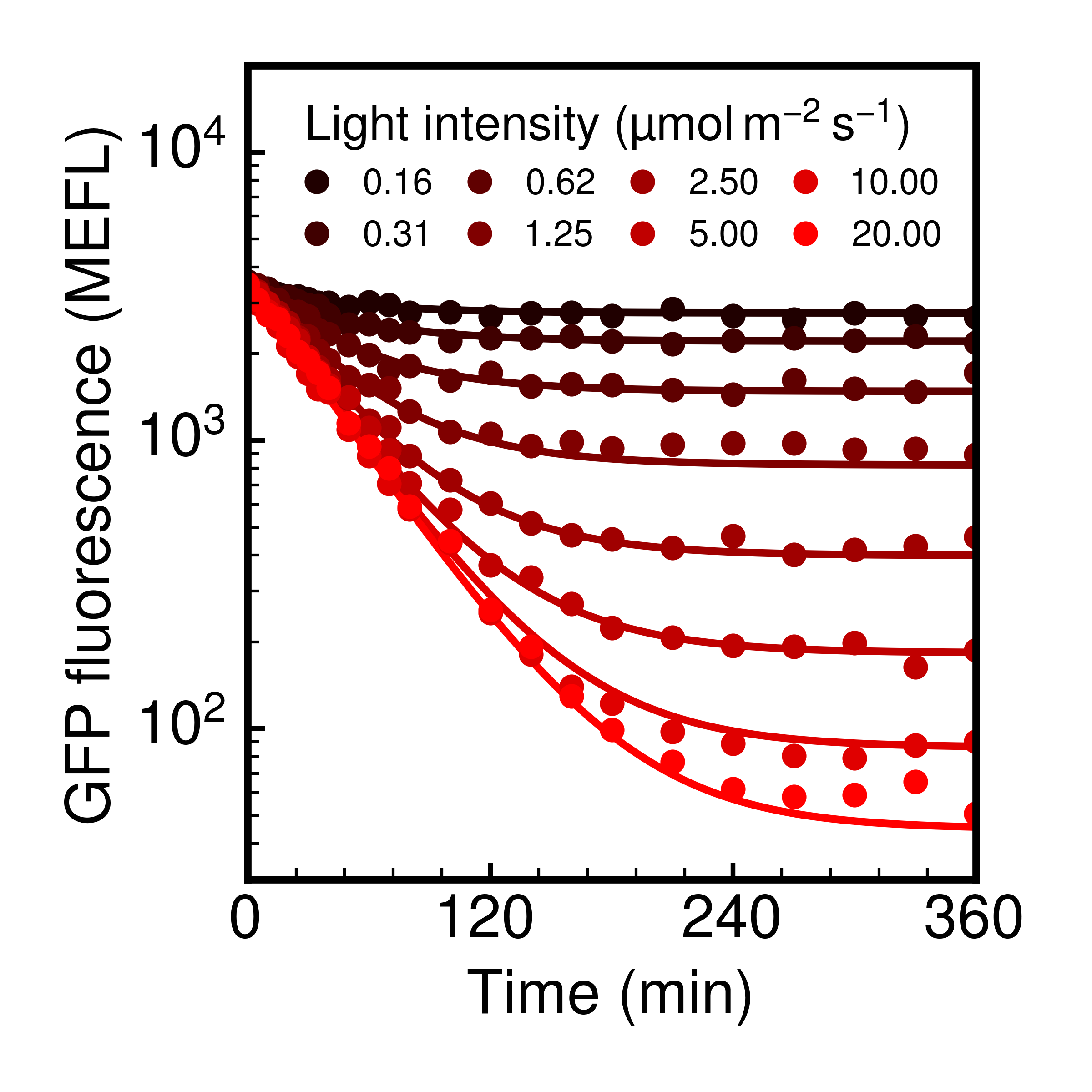

Supplement: Supplementary file 15 — Dataset EV7 [file MSB-13-926-s015.zip › dataset_ev7_cph8-ompr_data_and_analysis/cph8-ompr_analysis/plots/dta_logy_model.png]

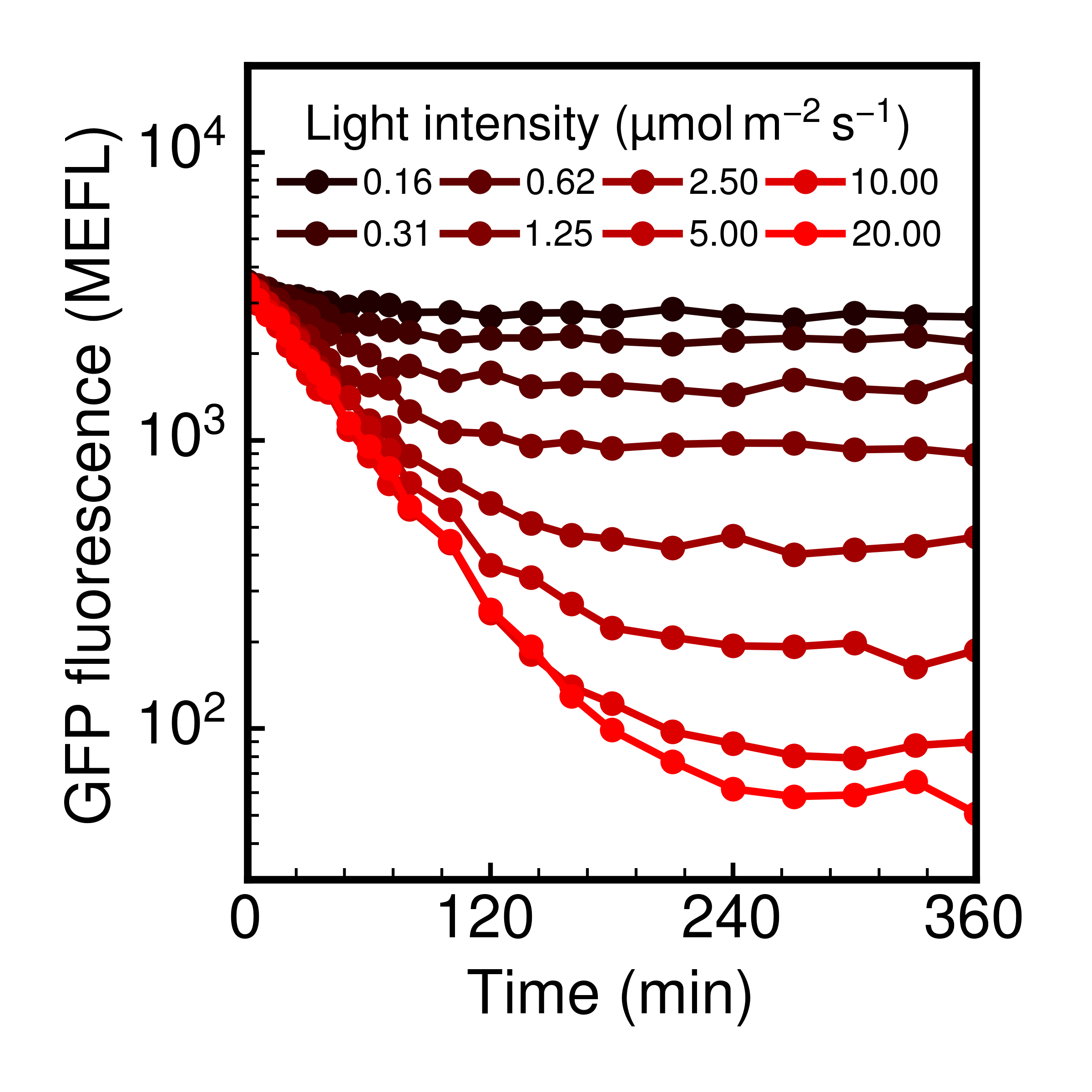

Supplement: Supplementary file 15 — Dataset EV7 [file MSB-13-926-s015.zip › dataset_ev7_cph8-ompr_data_and_analysis/cph8-ompr_analysis/plots/dta_logy_raw.png]

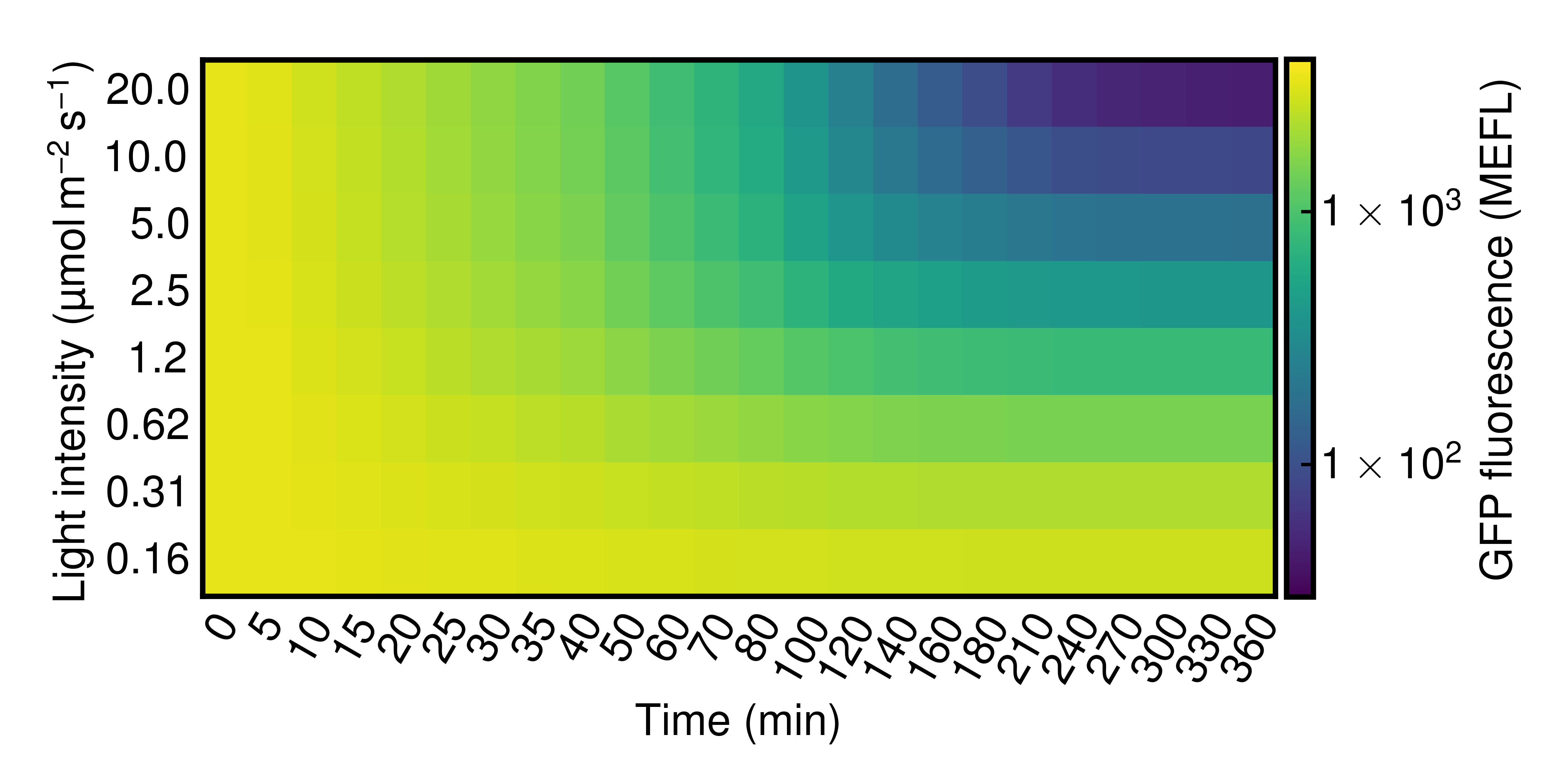

Supplement: Supplementary file 15 — Dataset EV7 [file MSB-13-926-s015.zip › dataset_ev7_cph8-ompr_data_and_analysis/cph8-ompr_analysis/plots/dta_logz_model_hmap.png]

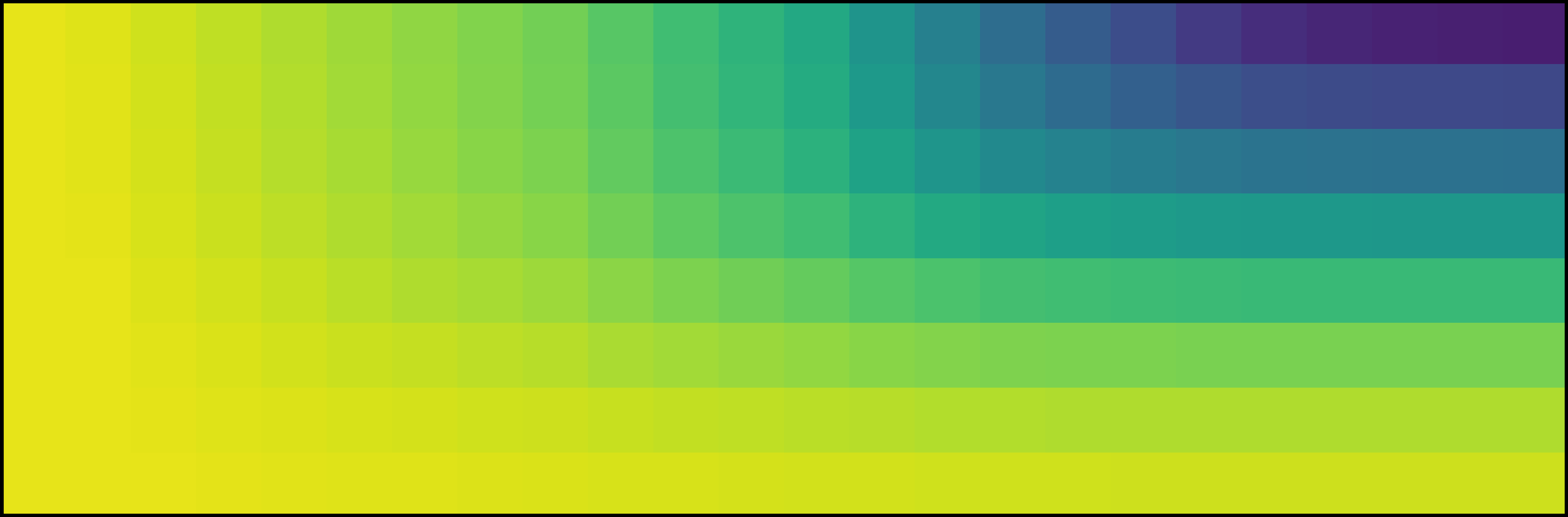

Supplement: Supplementary file 15 — Dataset EV7 [file MSB-13-926-s015.zip › dataset_ev7_cph8-ompr_data_and_analysis/cph8-ompr_analysis/plots/dta_logz_model_nolabel_hmap.png]

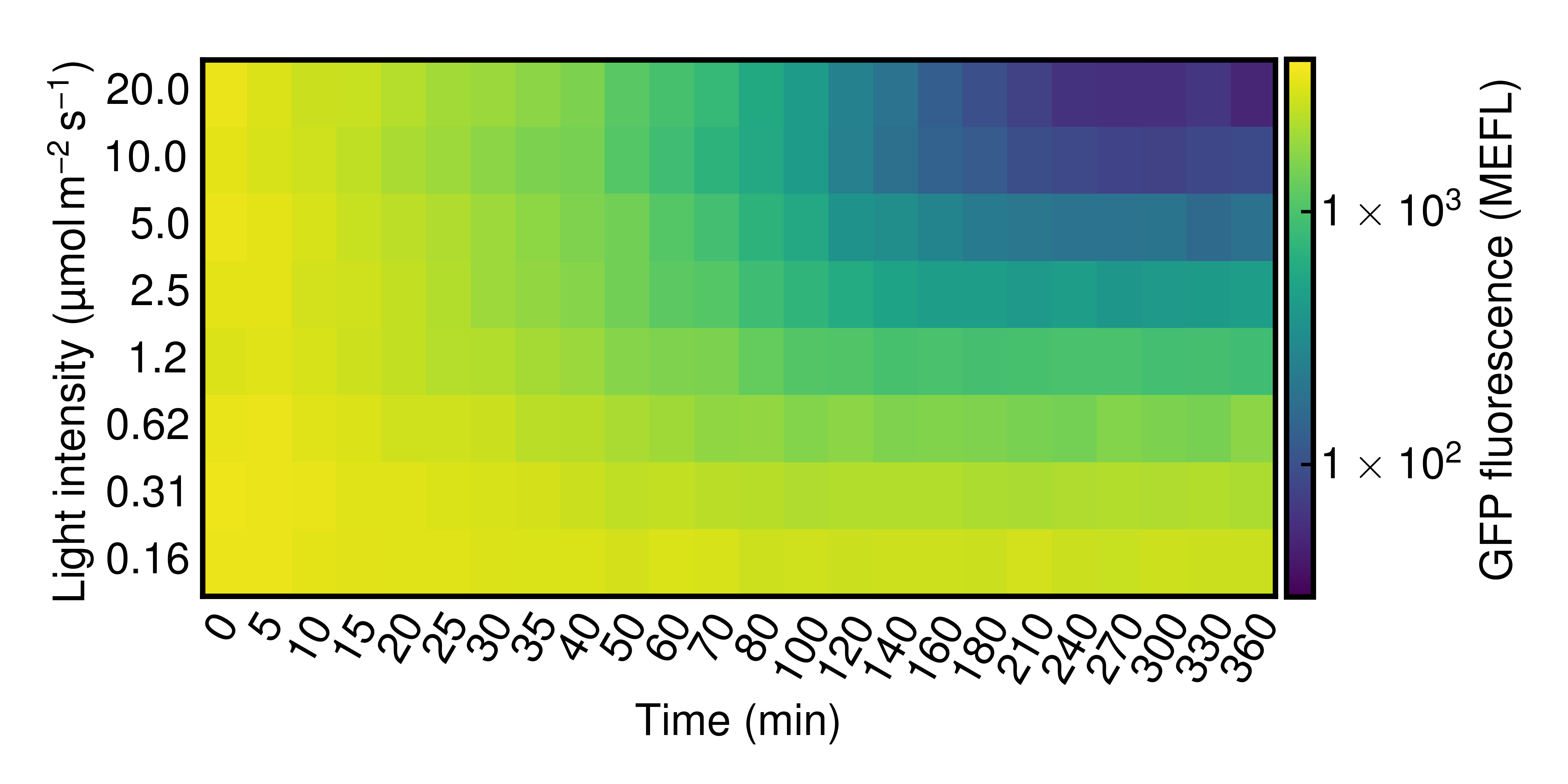

Supplement: Supplementary file 15 — Dataset EV7 [file MSB-13-926-s015.zip › dataset_ev7_cph8-ompr_data_and_analysis/cph8-ompr_analysis/plots/dta_logz_raw_hmap.png]

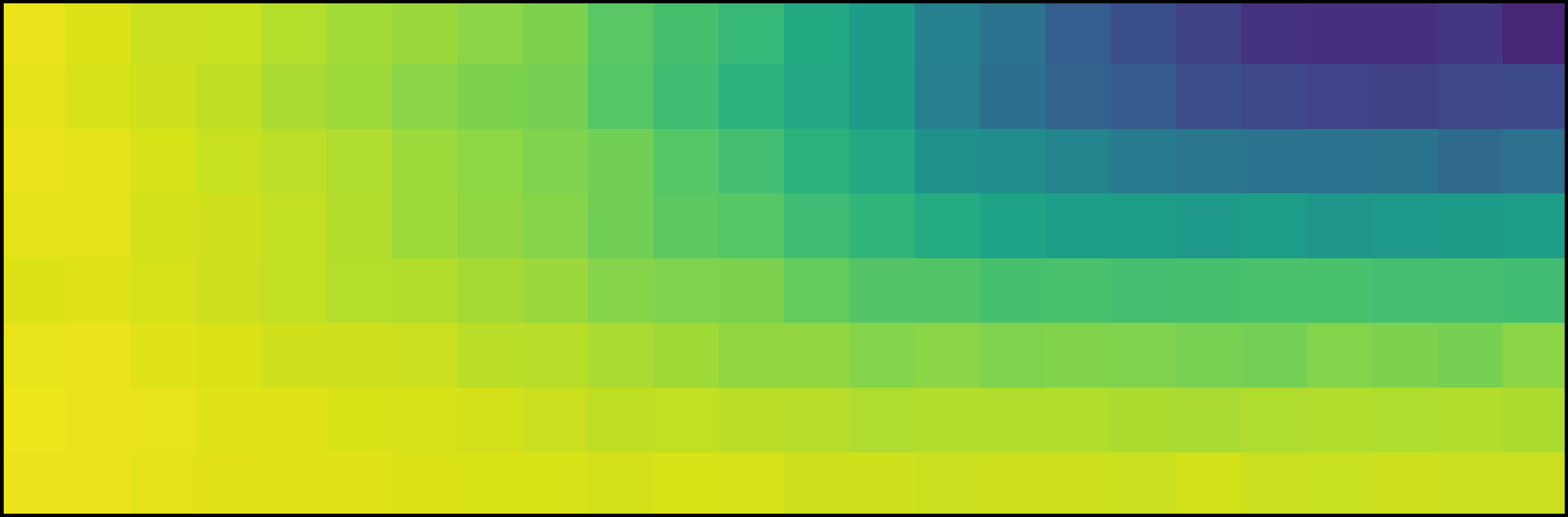

Supplement: Supplementary file 15 — Dataset EV7 [file MSB-13-926-s015.zip › dataset_ev7_cph8-ompr_data_and_analysis/cph8-ompr_analysis/plots/dta_logz_raw_nolabel_hmap.png]

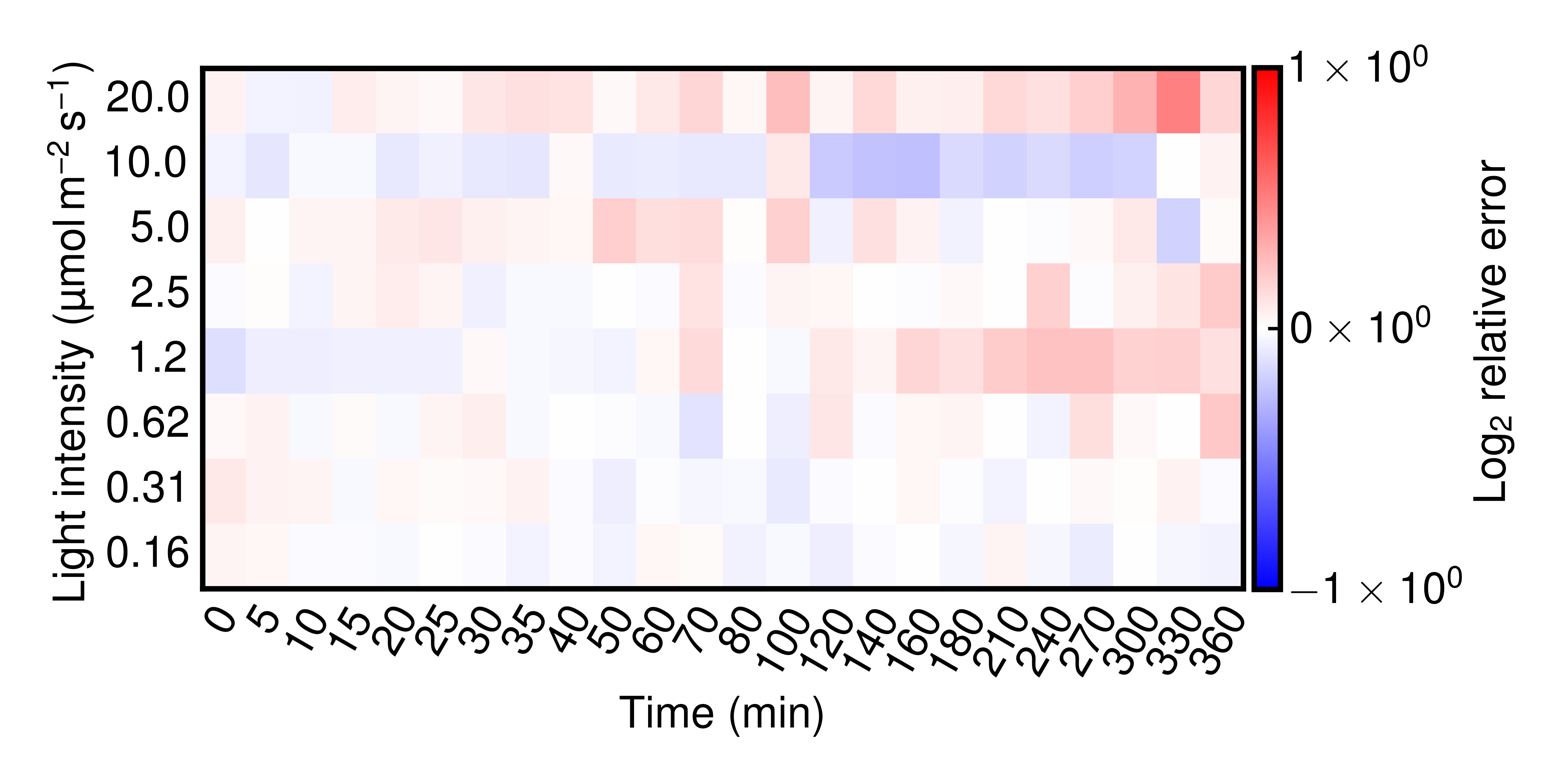

Supplement: Supplementary file 15 — Dataset EV7 [file MSB-13-926-s015.zip › dataset_ev7_cph8-ompr_data_and_analysis/cph8-ompr_analysis/plots/dta_rel_residual_hmap.png]

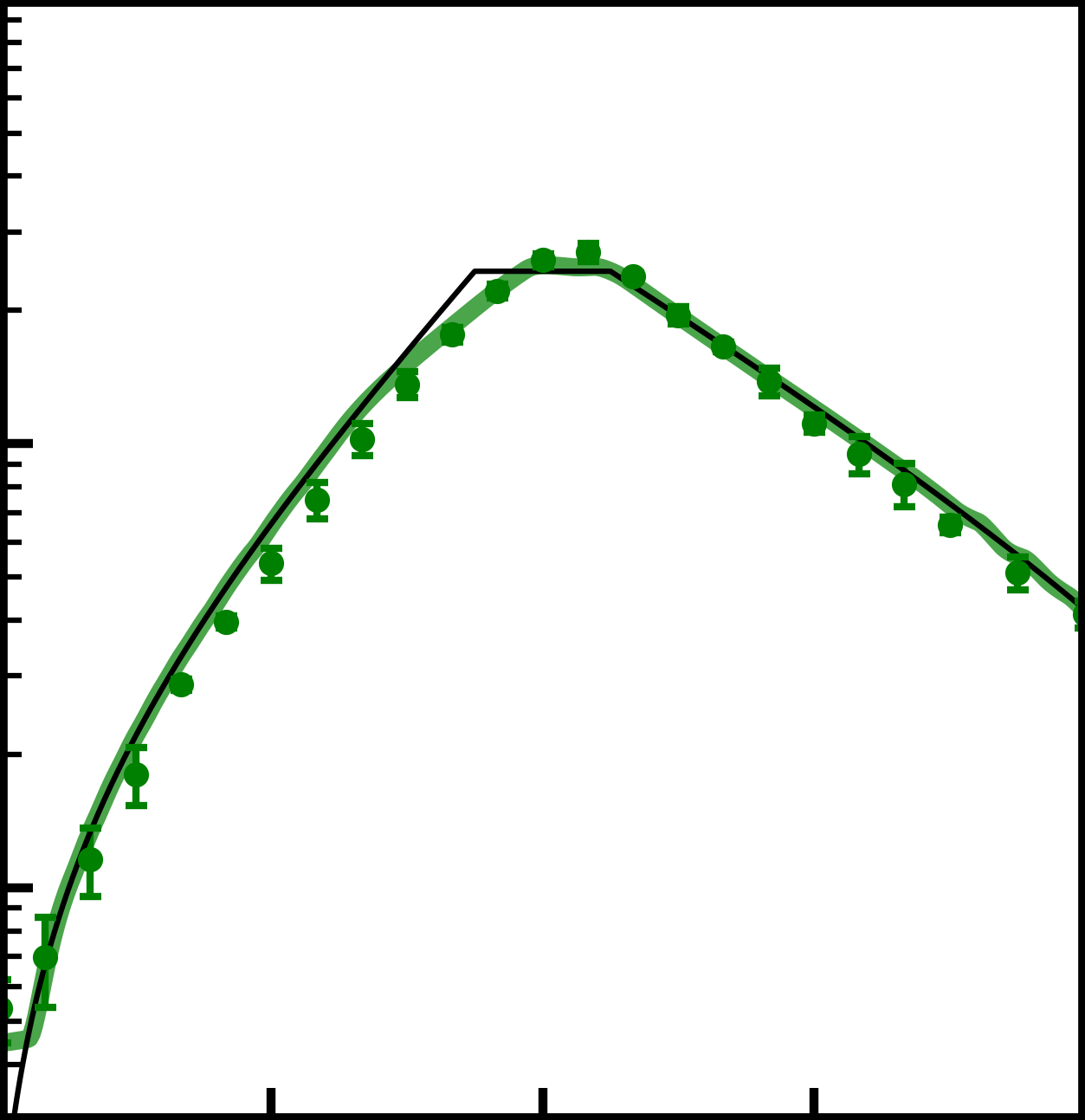

Supplement: Supplementary file 15 — Dataset EV7 [file MSB-13-926-s015.zip › dataset_ev7_cph8-ompr_data_and_analysis/cph8-ompr_analysis/plots/dv-comp_logy_full_data.png]

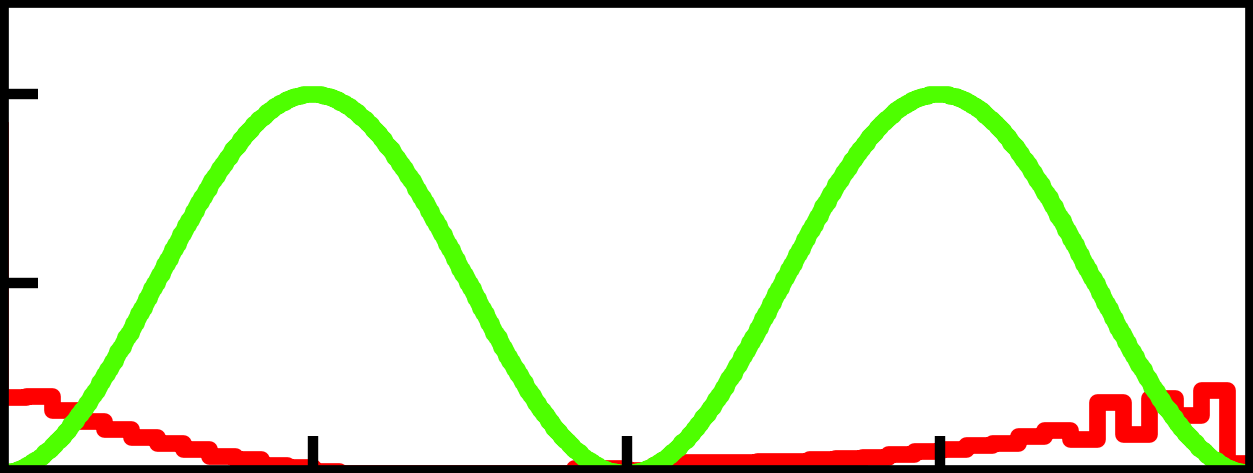

Supplement: Supplementary file 15 — Dataset EV7 [file MSB-13-926-s015.zip › dataset_ev7_cph8-ompr_data_and_analysis/cph8-ompr_analysis/plots/dv-comp_logy_full_intlin.png]

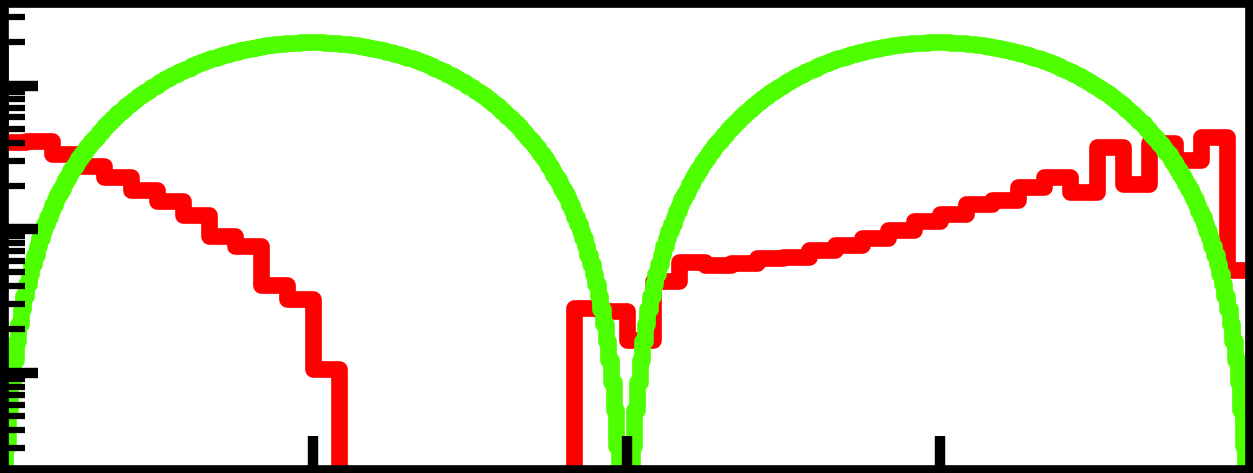

Supplement: Supplementary file 15 — Dataset EV7 [file MSB-13-926-s015.zip › dataset_ev7_cph8-ompr_data_and_analysis/cph8-ompr_analysis/plots/dv-comp_logy_full_intlog.png]

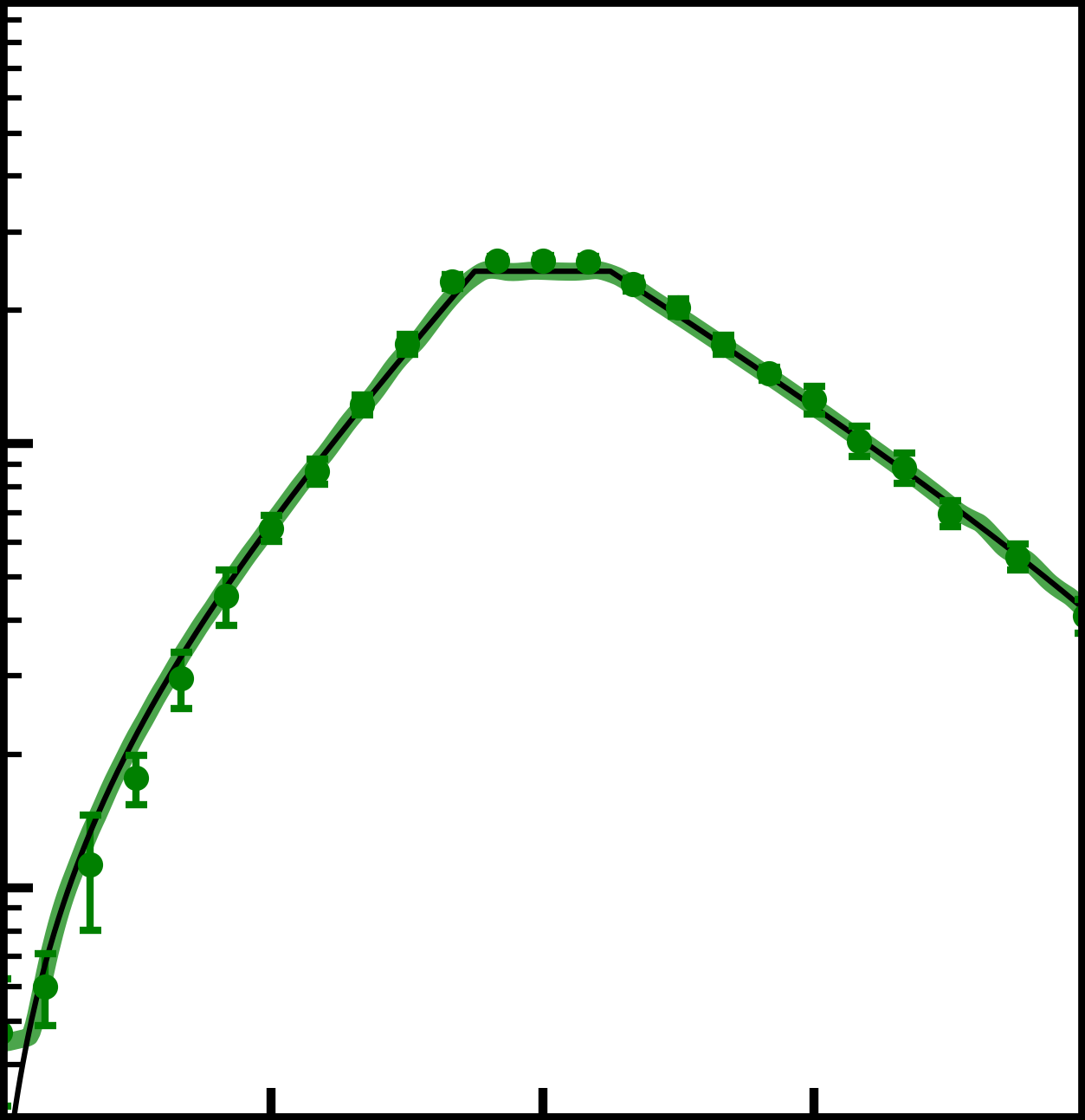

Supplement: Supplementary file 15 — Dataset EV7 [file MSB-13-926-s015.zip › dataset_ev7_cph8-ompr_data_and_analysis/cph8-ompr_analysis/plots/dv-mono-r_logy_full_data.png]

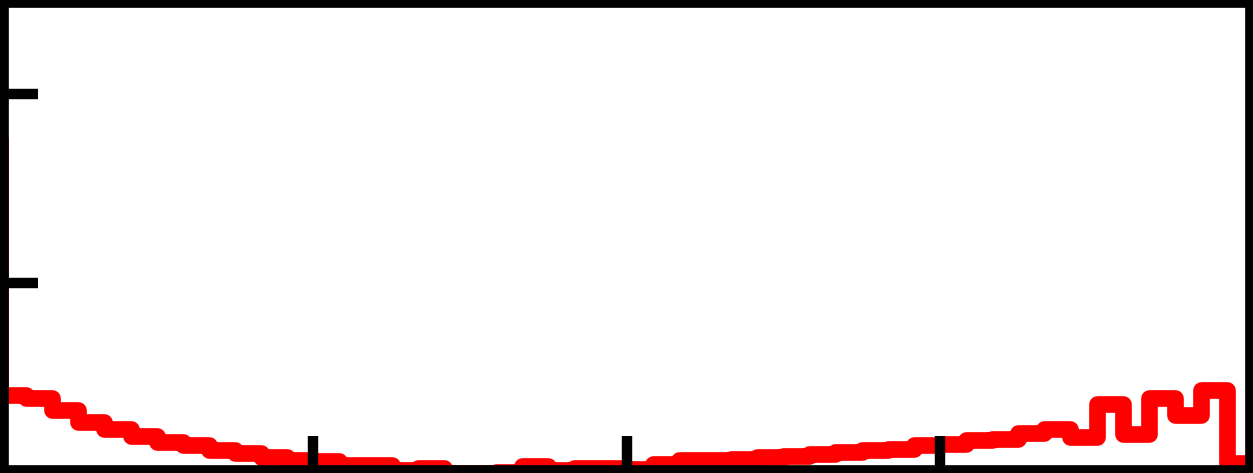

Supplement: Supplementary file 15 — Dataset EV7 [file MSB-13-926-s015.zip › dataset_ev7_cph8-ompr_data_and_analysis/cph8-ompr_analysis/plots/dv-mono-r_logy_full_intlin.png]

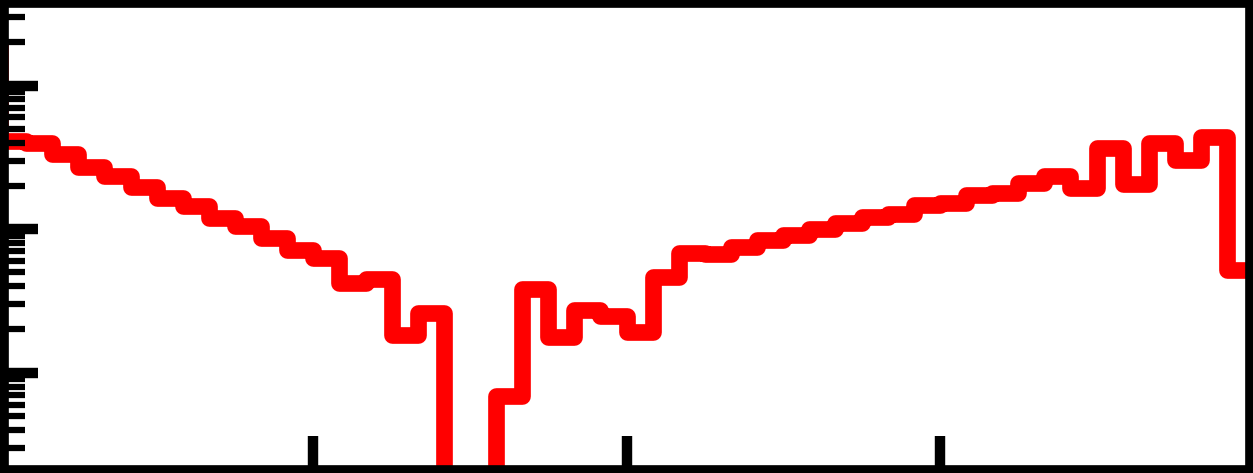

Supplement: Supplementary file 15 — Dataset EV7 [file MSB-13-926-s015.zip › dataset_ev7_cph8-ompr_data_and_analysis/cph8-ompr_analysis/plots/dv-mono-r_logy_full_intlog.png]

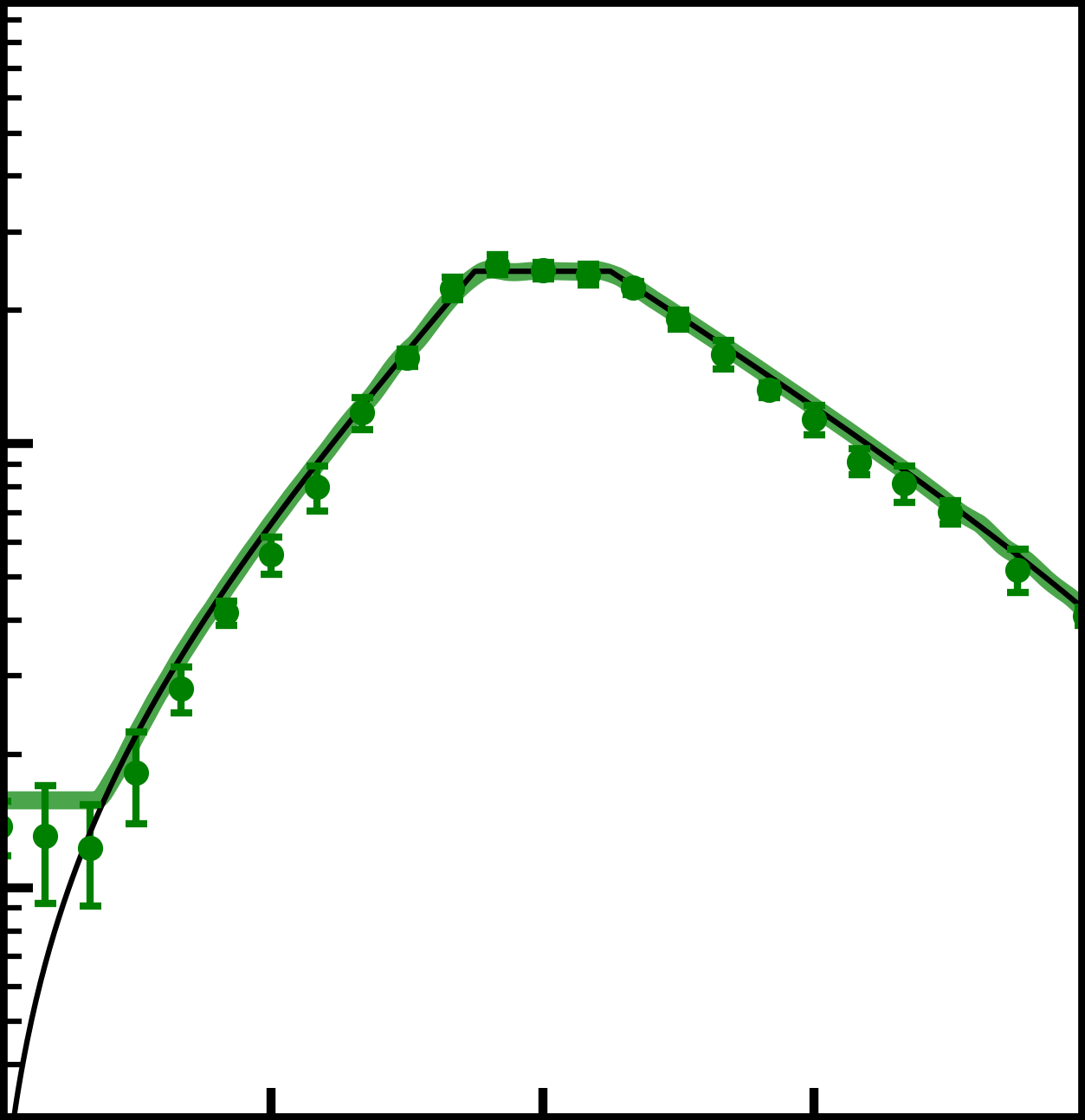

Supplement: Supplementary file 15 — Dataset EV7 [file MSB-13-926-s015.zip › dataset_ev7_cph8-ompr_data_and_analysis/cph8-ompr_analysis/plots/dv-mono-uv_logy_full_data.png]

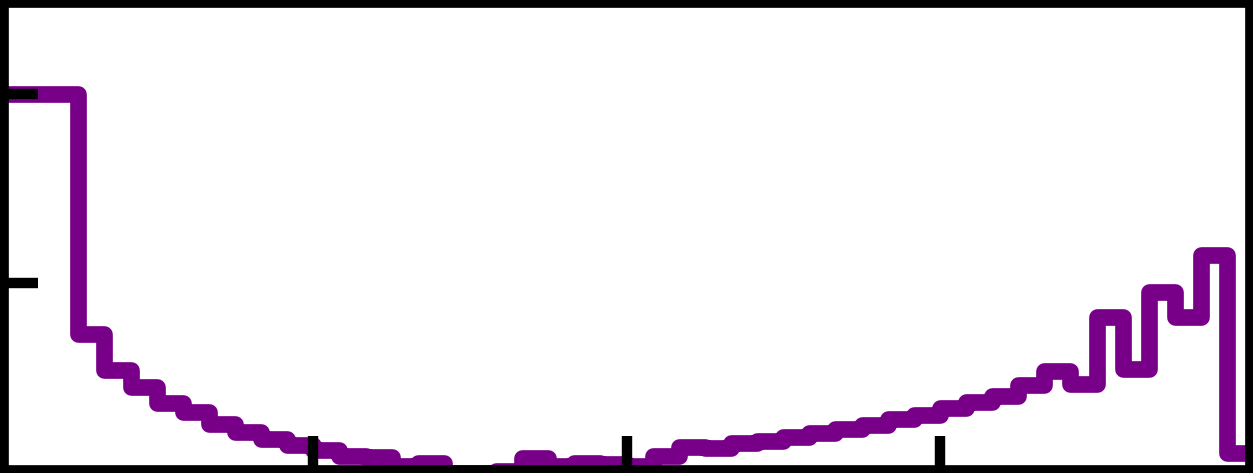

Supplement: Supplementary file 15 — Dataset EV7 [file MSB-13-926-s015.zip › dataset_ev7_cph8-ompr_data_and_analysis/cph8-ompr_analysis/plots/dv-mono-uv_logy_full_intlin.png]

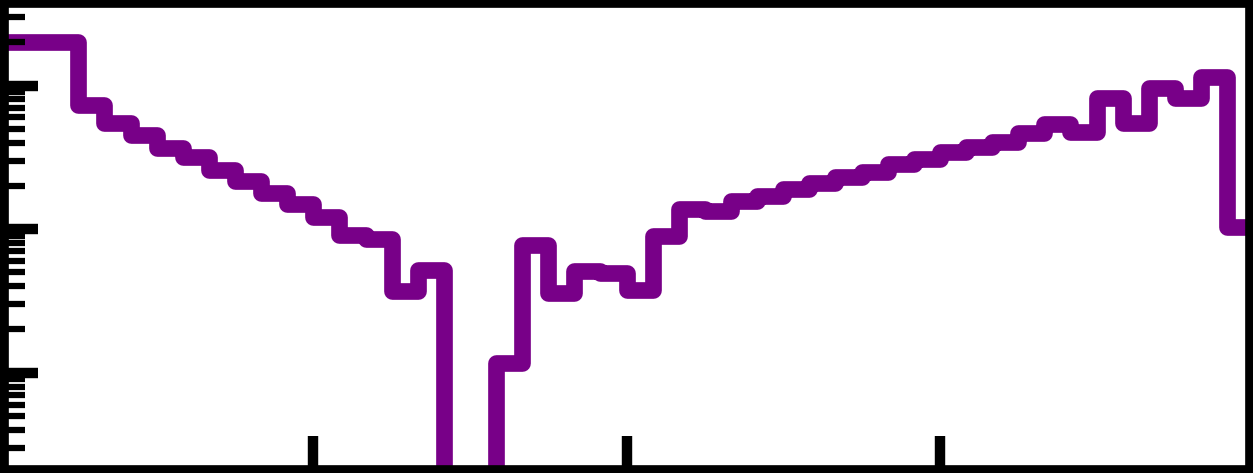

Supplement: Supplementary file 15 — Dataset EV7 [file MSB-13-926-s015.zip › dataset_ev7_cph8-ompr_data_and_analysis/cph8-ompr_analysis/plots/dv-mono-uv_logy_full_intlog.png]

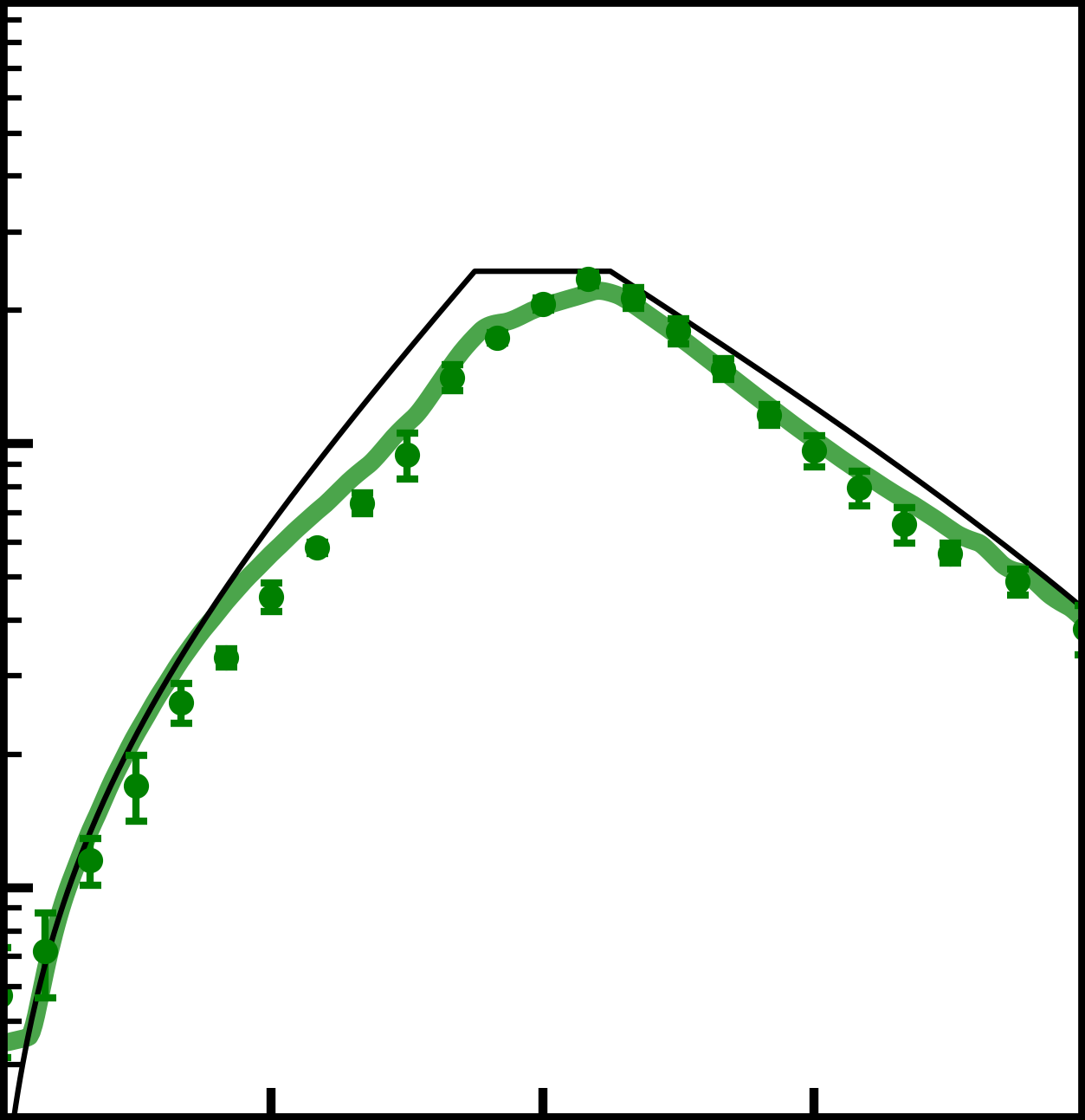

Supplement: Supplementary file 15 — Dataset EV7 [file MSB-13-926-s015.zip › dataset_ev7_cph8-ompr_data_and_analysis/cph8-ompr_analysis/plots/dv-pert_logy_full_data.png]

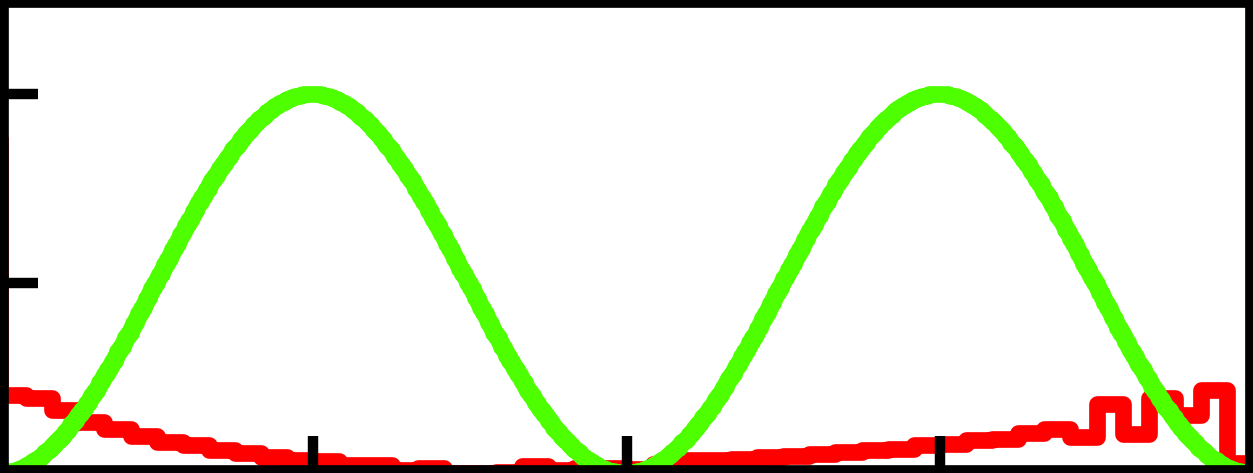

Supplement: Supplementary file 15 — Dataset EV7 [file MSB-13-926-s015.zip › dataset_ev7_cph8-ompr_data_and_analysis/cph8-ompr_analysis/plots/dv-pert_logy_full_intlin.png]

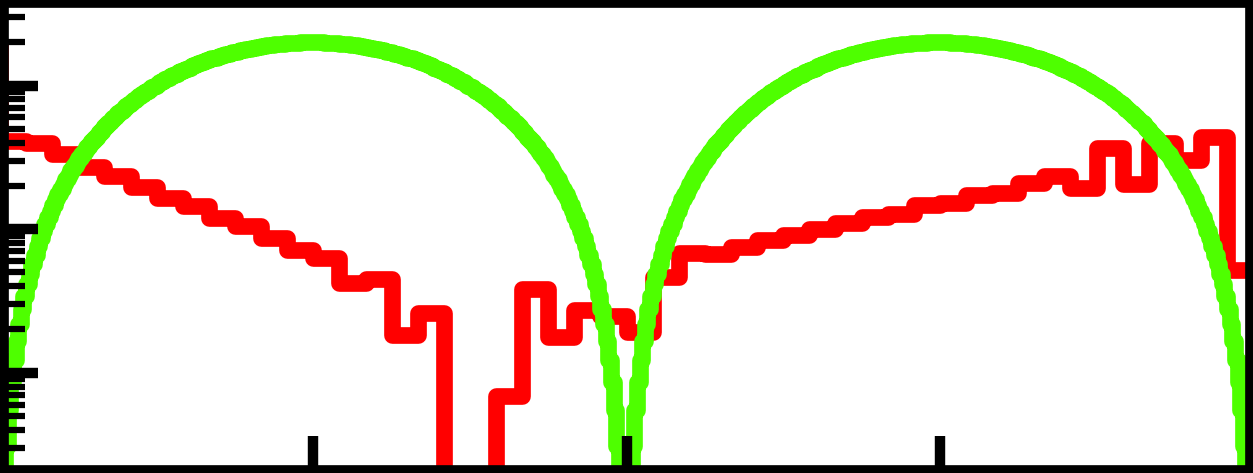

Supplement: Supplementary file 15 — Dataset EV7 [file MSB-13-926-s015.zip › dataset_ev7_cph8-ompr_data_and_analysis/cph8-ompr_analysis/plots/dv-pert_logy_full_intlog.png]

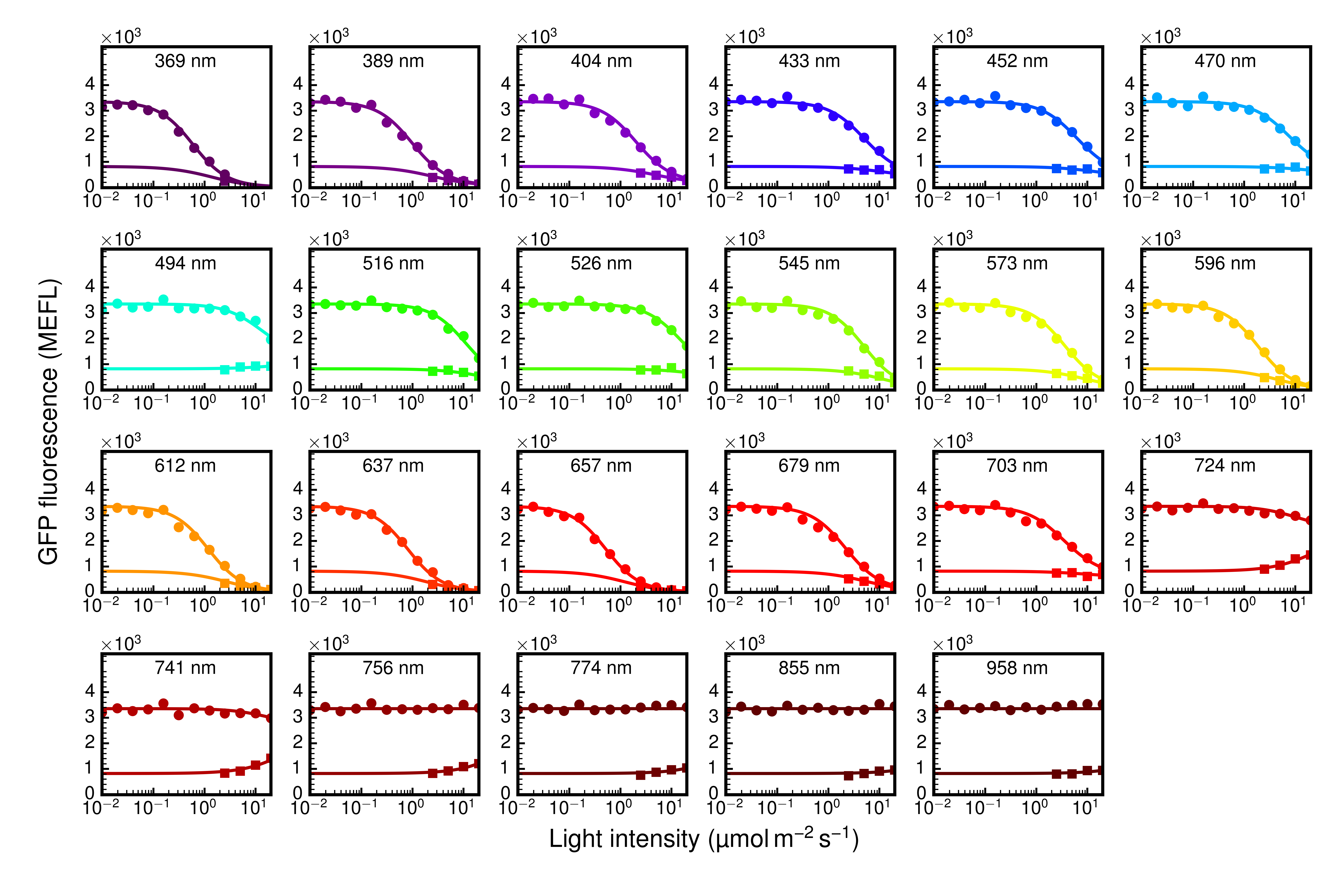

Supplement: Supplementary file 15 — Dataset EV7 [file MSB-13-926-s015.zip › dataset_ev7_cph8-ompr_data_and_analysis/cph8-ompr_analysis/plots/led_fit_lin.png]

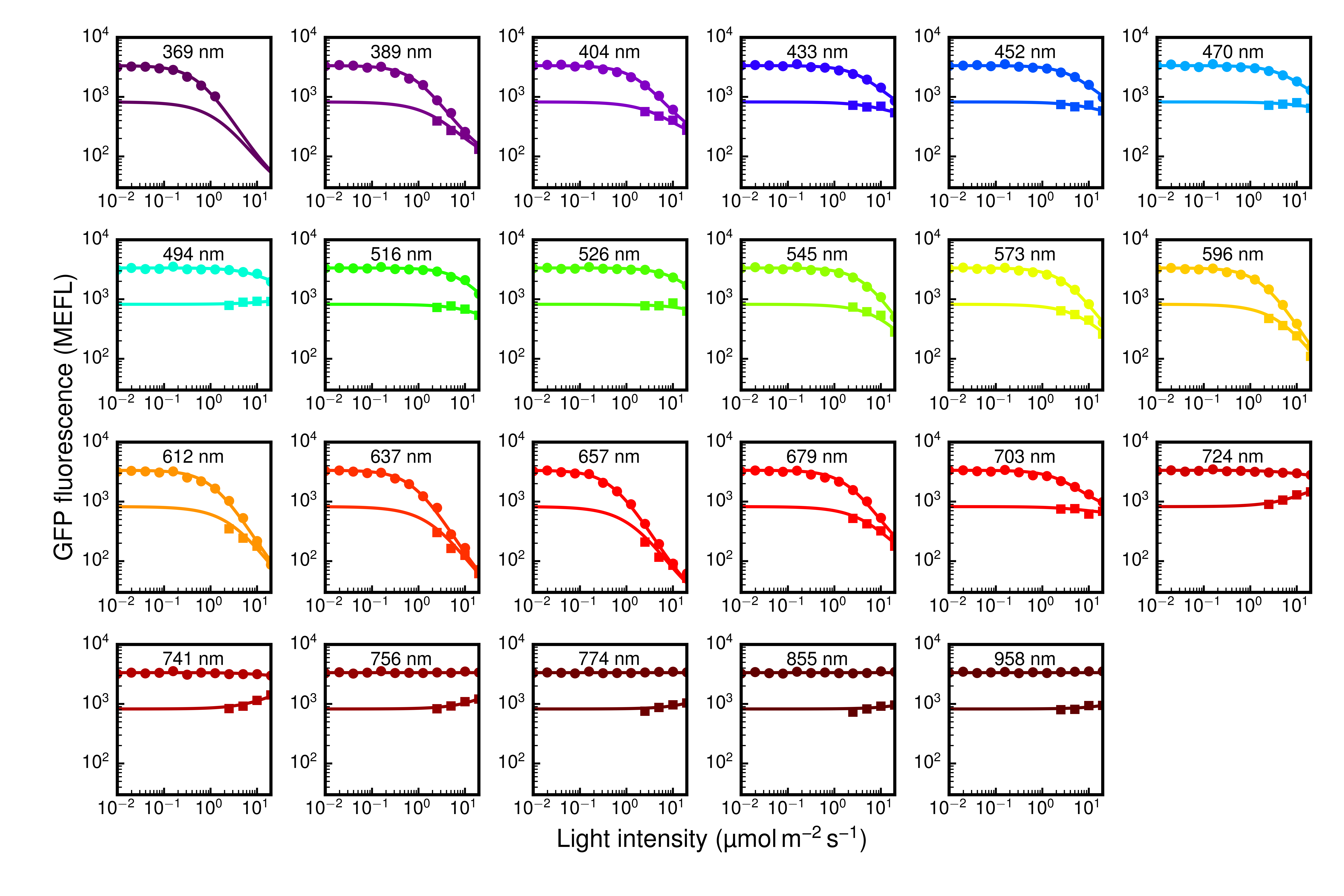

Supplement: Supplementary file 15 — Dataset EV7 [file MSB-13-926-s015.zip › dataset_ev7_cph8-ompr_data_and_analysis/cph8-ompr_analysis/plots/led_fit_log.png]

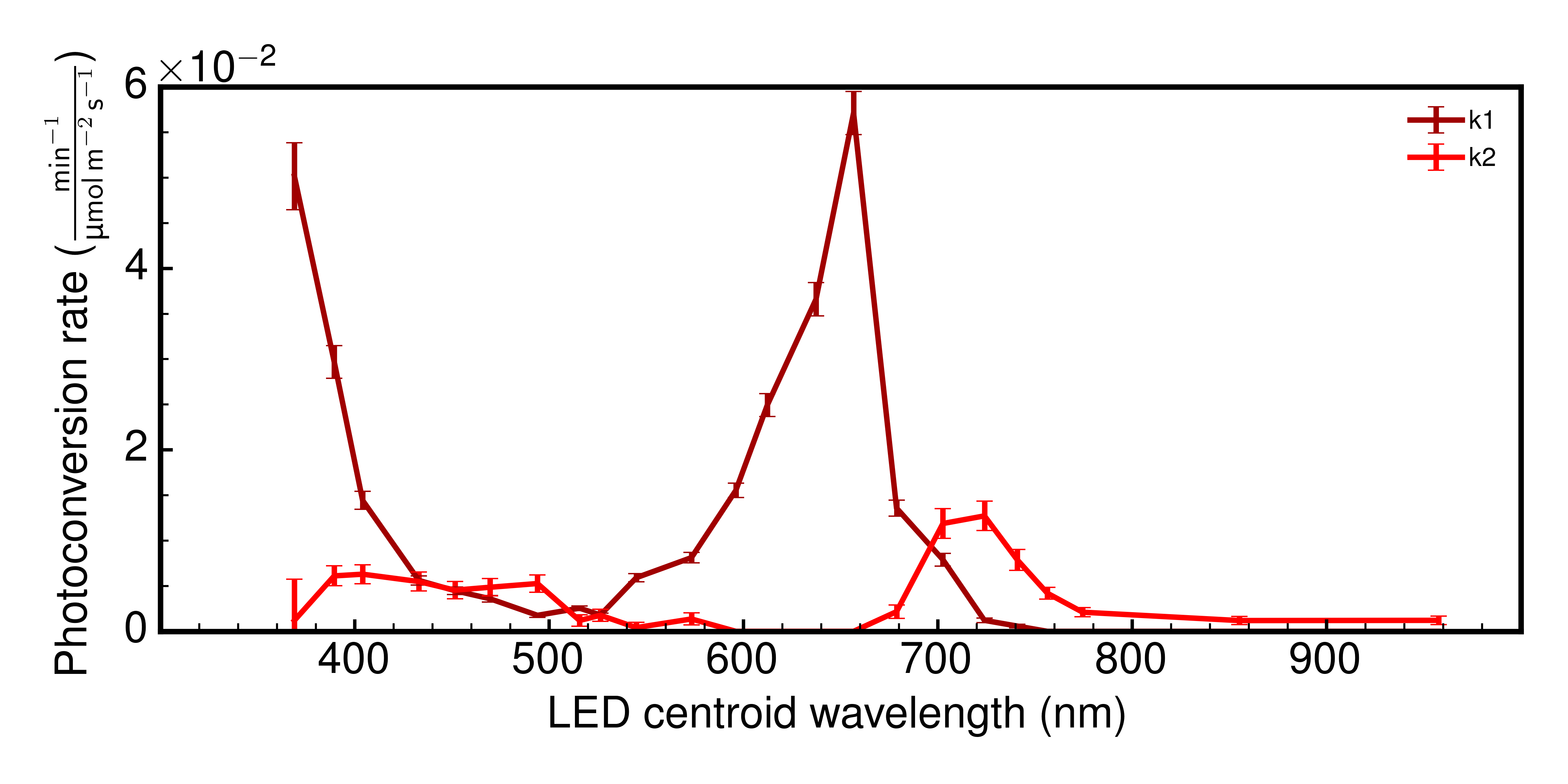

Supplement: Supplementary file 15 — Dataset EV7 [file MSB-13-926-s015.zip › dataset_ev7_cph8-ompr_data_and_analysis/cph8-ompr_analysis/plots/pcs.png]

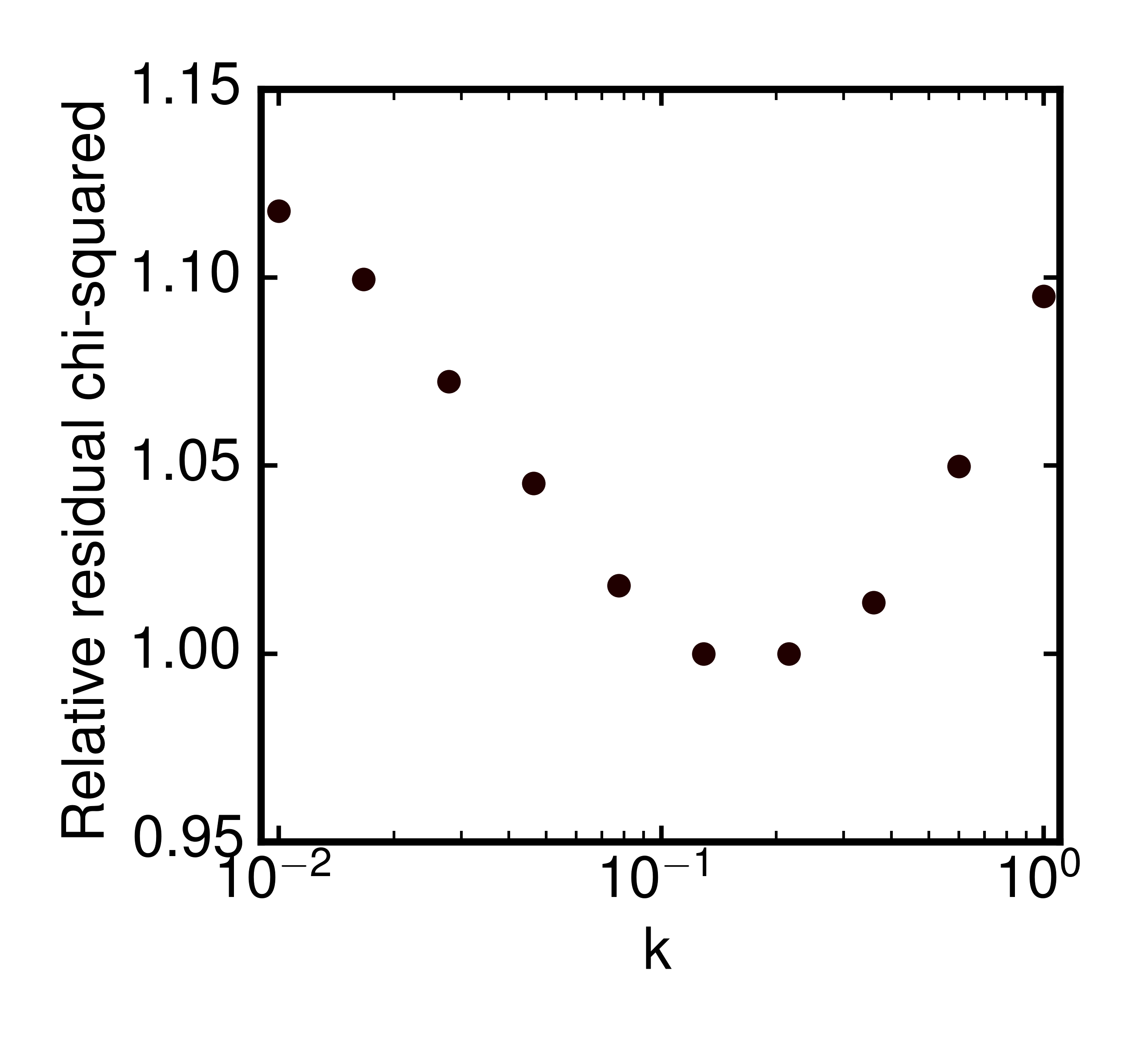

Supplement: Supplementary file 15 — Dataset EV7 [file MSB-13-926-s015.zip › dataset_ev7_cph8-ompr_data_and_analysis/cph8-ompr_analysis/plots/pcs.png_chisq.png]

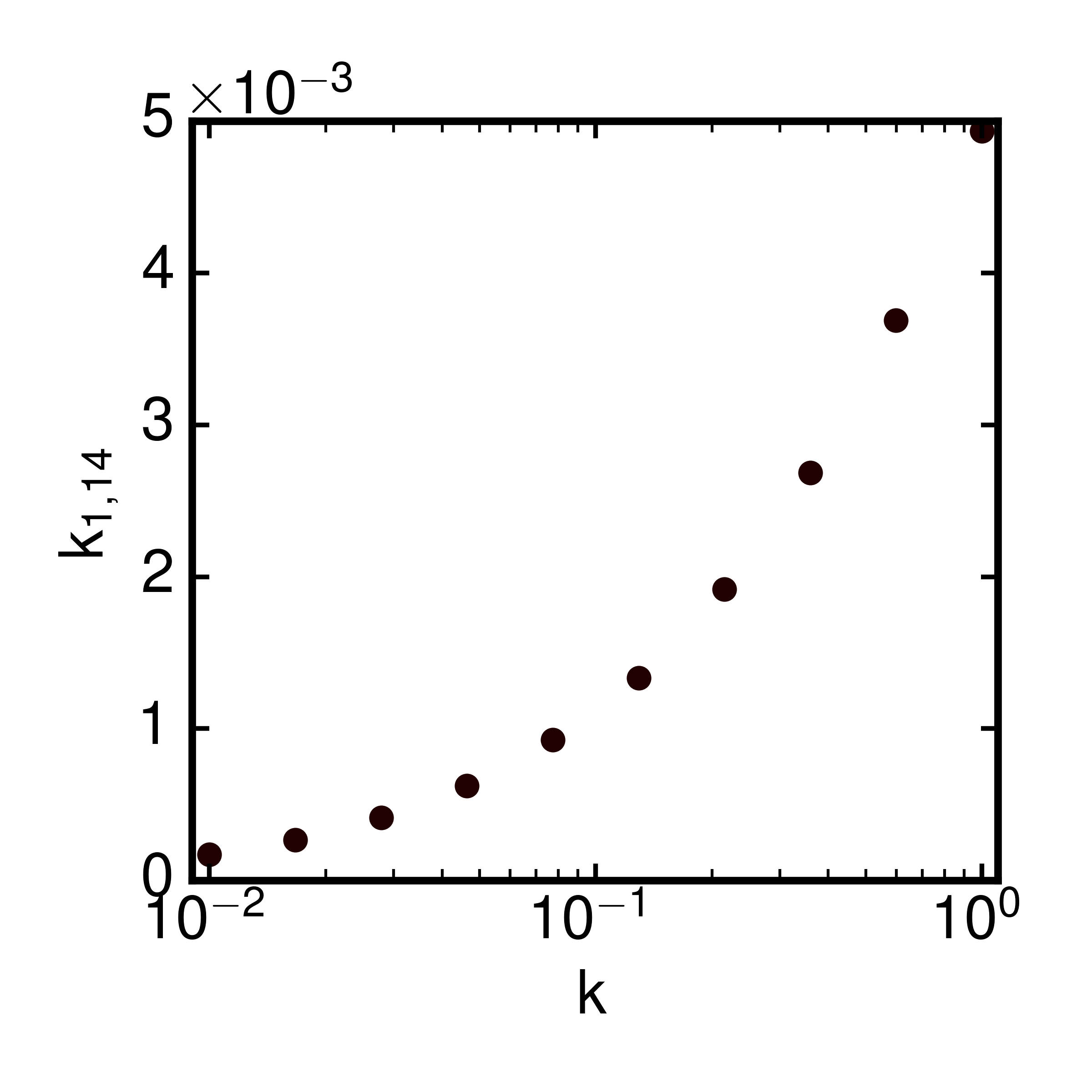

Supplement: Supplementary file 15 — Dataset EV7 [file MSB-13-926-s015.zip › dataset_ev7_cph8-ompr_data_and_analysis/cph8-ompr_analysis/plots/pcs.png_k1.png]

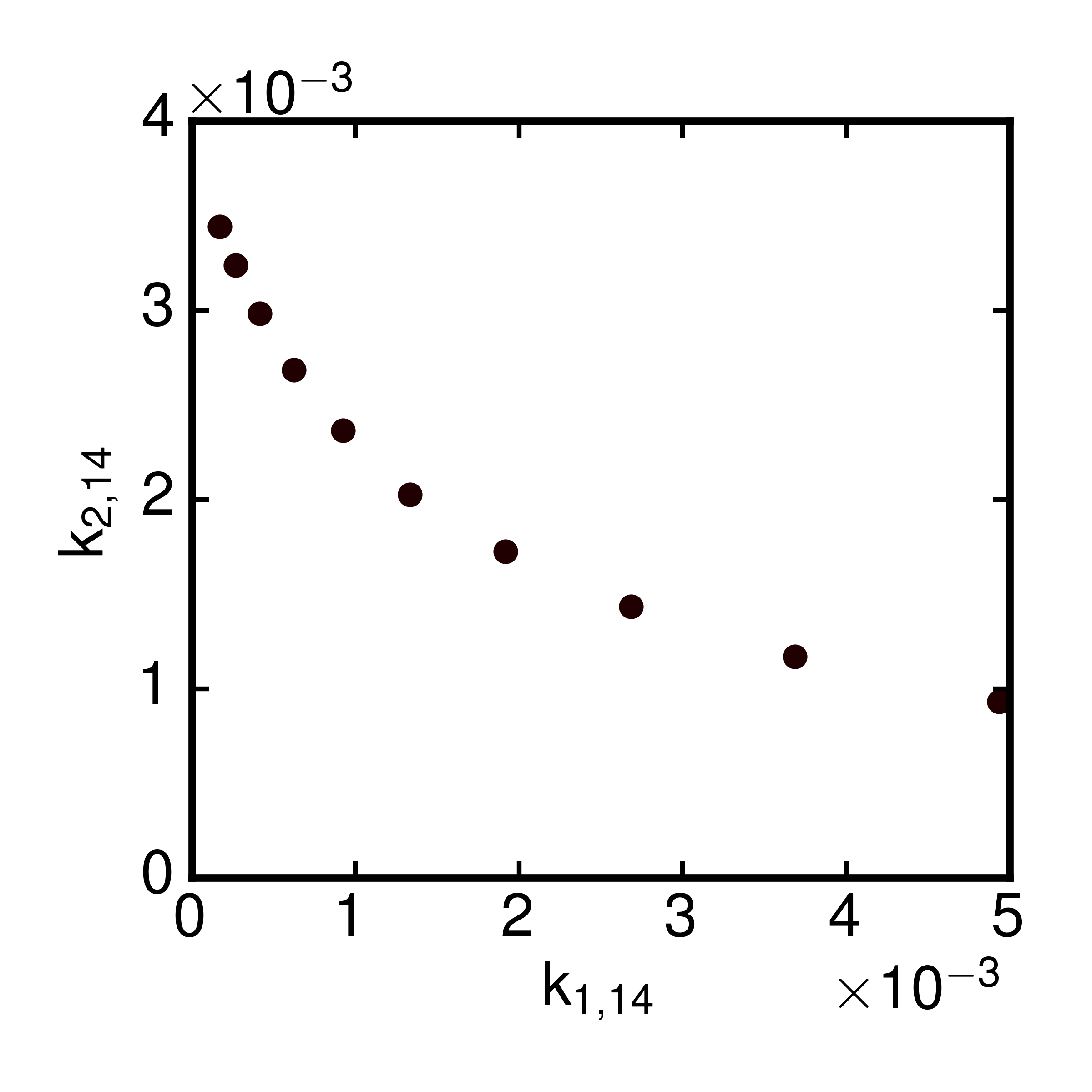

Supplement: Supplementary file 15 — Dataset EV7 [file MSB-13-926-s015.zip › dataset_ev7_cph8-ompr_data_and_analysis/cph8-ompr_analysis/plots/pcs.png_k1k2.png]

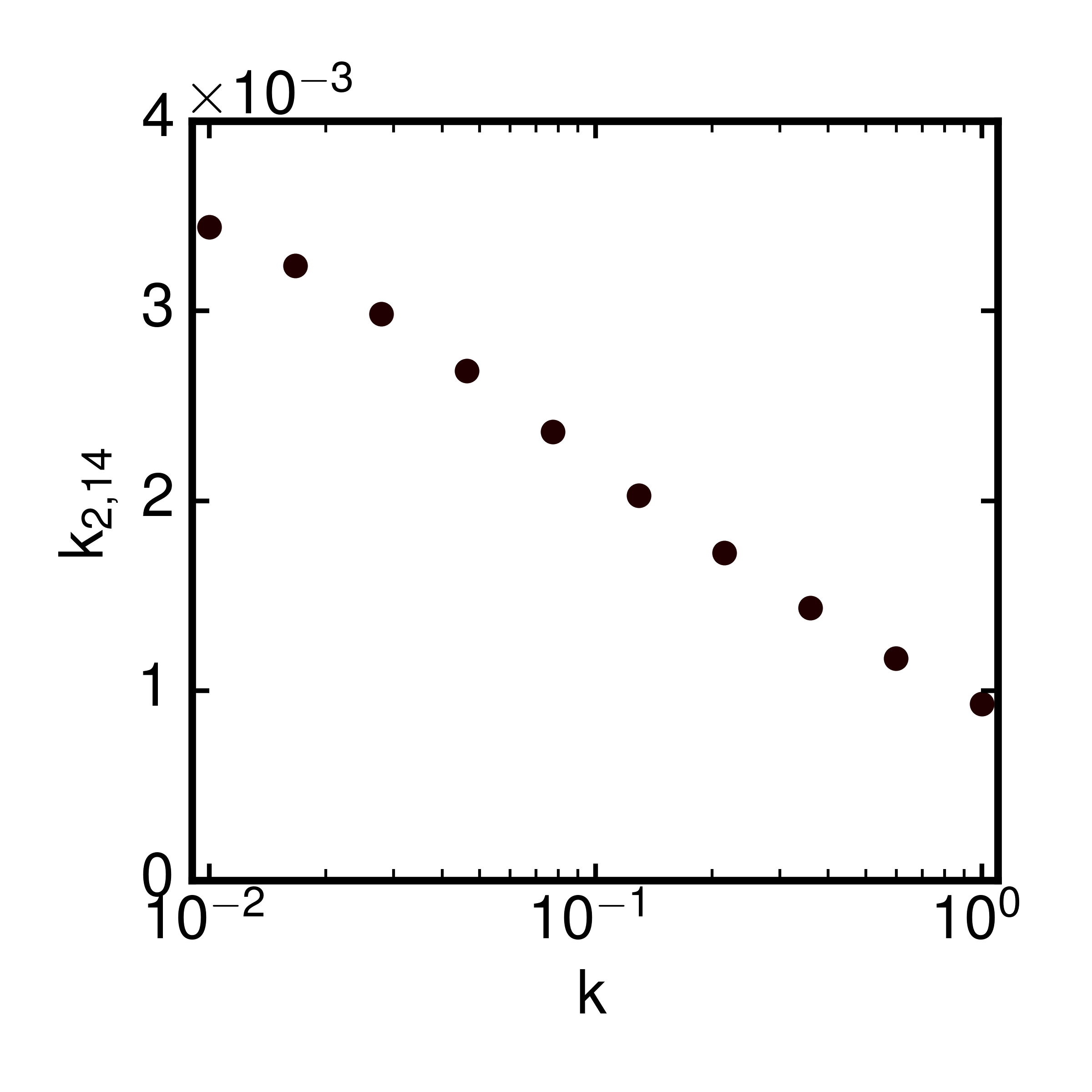

Supplement: Supplementary file 15 — Dataset EV7 [file MSB-13-926-s015.zip › dataset_ev7_cph8-ompr_data_and_analysis/cph8-ompr_analysis/plots/pcs.png_k2.png]

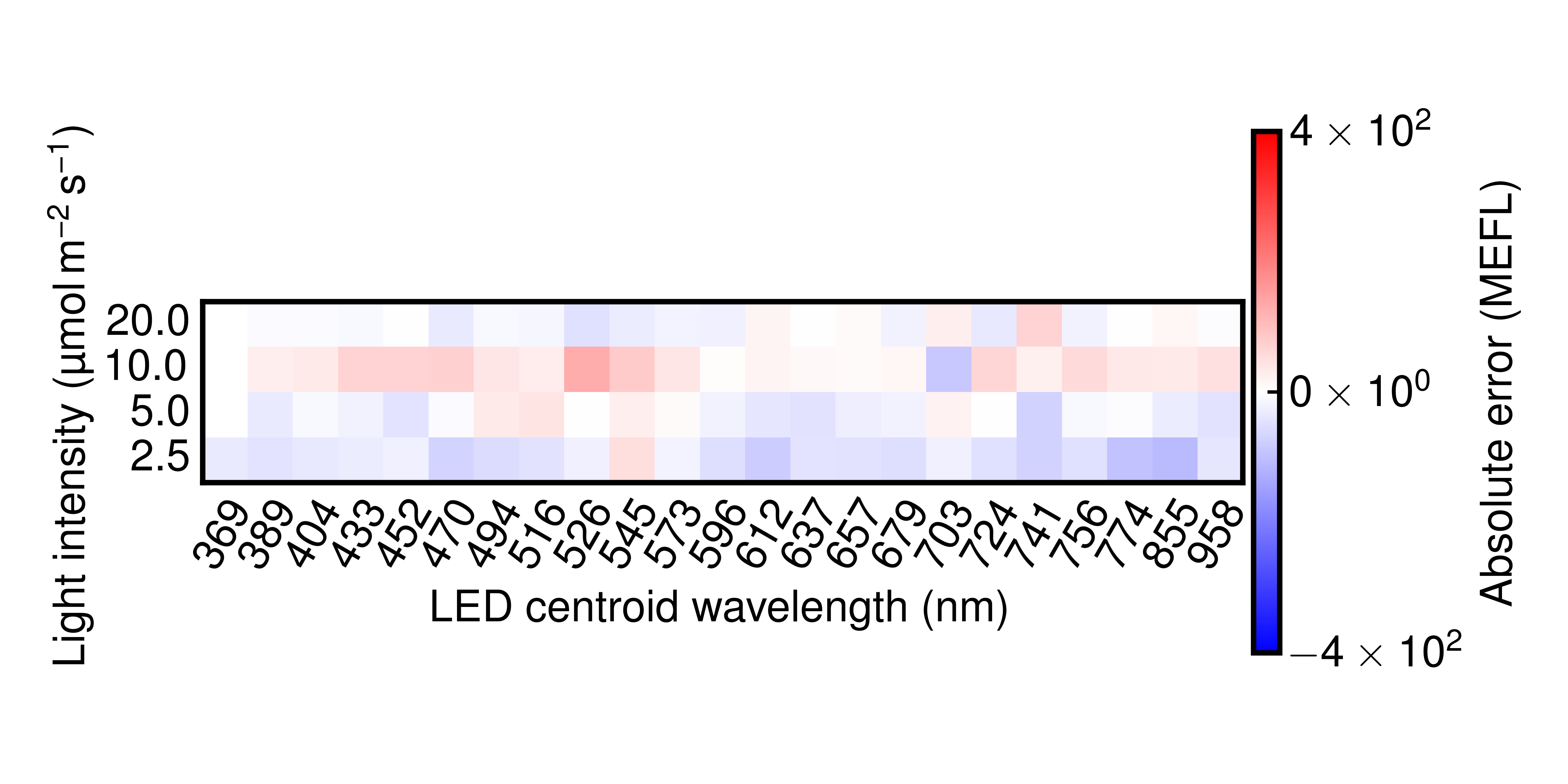

Supplement: Supplementary file 15 — Dataset EV7 [file MSB-13-926-s015.zip › dataset_ev7_cph8-ompr_data_and_analysis/cph8-ompr_analysis/plots/ras_abs_residual_heatmap.png]

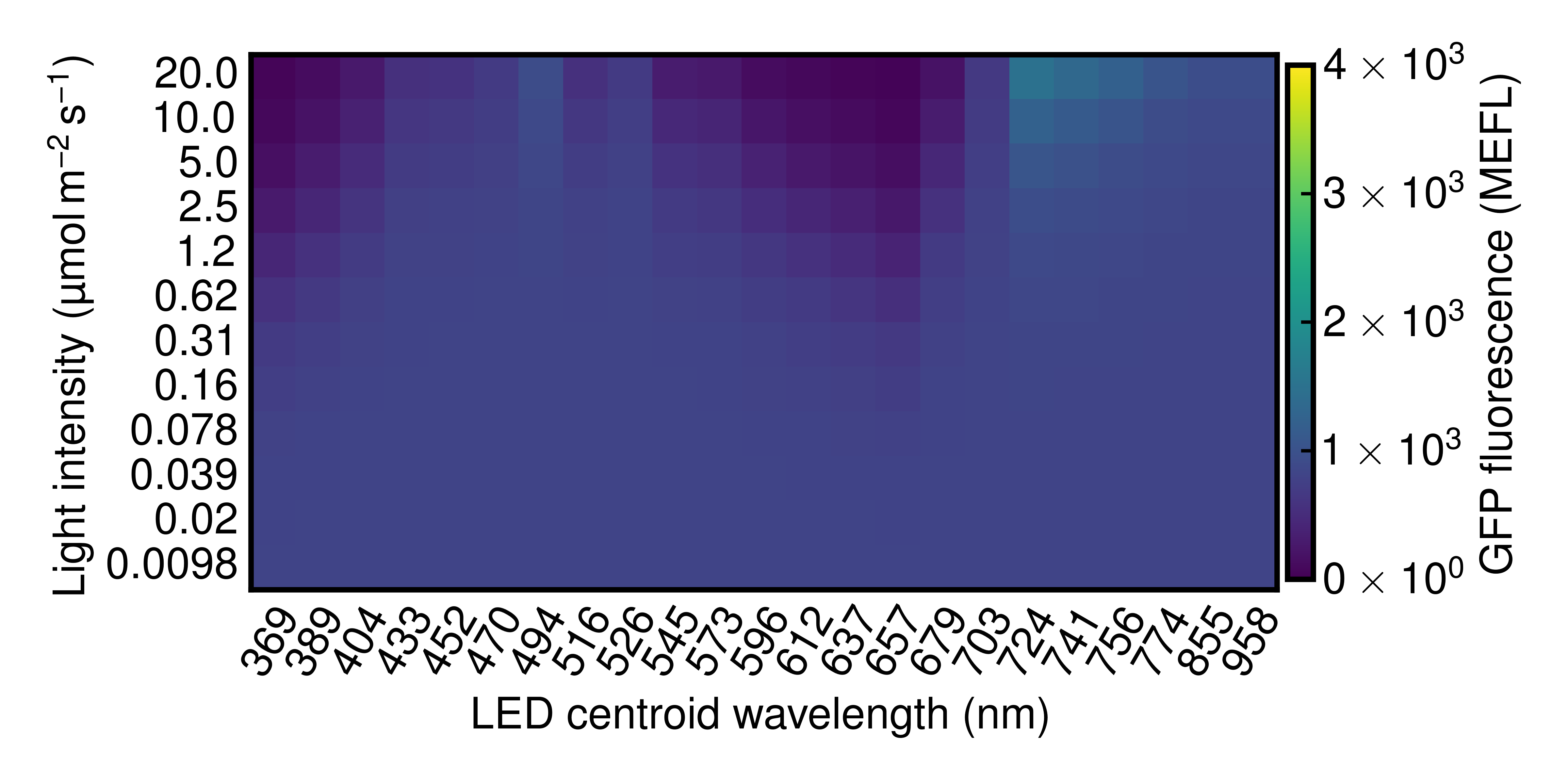

Supplement: Supplementary file 15 — Dataset EV7 [file MSB-13-926-s015.zip › dataset_ev7_cph8-ompr_data_and_analysis/cph8-ompr_analysis/plots/ras_lin_model_heatmap.png]

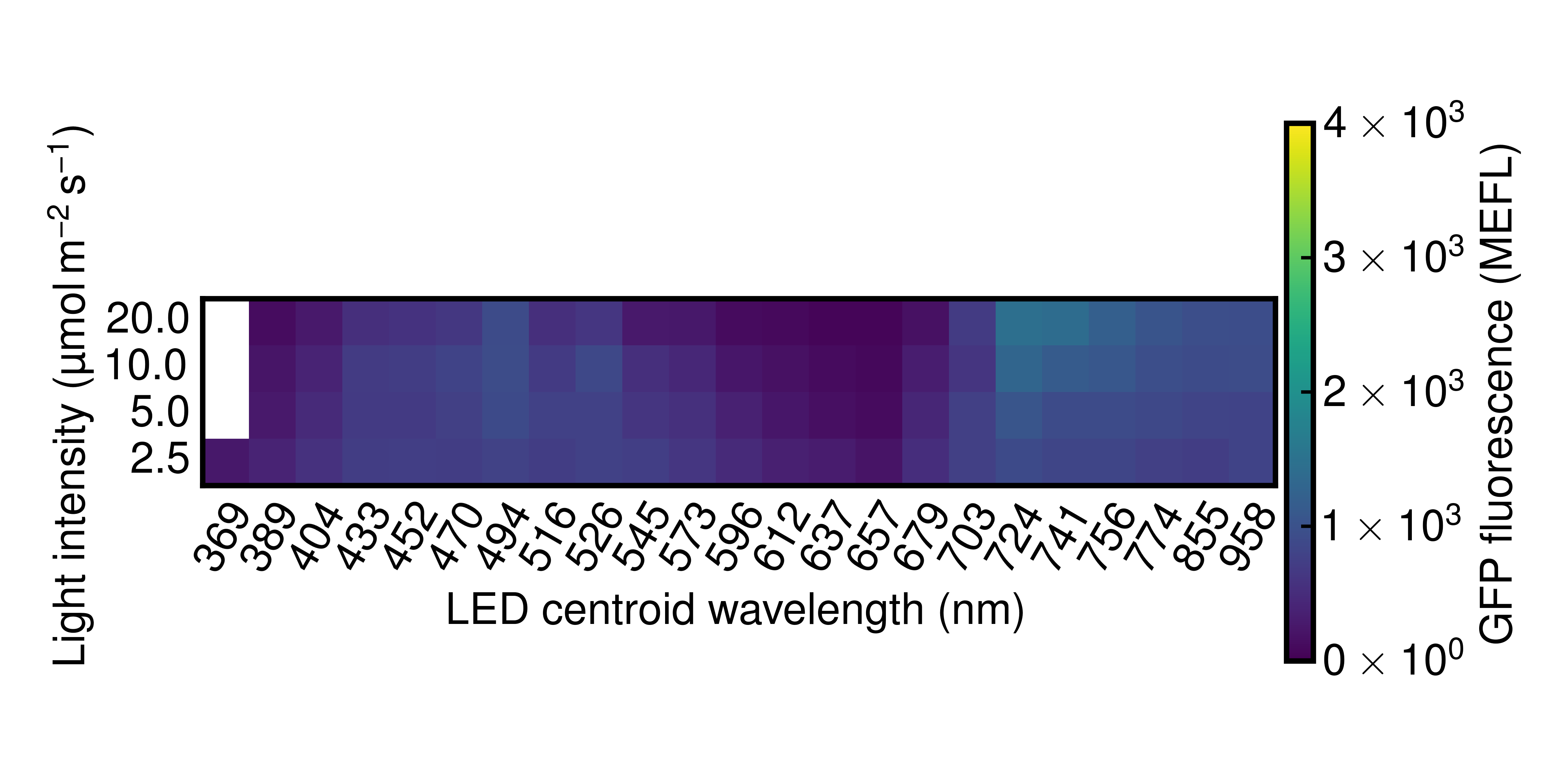

Supplement: Supplementary file 15 — Dataset EV7 [file MSB-13-926-s015.zip › dataset_ev7_cph8-ompr_data_and_analysis/cph8-ompr_analysis/plots/ras_lin_raw_heatmap.png]

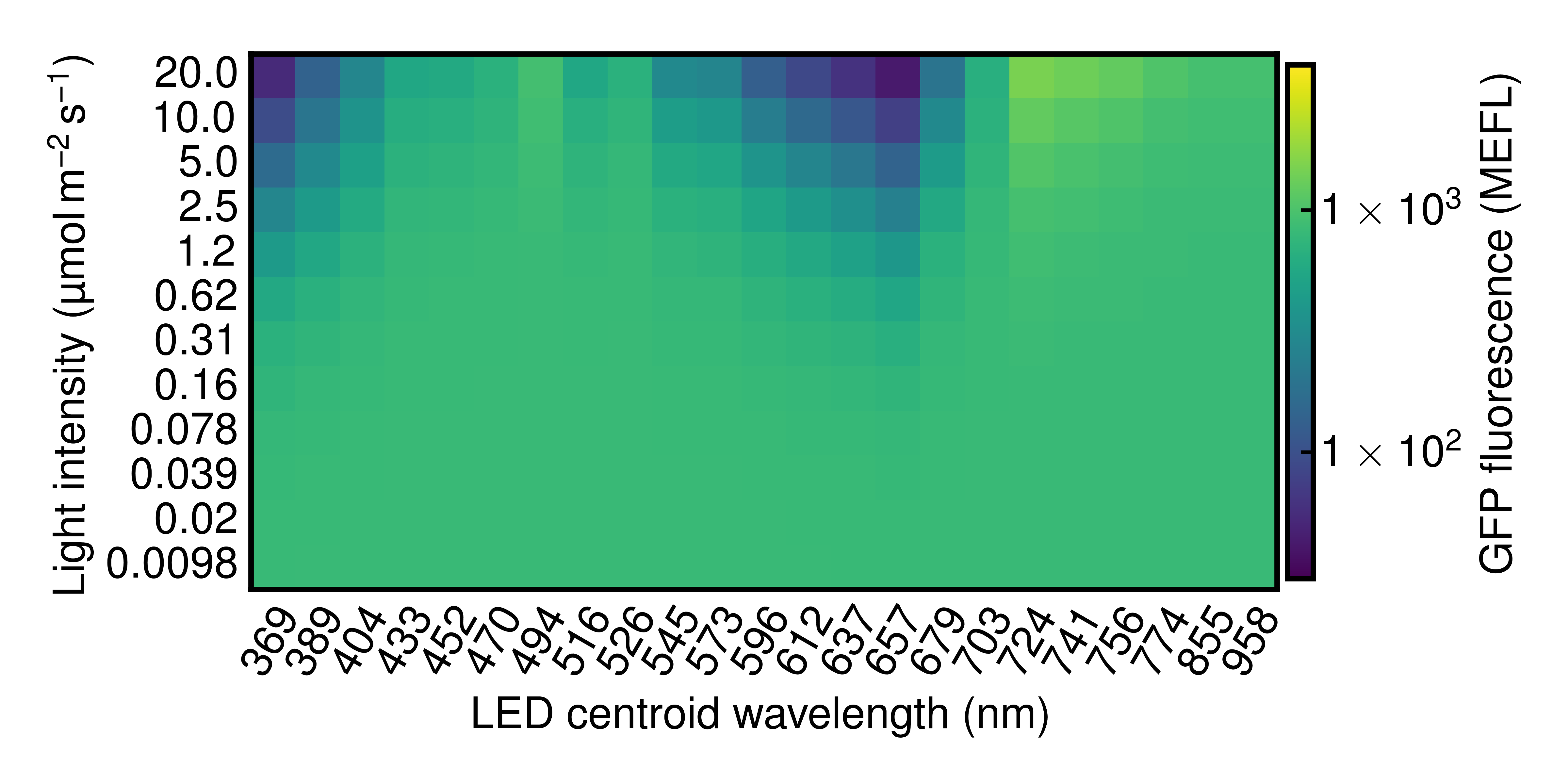

Supplement: Supplementary file 15 — Dataset EV7 [file MSB-13-926-s015.zip › dataset_ev7_cph8-ompr_data_and_analysis/cph8-ompr_analysis/plots/ras_logz_model_heatmap.png]

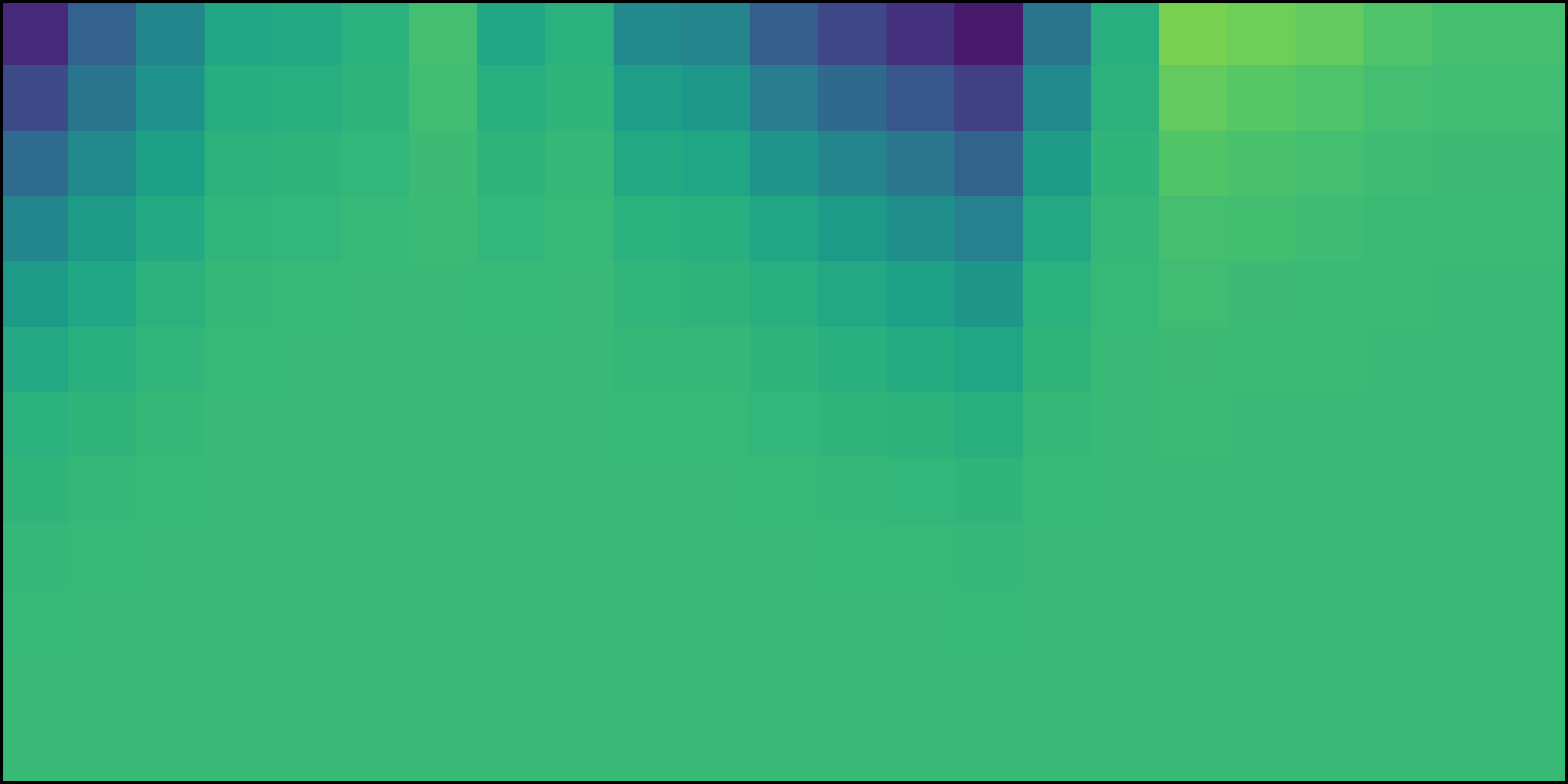

Supplement: Supplementary file 15 — Dataset EV7 [file MSB-13-926-s015.zip › dataset_ev7_cph8-ompr_data_and_analysis/cph8-ompr_analysis/plots/ras_logz_model_nolabel_heatmap.png]

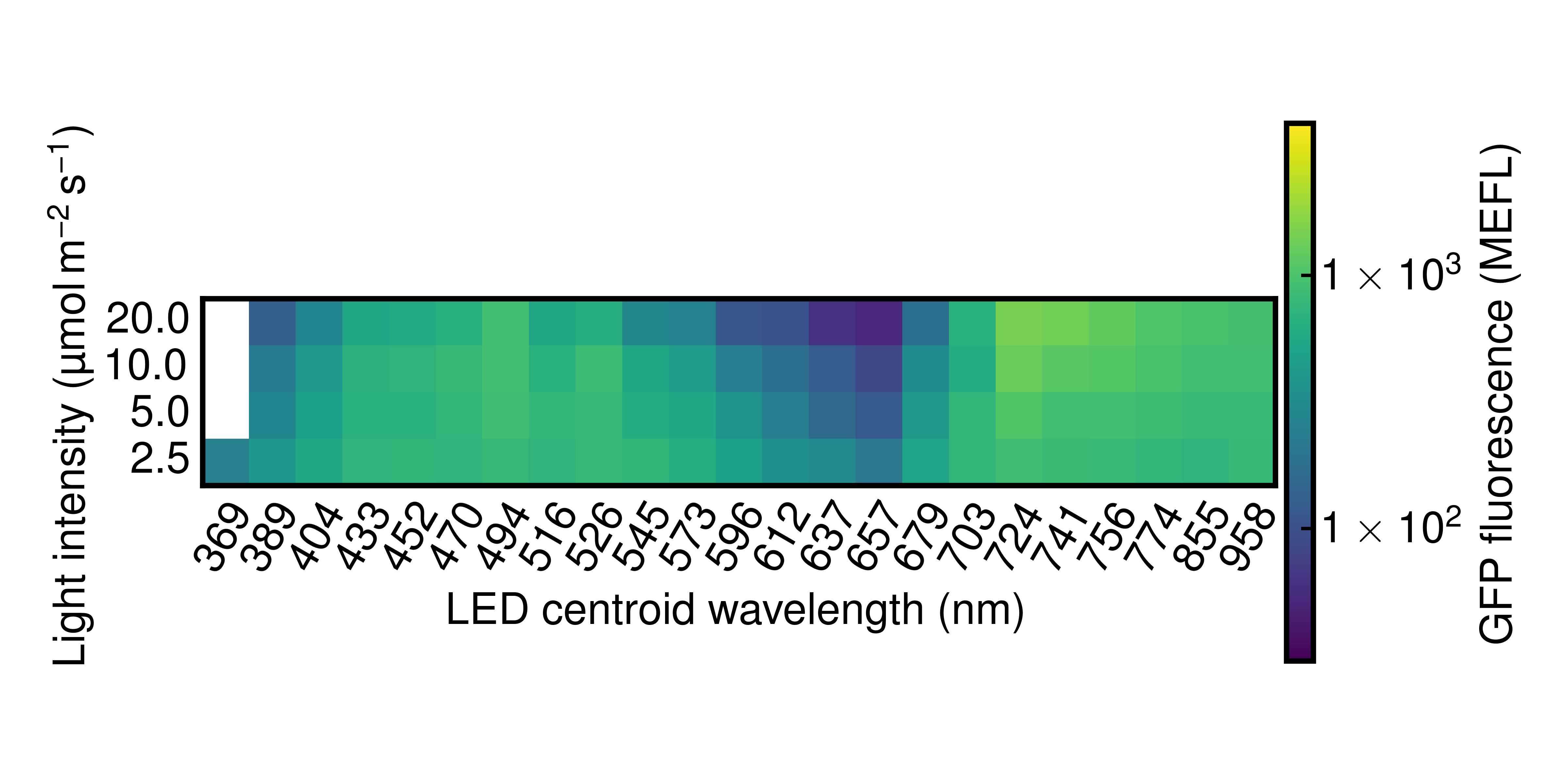

Supplement: Supplementary file 15 — Dataset EV7 [file MSB-13-926-s015.zip › dataset_ev7_cph8-ompr_data_and_analysis/cph8-ompr_analysis/plots/ras_logz_raw_heatmap.png]

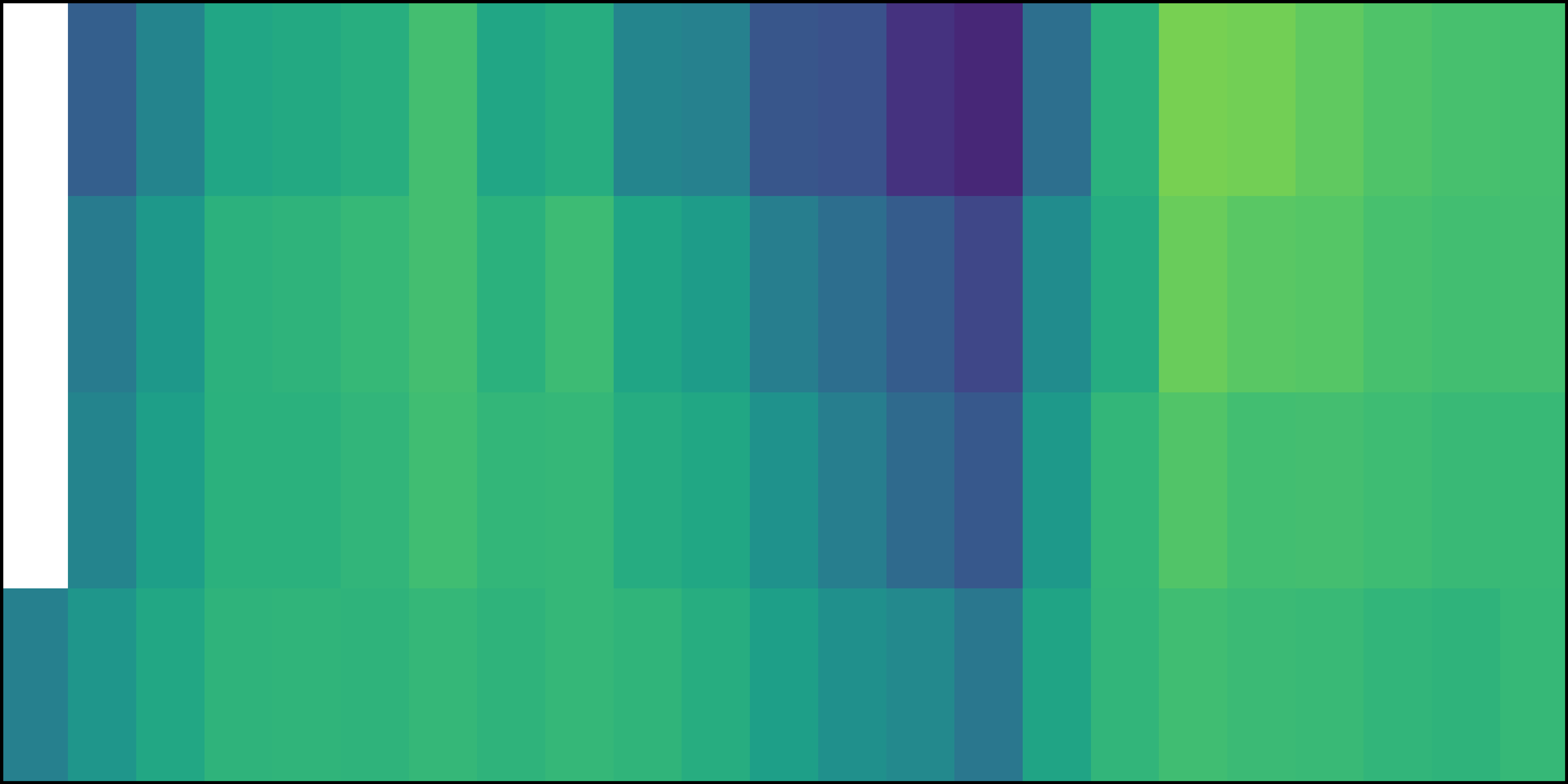

Supplement: Supplementary file 15 — Dataset EV7 [file MSB-13-926-s015.zip › dataset_ev7_cph8-ompr_data_and_analysis/cph8-ompr_analysis/plots/ras_logz_raw_nolabel_heatmap.png]

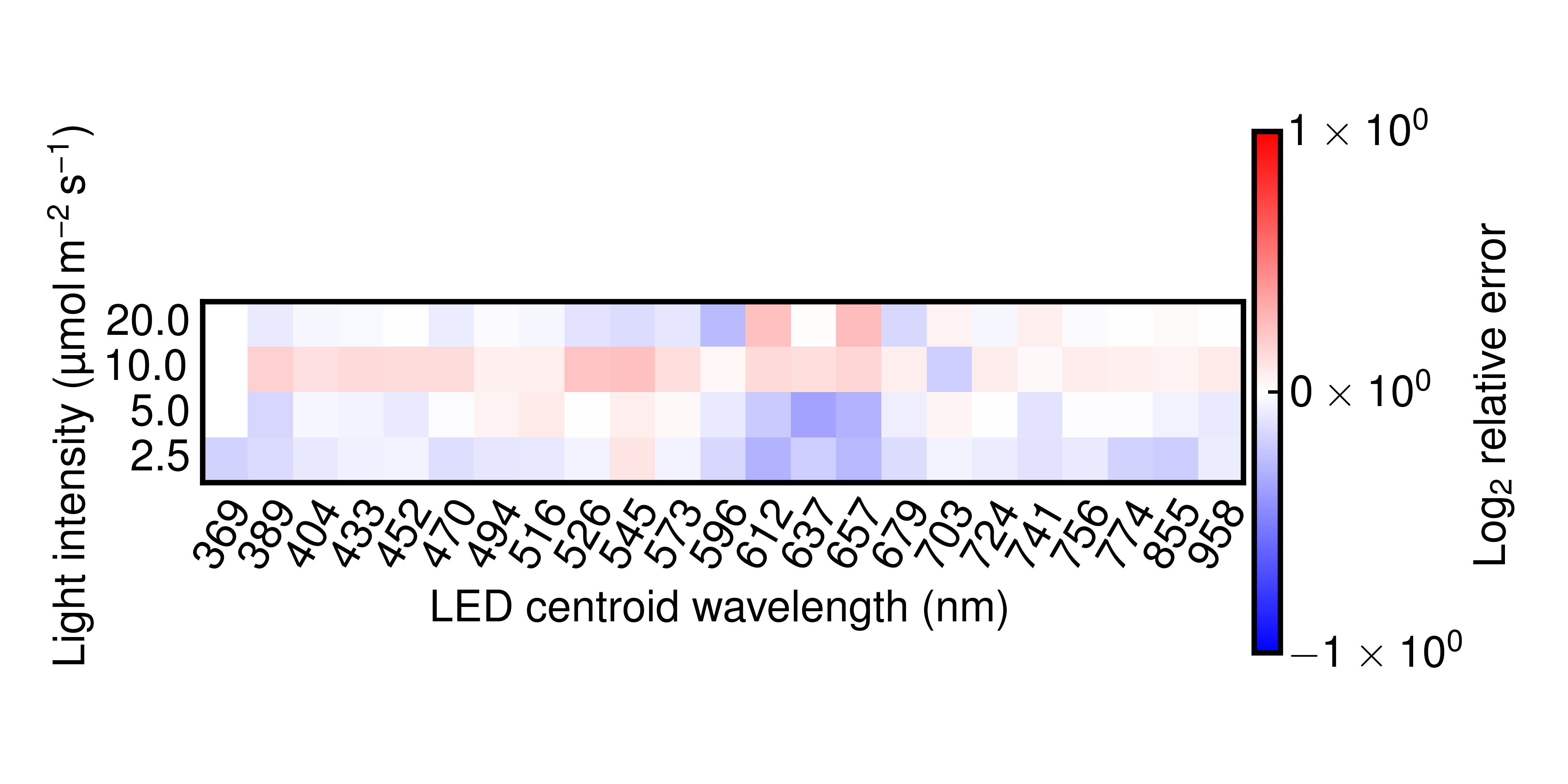

Supplement: Supplementary file 15 — Dataset EV7 [file MSB-13-926-s015.zip › dataset_ev7_cph8-ompr_data_and_analysis/cph8-ompr_analysis/plots/ras_rel_residual_heatmap.png]

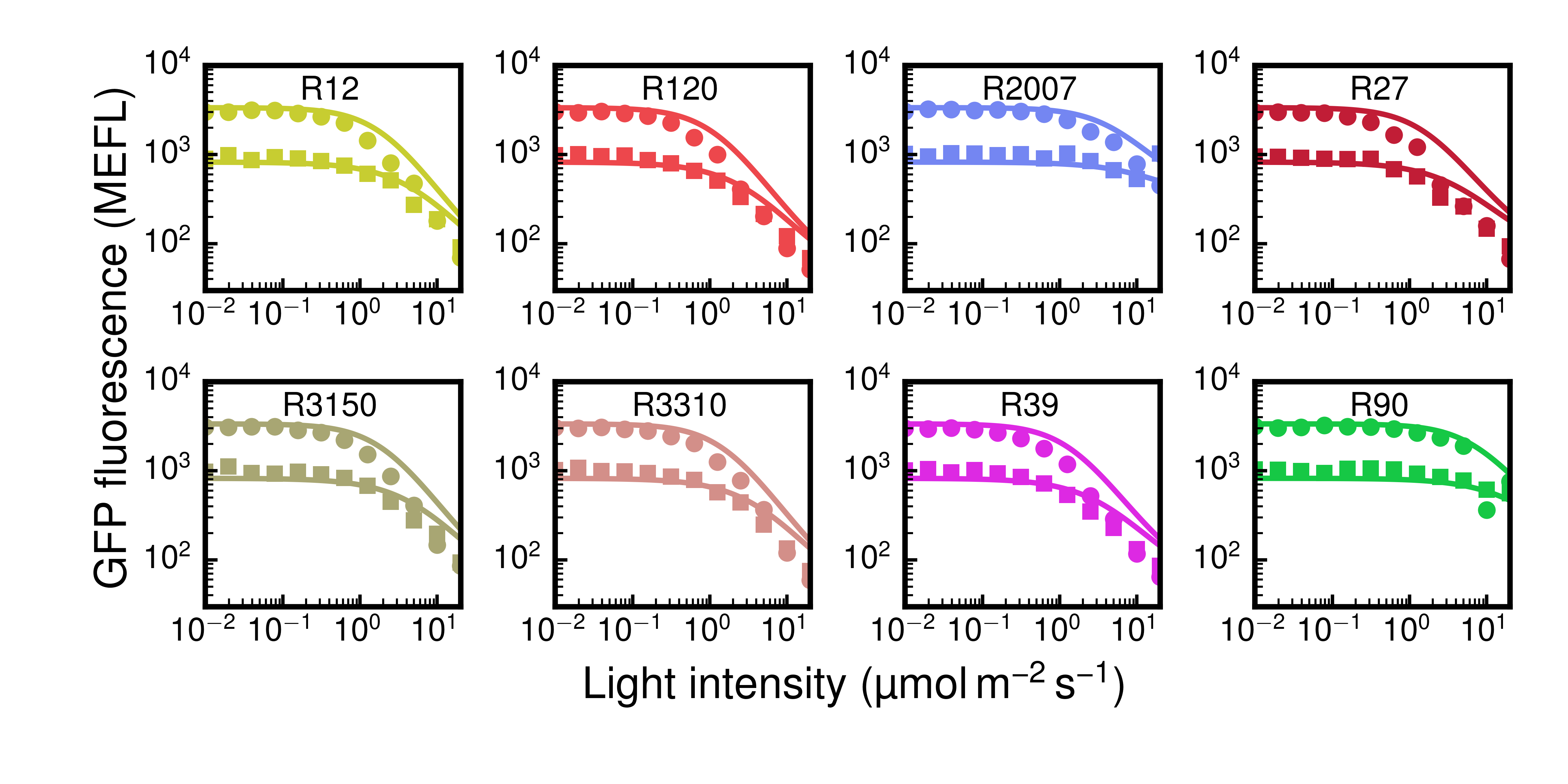

Supplement: Supplementary file 15 — Dataset EV7 [file MSB-13-926-s015.zip › dataset_ev7_cph8-ompr_data_and_analysis/cph8-ompr_analysis/plots/sv_led_fit_1_log.png]

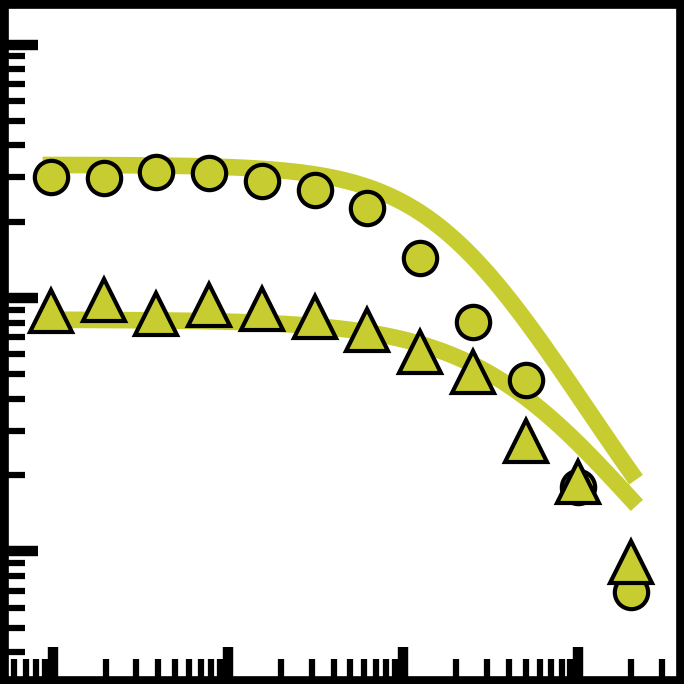

Supplement: Supplementary file 15 — Dataset EV7 [file MSB-13-926-s015.zip › dataset_ev7_cph8-ompr_data_and_analysis/cph8-ompr_analysis/plots/sv_led_fit_R12.png]
