# Supplementary figures and images for: A photoconversion model for full spectral programming and multiplexing of optogenetic systems (part 3 of 3)
Source: Mol Syst Biol. 2017 Apr 24;13(4):926. doi: 10.15252/msb.20167456 (PMC5408778; doi:10.15252/msb.20167456)

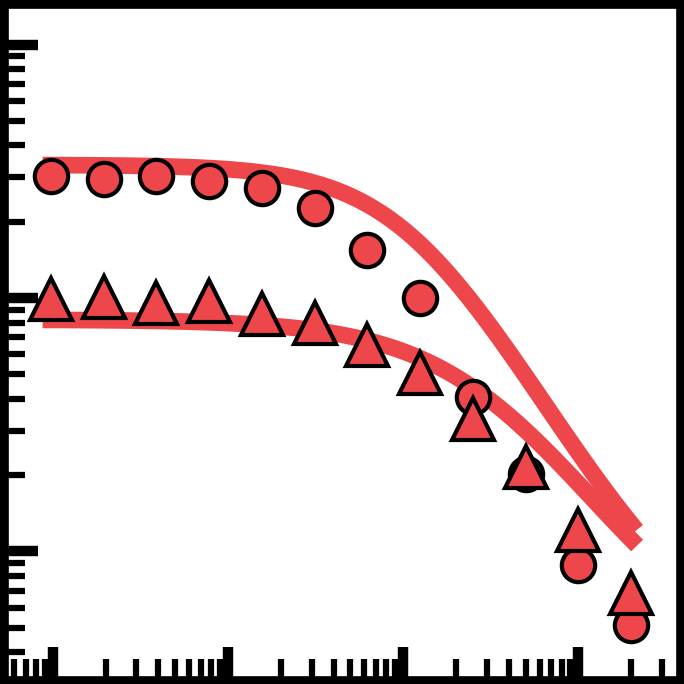

Supplement: Supplementary file 15 — Dataset EV7 [file MSB-13-926-s015.zip › dataset_ev7_cph8-ompr_data_and_analysis/cph8-ompr_analysis/plots/sv_led_fit_R120.png]

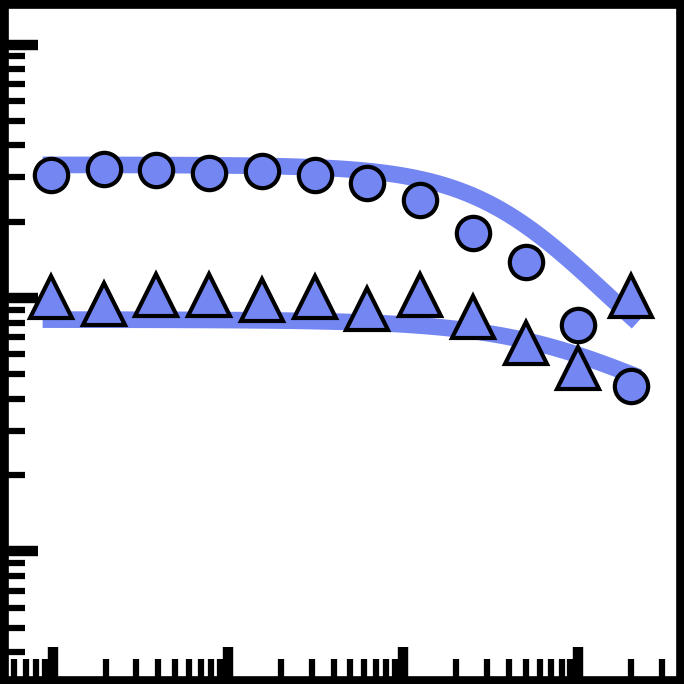

Supplement: Supplementary file 15 — Dataset EV7 [file MSB-13-926-s015.zip › dataset_ev7_cph8-ompr_data_and_analysis/cph8-ompr_analysis/plots/sv_led_fit_R2007.png]

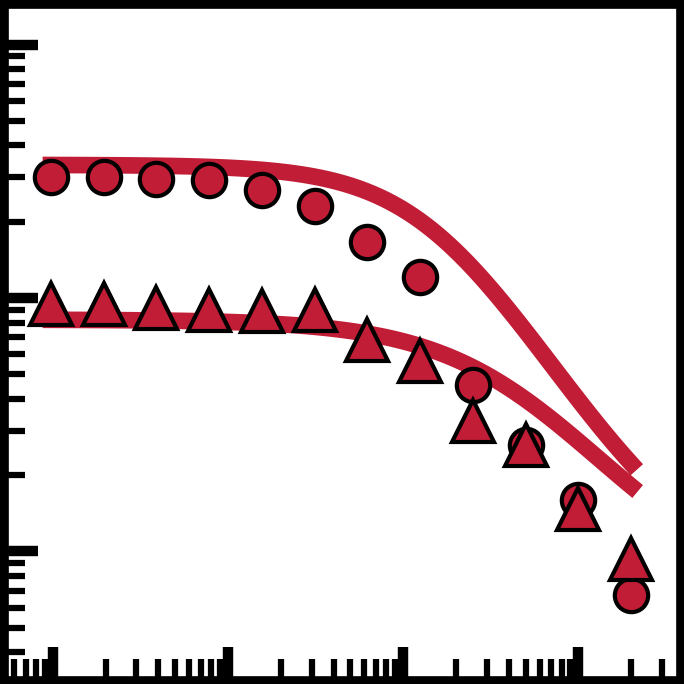

Supplement: Supplementary file 15 — Dataset EV7 [file MSB-13-926-s015.zip › dataset_ev7_cph8-ompr_data_and_analysis/cph8-ompr_analysis/plots/sv_led_fit_R27.png]

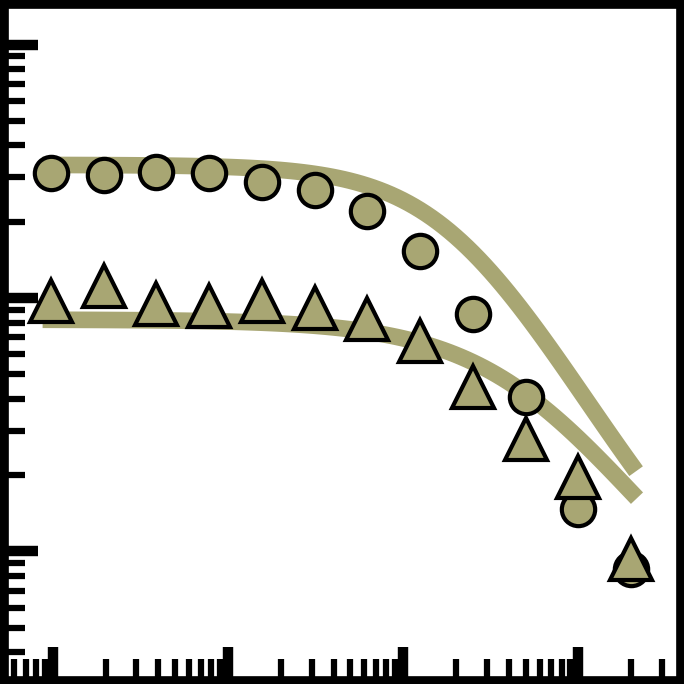

Supplement: Supplementary file 15 — Dataset EV7 [file MSB-13-926-s015.zip › dataset_ev7_cph8-ompr_data_and_analysis/cph8-ompr_analysis/plots/sv_led_fit_R3150.png]

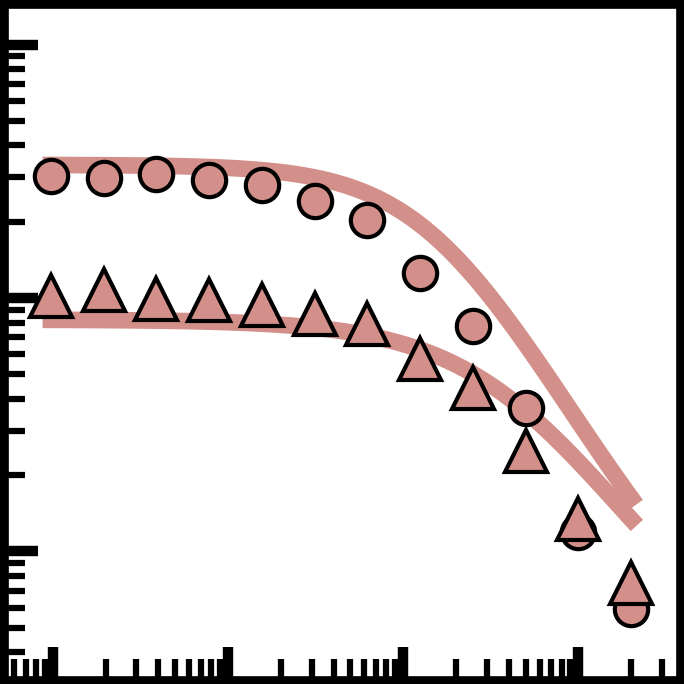

Supplement: Supplementary file 15 — Dataset EV7 [file MSB-13-926-s015.zip › dataset_ev7_cph8-ompr_data_and_analysis/cph8-ompr_analysis/plots/sv_led_fit_R3310.png]

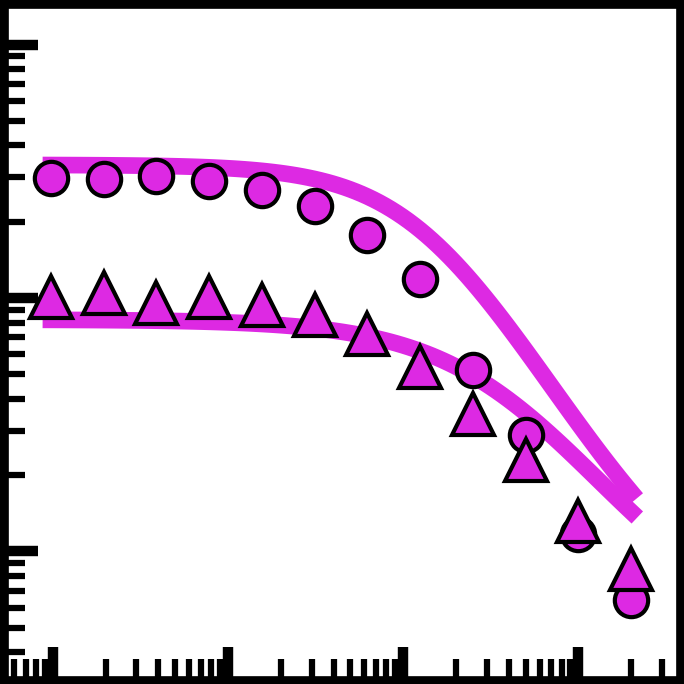

Supplement: Supplementary file 15 — Dataset EV7 [file MSB-13-926-s015.zip › dataset_ev7_cph8-ompr_data_and_analysis/cph8-ompr_analysis/plots/sv_led_fit_R39.png]

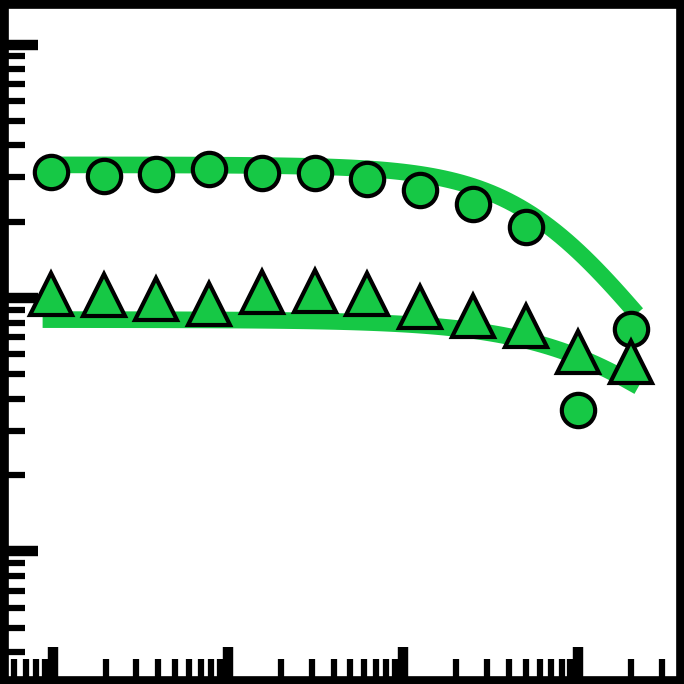

Supplement: Supplementary file 15 — Dataset EV7 [file MSB-13-926-s015.zip › dataset_ev7_cph8-ompr_data_and_analysis/cph8-ompr_analysis/plots/sv_led_fit_R90.png]

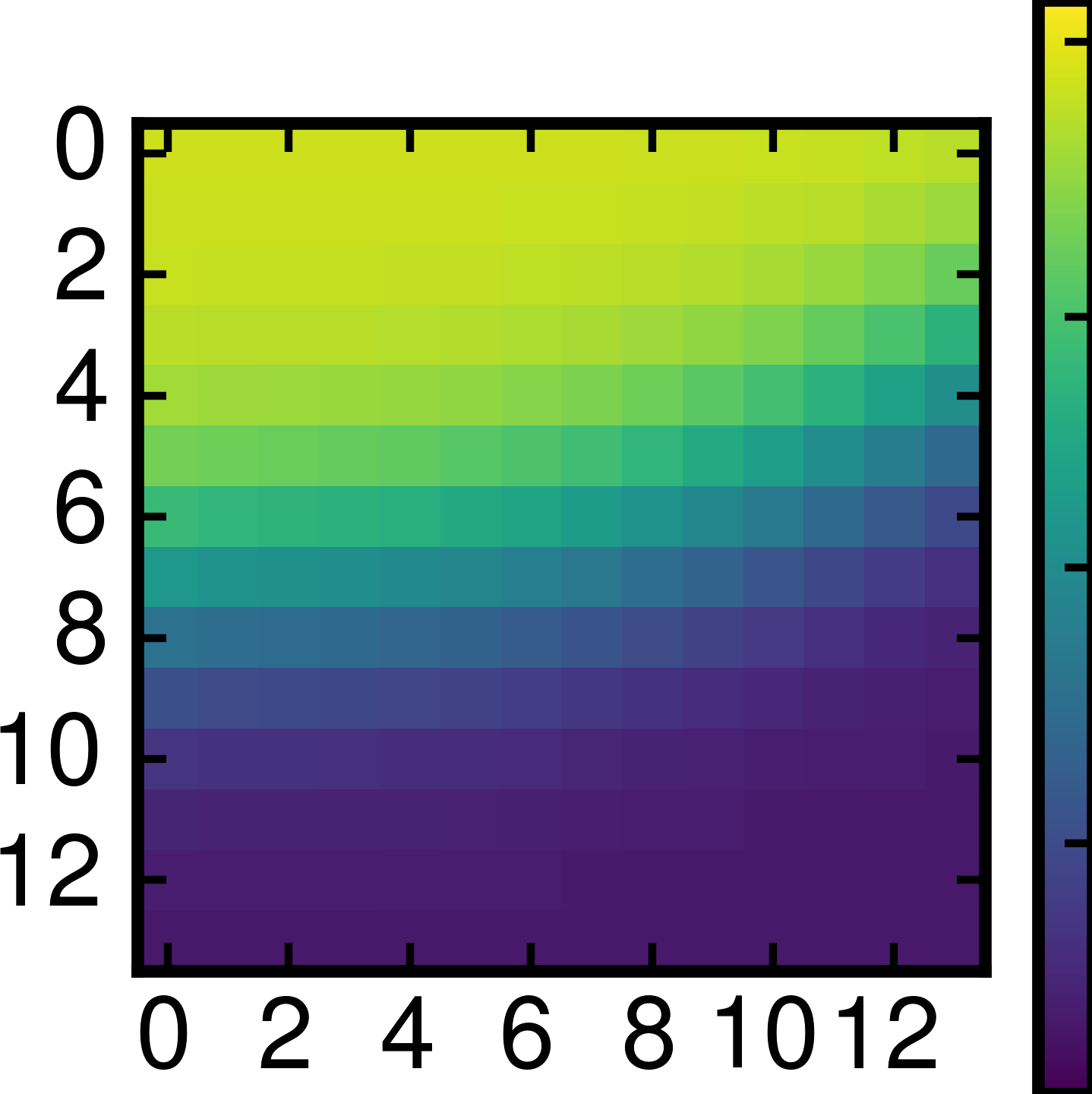

Supplement: Supplementary file 16 — Dataset EV8 [file MSB-13-926-s016.zip › dataset_ev8_mux_data_and_analysis/mux_analysis/plots/cbar_gfp.png]

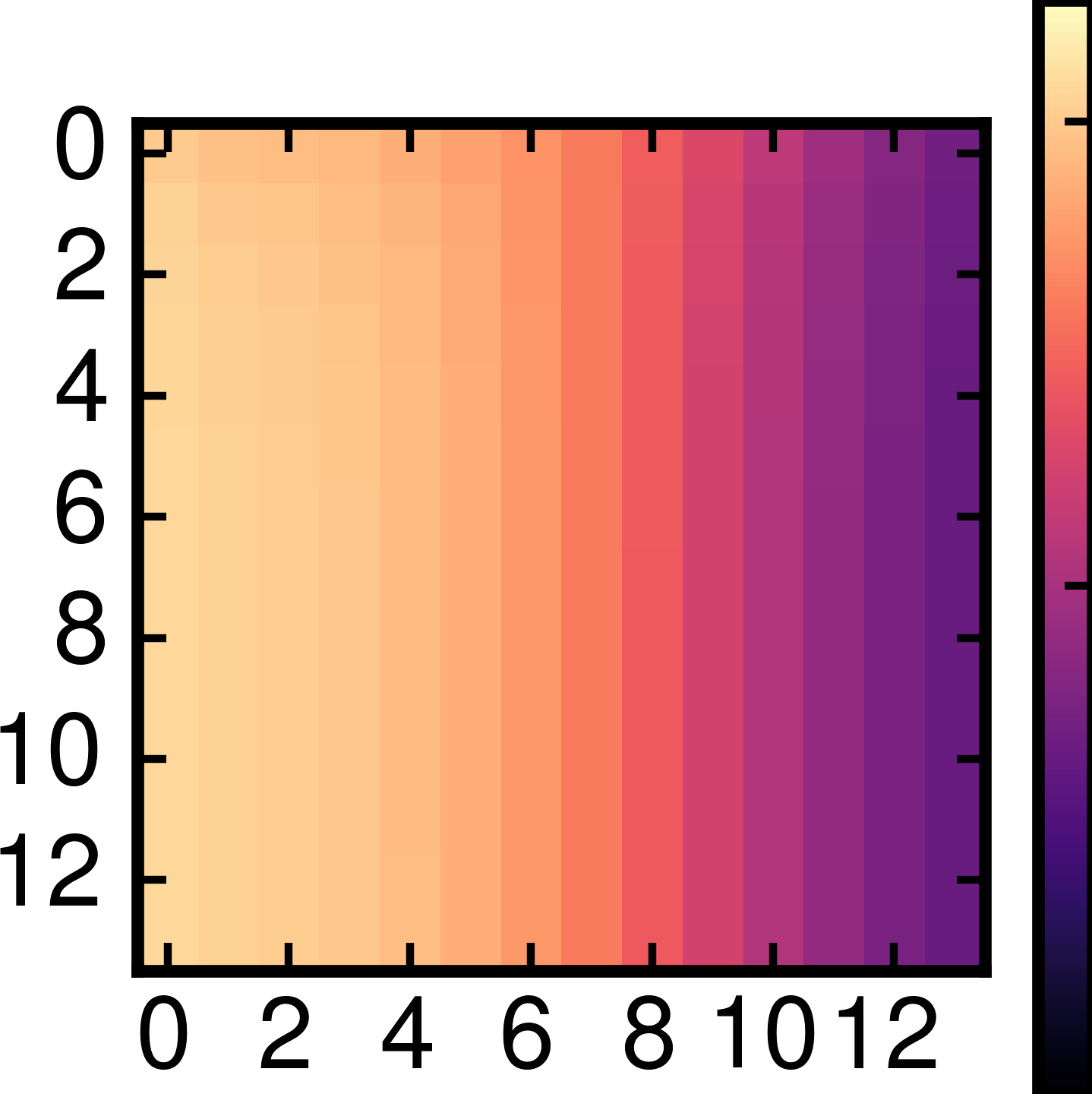

Supplement: Supplementary file 16 — Dataset EV8 [file MSB-13-926-s016.zip › dataset_ev8_mux_data_and_analysis/mux_analysis/plots/cbar_mch.png]

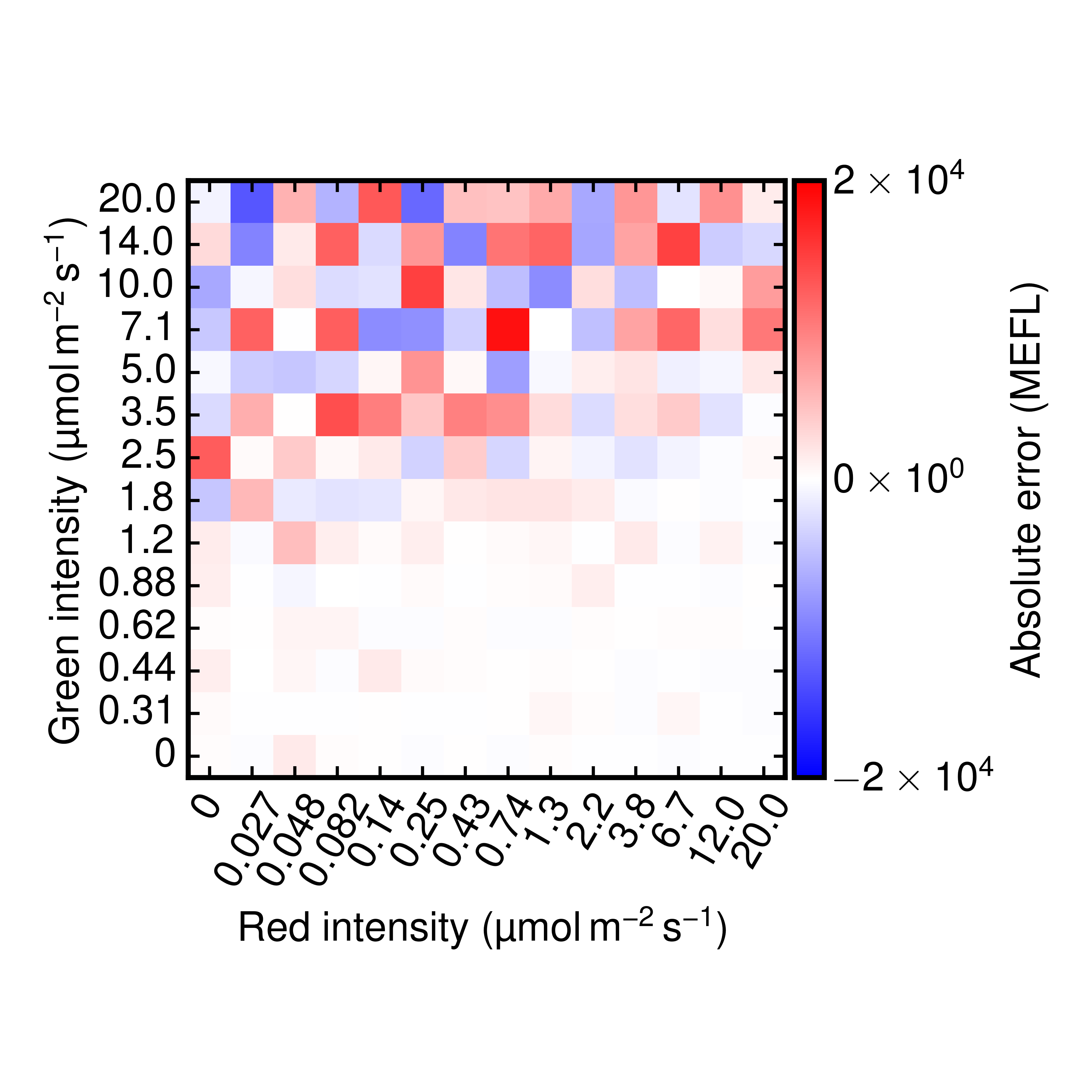

Supplement: Supplementary file 16 — Dataset EV8 [file MSB-13-926-s016.zip › dataset_ev8_mux_data_and_analysis/mux_analysis/plots/gfp_abs_residual_hmap.png]

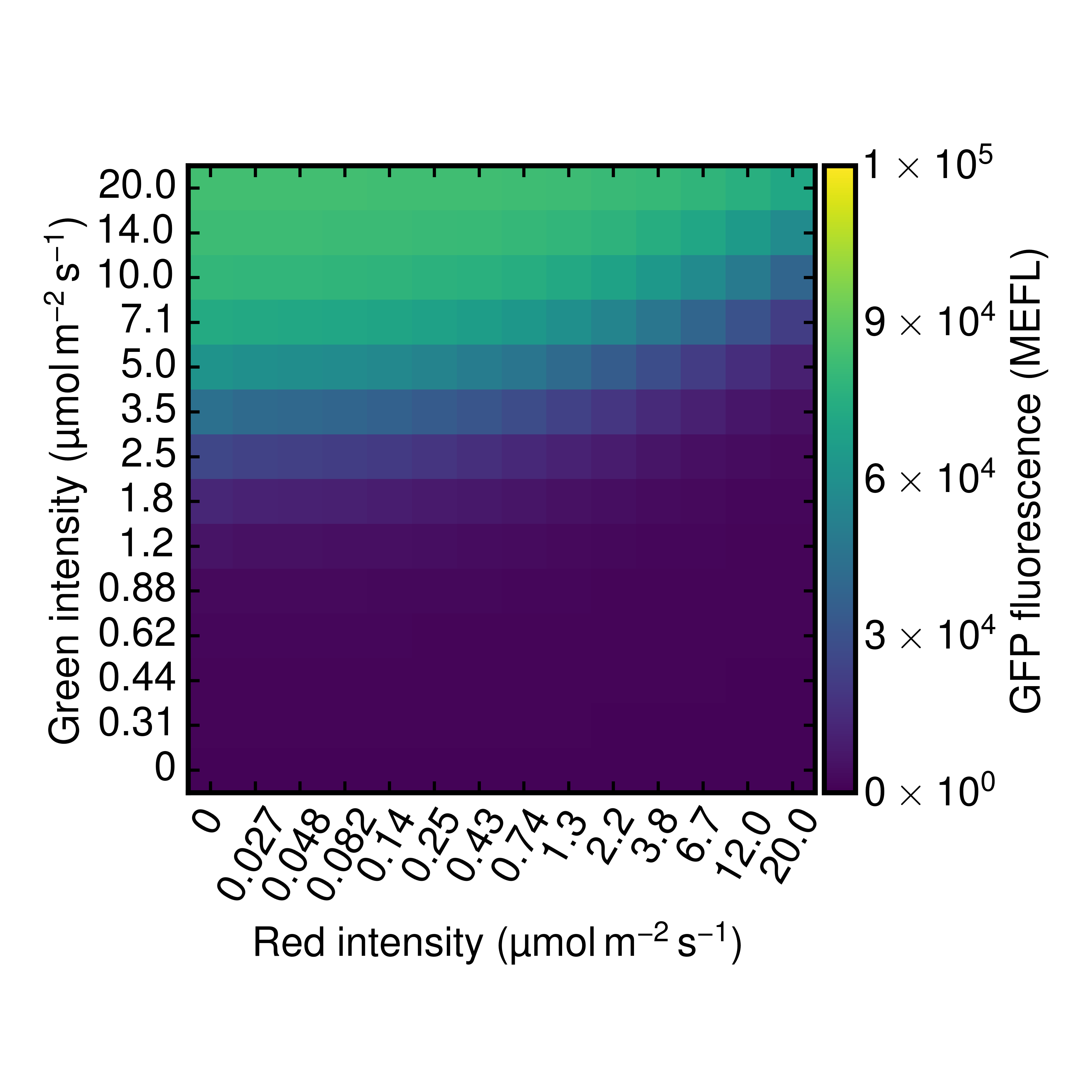

Supplement: Supplementary file 16 — Dataset EV8 [file MSB-13-926-s016.zip › dataset_ev8_mux_data_and_analysis/mux_analysis/plots/gfp_lin_model_heatmap.png]

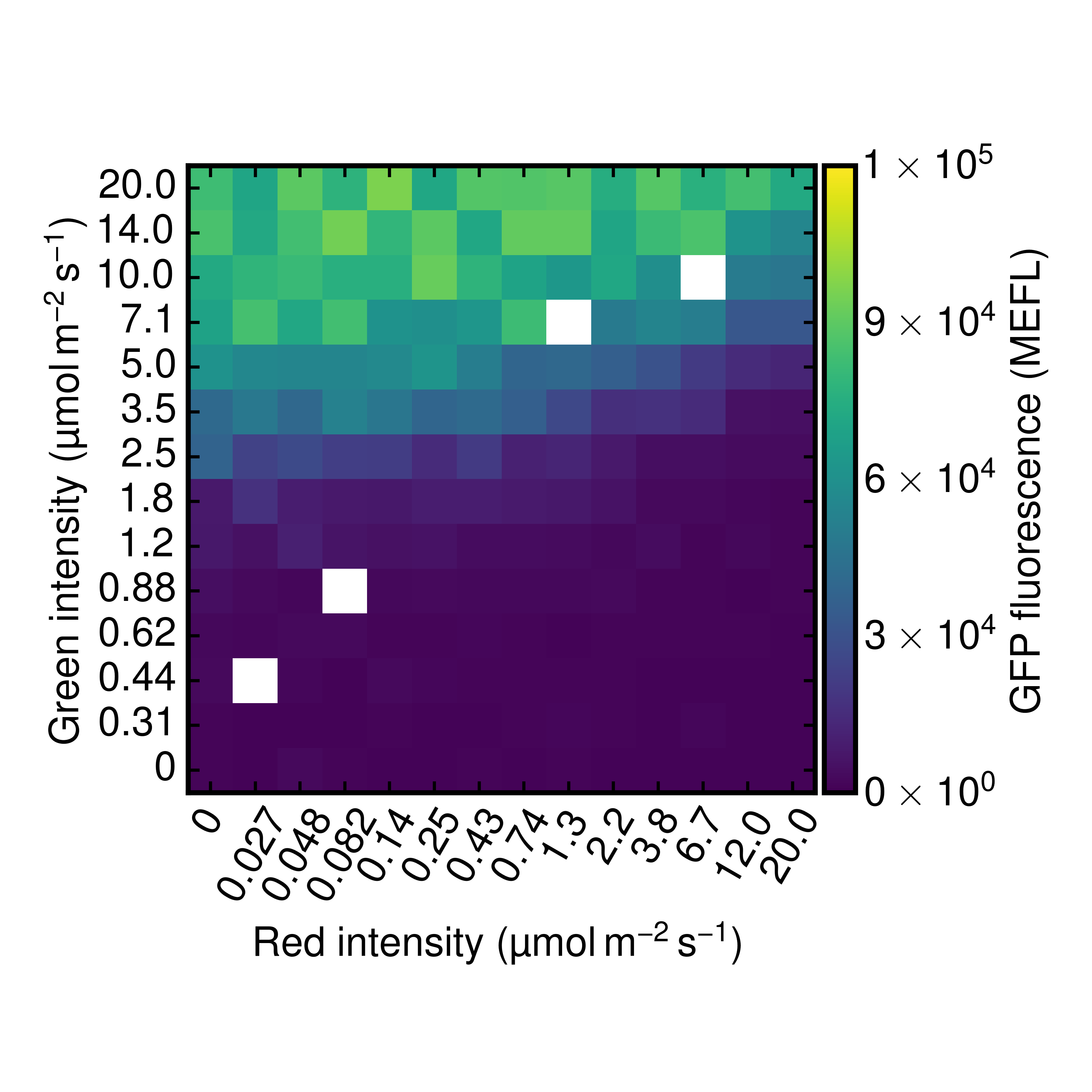

Supplement: Supplementary file 16 — Dataset EV8 [file MSB-13-926-s016.zip › dataset_ev8_mux_data_and_analysis/mux_analysis/plots/gfp_lin_raw_heatmap.png]

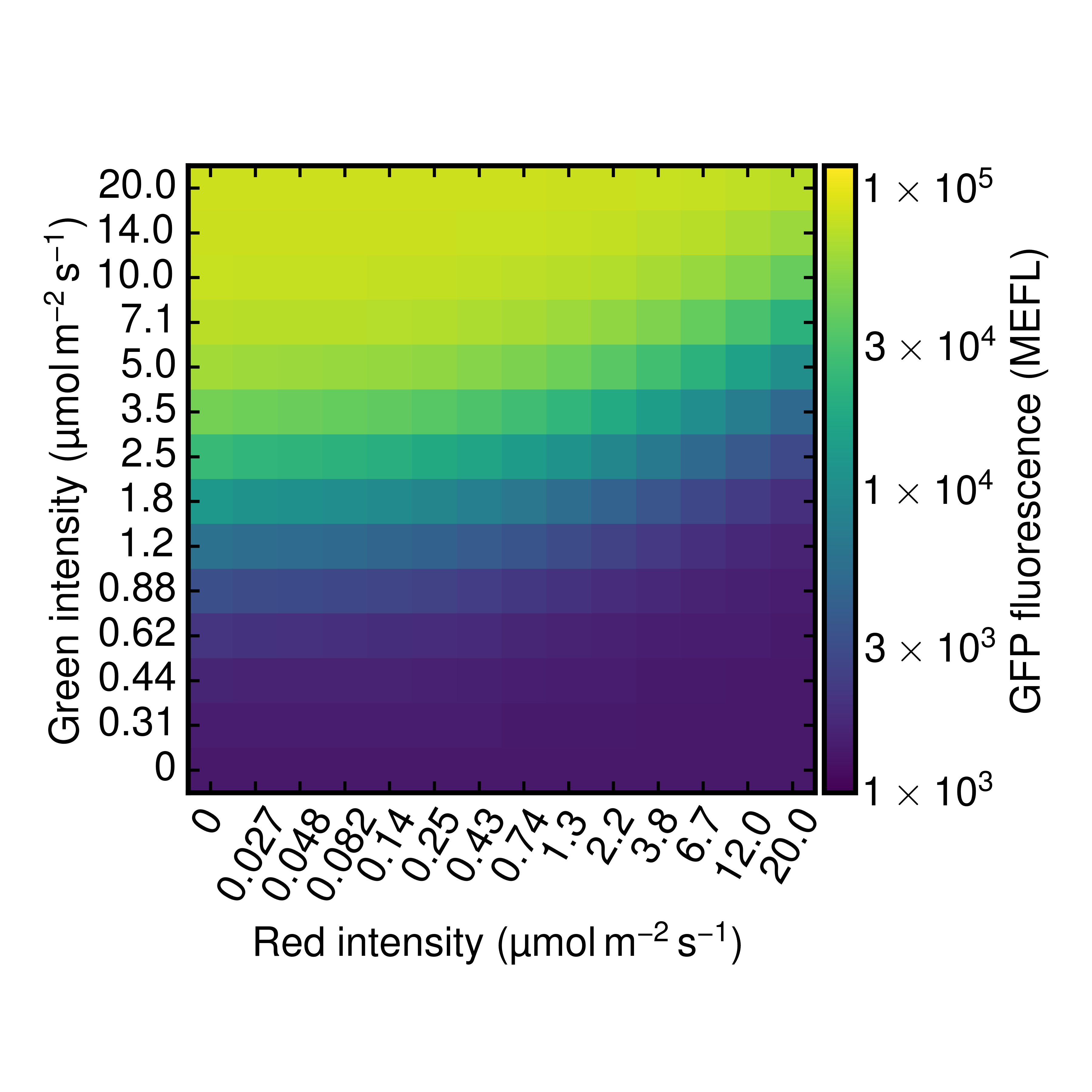

Supplement: Supplementary file 16 — Dataset EV8 [file MSB-13-926-s016.zip › dataset_ev8_mux_data_and_analysis/mux_analysis/plots/gfp_logz_model_heatmap.png]

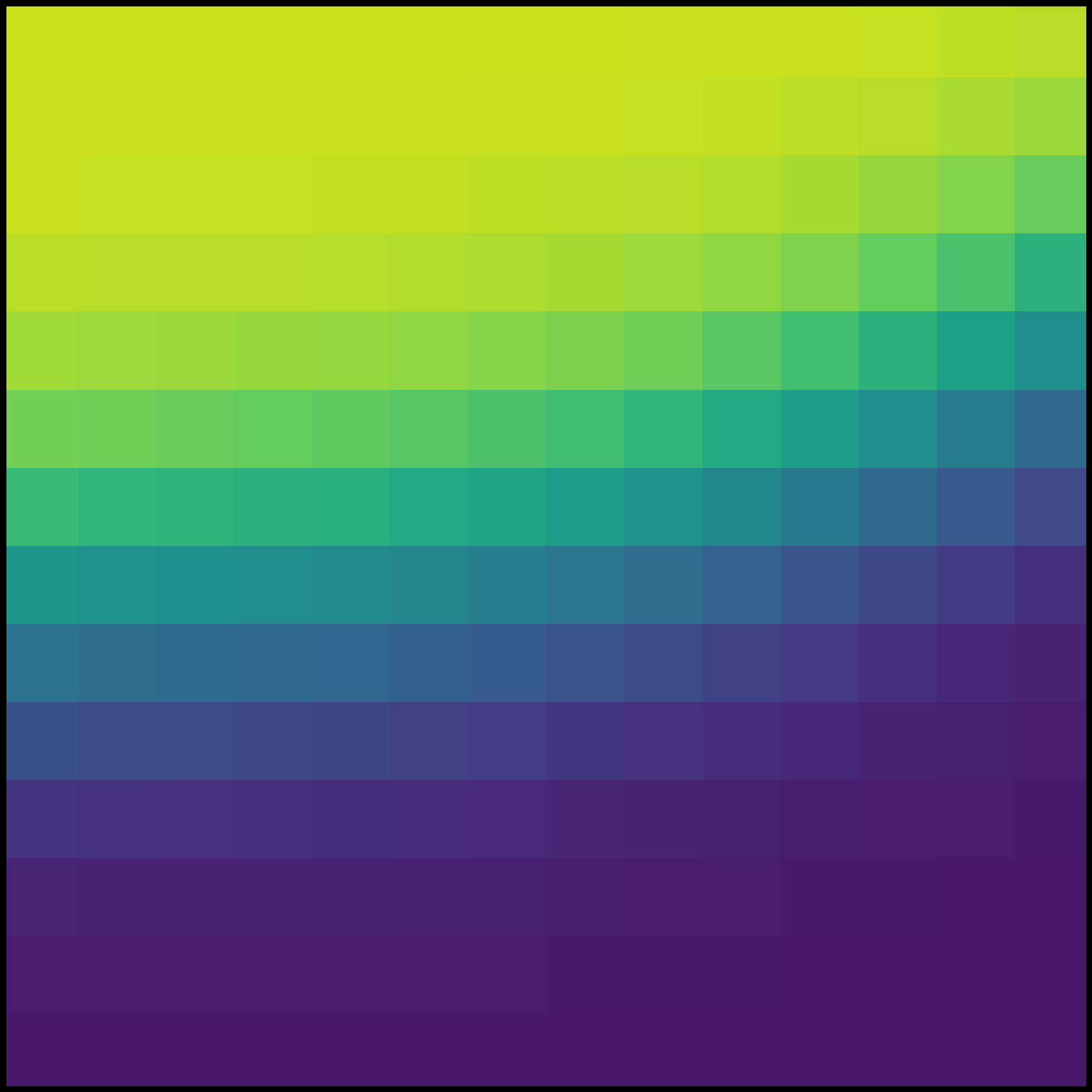

Supplement: Supplementary file 16 — Dataset EV8 [file MSB-13-926-s016.zip › dataset_ev8_mux_data_and_analysis/mux_analysis/plots/gfp_logz_model_nolabel_heatmap.png]

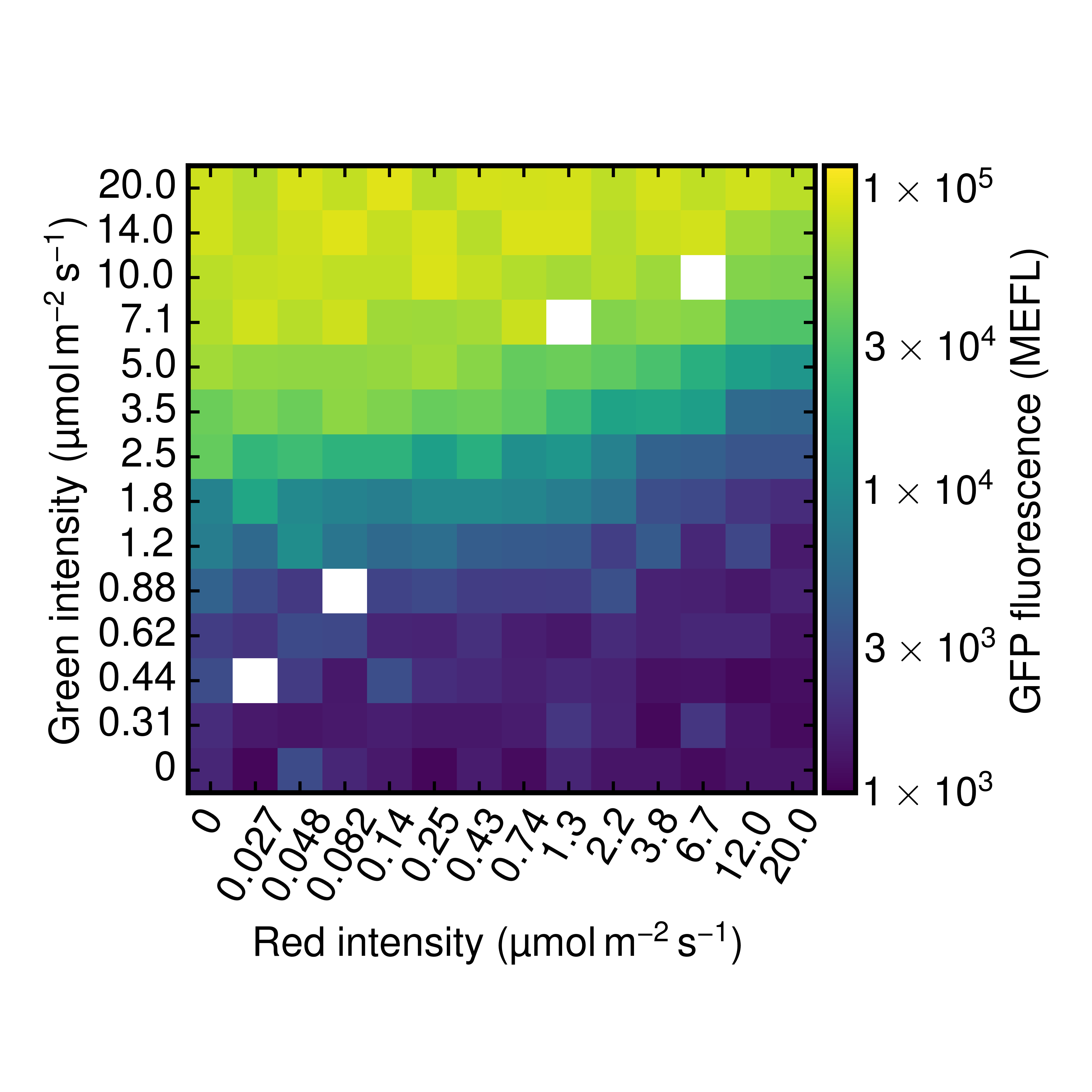

Supplement: Supplementary file 16 — Dataset EV8 [file MSB-13-926-s016.zip › dataset_ev8_mux_data_and_analysis/mux_analysis/plots/gfp_logz_raw_heatmap.png]

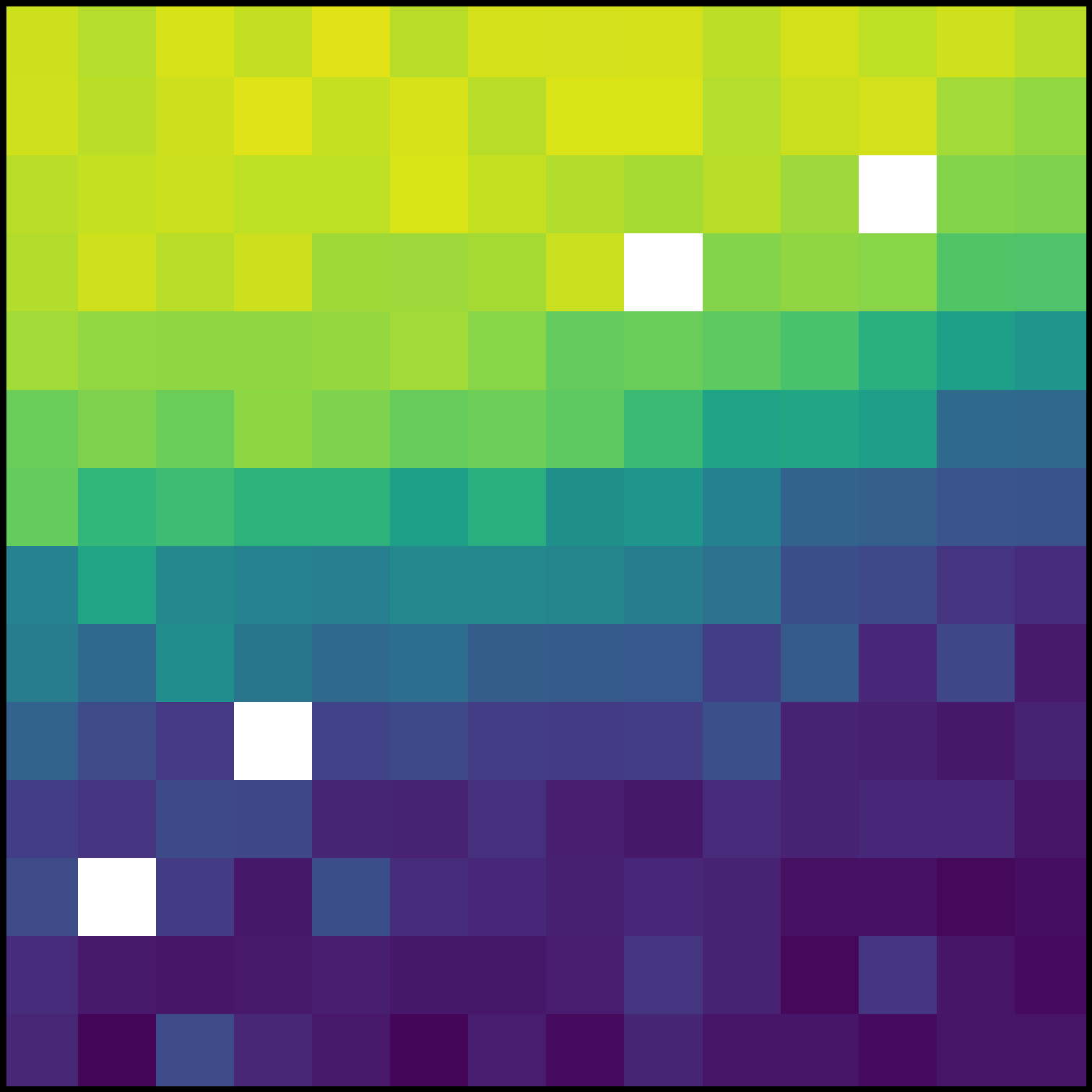

Supplement: Supplementary file 16 — Dataset EV8 [file MSB-13-926-s016.zip › dataset_ev8_mux_data_and_analysis/mux_analysis/plots/gfp_logz_raw_nolabel_heatmap.png]

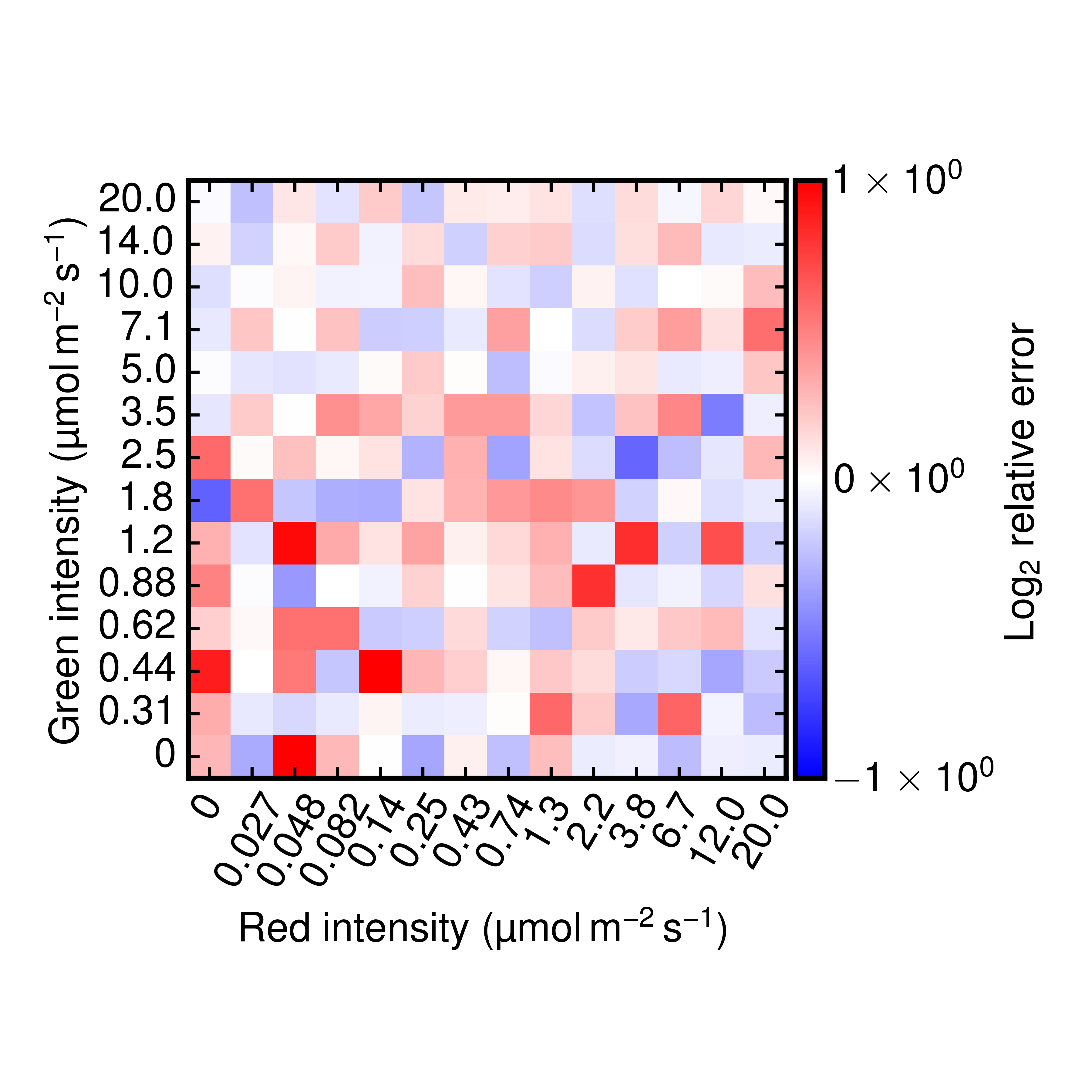

Supplement: Supplementary file 16 — Dataset EV8 [file MSB-13-926-s016.zip › dataset_ev8_mux_data_and_analysis/mux_analysis/plots/gfp_rel_residual_hmap.png]

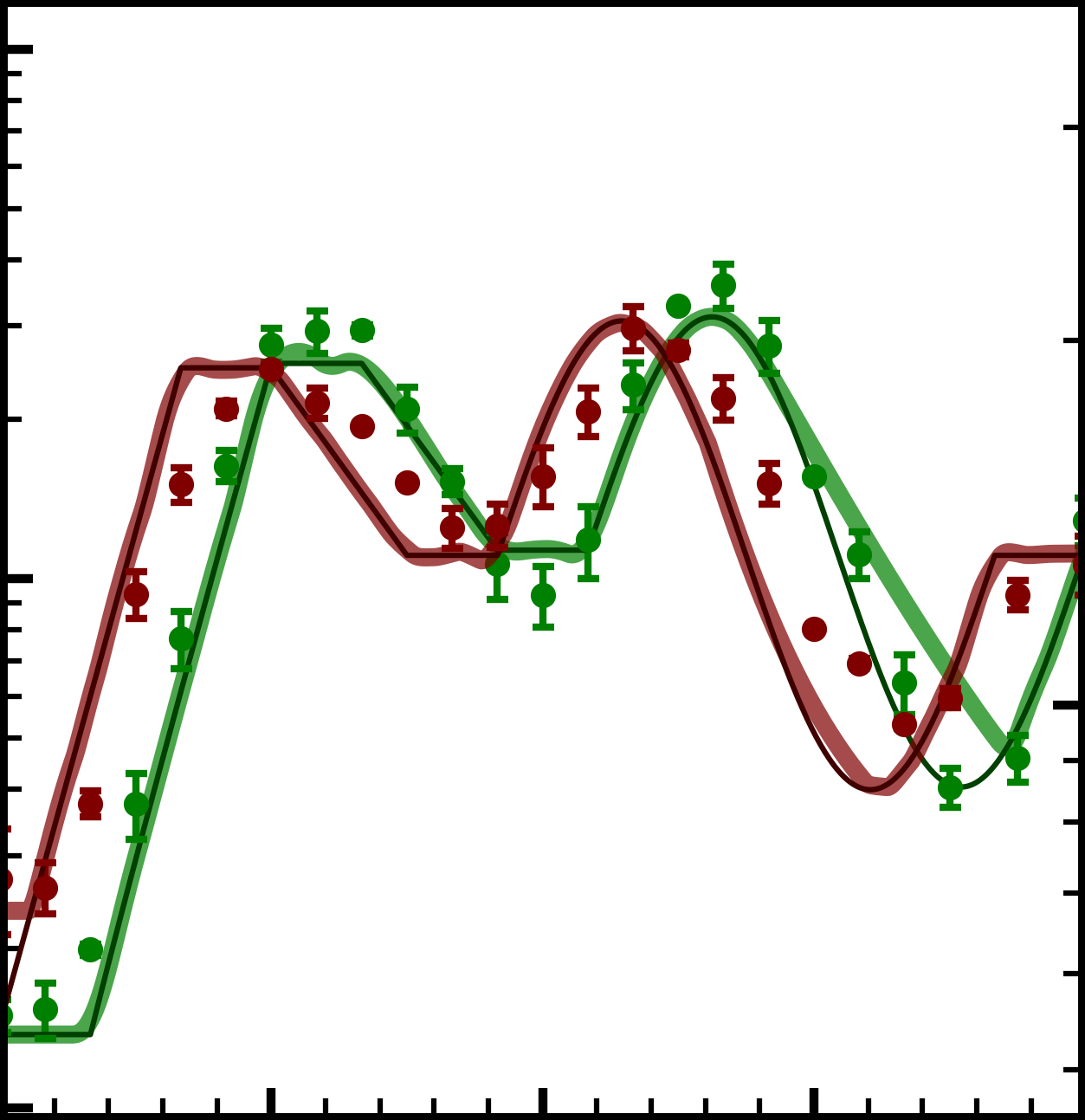

Supplement: Supplementary file 16 — Dataset EV8 [file MSB-13-926-s016.zip › dataset_ev8_mux_data_and_analysis/mux_analysis/plots/Janeway_logy_full_data.png]

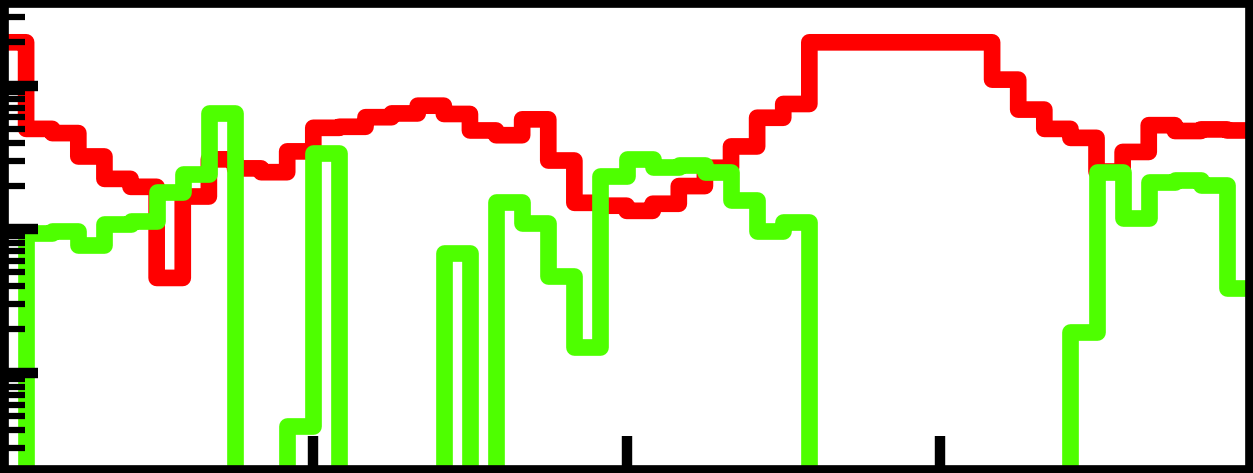

Supplement: Supplementary file 16 — Dataset EV8 [file MSB-13-926-s016.zip › dataset_ev8_mux_data_and_analysis/mux_analysis/plots/Janeway_logy_full_intlog.png]

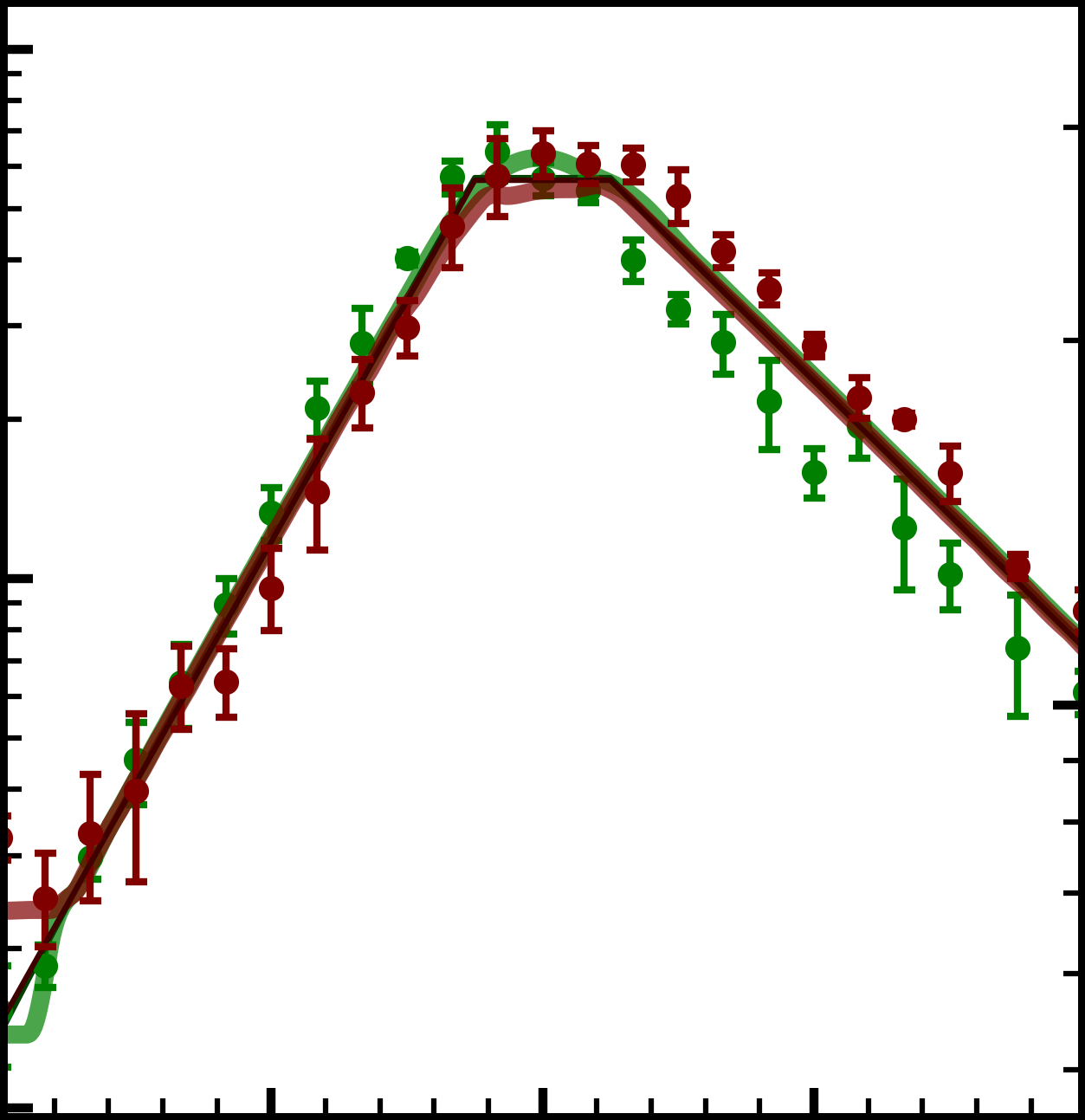

Supplement: Supplementary file 16 — Dataset EV8 [file MSB-13-926-s016.zip › dataset_ev8_mux_data_and_analysis/mux_analysis/plots/Jennie_logy_full_data.png]

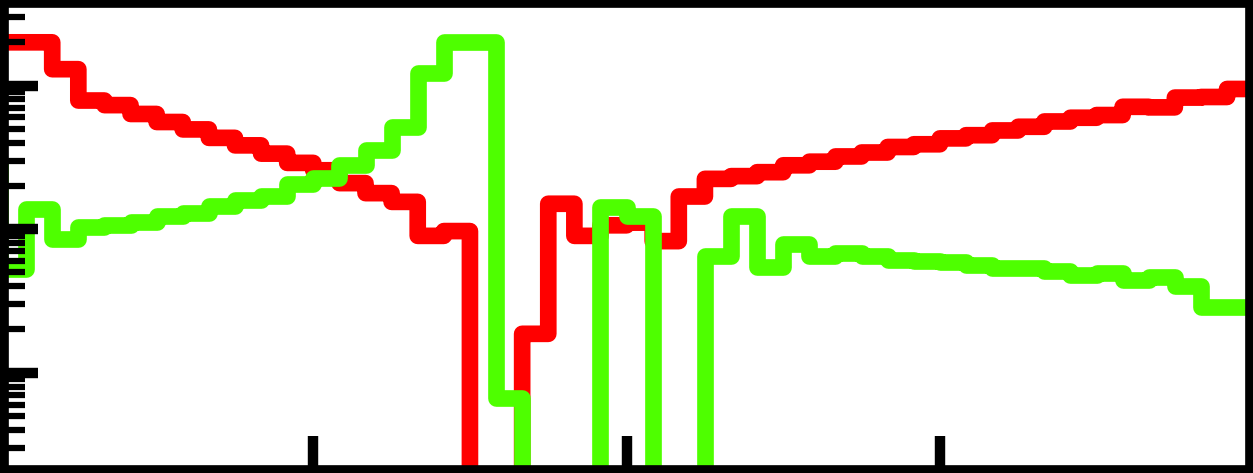

Supplement: Supplementary file 16 — Dataset EV8 [file MSB-13-926-s016.zip › dataset_ev8_mux_data_and_analysis/mux_analysis/plots/Jennie_logy_full_intlog.png]

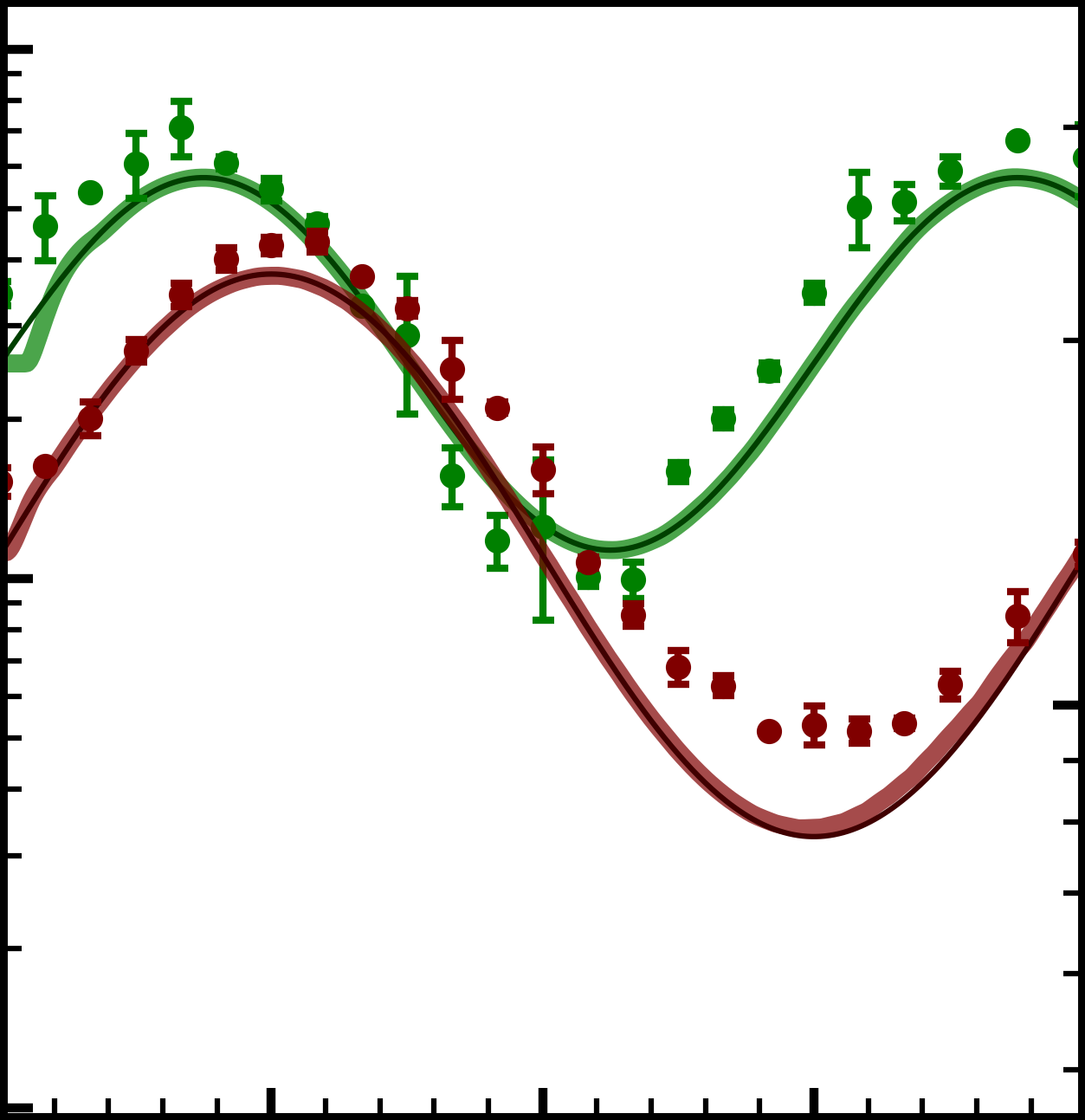

Supplement: Supplementary file 16 — Dataset EV8 [file MSB-13-926-s016.zip › dataset_ev8_mux_data_and_analysis/mux_analysis/plots/Kirk_logy_full_data.png]

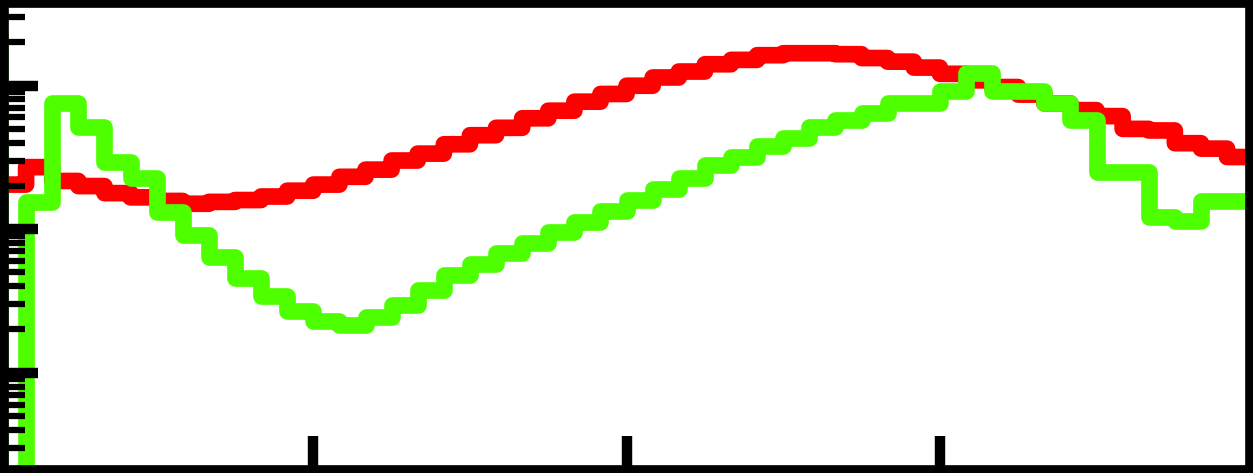

Supplement: Supplementary file 16 — Dataset EV8 [file MSB-13-926-s016.zip › dataset_ev8_mux_data_and_analysis/mux_analysis/plots/Kirk_logy_full_intlog.png]

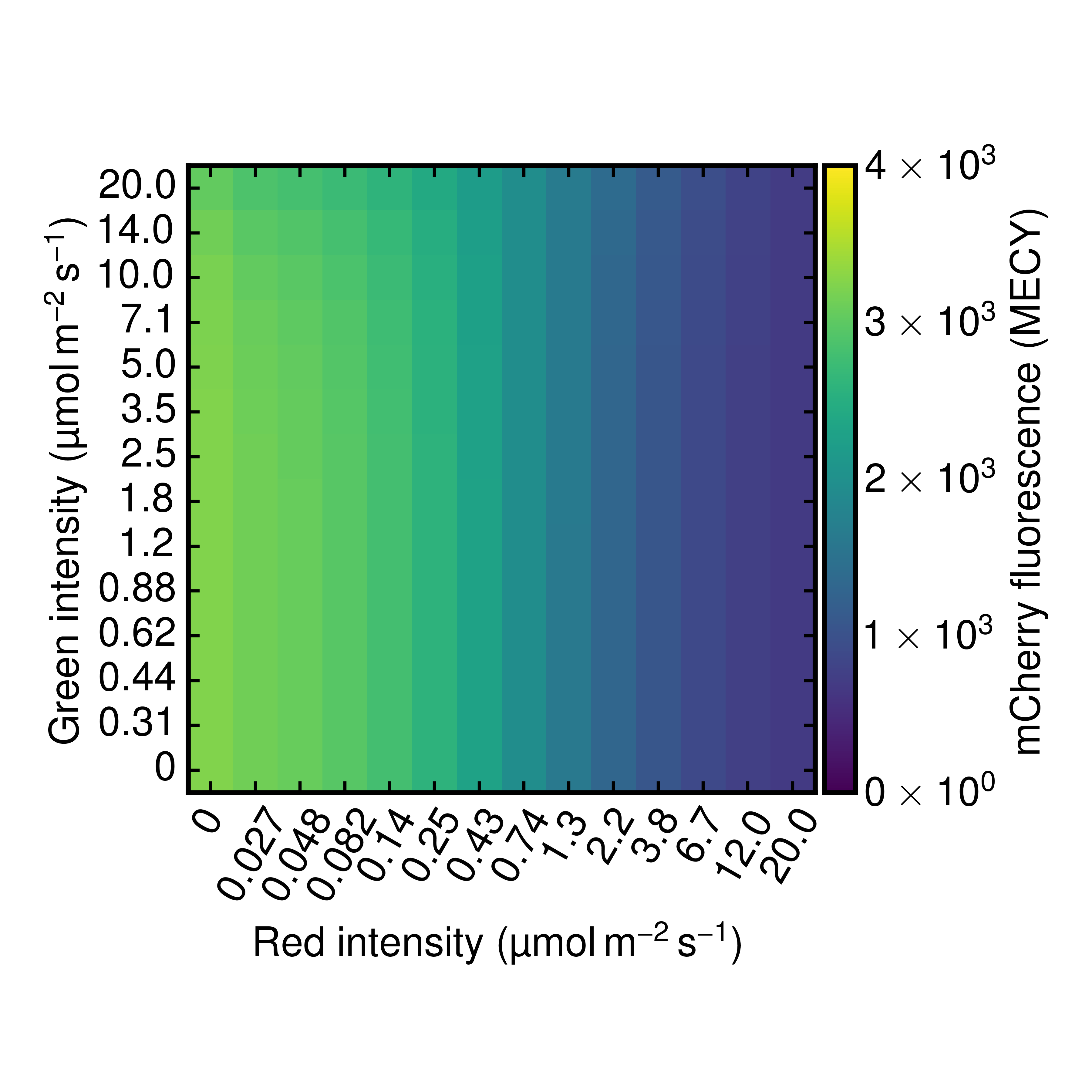

Supplement: Supplementary file 16 — Dataset EV8 [file MSB-13-926-s016.zip › dataset_ev8_mux_data_and_analysis/mux_analysis/plots/mcherry_lin_model_heatmap.png]

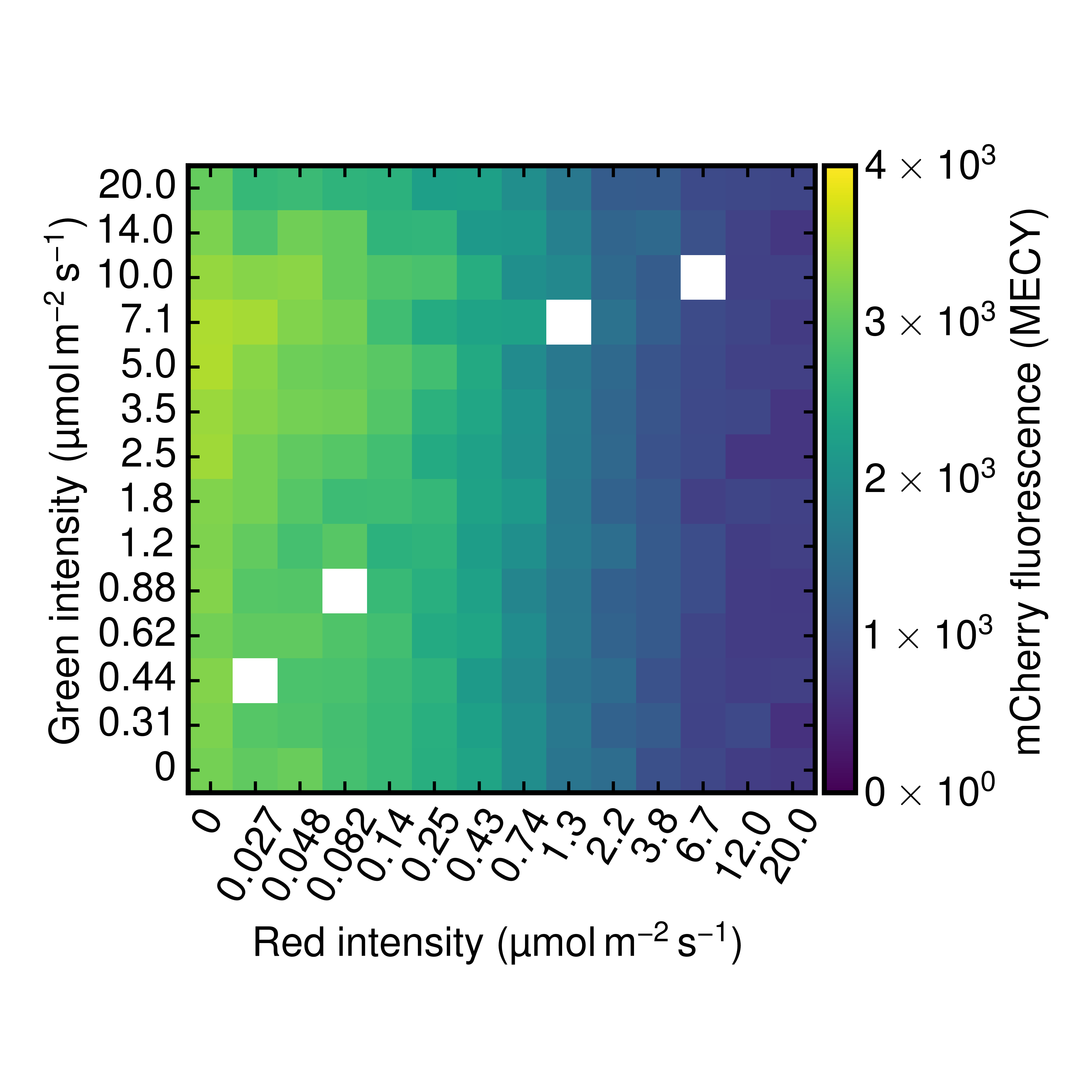

Supplement: Supplementary file 16 — Dataset EV8 [file MSB-13-926-s016.zip › dataset_ev8_mux_data_and_analysis/mux_analysis/plots/mcherry_lin_raw_heatmap.png]

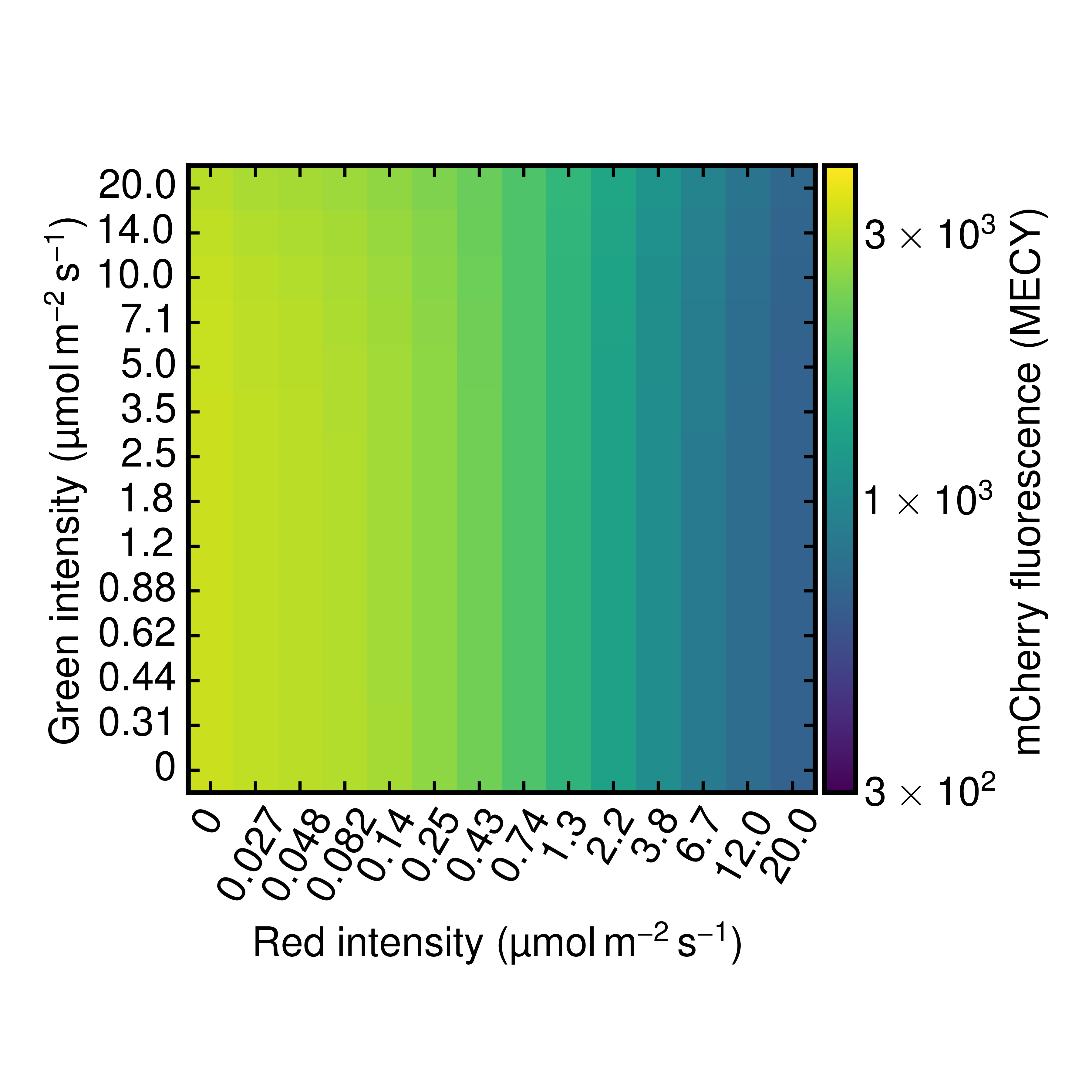

Supplement: Supplementary file 16 — Dataset EV8 [file MSB-13-926-s016.zip › dataset_ev8_mux_data_and_analysis/mux_analysis/plots/mcherry_logz_model_heatmap.png]

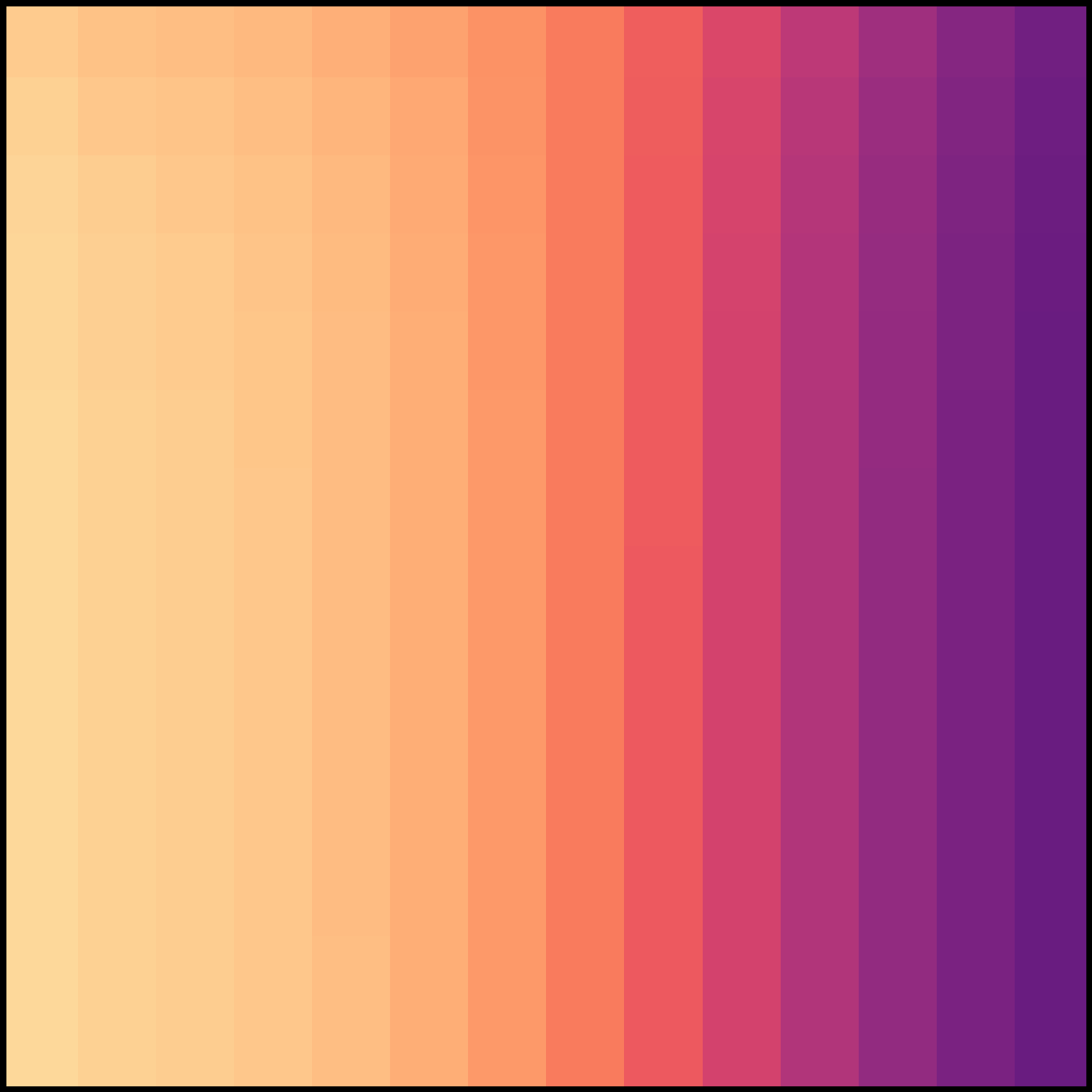

Supplement: Supplementary file 16 — Dataset EV8 [file MSB-13-926-s016.zip › dataset_ev8_mux_data_and_analysis/mux_analysis/plots/mcherry_logz_model_nolabel_heatmap.png]

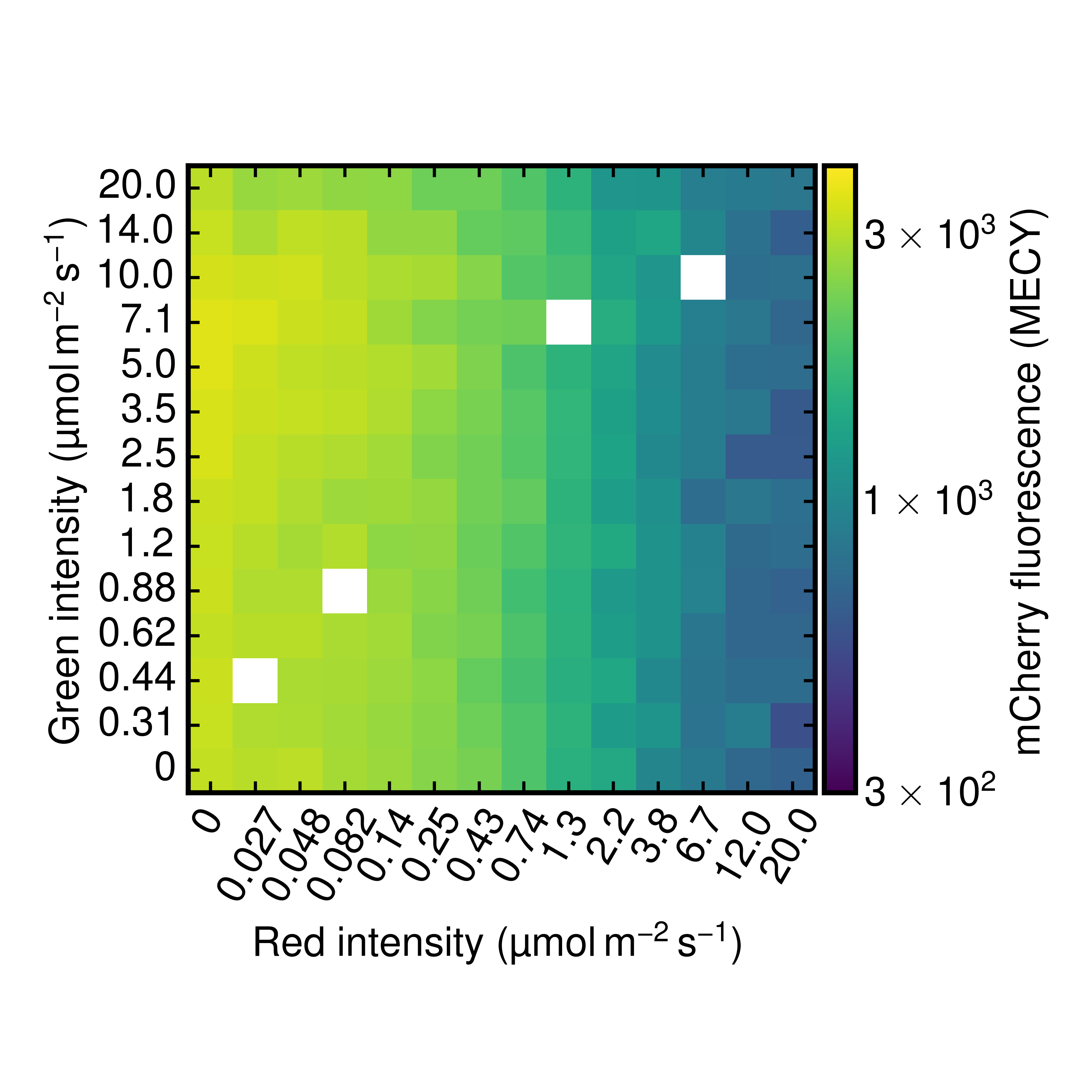

Supplement: Supplementary file 16 — Dataset EV8 [file MSB-13-926-s016.zip › dataset_ev8_mux_data_and_analysis/mux_analysis/plots/mcherry_logz_raw_heatmap.png]

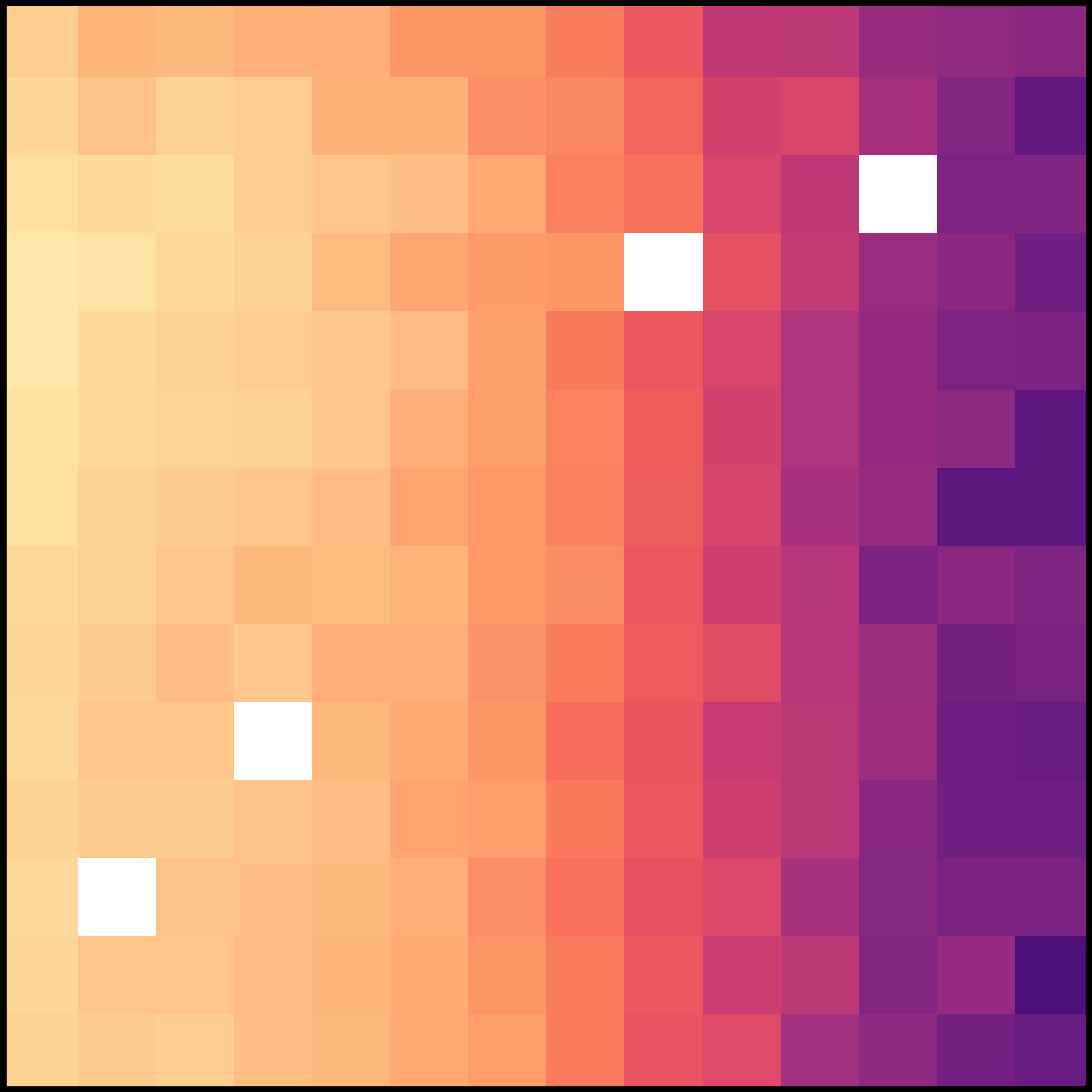

Supplement: Supplementary file 16 — Dataset EV8 [file MSB-13-926-s016.zip › dataset_ev8_mux_data_and_analysis/mux_analysis/plots/mcherry_logz_raw_nolabel_heatmap.png]

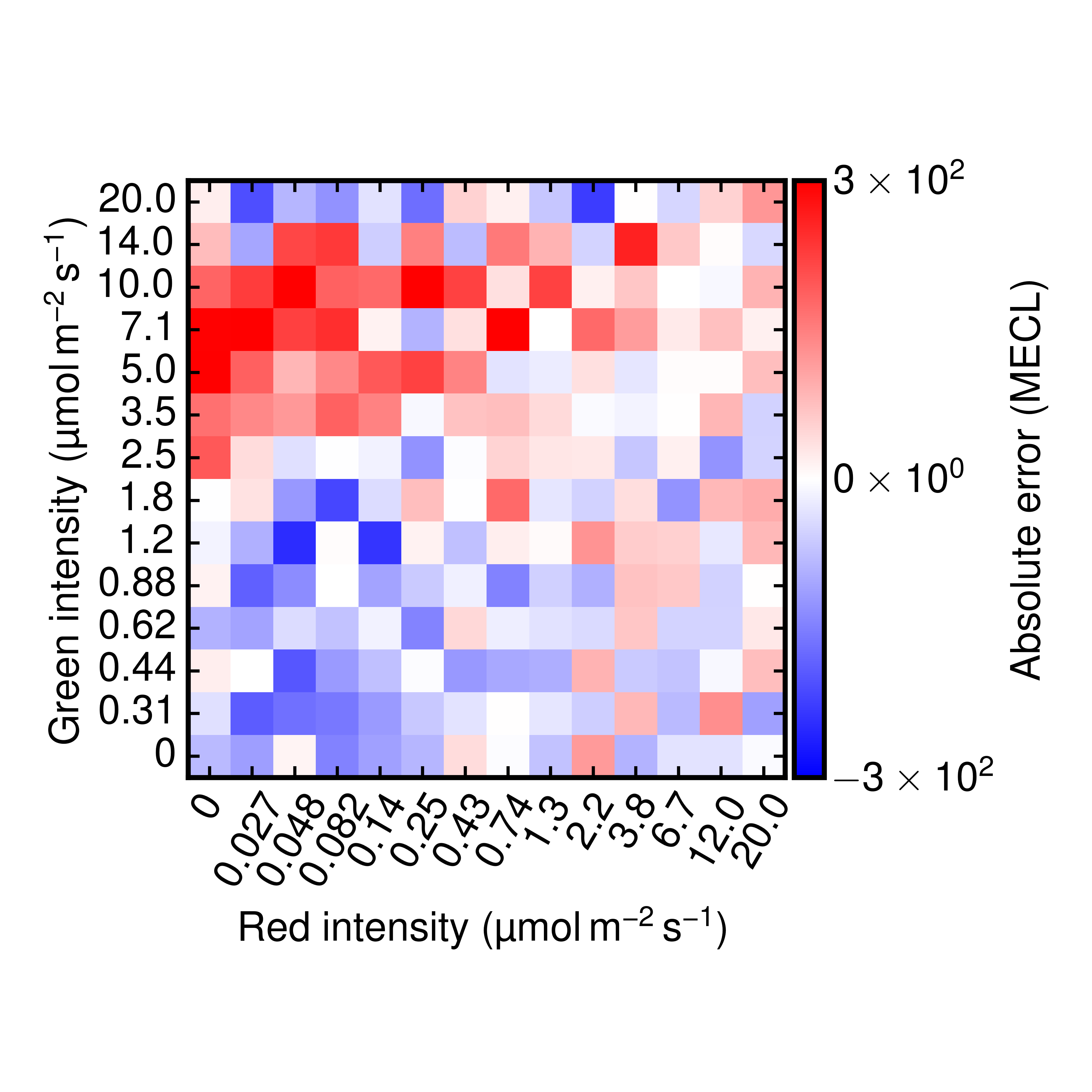

Supplement: Supplementary file 16 — Dataset EV8 [file MSB-13-926-s016.zip › dataset_ev8_mux_data_and_analysis/mux_analysis/plots/mch_abs_residual_hmap.png]

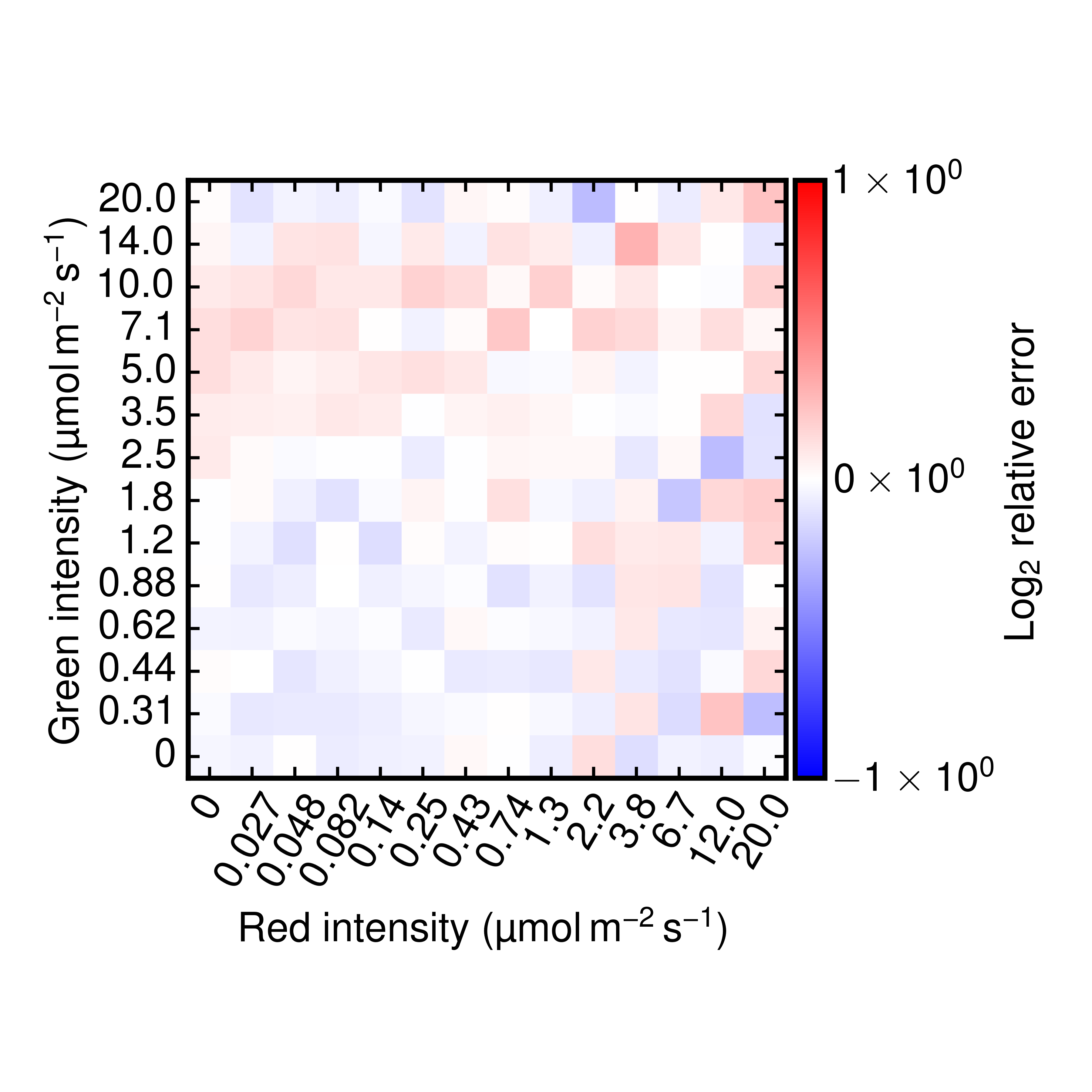

Supplement: Supplementary file 16 — Dataset EV8 [file MSB-13-926-s016.zip › dataset_ev8_mux_data_and_analysis/mux_analysis/plots/mch_rel_residual_hmap.png]

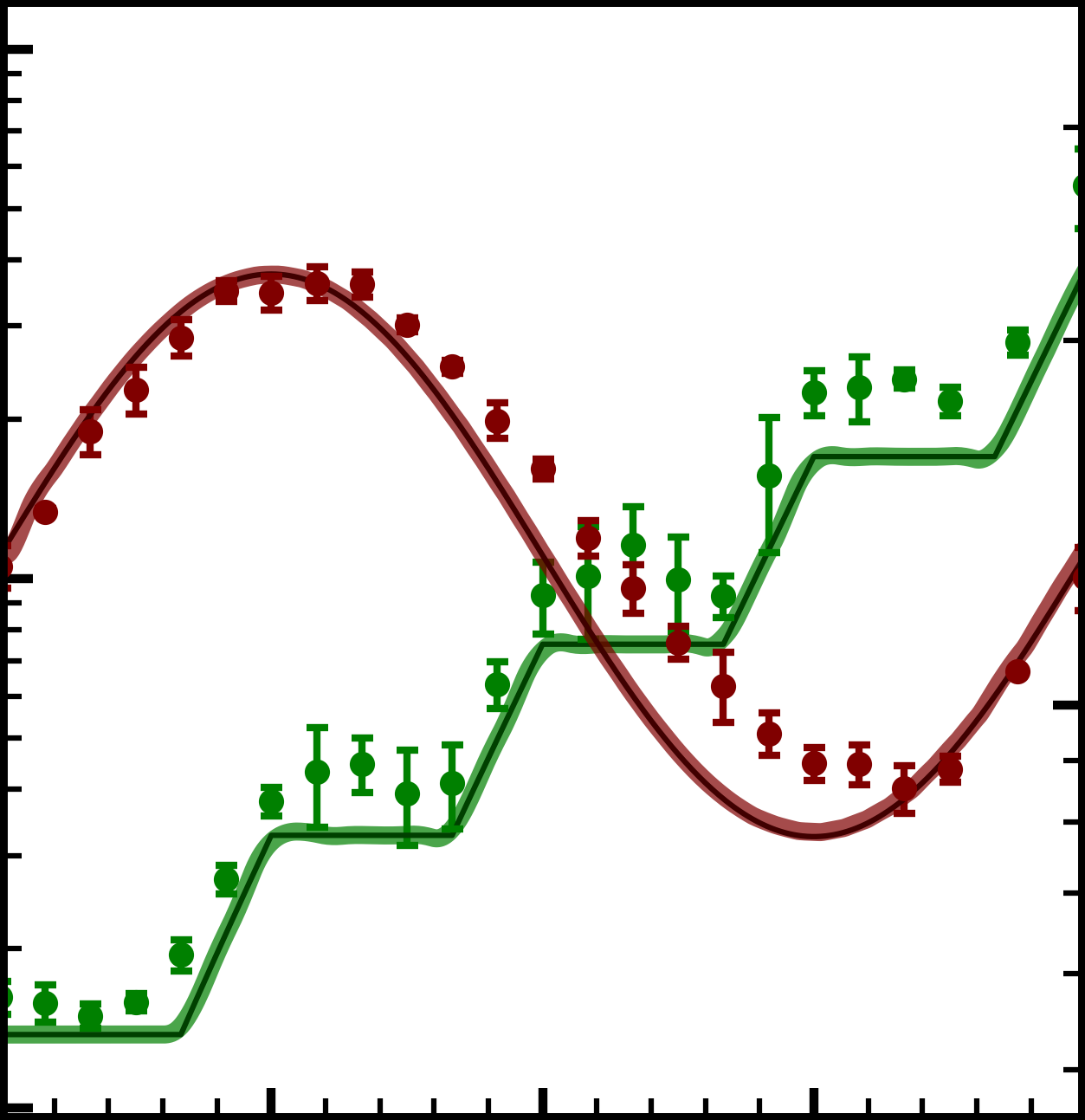

Supplement: Supplementary file 16 — Dataset EV8 [file MSB-13-926-s016.zip › dataset_ev8_mux_data_and_analysis/mux_analysis/plots/Picard_logy_full_data.png]

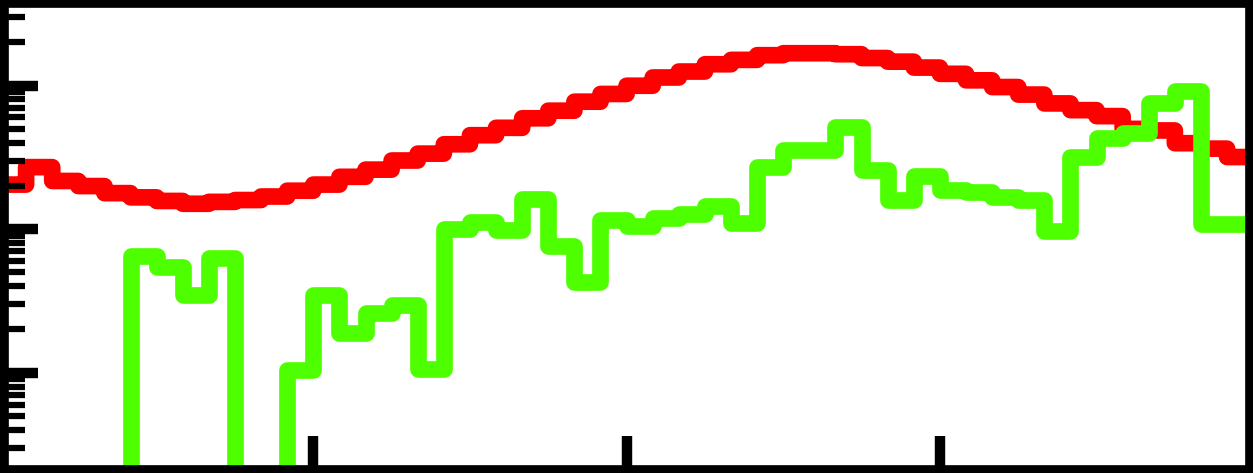

Supplement: Supplementary file 16 — Dataset EV8 [file MSB-13-926-s016.zip › dataset_ev8_mux_data_and_analysis/mux_analysis/plots/Picard_logy_full_intlog.png]

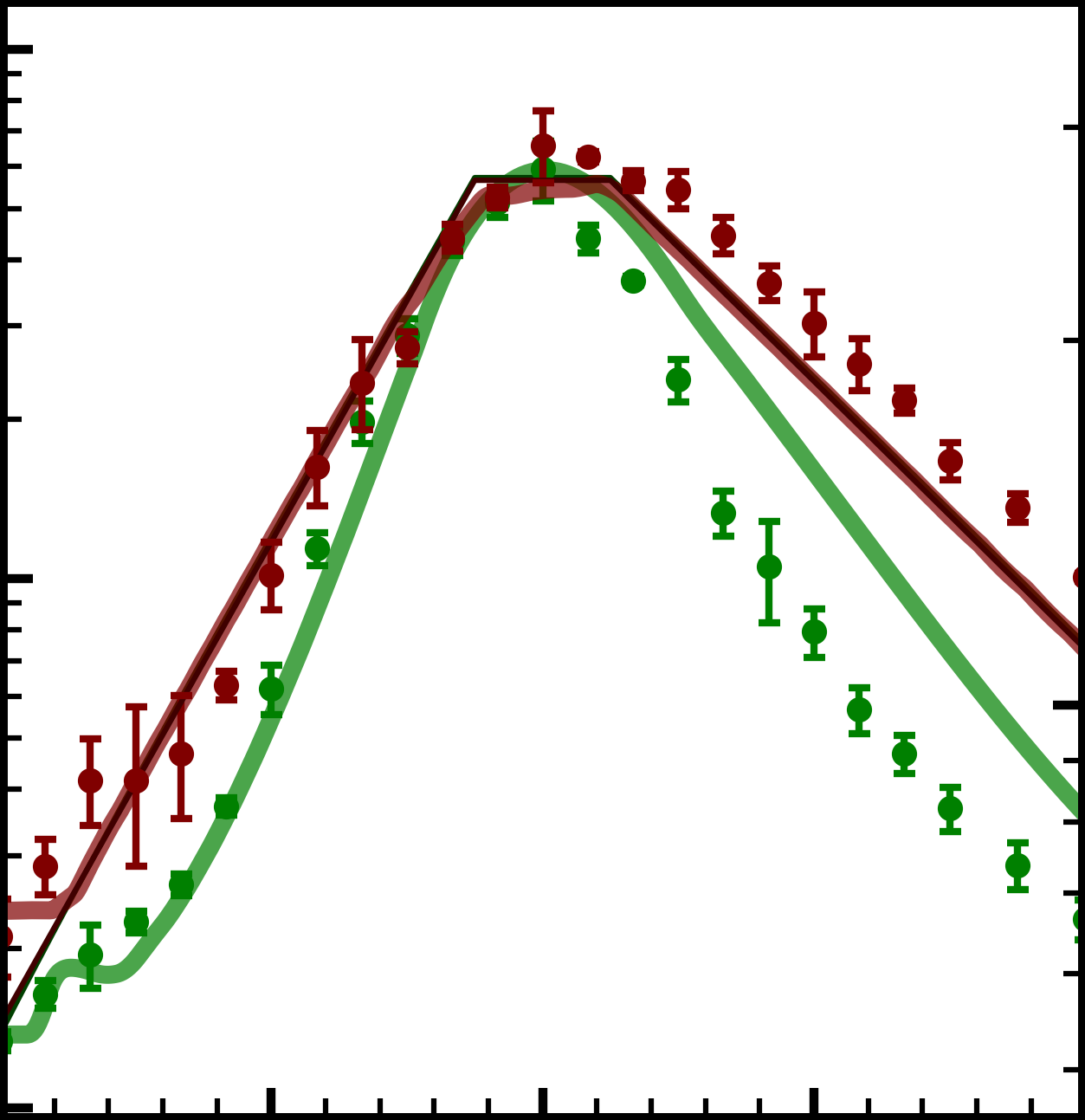

Supplement: Supplementary file 16 — Dataset EV8 [file MSB-13-926-s016.zip › dataset_ev8_mux_data_and_analysis/mux_analysis/plots/Shannen_logy_full_data.png]

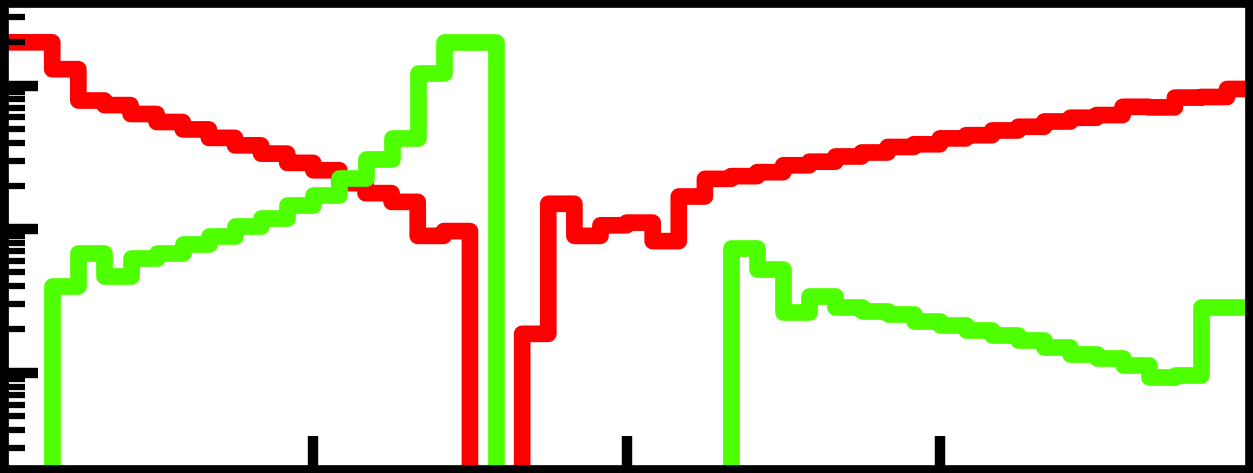

Supplement: Supplementary file 16 — Dataset EV8 [file MSB-13-926-s016.zip › dataset_ev8_mux_data_and_analysis/mux_analysis/plots/Shannen_logy_full_intlog.png]

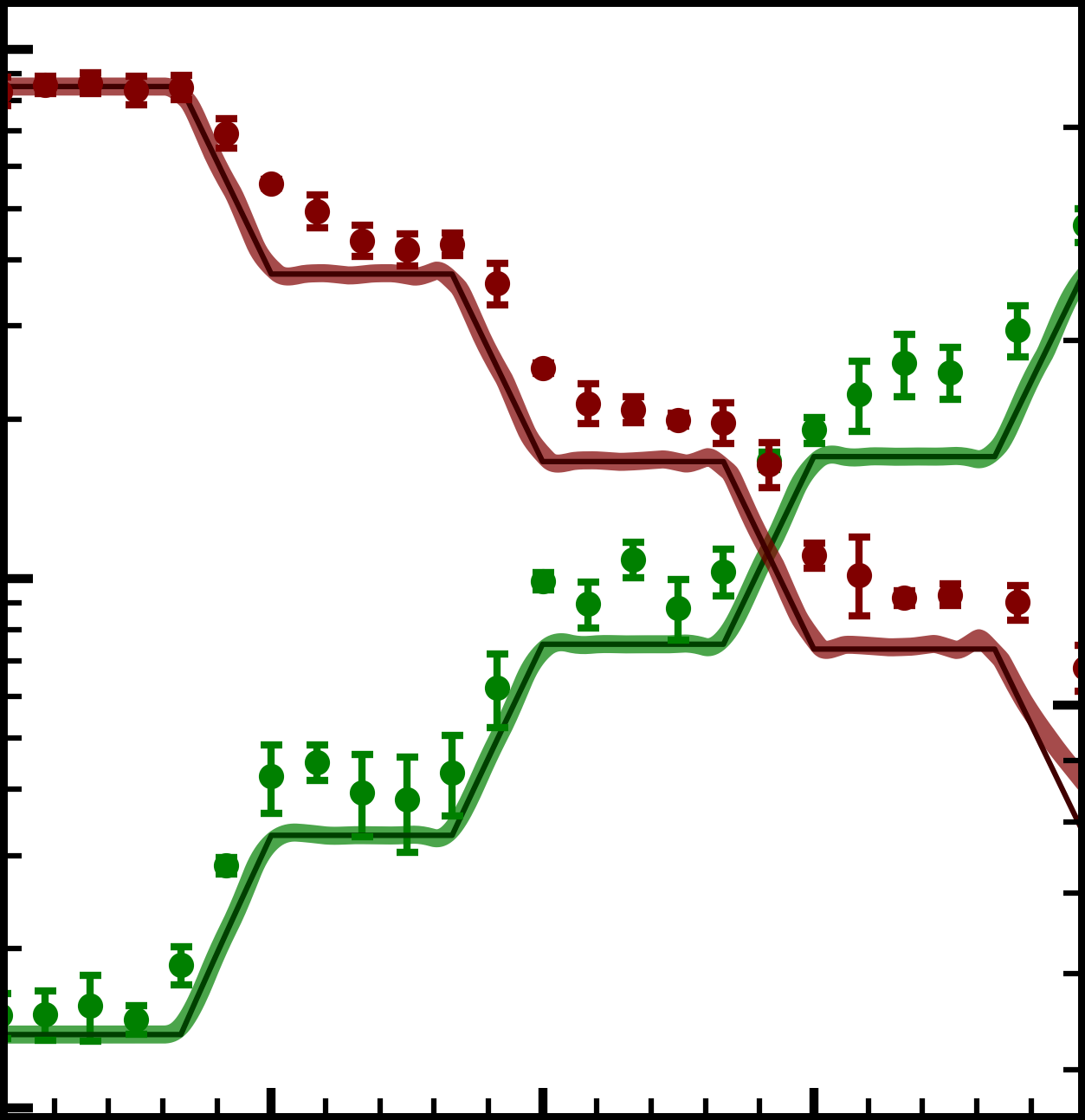

Supplement: Supplementary file 16 — Dataset EV8 [file MSB-13-926-s016.zip › dataset_ev8_mux_data_and_analysis/mux_analysis/plots/Sisko_logy_full_data.png]

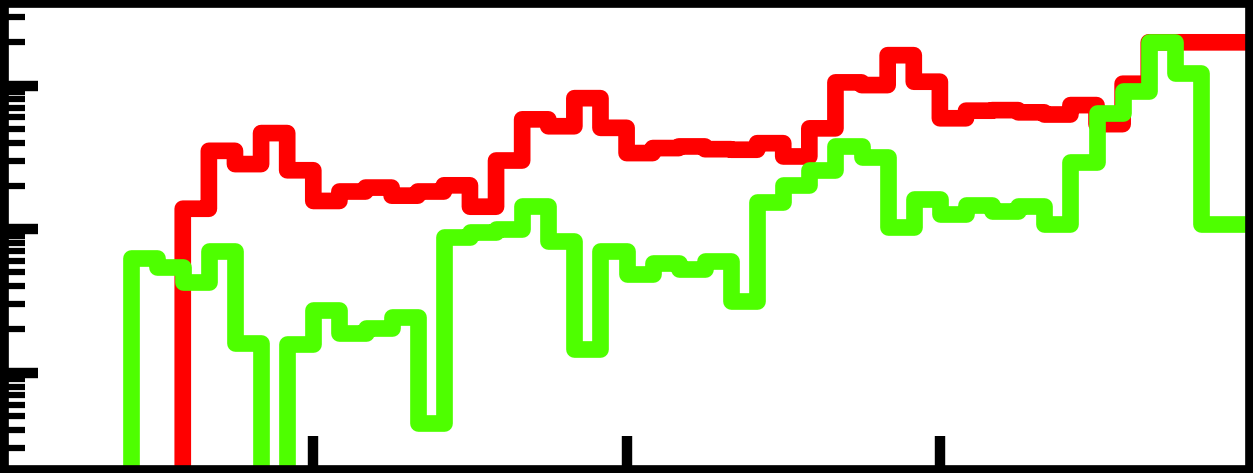

Supplement: Supplementary file 16 — Dataset EV8 [file MSB-13-926-s016.zip › dataset_ev8_mux_data_and_analysis/mux_analysis/plots/Sisko_logy_full_intlog.png]

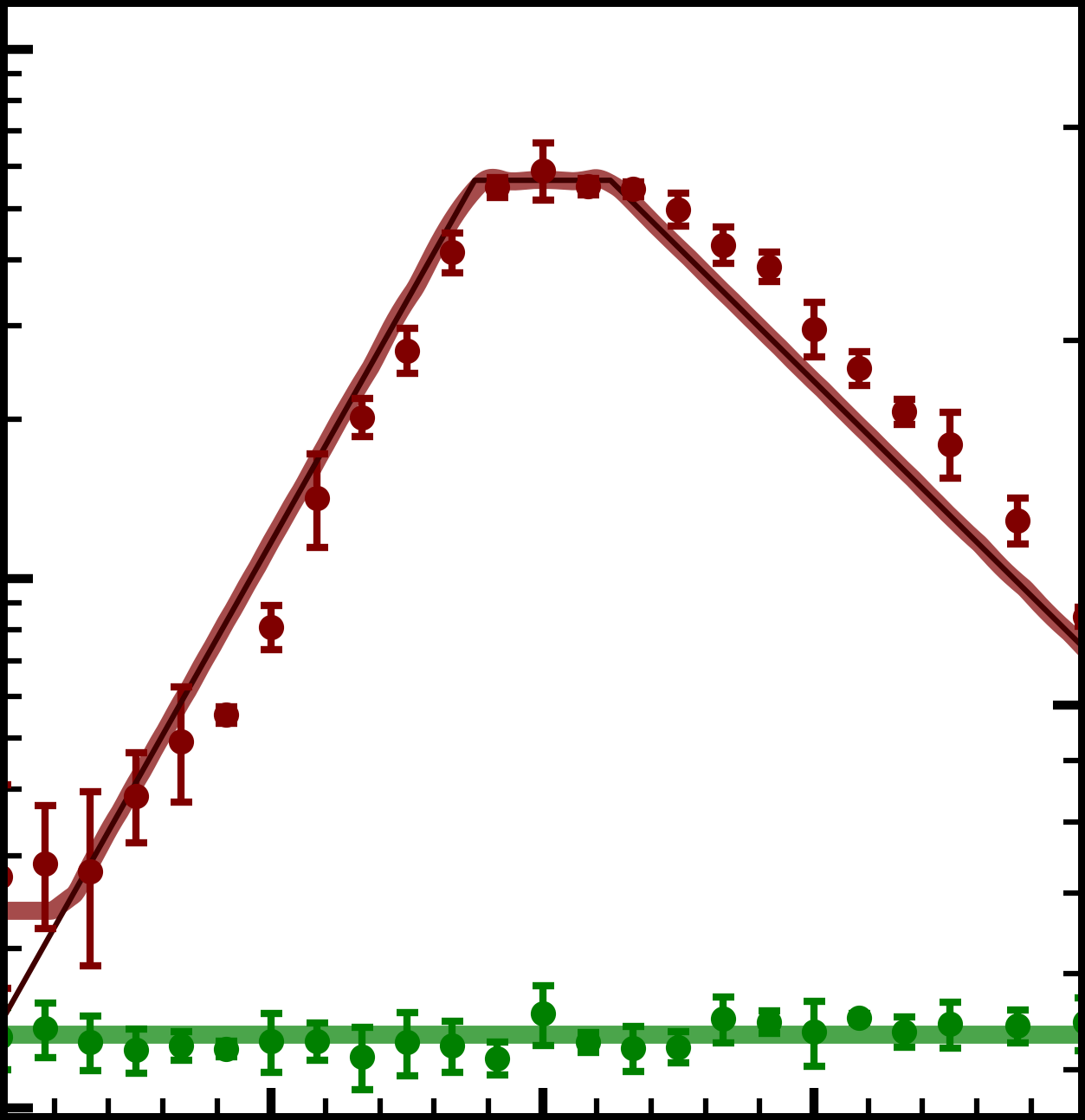

Supplement: Supplementary file 16 — Dataset EV8 [file MSB-13-926-s016.zip › dataset_ev8_mux_data_and_analysis/mux_analysis/plots/Tiffani_logy_full_data.png]

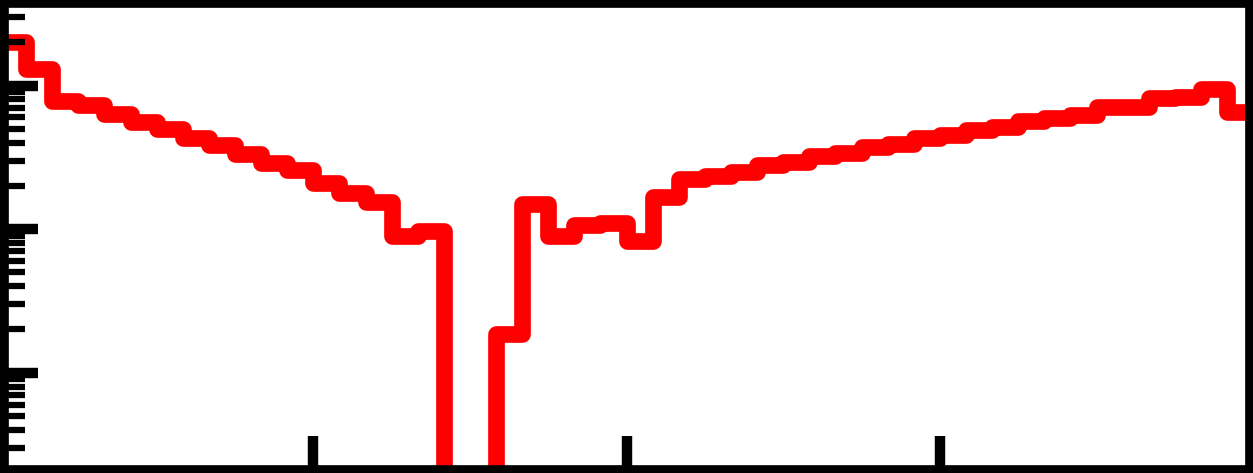

Supplement: Supplementary file 16 — Dataset EV8 [file MSB-13-926-s016.zip › dataset_ev8_mux_data_and_analysis/mux_analysis/plots/Tiffani_logy_full_intlog.png]

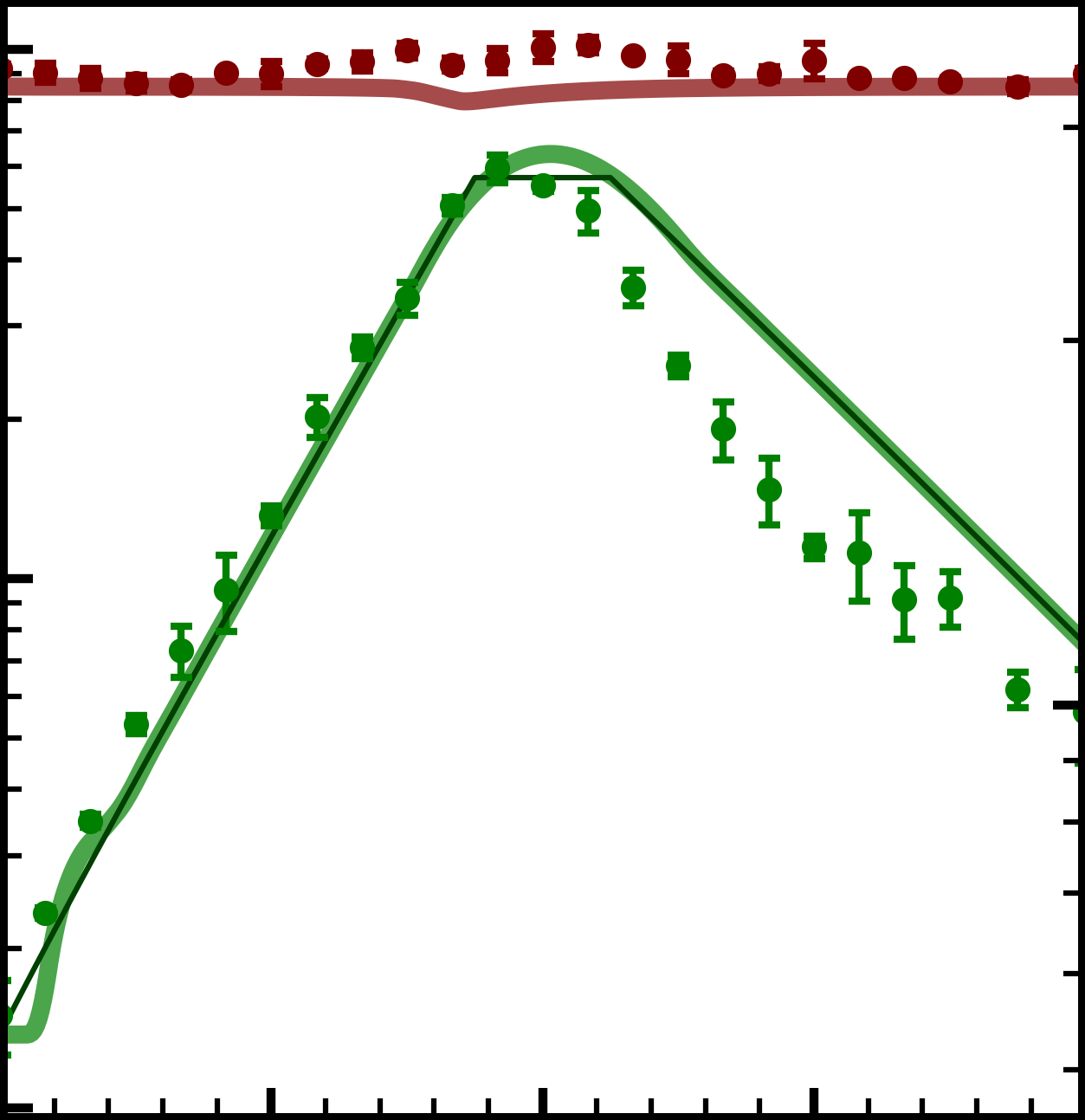

Supplement: Supplementary file 16 — Dataset EV8 [file MSB-13-926-s016.zip › dataset_ev8_mux_data_and_analysis/mux_analysis/plots/Tori_logy_full_data.png]

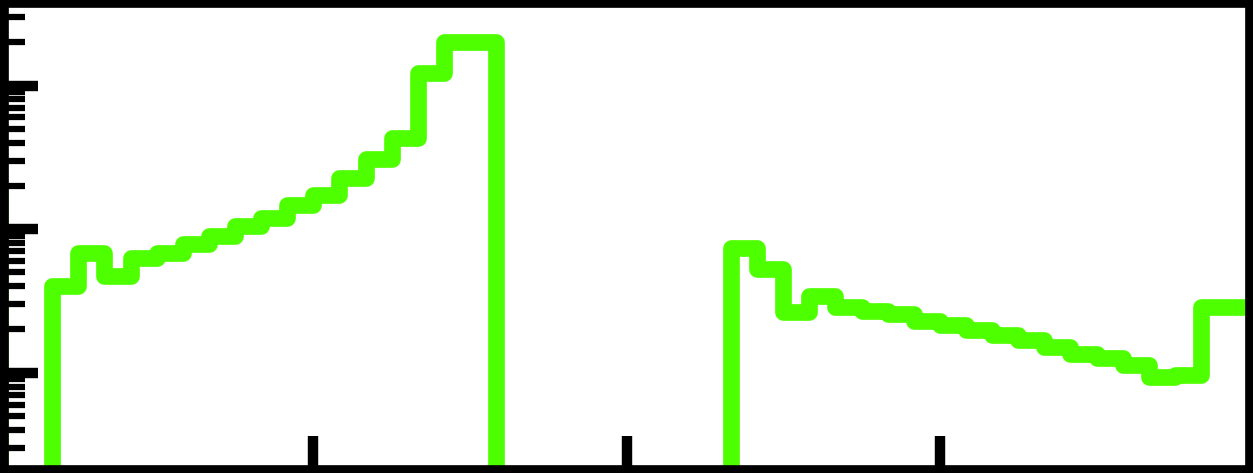

Supplement: Supplementary file 16 — Dataset EV8 [file MSB-13-926-s016.zip › dataset_ev8_mux_data_and_analysis/mux_analysis/plots/Tori_logy_full_intlog.png]

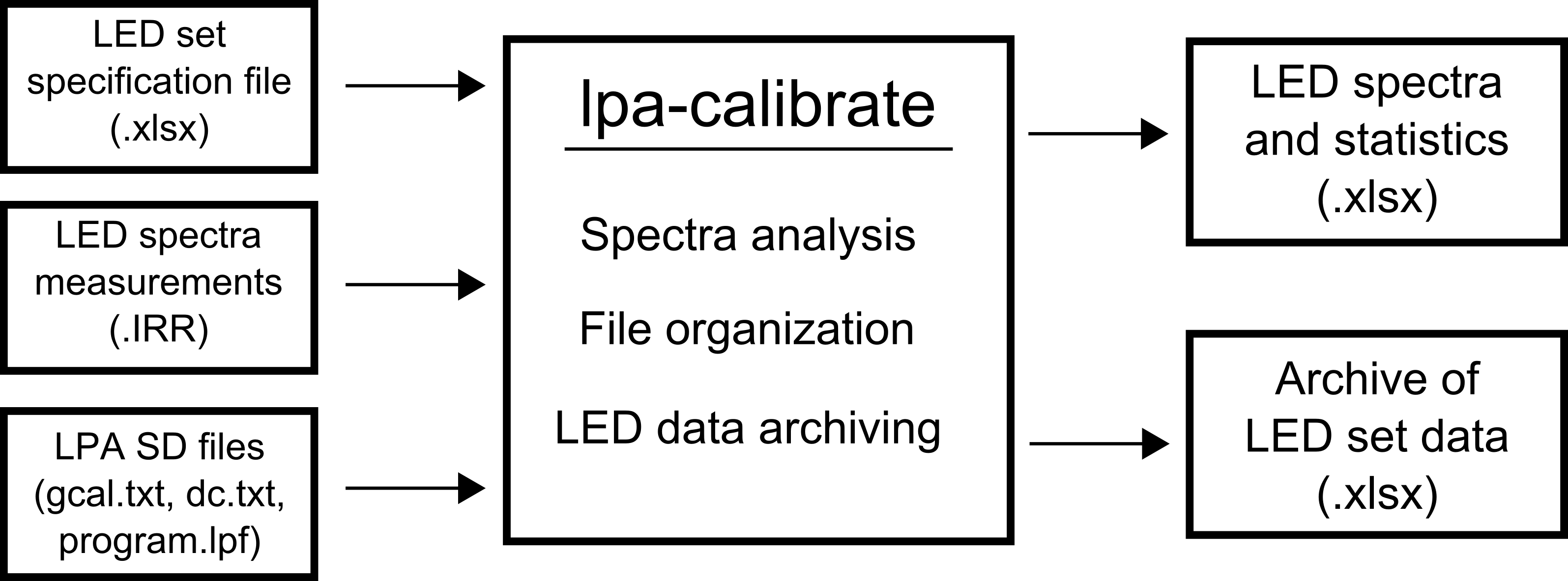

Supplement: Supplementary file 17 — Dataset EV9 [file MSB-13-926-s017.zip › dataset_ev9_lpa-tools/figures/lpa-calibrate.png]

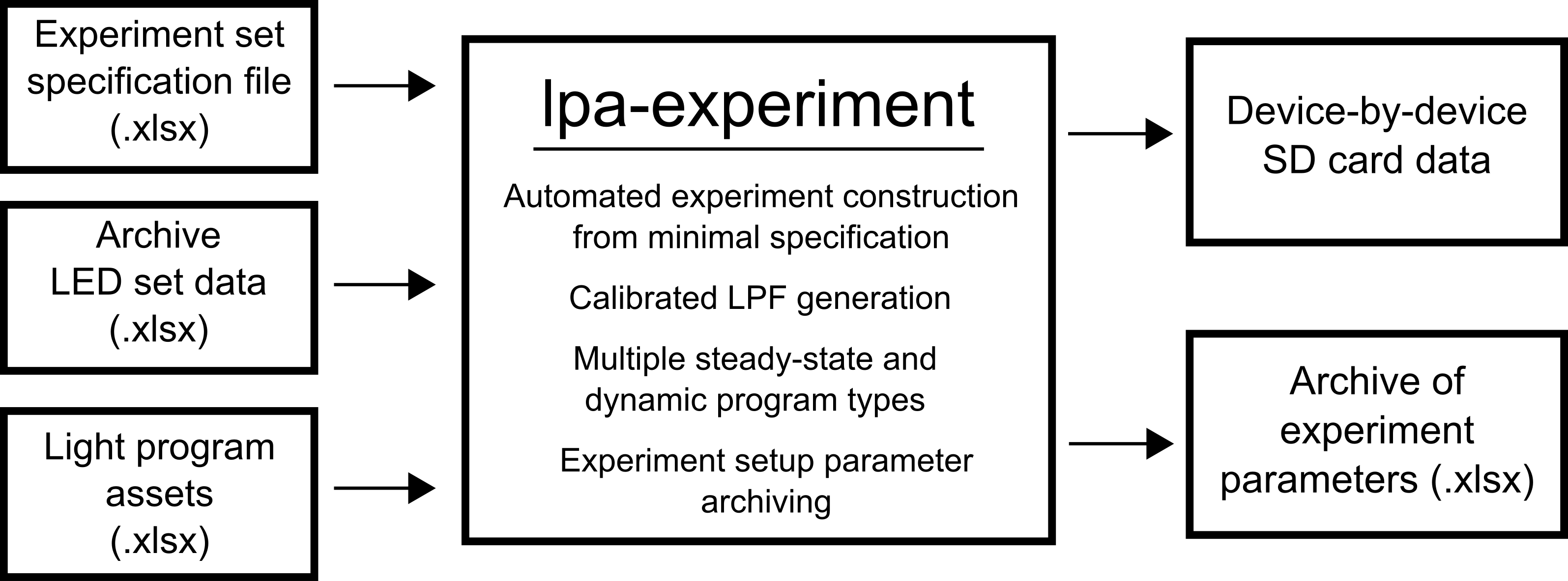

Supplement: Supplementary file 17 — Dataset EV9 [file MSB-13-926-s017.zip › dataset_ev9_lpa-tools/figures/lpa-experiment.png]

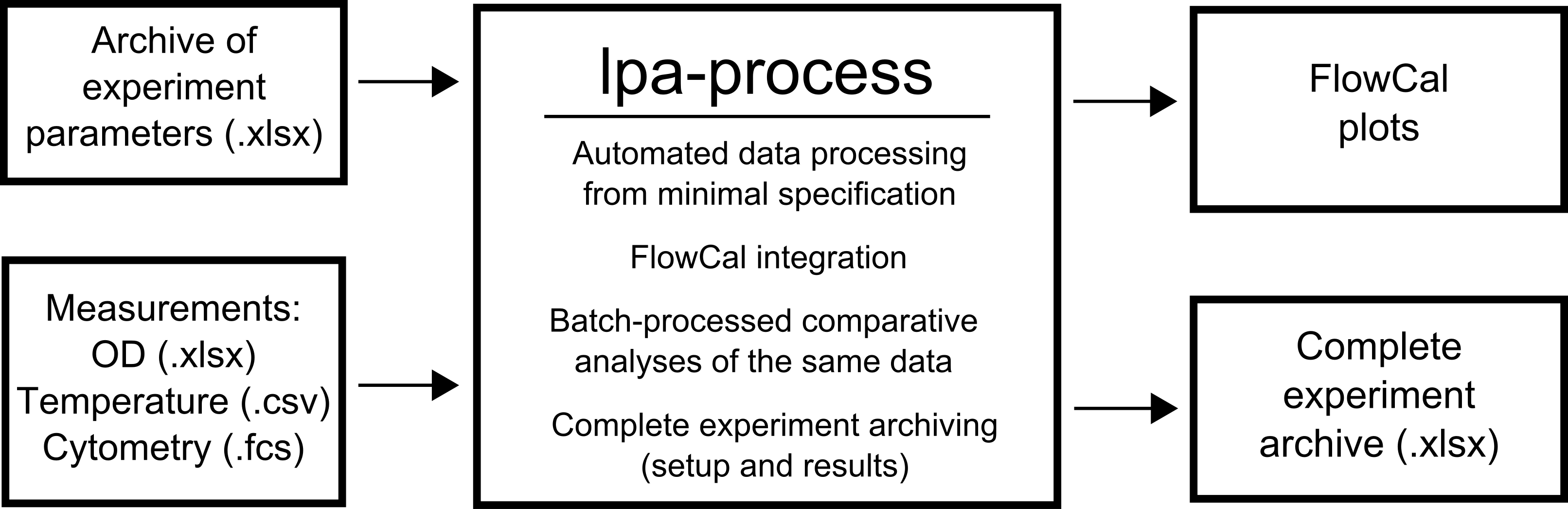

Supplement: Supplementary file 17 — Dataset EV9 [file MSB-13-926-s017.zip › dataset_ev9_lpa-tools/figures/lpa-process.png]

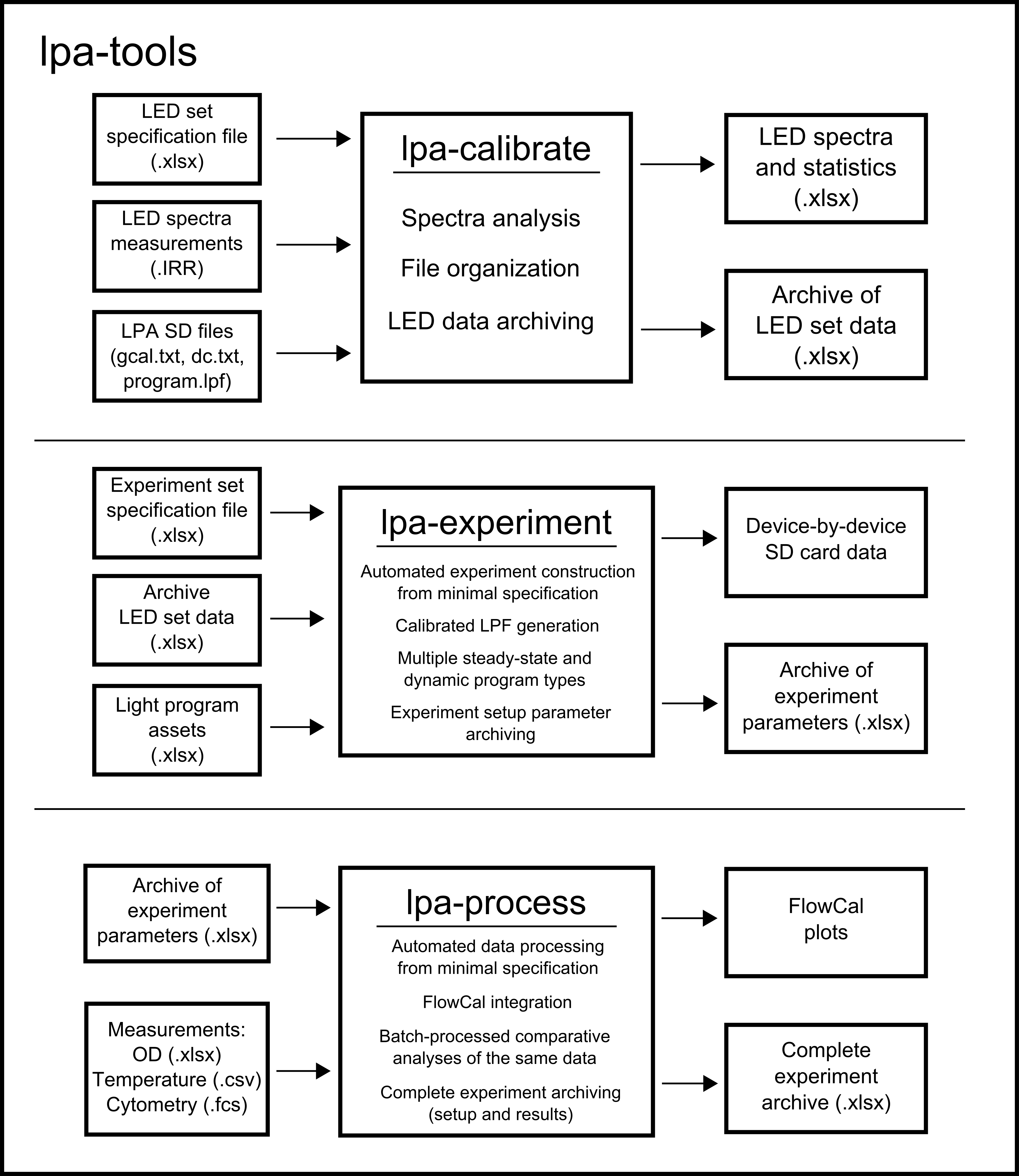

Supplement: Supplementary file 17 — Dataset EV9 [file MSB-13-926-s017.zip › dataset_ev9_lpa-tools/figures/lpa-tools.png]
